# Same-visit HIV testing in Trinidad and Tobago

**DOI:** 10.1186/1471-2458-10-185

**Published:** 2010-04-09

**Authors:** Violet Duke, Sheila Samiel, David Musa, Cameile Ali, Catherine Chang-Kit, Cynthia Warner

**Affiliations:** 1 Ministry of Health, Port of Spain, Trinidad and Tobago; 2 Pan American Health Organization, Bridgetown, Barbados; 3 George Street Health Center, Port-of-Spain, Trinidad and Tobago; 4 Petit Valley, Port of Spain, Trinidad and Tobago; 5 St. Anne's, Port of Spain, Trinidad and Tobago; 6 Centers for Disease Control and Prevention, Atlanta Georgia, USA

## Abstract

**Background:**

The Ministry of Health (hereafter, Ministry) of Trinidad and Tobago is responsible for delivery of all health services in the country. The Ministry takes responsibility for direct delivery of care in the public sector and has initiated a process whereby those seeking HIV test results could obtain confidential reports during a single-visit to a testing location. The Ministry requested technical assistance with this process from the Caribbean Epidemiology Centre (CAREC). The United States Centers for Disease Control and Prevention (CDC) played an important role in this process through its partnership with CAREC.

**Methods:**

Under the technical guidance of CAREC and CDC, the Ministry organized a technical working group which included representatives from key national HIV program services and technical assistance partners. This working group reviewed internationally-recognized best practices for HIV rapid testing and proposed a program that could be integrated into the national HIV programs of Trinidad and Tobago. The working group wrote a consensus protocol, defined certification criteria, prepared training materials and oversaw implementation of "same-visit" HIV testing at two pilot sites.

**Results:**

A Ministry-of-Health-supported program of "same-visit" HIV testing has been established in Trinidad and Tobago. This program provides confidential testing that is independent of laboratory confirmation. The program allows clients who want to know their HIV status to obtain this information during a single-visit to a testing location. Testers who are certified to provide testing on behalf of the Ministry are also counselors. Non-laboratory personnel have been trained to provide HIV testing in non-laboratory locations. The program includes procedures to assure uniform quality of testing across multiple testing sites. Several procedural and training documents were developed during implementation of this program. This report contains links to those documents.

**Conclusions:**

The Ministry of Health has implemented a program of "same-visit" HIV testing in Trinidad and Tobago. This program provides clients confidential HIV test reports during a single visit to a testing location. The program is staffed by non-laboratory personnel who are trained to provide both testing and counseling in decentralized (non-laboratory) settings. This approach may serve as a model for other small countries.

## Background

### Overview of HIV Testing

#### Technology for testing

In 1981, the index case for what is now known as Acquired Immuno-deficiency Syndrome (AIDS) was reported [1]. Within a few years, the Human Immuno-deficiency Virus (HIV) had been identified as the infectious agent that causes AIDS [2]. The laboratory work that led to the identification of HIV as the infectious agent of AIDS also provided a foundation for development of HIV testing. By the mid-1980s, laboratory tests which could detect HIV antibody had been developed [2].

During the 1990s, HIV testing technology exploded. This technology explosion included an array of HIV rapid test devices. Rapid tests are instrument-free assays designed to detect both HIV antibody and HIV-associated antigen. Many of these devices have sensitivities and specificities comparable to those of laboratory-based tests and provide the technical basis for decentralization of HIV testing into non-laboratory locations. Test decentralization provides policy-makers with opportunities to expand HIV testing into non-laboratory settings. As with many opportunities, these were more complex than originally imagined.

#### Policy for testing

In 2001, the United Nations General Assembly Special Session (UNGASS) on HIV/AIDS asked each of the 189 member countries to prepare individual national responses to the HIV/AIDS epidemic. In response to UNGASS, many countries prepared National Strategic Plans (NSPs) outlining country-specific activities for delivery of HIV/AIDS services. In many places, these NSPs became tools for improvement of existing HIV/AIDS services and introduction of new ones.

During 2001, in the wake of UNGASS, many countries committed to public-health delivery of anti-retroviral therapy (ART) services and some countries adopted the goal of Universal Access to ART. In this context, scale-up of HIV testing services became pivotal. In addition, in December 2003, the World Health Organization (WHO) announced the 3 by 5 Initiative [3] in which three million people would have access to ART services by December 2005. In this Initiative, HIV testing was described as the "entry point" to all other HIV services, including ART. This Initiative focused increased attention on plans and policies to expand and decentralize HIV testing.

By 2003, both growing availability and acceptance of HIV rapid testing technology and increasing availability of drugs for ART encouraged policy-maker interest in point-of-service HIV testing. Ideally, HIV rapid testing would be used to allow individuals who wanted to know their HIV status to obtain that information during a single-visit (or the "same-visit") to a testing location. Reports indicate that clients who receive HIV status reports during the "same-visit" to an HIV testing location are much more likely to obtain their test results than those who must return for a "second-visit" days or weeks later to receive results obtained after laboratory-based testing. "Same-visit" HIV-testing also increases client confidence in, and acceptance of, HIV testing [4].

Rapid HIV test technology could have significant impact on control of the HIV/AIDS epidemic. When HIV testing is more accessible, more people learn their HIV-infection status. When the "entry point" to ART is readily accessible, those eligible for ART experience fewer obstacles to service delivery. Policy advisors face significant challenges in addressing questions about HIV testing policy. Integrating the most appropriate technical options for HIV testing into effective government policies for prevention, care and treatment of HIV/AIDS has proven complex [5].

#### Policy development

Policies concerning scale-up of HIV testing based on HIV rapid test technology must address several questions:

1. Which rapid tests will be used?

2. What algorithm will be used to determine the HIV status of a client?

3. Who will provide testing?

4. How will testers be trained?

5. Will testing always include counseling?

6. Will testers and counselors be the same person?

7. How will quality assurance be integrated into the testing process?

8. Will HIV test results be incorporated into national HIV/AIDS surveillance reports?

None of these questions has either a single or a simple answer. Different policy makers have taken different approaches on each of them. Both international and regional guidance on these questions is limited. Thus far, individual countries intending to introduce HIV rapid testing have had to consider these questions independently. Obtaining evidence-based responses for design of national testing policies has been difficult.

Consensus responses to difficult questions like those listed above provide the foundation for effective policy. Effective policy addressing HIV rapid testing benefits many programs. In countries with effective policies, non-governmental organizations (NGOs) can provide HIV rapid testing in accordance with national policy. National policy for HIV testing requires input from many stakeholders. This report summarizes experiences from one country, Trinidad and Tobago (TT) with implementation of effective national policy for HIV testing using rapid test technology. Before describing the policy development process in TT, a review of HIV testing there is appropriate.

### HIV/AIDS in Trinidad & Tobago

Trinidad and Tobago (TT) is a twin-island Caribbean nation at the southern-most end of the Lesser Antilles. The population of TT is approximately 1.3 million. The first cases of AIDS in TT were reported in 1983 [6]. UNAIDS characterizes the HIV/AIDS epidemic in TT as having a prevalence of 2.6%, primarily transmitted by heterosexual intercourse, and generalized [7]. These numbers indicate that perhaps 30,000 people in TT are sero-positive for HIV infection. At the end of 2007, approximately 4000 patients were receiving ART services provided by the MOH.

#### HIV testing in TT

Laboratory-based testing for HIV infection has been available in TT since the 1980s. Although this testing was used primarily for diagnostic purposes, it was not routinely provided. This situation changed in the mid-1990s with the introduction of the Prevention-of-Mother-To-Child-Transmission (PMTCT) Program. This program included HIV testing for all women who attended public ante-natal clinics. When the PMTCT program began, all HIV testing in TT was laboratory based. The diagnostic algorithm for HIV infection in TT was complex and women who provided samples for HIV testing had to wait one to two months for their results. Many women who were tested never obtained their test results.

Dependence on laboratory-based HIV testing presented serious challenges to the PMTCT program. Timely receipt of results was essential to maximize the public health impact of the program. The Ministry wanted to make programmatic and policy transitions that would liberate HIV testing from the laboratory and institute "same-visit", or point-of-service, HIV testing *within *PMTCT clinics. Policy advisors recognized that well-designed, point-of-service HIV testing would benefit other Ministry programs, especially those involving ART.

#### HIV policy in TT

Early in 2004, TT established a National AIDS Coordination Committee (NACC). The primary tasks of this committee were to coordinate and monitor implementation of the NSP for HIV/AIDS. One of the NSP priorities for TT was to expand access to HIV testing. This priority was assigned to the Ministry. The Ministry policy advisors understood that this priority would best be accomplished by transition from HIV testing that required laboratory confirmation to testing that was independent of laboratory confirmation.

Like many other countries, TT has a shortage of trained laboratory personnel. In contrast to many other countries, TT had many counselors who had already been trained in HIV counseling. Thus, in TT, optimal transition from laboratory-based testing would be achieved by training counselors to provide "same-visit" HIV testing. In this situation, one person would serve as both counselor and tester to individuals who requested HIV testing. This approach facilitates decentralization of HIV testing and has been described as both "same-visit" HIV testing and "in-room" HIV testing [4].

In 2004, limited international and regional guidance was available for "same-visit" HIV testing. In September 2004, the Chief Medical Officer of TT requested technical assistance from CAREC with implementation of "same-visit" HIV testing. CAREC is an international organization administered on behalf of 21 CAREC Member Countries. In September 2004, CAREC was a technical partner of the Global AIDS Program (GAP) at CDC. From October 2003 until December 2005, a laboratory advisor was assigned by CDC to the GAP office in Trinidad to provide technical assistance to CAREC.

CAREC, in partnership with CDC, and in consultation with local expertise within both the Ministry and the private sector, developed an implementation protocol. This protocol was accepted and ratified by the Ministry and now provides policy for further scale-up of HIV testing in TT. The remainder of this report describes this process as it unfolded in TT between September 2004 and mid-2007. This report contains links to the documents developed in that process.

## Methods

### Planning process

Implementation of "same-visit" HIV testing in TT was lead by a multidisciplinary technical working group (TWG). The TWG had representation from each administrative entity that would be involved with implementation of "same-visit" HIV testing. Thus the TWG included a broad array of professional expertise: physicians, nurses, laboratory personnel, program managers, counselors and social workers. The TWG also included individuals who provided technical assistance on behalf of both CAREC and CDC/GAP. The TWG diversity incorporated contrasting experiences and expectations about HIV testing. Over time, TWG diversity matured into strength of consensus.

### Role of the TWG

The TWG reviewed and identified international best practices for "same visit" HIV testing. These best practices were incorporated into a protocol (Additional file 1), or technical framework, that specified details for implementation of "same-visit" HIV testing in TT. In addition to the protocol, the TWG drafted several other documents including class-room training materials (Additional file 2), a Reference Manual (Additional file 3) for testers, certification criteria (Additional file 4), and a job description for the Ministry post of Quality Monitor.

### Challenges

The most formidable challenges in protocol design were contained in the terms "quality" and "same-visit" HIV testing. The Ministry request was for technical assistance with a testing program in which one person, who served as both counselor and tester, could report quality test results that were independent of laboratory confirmation. One of the program goals was to allow non-laboratory personnel to report test results from non-laboratory locations. By requesting technical assistance in this way, the Ministry maximized the potential impact of the testing program. The protocol provided a technical framework by which this program goal could be achieved.

### MOH protocol

The TWG reviewed evidence for the Ministry protocol from four sources. The first source was Guyana, another small country in the region. In 2004, Guyana introduced a national program for HIV testing that had many similarities to the program envisioned by Ministry policy advisors in TT. The Ministry of Health Guyana had shared their lessons learned with both CAREC and CDC/GAP. The second source was Brazil; in 2004, Brazil was mid-way in plans to implement national policy for HIV testing. The Brazilian lessons learned provided information from a much larger country in the region. Some of these lessons have since been published [8]. Experiences and data from both of these countries were used to inform TWG decisions. The third source of information was the PMTCT program in TT. This program had begun collecting data about the performance of HIV rapid tests in TT in 1999; these data were especially useful to the TWG. The fourth source included reference documents and recommendations from both WHO and CDC and other published materials. All sources are referenced in the Ministry protocol (Additional file 1).

In addition to referencing source materials, the Ministry protocol also provided detailed information on several technical topics including: 1) a description of routine testing integrated into a quality monitoring system, 2) the rational for "same-visit" HIV testing, 3) the evidence for the Ministry algorithm selection and 4) guidance for operation of the pilot and 5) guidance for expansion to additional sites.

The Ministry protocol also outlines three concepts that facilitated implementation of "same-visit" testing: 1) people who provide testing must be certified, 2) sites which provide testing must be certified; and 3) a Quality Monitor must provide regular oversight of the testing process.

### Key Feature

The most important feature of the protocol is the description of new relationships between laboratories and testers who provide HIV testing on behalf of the Ministry. The biggest challenge faced by the TWG was contained in question 7 above: How will quality assurance be integrated into the testing process? The Ministry wanted clients who sought HIV testing at "same-visit" venues to obtain results comparable in quality and reliability to those they would obtain in laboratory-based venues. The protocol describes how this can be accomplished.

### Certification Process

The essential components of Ministry-certified testing are twofold: site-certification and tester-certification. Site-certification is facility-oriented. The site-readiness checklist (Additional file 5) identifies items that must be ready before testing can begin. Tester-certification is a three-step training program (Additional file 6). Both site- and tester-certification are subject to annual review. The Ministry reserves the right to stop testing in any situation where certification criteria are not met.

### Training Program

The protocol stipulates that only testers who are certified may provide "same-visit" HIV testing on behalf of the Ministry. The TWG devoted considerable attention to creating a training program for tester certification. The classroom materials used in the training program were customized from the HIV Rapid Testing Training Package [9]. The Ministry-customized version of the classroom training materials can be found in Additional file 2. The laboratory training materials were developed by two of the authors of this document (CA and CW) and outlines for these materials are in Additional file 7.

Full certification for Ministry testers is a three-step process (Additional file 6). Each person who is certified to provide testing must complete a training workshop, a laboratory internship and a proficiency exam.

The training workshop is a 3-day event that includes both classroom time (14 hours) and hands-on training (10 hours). During classroom time, workshop participants listen to lectures, participate in group discussions and use teaching aids to increase their interpretative skills. Skill with interpretation of HIV-status according to national testing algorithm is integral to the quality system for "same-visit" HIV testing. In addition, trainees must learn to interpret results from testing quality-control (QC) materials.

During hands-on training, workshop participants were encouraged "to think like lab people". During this training, the ratio of trainees to facilitators was 4:1. Facilitators observed and interacted individually with trainees as they learned the details of testing. Testers must acquire skills that are routine for laboratory staff. During the practical sessions, workshop participants learned to use transfer pipettes, to perform finger-stick blood collection, to use rapid-test devices, to keep accurate records, to conduct routine testing of QC materials and to use the Ministry national algorithm to provide HIV infection status reports.

To integrate quality into "same-visit" HIV testing, each component of training was customized to the Ministry national testing algorithm. The training included use of standard operating procedures (SOPs) for each step of the testing process. All hands-on practical time was SOP-driven. Each workshop participant was given a Reference Manual (Additional file 3) containing all SOPs.

Training workshops included both written and practical examinations. To successfully complete a training workshop, participants were required to score 80% on a written exam and 100% on a practical exam. Sample test questions from the written exams can be found in Additional file 8.

After successfully completing a training workshop, certification-candidates enrolled in a laboratory internship. During the internship, each candidate tested 50 known samples under supervision of a certified tester. A member of the clinical laboratory staff at Port of Spain General Hospital served as supervisor for laboratory interns.

After successfully completing an internship, certification-candidates were required to complete a proficiency exam. The proficiency exam involved accurately testing a panel of unknowns. The unknowns were purchased from an external supplier. Additional information about the panels used for Ministry certification can be found in Additional file 1.

### Samples for Testing

Finger-stick blood draw is used for sample collection for "same-visit" HIV testing in TT. This method provides suitable samples for client testing and significantly reduces biological risk hazards at testing sites.

### Quality Assurance

The Ministry protocol specifies daily testing of quality control (QC) materials by each person who provides testing. Each tester must test both known positive and negative QC samples with each of the tests in the Ministry algorithm on each day of testing. This means that daily QC testing requires 6 tests per tester per site and the total daily cost of testing must include costs of these supplies.

Regular testing of QC materials is the most important feature of the quality program described in the Ministry protocol. Results from testing of QC materials must be available each day before testing can begin. If there are problems with the daily QC testing, "same-visit" HIV testing must be halted until the problems are resolved. In addition, these QC test results are regularly reviewed by the Quality Monitor as part of each site audit. These QC test results are essential to trouble shooting problems that arise with decentralized testing. In short, QC sample results are integral to allowing HIV testing to be independent of laboratory confirmation.

### Pilot Site Testing

The Ministry selected a newly refurbished public health sector clinic in Port of Spain as the pilot site for "same-visit" HIV testing. Differences existed between what the TWG had discussed as suitable space for HIV testing and what was found at the pilot site. These differences led to the development of a site-readiness checklist (Additional file 5). The manager of the pilot site and others from the TWG oversaw renovations at this site. When the site fulfilled the readiness criteria, it was designated as the pilot site for "same-visit" HIV testing.

After the "same-visit" HIV testing was optimized at the public health sector pilot site, it was expanded to a second site. The second site was an NGO contracted by the Ministry to provide reproductive health services at five locations in TT. The site selected for expansion of testing had facilities that met site-certification criteria and three staff members who had completed Ministry tester-certification. CAREC recommended that this NGO location add "same-visit" HIV testing to its program services. CAREC funded and provided technical oversight to this NGO. CAREC also advocated for national policy that would include NGO provision of "same-visit" HIV testing.

## Results

### Consensus process

Protocol consensus facilitated transition to "same-visit" HIV testing in TT. The professional diversity of the TWG meant that consensus for the protocol developed slowly. By creating a diverse TWG to guide this process, the Ministry obtained a protocol for HIV testing that is independent of laboratory confirmation. This became the policy tool that made "same-visit" testing a reality.

### Policy adoption

The TWG protocol was completed in December 2005. The protocol was reviewed at CAREC and in January 2006, the Ministry ratified the protocol and adopted it as policy for HIV testing in TT. This Ministry policy facilitated additional piloting of "same-visit" HIV testing in the public-health-sector-clinic pilot site and allowed expansion to the NGO-sponsored-clinic site. During the first year after policy adoption, more than 2500 clients received "same-visit" HIV test results.

### Pilot testing

A pre-pilot stage of testing began in July 2005. The public health clinic selected as the pilot location had two rooms and three clinic staff members which met Ministry-certification criteria. During July 2005, 22 clients received test results at this site. Members of the TWG and other consultants visited the site often to monitor the initial weeks of testing. These visits provided opportunities for testers to discuss problems and find solutions. Testing expanded slowly and by the end of 2006, 2323 clients had received test results at this site; for an average of 185 clients per month. By June 2007, 83 clients at this location had been diagnosed HIV-positive and referred to an ART location. Testing at this site is provided to clients free of charge. This site is included in the Ministry medical warehouse supply system and all testing supplies at this site are obtained through this system. Clinical follow-up for the first year of clients who tested HIV-positive at this site will be presented in a subsequent publication.

In May 2006, "same-visit" HIV testing was expanded to a second site, an NGO clinic near the pilot site. Testing at this site began with three certified testers and one certified testing room. This site is not part of the Ministry public health sector and maintains an independent inventory supply chain. Because this site could not procure testing supplies through the Ministry medical warehouse system, CAREC agreed to support the initial costs of HIV test kits. This site opted to provide testing on a "cost-recovery" basis. Uptake of testing at this site has been significantly less than at the public health clinic location. During the first month 15 people were tested. By June 2007, more than 400 clients had been tested at this site. Five were diagnosed HIV-positive and referred to a nearby ART location.

### Use of third test

The Ministry protocol stipulated that the third test in the algorithm would only be used for client testing when discordant results were obtained with the first two tests. By January 2007, more than 2500 clients had obtained HIV test results. Two of those clients had discordant results in the first two tests of the algorithm. Both of these clients tested negative with the third test in the algorithm. Both clients received negative HIV status reports. To date the primary use of the third HIV rapid test is as one part of the QC material testing process.

### QC Results

As indicated previously, the approach used to monitor quality of testing in the TT program had not been used elsewhere. Limited international guidance was available to the TWG on this topic. The TWG recommended each tester should test one positive and one negative QC sample with each rapid test on each day of testing. The QC sample results compiled at the pilot site and the NGO site between July 2005 and April 2007 are shown in Table 1. By June 2007, more than 3800 individual QC samples had been tested. These represent more than 900 "tester days" of HIV testing. The samples were tested using more than 10 different lot numbers of rapid tests by 5 different testers. None of the individual QC sample tests gave invalid or incorrect results. (QC samples were not tested with Stat-Pak for several months and these data are not available.)

**Table 1:** Summary of QC sample data.

	QC positive samples tested	QC negative samples tested	Total
Pilot Site			
Determine	638	638	1276
UniGold*	635	635	1270
Stat-Pak**	348	348	696
NGO site			
Determine	95	95	190
UniGold	95	95	190
Stat-Pak	95	95	190
Total	1906	1906	**3812**

*Three sets of UniGold QC results were missing from the data log.

**QC results for Stat-Pak were not collected at the pilot site for an extended period in mid-2005.

### Cost of testing

Review of the cost of "same-visit" HIV testing in accordance with the Ministry policy is instructive. A price list of the supplies needed for testing is included (Additional file 9. All supplies used at the health-clinic pilot site and for tester certification training were obtained through the Ministry medical warehouse system. All price negotiations were internal to that system. The Ministry protocol specifies daily use of QC materials by each person who provides testing. This means that daily QC testing requires 6 tests per tester and the total daily cost of testing supplies must include costs of these supplies. During 2006, the daily cost of testing QC materials was TT$106.06 (or US$17.67). Test supplies for the two tests for client testing cost TT$31.79 (or US$5.30).

The daily cost of testing ***per client ***varies according to the number of clients tested. The number of clients tested by each tester during a single day can vary from 1 to 10. When only one client is tested during a day, the total cost of supplies must include both the QC supply testing cost (TT$106.06) and the client supply testing cost (TT$31.79) for a total of TT$137.79 (or US$22.96). When 10 clients are tested in a day, the total cost of supplies (TT$106.06 for QC supplies and TT$317.90 for client supplies) is TT$423.96 (US$70.66). This cost can be averaged over 10 clients reducing the cost per client to TT$42.40 (US$6.73). (One US$ has a value of approximately six TT$.)

A final aspect of testing cost is the cost of training supplies needed to train testers. Under the Ministry protocol, testers seeking full-certification to provide "same-visit" HIV testing are required to test 50 samples under supervision of a certified tester. In TT, these supplies were also obtained through the Ministry warehouse system. During 2006, the price of training supplies for each candidate cost was TT$6,353.00 (or US$1,065.00).

## Conclusions

### Policy process

The process of developing a national policy for "same-visit" HIV in TT was challenging. In September 2004, when the Ministry requested technical assistance from CAREC, several other Caribbean nations were also interested in introducing similar testing. At that time, only one Caribbean country, Guyana, had Ministry of Health policy for HIV testing based on rapid test technology.

Several different programs within the Ministry in TT were interested in implementing "same-visit" HIV testing. The Ministry had the wisdom to designate a TWG that represented these diverse program interests. This TGW diversity was an enormous challenge at the outset of protocol development. Forging diversity into an evidence-based, consensus protocol took many months. The Ministry policy satisfies multiple interests and concerns within the health sector of the country.

### Quality Monitoring

The challenges inherent in quality monitoring of "same-visit" HIV testing should not be under estimated. Programs that involve HIV testing by non-laboratory staff in non-laboratory settings provide unique challenges to quality monitoring. Because rapid test devices are single use devices, the traditional approaches used to monitor quality of laboratory-based testing are not directly applicable.

Guidelines for quality monitoring of HIV testing have been prepared [10]. These guidelines have been used in high-volume settings where veni-puncture blood is used for testing. These guidelines are less useful in low-volume settings where finger-stick blood is tested. The quality monitoring approach described in these guidelines is known as re-sampling. The re-sampling procedure uses dried-blood-spots (DBS). This quality-monitoring approach, DBS re-sampling, is incompatible with finger-stick blood collection. Dried blood spots require approximately 100 microliters of blood per spot. Re-sampling procedures usually specify 3 spots for testing. Thus DBS resampling requires 300 microliters of blood. Blood volumes collected by finger-stick collection vary significantly, but on average 200 microliters or less can be collected from one finger-stick procedure.

### Finger-stick blood collection

The TWG recommended use of finger-stick blood collection for HIV testing in TT. This method of blood collection provides suitable samples for client testing and streamlines the testing process. This choice had several advantages. The two or three drops of blood obtained by finger-stick collection are sufficient to complete testing according to the Ministry protocol. Personnel and supplies for phlebotomy are not needed. No "extra" blood is collected, and thus there are no risks of accidental spills, sample mix-up or mislabeling.

An important corollary of finger-stick blood collection is that no samples are available for laboratory-based confirmation. The approach to quality monitoring must be designed accordingly. Over a period of several months the TWG developed consensus for quality monitoring with the following words: "At the beginning of each day of HIV rapid testing, each staff member who will provide test results must test two externally-provided QC samples (one HIV-positive sample and one HIV-negative sample) using each type of HIV rapid test." (Encouragement to pursue this approach was provided by David Barnett in November 2004; CW personal communication).

The TWG recognized that this approach to quality monitoring would increase the cost of testing. The cost of this process per test would be averaged over the total number of clients tested each day. This number was predicted to vary between one and ten. In their consensus decision, the TWG agreed that concerns about maintaining quality testing took precedence over concerns about the cost of testing. Their protocol decision meant that results from 6 rapid tests would be part of the quality system for each tester on each day of testing. The results are kept in the QC sample log which is one of the most important records of the quality monitoring system (see Additional file 1).

### Limitations

One limitation for "same-visit" HIV testing in TT is associated with maintenance of QC sample log records. QC sample log records can be maintained either on paper or electronically. Electronic records are much easier to review. The review of paper records is tedious. Review of these records is one of the "rate-limiting" steps in further expansion of "same-visit" HIV testing in TT.

A second limitation of this approach is that it is labor intensive. Although this approach uses non-laboratory personnel to provide testing, the people who provide testing must be trained. When personnel turn-over is high, training expenses can become a significant component of overall costs.

### Summary

In 2006, Trinidad and Tobago adopted National Policy for "same-visit" HIV testing. This policy incorporated international best practices into a program of Ministry-certified HIV testing. In this program, one person serves as both counselor and tester to individuals who seek confidential HIV status reports. Finger-stick blood samples are tested in a parallel algorithm and clients receive test results within 30 minutes. The quality monitoring aspects of this testing program can be used in non-laboratory settings and are suitable to monitor testing across multiple testing sites. The program is staffed by non-laboratory personnel who are Ministry-certified to provide quality testing in decentralized (non-laboratory) settings. This approach is working well in TT and may serve as a model for other venues.

## Abbreviations

**AIDS**: Acquired Immune Deficiency Syndrome; **ART**: Anti-retroviral Therapy; **CAREC**: Caribbean Epidemiology Centre; **CDC**: United States Centers for Disease Control and Prevention; **DBS**: Dried Blood Spot; **GAP**: Global AIDS Program; **HIV**: Human Immunodeficiency Virus; **NACC**: National AIDS Coordinating Committee; **NSP**: National Strategic Plan; **NGO**: Non-Governmental Organization; **PMTCT**: Prevention of Mother-to-Child Transmission; **QC**: Quality Control; **SOP**: Standard Operating Procedure; **TT**: Trinidad & Tobago; **TWG**: Technical Working Group; **UNGASS**: United Nations General Assembly Special Session; **WHO**: World Health Organization.

## Competing interests

The authors declare that they have no competing interests.

## Authors' contributions

VD, SS, and CW were members of the technical working group. VD, SS, CA and CW customized the training materials and facilitated training workshops. DM was head of the MOH pilot project. SS coordinated the NGO pilot project. CA and CCK compiled and analyzed data from the pilot sites. All authors read and approved this manuscript.

## Pre-publication history

The pre-publication history for this paper can be accessed here:

http://www.biomedcentral.com/1471-2458/10/185/prepub

## Supplementary Material

Additional file 1 **Protocol for implementation of diagnostic HIV rapid (same-visit) testing: Trinidad and Tobago**. This document is the technical framework (protocol) adopted by the Ministry for implementation of "same-visit" HIV testing. Click here for file

Additional file 2 **Classroom training materials**. This document includes the classroom-training materials used to train non-laboratory personnel to use rapid tests to provide "same-visit" HIV test results. Click here for file

Additional file 3 **Reference Manual**. This is the Reference Manual provided to each person trained to provide rapid tests to provide "same-visit" HIV test results. Click here for file

Additional file 4 **Certification Criteria**. This document lists certification criteria and supplies for both testers and venues that provide "same-visit" HIV test results. Click here for file

Additional file 5 **Check List: HIV Testing Site**. This document lists items that must be ready on-site before testing can begin. Click here for file

Additional file 6 **Tester certification training program**. This document provides an overview of the three-step training program required for tester certification. Click here for file

Additional file 7 **Hands-on training materials**. This document describes the hands-on portion of training for tester-certification. Click here for file

Additional file 8 **Sample questions**. This document lists sample test questions used to prepare exams required for tester certification. The question list was submitted to a web-based program which generated a random selection of test questions for each exam. Each exam included 25 questions. No two exams were identical. Click here for file

Additional file 9 **Cost of testing**. This document presents costs associated with "same-visit" HIV testing. Click here for file
