# Supplementary material for: Same-visit HIV testing in Trinidad and Tobago
Source: BMC Public Health. 2010 Apr 9;10:185. doi: 10.1186/1471-2458-10-185 (PMC2858728; doi:10.1186/1471-2458-10-185)
Supplement: Additional file 1 — Protocol for implementation of diagnostic HIV rapid (same-visit) testing: Trinidad and Tobago. This document is the technical framework (protocol) adopted by the Ministry for implementation of "same-visit" HIV testing. [file 1471-2458-10-185-S1.PDF]

Protocol for Implementation of Diagnostic HIV Rapid (Same-visit) Testing: Trinidad and Tobago

FINAL

December 2005

Executive Summary

Purpose. This protocol provides the framework for implementation of diagnostic HIV testing in Trinidad and Tobago (TT). This framework provides TT Ministry of Health (MOH) the tools needed to establish **same-visit** diagnostic HIV testing in TT. These tools will allow reporting of **same-visit** HIV test results. The capacity to deliver **same-visit** HIV test results is urgently needed by health care programs and partners in TT to expand access to HIV/AIDS care and treatment.

Background. MOH TT is responsible for the delivery of publicly-funded HIV care and treatment services. Those planning implementation of these services recognize the need for **same-visit** HIV testing. An **MOH-certified** HIV-testing process based on HIV rapid testing is vital to scale-up of HIV care and treatment services in TT. HIV **same-visit** testing would serve as the first step in access to MOH-funded HIV care and treatment services. In the absence of **MOH-certified** HIV-testing, optimal delivery of many HIV care and treatment services is not possible.

Protocol. This protocol was developed within MOH TT with input from various health care providers and partners, including CAREC. Those who have participated in this process have signed a statement indicating their support for it.

Algorithm. This protocol describes a process for implementation of **MOH-certified same-visit** HIV testing in TT. Upon completion of this protocol, **same-visit** HIV test results will be reported using an algorithm comprised of three HIV rapid tests: Determine® and Uni-Gold™, and Stat-Pak. These test results will be diagnostic and **MOH-certified**. A positive diagnostic test result will be the entry point for access to publicly-funded HIV care and treatment services in TT. The test result will be recognized at all MOH locations throughout the country.

Challenges. Many of the challenges to scale-up of **same-visit** HIV testing involve assurance that the quality of testing is uniform at all testing locations. HIV rapid tests are easy to use and readily available. MOH-certification of **same-visit** HIV testing will include MOH-approved training for each person who provides testing on behalf of MOH. Guidance for the certification process is included in this protocol. Each site where testing is performed will be included in an MOH-monitoring process. Guidance for site monitoring is also included in the protocol.

Timeline. This protocol continues for six (6) months from the start date.

Contents

1. Overview
    A. General Background
    B. CAREC Background
    C. Trinidad and Tobago Background
2. Current Situation
3. Recommendations
4. Project Goal
5. Objectives
6. Methods
    A. Training
    B. Pilot Site Assessment
    C. Standard Operation Overview
    D. Pilot Site Operation
    E. Additional Sites
7. Monitoring
    A. Quality Management
    B. Quality Control
8. Data
9. Confidentiality
10. Protocol Process
11. Table 1
12. References
13. Timeline

Appendices (1 -11)

PRINCIPAL IMPLEMENTOR

Ministry of Health Trinidad and Tobago

IMPLEMENTATION PARTNERS

National AIDS Coordinating Committee

National PMTCT Coordinator

Family Planning Association of Trinidad & Tobago

Caribbean Epidemiology Centre (CAREC)

Global AIDS Program Caribbean Regional Office

CDC/GAP Caribbean Regional Laboratory Advisor

SPONSORING INSTITUTIONS

Ministry of Health Trinidad and Tobago

Caribbean Epidemiology Center (CAREC)

List of Acronyms

**AIDS**	Acquired Immune Deficiency Syndrome
**APHL**	American Public Health Laboratories
**CCPRI**	Caribbean Care and Prevention Institute
**CDC**	United States Centers for Disease Control and Prevention
**CMC**	CAREC Member Country
**CMOH**	Chief Medical Officer for Health
**EIA**	Enzyme-Immunoassays
**EQA**	External Quality Assurance
**FPATT**	Family Planning Association of Trinidad and Tobago
**GAP**	Global AIDS Program
**HIV**	Human Immunodeficiency Virus
**MOH**	Ministry of Health
**NACC**	National AIDS Coordinating Committee
**NAP**	National AIDS Program
**NGO**	Non-Governmental Organization
**NWRHA**	North West Regional Health Authority of Trinidad and Tobago
**PMTCT**	Prevention of Mother-to-Child Transmission
**POSGH**	Port of Spain General Hospital
**QA**	Quality Assurance
**QC**	Quality Control
**SOP**	Standard Operating Procedure
**TT**	Trinidad & Tobago
**TPHL**	Trinidad Public Health Laboratory
**VCT**	Voluntary Counseling and Testing
**WB**	Western Blot
**WHO**	World Health Organization

1. Overview

A. General Background

Role of HIV testing. Testing plays an essential role in prevention of HIV transmission. Since the beginning of the HIV/AIDS epidemic, surveillance testing for detection of HIV infection has been widely used. Surveillance testing for HIV permits public health authorities to follow the spread of HIV in *populations.* Surveillance testing does not allow *individuals* to know their HIV status. On the contrary, those who provide samples for surveillance testing are not routinely offered access to test results for the samples they provide. As the HIV epidemic has grown, the need for *individuals* to know their HIV status has become apparent. This need has fostered development of diagnostic HIV testing. Results of diagnostic testing are provided to *individuals*. In some places, diagnostic HIV test results are also used to provide surveillance information. Diagnostic HIV test results can be stripped of information connecting them to specific individuals and used to compile surveillance data about HIV in populations. Because diagnostic HIV testing can provide HIV-infection status information for ***both*** *individuals* and *populations*, the trend in HIV testing is toward diagnostic testing [1]. An early indication of this trend came in 1999, when CDC issued guidelines recommending addition of individual AIDS-case reports to surveillance data [2]. The recommendations included information for ensuring patient confidentiality in this process.

Limitations of all testing. In the early years of the HIV epidemic, laboratory facilities were required to obtain HIV test results. Because early testing was conducted to *screen* for HIV-infection, the most useful HIV tests were those optimized for this capacity. Technically, this meant that many HIV tests were designed to have 100% sensitivity. One reality of test design is that no test can be both 100% sensitive and 100% specific. Thus tests optimized for HIV *screening* sensitivity occasionally yield false positive results. These tests will detect HIV antibodies in *all* samples where HIV antibodies are present. Occasionally they will also erroneously detect something that “looks like” HIV antibody, but is not. Unfortunately almost no technical information about the biochemical and immunological aspects of false positive HIV test results is available.

Need for a testing algorithm. When HIV testing was limited to surveillance testing, occasional false positive results were not significant problems. As the need for diagnostic testing increased, the problem of false-positive HIV test results also increased. Those reporting *individual* (diagnostic) HIV test results must be confident that they are not reporting false positive results. Much work has been devoted toward increasing confidence in the quality of diagnostic HIV testing. Those who design HIV tests have developed tests with ever increasing specificity (while retaining 100% sensitivity). Others have developed *combinations* of HIV tests which yield HIV testing suitable for diagnostic purposes. The combinations of tests used to provide diagnostic HIV testing are also referred to as HIV testing algorithms. The most important difference between surveillance testing and diagnostic testing is that diagnostic testing always requires a testing algorithm and does not rely on a single test. Algorithms describe the path by which a *single* sample can be tested

more than one time. They define both the specific tests needed and the order in which they must be used.

Algorithms for same-visit HIV testing. Much attention has been devoted to HIV testing algorithms and many alternatives are currently in use [3-10]. One of the most useful references about HIV testing algorithms has been jointly developed by WHO, CDC, and APHL [11]. Although this document was originally developed for use in Africa, the information it contains is applicable in other locations. In many countries, the Ministry of Health (MOH) is responsible for delivery of publicly-funded HIV services. Early in the delivery process, those planning the implementation realize the need for an MOH-certified HIV-testing algorithm. Diagnostic testing according to an MOH-adopted HIV-testing algorithm can also be the first step in access to MOH-funded HIV care and treatment programs. The WHO-sponsored 3 By 5 Initiative [12] defines diagnostic HIV testing as the “entry point” to HIV care and treatment, including delivery of anti-retroviral therapy (ART). The primary goal of the 3 By 5 Initiative is scale-up of delivery of ART and other HIV services. This goal assumes a comparable scale-up of diagnostic HIV testing. In the absence of an MOH-certified HIV-testing algorithm, optimal delivery of HIV care and treatment services will be undermined.

Limitations of HIV testing. As noted earlier, technical limitations prevent design of HIV tests which are both 100% sensitive and 100% specific. This technical limitation of test design means that no HIV testing algorithm is perfect. Since the early days of diagnostic HIV testing, ambiguous test results have been documented. Occasional samples provide test results that confound those most skilled in interpretation of the results. As the pressures on diagnostic HIV testing increased, the disagreements over interpretation of HIV test results also increased. In 1989, CDC published recommendations for interpretation of HIV serology results [13]. This publication provides guidance for interpretation of conventional laboratory-based diagnostic HIV testing.

Changes in HIV testing. Since 1989, the scale of the HIV epidemic has changed drastically and comparable changes have occurred in HIV testing [14]. In 1989, conventional HIV testing was limited to two types of tests. One test type was designed to detect HIV-antibody: the enzyme immuno-assay (EIA). The other test type was designed for indirect detection of antigen produced during the HIV-infectious process: the Western Blot (WB). Both of these tests require laboratory facilities and well-trained personnel. Both tests require several hours to complete. Both tests are still in use. The revolution in HIV testing which has occurred since 1989 is the advent of HIV rapid tests. HIV rapid tests are instrument-free assays designed to detect both HIV antibody and HIV-associated antigen. HIV rapid tests have sensitivities and specificities comparable to (or exceeding) those of conventional EIA and WB tests [5-6, 8, 15 and 16]. HIV rapid tests can be used by non-laboratory personnel in non-laboratory settings. HIV rapid test results can be available in less than 20 minutes. Combinations (algorithms) of rapid tests can provide diagnostic HIV rapid testing.

Transition to HIV rapid testing. In countries where MOH-certified diagnostic HIV-testing algorithms are in place, transition to algorithms for diagnostic HIV *rapid* testing is simplified. Data from individual rapid tests can be evaluated against the national HIV testing algorithm. Combinations of rapid tests which provide comparable (or improved) diagnosis of HIV can be selected from such evaluations. Guidance for this type of evaluation is presented in the Guidelines [11] cited earlier. In the early years of diagnostic HIV rapid testing, no source of reliable comparative information about HIV rapid test sensitivities and specificities was available. Thus in many cases, large-scale evaluations of rapid-test devices were needed to support evidence-based selections of diagnostic HIV rapid testing algorithms.

Evidence for HIV algorithm selection. In the late 1990s, WHO began compiling comparative information on HIV rapid tests [15 -16]. This information has been collected from separate, unrelated evaluations of HIV rapid-test devices conducted in many locations over several years. This information provides an excellent source of information for those who must make evidence-based decisions about HIV rapid testing. Informed use of these data greatly simplifies the process of selecting individual HIV rapid tests for diagnostic HIV rapid testing algorithms. The existence of this composite database means that no one location must evaluate all possible rapid test options.

Characteristics of diagnostic HIV rapid testing. WHO recommends use of diagnostic HIV rapid testing in the context of the 3 By 5 Initiative [17]. Within The Initiative, diagnostic HIV testing is defined as the “entry-point” for access to all other services. Introduction of diagnostic HIV rapid testing significantly impacts delivery of HIV services [18]. Diagnostic HIV rapid testing can be decentralized. Diagnostic HIV rapid testing is suitable for use in non-laboratory settings. Diagnostic HIV rapid testing is especially well-suited for use in low volume settings [3, 5, 6-7]. This testing is an especially attractive option for Voluntary Counseling and Testing (VCT) services [19-20] and Prevention of Mother to Child Transmission (PMTCT) programs [21-22].

Impact of same-visit HIV test results. Implementation of diagnostic HIV rapid testing in decentralized settings permits those seeking knowledge of their HIV-status to obtain this information in a single visit to a VCT or PMTCT site. Access to “same-visit” HIV test results has greatly increased the number of people who know their HIV-status [23-24]. Diagnostic HIV rapid testing will be the doorway to all publicly-funded HIV/AIDS care and treatment services in TT.

B. CAREC Background

In the late 1990s CAREC began receiving requests to evaluate HIV rapid tests. In 1999, one such request came from the PMTCT program of Trinidad and Tobago (TT). Those responsible for the PMTCT program wanted to introduce diagnostic HIV rapid testing to provide same-visit HIV test results in PMTCT clinics. The PMTCT program requested CAREC assistance with identification of HIV rapid tests to define an HIV

rapid testing algorithm to provide these results. The original request was for evaluation data from five rapid tests. Unfortunately many problems and delays arose in this process. Two tests were withdrawn from the market before the evaluation began. CAREC evaluated three HIV rapid tests: Determine® (Abbott Labs), Insti HIV-1/HIV-2 (Bartels), and Uni-Gold™ (Trinity Biotech) on patient sera [25]. The results obtained using these rapid tests were compared with results obtained for the same samples using conventional diagnostic HIV testing available in TT at that time (EIA and WB). One of the rapid tests evaluated in this process (Insti HIV-1/HIV-2) has since been withdrawn from the market. Thus, this evaluation did not provide sufficient evidence to recommend design of diagnostic HIV rapid testing algorithms for TT.

The market for HIV rapid tests is dynamic. Many new tests are developed and marketed each year. More than 60 such tests are now available. Prices, sources and quality of HIV rapid tests vary enormously. No single organization has the capacity to provide needed comparative data. To improve this situation, WHO has provided comparative evaluation data for HIV rapid-test devices. Manufacturers seeking evaluation of newly-marketed HIV rapid-test devices may request WHO evaluation of them. Thus far WHO has released two reports [15-16] with side-by-side comparative data for 17 different HIV rapid-test devices. These WHO data are highly recommended to those seeking to make evidence-based selection of HIV rapid tests for national diagnostic HIV rapid test algorithms.

MOH leadership is vital to the process of adopting a national diagnostic HIV testing algorithm. The only difference between “rapid test” algorithms and “standard test” algorithms is the type of test used. Any national HIV testing algorithm should be supported by reliable data describing the characteristics of the tests selected for inclusion in the algorithm. As mentioned previously, WHO has simplified the national process of test selection by compiling data on many HIV rapid-test devices [15-16]. These data are especially useful for selecting individual rapid tests for inclusion in diagnostic HIV rapid test algorithms. A recent WHO report provides a full explanation of this process [3]. In countries with HIV prevalence of less than 10%, WHO recommends adoption of parallel testing algorithms [3]. The first step of a parallel algorithm is testing of finger-stick blood draws with *two* rapid tests in parallel. If the results of these two tests are concordant, the result is final and can be reported to the client. If the results of the first two tests are discordant, a third test must be used to resolve the discordance. The result of the third test is the result of the diagnosis. If the result of the third test is negative, the diagnosis is reported to the client as negative. If the result of the third test is positive, the diagnosis is reported as positive.

For several years, CAREC Member Countries (CMCs) have looked to CAREC for regional recommendations about diagnostic HIV rapid testing. These recommendations have been a long-standing challenge at CAREC. Use of HIV rapid testing for HIV diagnosis was discussed during both the 4th Caribbean National Epidemiologists and Laboratory Directors Meeting (July 2002) and a CDC/GAP Laboratory assessment team visit (Aug 2002) to CAREC in Trinidad. Consensus among key participants (CAREC,

National Laboratory Directors, and CDC) at those meetings was that regional recommendations about diagnostic HIV rapid/simple testing required more evaluation. Much has happened since that consensus.

C. Trinidad and Tobago Background

Since 2000, the PMTCT program of TT has collected data about performance of both Determine® and Uni-Gold™ HIV rapid tests. Clients who request PMTCT-based HIV testing are tested using these two tests ***and*** using conventional diagnostic HIV testing available in TT. Only clients who test negative with both HIV rapid tests receive their final test results. Clients who test positive with either rapid test are told that their test result requires additional laboratory-based testing. Recently, all results from samples tested by both testing routes during 2002 and 2003 were compared. As expected, occasional false positive results were recorded. Seven (7) of 880 HIV rapid tests (0.8%) yielded discordant results. (This means that one of the two rapid test results was positive and the other rapid test result was negative.) All discordant samples tested negative with conventional diagnostic HIV testing available in TT. As explained previously, this low-frequency of false-positive results is inherent in HIV test design.

Agreement on a national HIV *rapid* testing algorithm would significantly strengthen delivery of all MOH-supported HIV services in TT. In 2002, a report describing the HIV testing situation in TT was submitted to MOH. That report emphasized the need for selection of a third test to resolve initially discordant results in HIV testing. Selection of a third test would permit implementation of a national diagnostic HIV rapid testing algorithm. Lack of agreement about an algorithm for diagnostic HIV testing has confounded attempts to scale-up HIV testing in TT. Recently consensus was achieved on evaluation of a third-test for inclusion in the TT algorithm. This consensus was achieved by amending the protocol used for PMTCT testing in TT.

The amended PMTCT protocol requires all PMTCT samples to be tested with Stat-Pak. Until August 2005, PMTCT clients in TT were tested with two HIV rapid tests: Determine® and Uni-Gold™. Beginning in August 2005, a third HIV rapid-test device, Stat-Pak, was added as one of the HIV tests for each PMTCT sample. More than one thousand samples were tested using all three rapid tests: Determine® and Uni-Gold™ and Stat-Pak. Each of these samples was also tested using laboratory-based testing. This change in the PMTCT testing process provided comparative data for evaluation of Stat-Pak performance in TT. As in the previous process, clients who tested negative with each HIV rapid-test device received their final test results. Clients who tested positive with *any* of the three tests were told that their test result required additional laboratory-based testing.

2. Current Situation

In 2003 WHO/PAHO announced the 3 By 5 Initiative with a goal of anti-retroviral therapy (ART) for 3 million HIV-infected persons by the end of 2005 [11]. An important

aspect of the 3 By 5 Initiative is increased access to HIV diagnosis. Diagnosis is the entry-point for HIV care and treatment including ART. The 3 By 5 Initiative encourages all national HIV/AIDS programs to expand nationally-recognized (MOH-certified) diagnostic HIV testing. The optimal route for this expansion is through the introduction of ***diagnostic HIV rapid testing.***

Many people acknowledge the need for expansion of MOH-certified diagnostic HIV testing in TT. Efforts to identify the documentation supporting present HIV testing procedures in TT have been unsuccessful. The apparent absence of documentation for the present process has complicated efforts to implement same-visit testing. The HIV epidemic has brought consensus among several partners who now look to MOH to provide nationally recognized HIV testing in TT. This document has been prepared to provide a framework for that process. Partners who have sought MOH leadership in HIV testing in TT have been invited to review this document. Those who have signed the document support implementation of the protocol pending approval by MOH TT.

All partners agree that the present HIV testing situation in TT is unacceptable. Currently, HIV diagnosis in publicly-funded clinics (VCT, PMTCT and non-governmental organization (NGO) depends on laboratory-based testing. In antenatal clinics in TT, clients seeking HIV testing must await the results of as many as 10 tests before receiving their HIV test results. In other publicly-funded clinics, clients must await only one laboratory-based HIV test result. All partners recognize the need to standardize HIV testing. The partners supporting this document recommend that MOH-certified diagnostic HIV testing in TT be based on HIV *rapid* testing. Adoption of a national diagnostic HIV rapid testing algorithm in TT would significantly enhance implementation and delivery of HIV services.

The purpose of this document is to provide the technical framework for MOH-certified same-visit HIV testing in TT. This document focuses the agreement that exists within TT on same-visit HIV testing into a technical framework for implementation of MOH-certified diagnostic HIV rapid testing in TT. The starting place for agreement is adoption of a parallel-testing algorithm using HIV rapid tests. WHO recommends parallel-testing algorithms for HIV diagnosis in locations where HIV prevalence is less than 10%. To conduct this testing, whole blood from finger-stick blood draws is tested "in parallel" on two different HIV rapid-test devices. If the results of these two tests are concordant, the result is final and can be reported to the client. If the results of these first two tests are discordant, a third test must be used to resolve the discordance. The result of the third test is the result of the diagnosis. If the result of the third test is negative, the diagnosis is reported to the client as negative. If the result of the third test is positive, the diagnosis is reported as positive.

3. Recommendations

Those who have reviewed this document recognize the urgent need to improve the HIV testing situation in TT. After careful review of several sources of information, they offer the following recommendation: MOH TT should adopt a diagnostic HIV rapid testing using parallel testing as described by WHO [17]. **The first two tests in the parallel algorithm should be Determine® and Uni-Gold™ and the third test should be Stat-Pak.** The MOH TT algorithm is shown in Appendix 1.

The reasons for this recommendation are five-fold: 1) current testing in Guyana (GY), 2) recent test evaluations in Brazil, 3) recent evaluation of Stat-Pak in TT, 4) simplification of procurement, and 5) simplification of quality monitoring. More complete explanation of these reasons follows:

1) Current testing in Guyana. In 2003, MOH GY requested assistance from both CAREC and CDC/GAP with introduction MOH-certified diagnostic HIV rapid testing. Collaboration between CAREC and CDC/GAP in GY produced evidence needed for MOH GY to adopt a national algorithm for diagnostic HIV rapid testing. In 2004, GY became the first CMC to implement diagnostic HIV rapid testing. In July 2004, MOH GY (with assistance from CAREC and CDC/GAP) provided training for both government health care workers and NGO staff to implement diagnostic HIV rapid testing. Within weeks, MOH GY introduced same-visit HIV testing in six VCT sites in GY. By the end of 2004, VCT service was expanded to 25 locations throughout the country. Preliminary reports from this introduction are encouraging (personal communications).

The diagnostic HIV testing algorithm adopted in GY employs parallel testing as described by WHO. This means that three tests are needed. The tests used in the GY algorithm are Determine®, Uni-Gold™ and Stat-Pak. When results obtained with the first two tests, Determine® and Uni-Gold™, are concordant, the diagnosis is are reported to the client on the basis of these two tests. When the results of these two tests are discordant, the third test is needed to resolve the discordance. In the GY algorithm, the third test is Stat-Pak. If the Stat-Pak result is negative, the diagnosis is negative. If the Stat-Pak result is positive, the diagnosis is positive. Both Determine® and Uni-Gold™ have been evaluated by WHO. These tests were also evaluated at CAREC in 2001. Both of these tests have also been used extensively in the PMTCT program in TT.

Those who conducted the HIV rapid test algorithm evaluation in GY also recommended selection an additional test. OraQuick was selected as an alternative “third” test. The performance characteristics of Stat-Pak and OraQuick were very similar in the algorithm evaluation. Because Stat-Pak is approximately 10 times less expensive per test than OraQuick, it was selected as the standard third test in GY. Although the data supporting MOH GY adoption of these tests are not published, they have been made available to CAREC.

In addition to evaluating rapid tests for use in a diagnostic HIV testing algorithm, MOH GY also established certification criteria for personnel performing testing on its behalf. Criteria for certification of personnel who perform HIV rapid testing include demonstration of both written and practical proficiency in HIV rapid testing. All of these materials are available for review through CDC and CAREC. These materials could be useful to MOH TT in implementation of diagnostic HIV testing.

2) Recent HIV rapid test evaluations in Brazil. MOH Brazil has recently completed a large-scale (1100 samples) evaluation of seven different HIV rapid tests for possible inclusion in a national HIV testing algorithm [10]. The results of this evaluation have been submitted for publication and will appear later this year. Although Brazil has not yet adopted a national HIV rapid testing algorithm, Determine®, Uni-Gold™ and Stat-Pak were found to have clinical sensitivities of 100% and are leading contenders for inclusion in the Brazilian national testing algorithm.

3) Recent evaluation of Stat-Pak in TT. More than 1000 samples were comparatively tested in TT using Determine®, Uni-Gold™ and Stat-Pak. These samples were also tested using laboratory-based testing. Summaries of test performance characteristics for these three rapid tests using samples from TT are shown in Table 1. In brief, Determine® demonstrated test efficiency of 99.2%, Uni-Gold™ demonstrated test efficiency of 100.0% and Stat-Pak demonstrated test efficiency of 99.6% with local samples. These test results compare favorably to those reported from other geographic locations. The results support selection of Stat-Pak as the third test in the MOH TT rapid testing algorithm.

4) Simplification of procurement. If both TT and GY use the same algorithm for HIV rapid testing, procurement of HIV rapid test kits would be simplified for both countries. Both would likely obtain better prices on all purchases. Even if no immediate price reductions were available, in the event of inventory problems, exchange of available kits between countries might be feasible. With two CMCs using the same HIV rapid testing algorithm, CAREC would be better positioned to make regional recommendations on this subject.

5) Simplification of quality monitoring. If both TT and GY used the same algorithm for HIV rapid testing, quality monitoring of HIV rapid testing would be simplified for both countries. If either GY or TT experienced quality problems with a specific batch of supplies from any vendor, trouble shooting the source of the problem would be simplified if both countries were using the same materials. With two CMCs using the same rapid testing algorithm, CAREC would be in a better position to make regional recommendations on this subject.

Dr. Kevin DeCock, one of the people most knowledgeable about HIV testing, has said:

"Current practice around HIV testing, counseling, and consent is an obstacle to scale up of services…. **How to use testing is perhaps the most challenging question in HIV/AIDS policy today**." (emphasis added) [18].

Those who have contributed to this protocol have incorporated many of the lessons learned elsewhere in the global HIV/AIDS epidemic [20]. The contributors acknowledge the limitations of this protocol and have attempted to balance these limitations with the urgent need to scale up HIV testing in TT.

The contributors anticipate that MOH TT will provide the leadership needed to move TT through this challenging policy process. They recognize that CAREC is currently preparing recommendations for diagnostic HIV rapid testing. When those recommendations are complete, review of the MOH-adopted HIV testing in TT may be appropriate. In the interim, they recommend proceeding with the framework outlined in this document.

4. Project Goal

The goal of this project is scale up of diagnostic HIV testing in TT through establishment of MOH-certified "same-visit" HIV testing. Same-visit HIV test results will be obtained using diagnostic HIV rapid testing. The results will be reported in accordance with MOH-certified procedures based on a national diagnostic HIV rapid testing algorithm. The process will permit decentralization of HIV testing in TT within a context of quality monitoring by MOH TT and CAREC.

5. Objectives

The objectives of this proposal are to:

1. Review evidence for MOH diagnostic HIV testing algorithm.
2. Provide a framework for implementation of "same-visit" HIV testing in TT.
3. Select and assess a site to pilot "same-visit" HIV testing.
4. Train counselors to conduct diagnostic HIV rapid testing.
5. Provide MOH-certification of counselors for diagnostic HIV rapid testing.
6. Provide MOH-certification of facilities for diagnostic HIV rapid testing.
7. Pilot diagnostic HIV rapid testing at one site.
8. Monitor the pilot program for implementation success.
9. Use problems arising at pilot site to improve testing.
10. Expand testing to additional sites.
11. Provide a plan for on-going monitoring of diagnostic HIV rapid testing.

6. Methods

Methods needed to accomplish these objectives include training, assessment, standard operation, piloting, and expansion. These methods are elaborated below.

A. Training

In January 2005, CDC introduced materials entitled “HIV Rapid Test Training Package” [26]. This training package was designed to permit customized use in many different locations. The materials must be customized for use with a particular HIV rapid testing algorithm. The algorithm used in TT will be a parallel algorithm comprised of Determine®, Uni-Gold™ with Stat-Pak. Training will be composed of two parts: (1) training for trainers and (2) training for testers. The initial training for testers will be provided by invited facilitators who are experienced in HIV rapid test training. Upon successful completion of training, candidates will be eligible to complete MOH TT-certification for HIV testing. The certification will be good for one year.

The training will be conducted at CAREC and will last 3 days. The training will cover several topics: 1) overview of HIV infection 2) testing algorithms, 3) finger-stick blood draw procedures [27], 4) standard operating procedures (SOPs) for algorithm tests (Determine®, Uni-Gold™, and Stat-Pak), 5) result reporting procedures, 6) quality control and monitoring of HIV testing, 7) confidentiality and 8) bio-safety information including observance of universal precautions.

At the end of the training program, participants must satisfactorily complete both written and practical exams. Each successful participant must make a score of at least 80% on the written exam and 100% on the practical exam. The practical exam will require accurate identification of unknown samples. Each participant must test each unknown sample with each HIV rapid test in the algorithm. Participant records of proficiency will be essential to MOH-certification of testing. Overall performance of participants will be observed and recorded by the training team. Those who complete this training will be considered candidates for MOH TT-certification as HIV testers.

B. Pilot Site Assessment

George Street Clinic (GSC), staffed by Dr. David Musa, is the MOH-designated pilot site for “same-visit” HIV testing in TT. The pilot site, the clinic will be assessed for readiness as pilot site by a team from MOH, CAREC and CDC/GAP. **The top priority of the assessment team will be guarantees of patient confidentiality during testing.** The floor plan, lighting and sound qualities of the facility of the proposed testing sites will be reviewed. Another important priority for the assessment team will be the quality management activities in use at the site. Minimal quality management activities must be in place before piloting of same-visit diagnostic HIV rapid testing can begin. These minimal quality management activities include: 1) daily records of temperatures for the testing area, the kit storage area and the refrigerator used to store externally-provided

quality control (QC) materials, 2) inventory control records for supplies currently in use at the site, and 3) accuracy and security of records currently kept at the facility. A patient monitoring database will be set up at the pilot site. Staff will be trained in both manual and electronic data entry.

C. Standard Operation Overview

Each day a testing site is open, a staff member must update three temperature logs: 1) testing site temperature (less than 28°C), 2) HIV rapid kit storage room temperature (less than 28°C) and 3) temperature of the refrigerator where the QC samples are stored (2° - 8°C). Log sheets for these records are Appendices 2, 3 and 4 respectively.

At the beginning of each day of diagnostic HIV rapid testing, each staff member who will provide test results must test two externally-provided QC samples (one HIV positive sample and one HIV negative sample) using each type of HIV rapid test. Performance of each test should follow the manufacturer's working protocol included in each test kit package. One page summaries for these standard operating procedures (SOPs) are appended to this document: 1) Determine® (Appendix 5), 2) Uni-Gold™ (Appendix 6) and 3) Stat-Pak (Appendix 7). Devices used for QC sample testing should be labeled "QC +" or "QC –". Results of each QC sample test must be recorded in the QC sample log (Appendix 8). If a QC test result is wrong (a QC positive sample tests negative or vice versa), the test must be repeated with a second testing device. Both sets of results should be recorded in the QC log. If the second test is also wrong, no further testing can be conducted at that site until the problem is resolved. If a QC test result is invalid (no bands appear in the control window), the QC sample must be retested with a second device and the second result must also be recorded. As in the previous case, both sets of results should be recorded in the QC log. If the second test is also invalid, no further testing can be conducted at that site until the problem is resolved. No test should be reported as "weakly reactive".

After these routine QC tests have been completed, the counselor/tester is ready to receive clients for testing. Before each test, the counselor/tester must record the lot number and expiry dates for one Determine® and one Uni-Gold™ device on the HIV Rapid Test Report (Appendix 9). When this information has been recorded, the counselor/tester should open the Determine® and Uni-Gold™ rapid-test devices. After preparing the client for collection of blood by finger stick blood draw, the counselor/tester should follow the procedure for blood collection (Appendix 10). The blood should be tested according to the SOPs for the two devices (Appendices 5 and 6). During incubation of the test, the devices should be out-of-sight for both the client and the counselor/tester.

When the incubation period is complete, the counselor/tester should uncover the testing devices. If the results of the two tests are concordant, the diagnosis can reported to the client on the basis of the two tests. If the results of these two tests are discordant, a third rapid test must be used to resolve the discordance.

If a third test is required, the counselor must perform repeat the finger-stick and blood collection. This second sample should be tested using a Stat-Pak testing device. The SOP for Stat-Pak is in Appendix 7. This test should also be incubated out-of-sight of the client and the counselor. The result of the Stat-Pak test is the diagnostic result: if the Stat-Pak result is negative, the diagnosis is negative. If the Stat-Pak result is positive, the diagnosis is positive. The results of the Stat-Pak test should be added to the HIV Rapid Test Report. The reported result should also be indicated on the form. These records may be either manual or electronic.

D. Pilot Site Operation

After being found suitable for pilot site operation, GSC will begin offering “same-visit” HIV testing to interested clients according to the process outlined in this protocol. Testing will accompany HIV counseling protocols currently in use at the site.

The testing will be closely monitored by the assessment team for one month. The team will look for quality record keeping and will listen for problems reported by the counselors/testers who provide testing. Any problems identified during the pilot phase will be used to improve the testing process. During the pilot program, clients seeking HIV testing will receive same-visit HIV test results. After HIV testing at the pilot site has been optimized, the pilot phase will end and full-service MOH-certified “same-visit” HIV testing will be routinely available at GSC.

E. Additional Sites

After diagnostic HIV rapid testing has been successfully integrated into routine operation of the pilot site, testing will be expanded to a second site. The second site will follow the procedure described in **6.C** above and incorporate improvements made during pilot site operation. After one month of successful operation at a second site, diagnostic HIV rapid testing will be expanded into additional HIV testing sites in TT.

7. Monitoring

A. Quality Management

Minimal quality management activities must be in place at all testing locations. These include:

- Routine testing of externally-provided positive and negative QC samples.
- Log of results obtained each time these materials are tested (QC samples should be tested at least once each day of testing by each tester/counselor reporting HIV rapid test results.

- If a QC sample test result is recorded as invalid (no bands appear in the control window), the sample must be retested with a new device and the second result must also be recorded. If the second test is also invalid or inconclusive, no further testing can be conducted at that site until the problem is resolved.
- Daily records of temperature in the refrigerator where QC samples are stored.
- Daily records of the storage area where the rapid test kits are stored.
- Daily records of the room temperature where rapid testing is conducted.

In addition, unannounced “site audits” will be made by inspectors familiar with HIV rapid testing procedures and quality systems. These inspectors will review record keeping and testing procedures at the testing site. They will also look for evidence that confidentiality of testing is assured. Occasionally, quality inspectors will deliver verification panels [28] and expect the counselors performing rapid testing at that location to accurately identify these samples. Problems arising during completion of a verification panel will be used to review testing procedures and improve testing.

B. Quality Control

QC is integral to any quality assurance process. QC for diagnostic HIV rapid testing in TT will have two major components: 1) proper record keeping, including temperature logs for 3 areas (testing area, kit storage area and refrigerator storing externally-provided QC samples) and 2) regular testing of externally-provided QC samples. Testing of two externally-provided QC samples (one HIV-positive and one HIV-negative sample) is the most important feature of the testing site quality assurance plan. Each counselor/tester reporting HIV test results must test each QC sample using each testing device once each day that test results are reported.

The QC samples should be the first samples tested each day. These samples must be tested in accordance with SOPs for each device. The results obtained must be recorded in the QC Sample Log (Appendix 8). If test results are reported each day, QC samples must be tested each day. If externally-provided QC samples fail to test as expected with any testing device, a second device from the same vendor should be used. If the second device also fails to provide the expected result for the QC sample, testing cannot proceed for the day. Any clients tested since the most recent successful QC sample run must be notified to return for retesting. Ideally individual counselors/testers will test approximately 10 clients per day. This testing is intended for incorporation into a larger system of counseling and referral services and projected time per client is approximately 30 minutes.

8. Data Collection, Sharing and Ownership

Individual client data will belong to MOH TT. All persons with access to HIV testing data will be expected to take oaths of confidentiality. CAREC and CDC/GAP are interested in the numbers of discordant results which are recorded in this process. When the

testing record keeping is completely electronic, access to data for individual lot numbers of rapid test kits can be used to strengthen the quality monitoring system. We anticipate that MOH TT data policies will not prevent integration of this data into a larger quality system.

9. Confidentiality

All persons with access MOH TT data must sign oaths of confidentiality. Violation of this oath is grounds for dismissal.

10. Protocol Process

This protocol is includes input from several health care providers and partners who have worked with MOH TT. Their participation in the process and support of this protocol is indicated in Appendix 11.

11. Table 1 Results for Tests in TT

Summary of results for 1003 samples using HIV tests included in the MOHTT algorithm.

		Determine		Total
		+	-	
**Result**	+	104	0	**104**
	-	8	891	**899**
**Total**		**112**	**891**	**1003**

Sensitivity = 100.0% PPV = 92.9%
Specificity = 100.0% NPV= 100.0%

Test Efficiency = 100.0%

		Uni-Gold		Total
		+	-	
**Result**	+	104	0	**104**
	-	0	899	**899**
**Total**		**104**	**899**	**1003**

Sensitivity = 100.0% PPV=100.0%
Specificity = 99.1% NPV=100.0%

Test Efficiency = 99.2%

		Stat-Pak		Total
		+	-	
**Result**	+	101	3	**104**
	-	1	898	**899**
**Total**		**102**	**901**	**1003**

Sensitivity = 97.1% PPV = 99.0%
Specificity = 99.9% NPV= 99.7%

Test Efficiency = 99.6%

Sensitivity = True Positives / (True Positives + False Negatives) X 100
Specificity = True Negatives / (True Negatives + False Positives) X 100
PPV (Positive Predictive Value) = True Positives / (True Positives + False Positives) X 100
NPV (Negative Predictive Value) = True Negatives / (True Negatives + False Negatives) X 100

Test Efficiency =
(True Positives + True Negatives)
(True Positives + False Postives + True Negatives + False Negatives)

12. References

1. Fairchild AL, Colgrove J, and Bayer R. The myth of exceptionalism: the history of venereal disease reporting in the twentieth century. 2003. *Journal of Law and Medicine Ethics* 31:624-37.

2. CDC Guidelines for National Human Immunodeficiency Virus Case Surveillance, Including Monitoring for Human Immunodeficiency Virus Infection and Acquired Immunodeficiency Syndrome. 1999. *MMWR* 48(No.RR-13).

3. World Health Organization and United Nations Joint Program on HIV/AIDS Revised Recommendations for the selection and use of HIV antibody tests. 1997. *WHO Weekly Epidemiology* 72:12, March 21, 81-87.

4. Stetler HC, Granade TC, Nunez CA, Meza R, Terrell S, Amador L, and George JR. Field evaluation of rapid HIV serologic tests for screening and confirming HIV-1 infection in Honduras. 1997. *AIDS* 11:369-375.

5. Respess R, Rayfield M, and Dondero TJ. 2001. Laboratory testing and rapid HIV assays: applications for HIV surveillance in hard-to-reach populations. *AIDS Supplement* 15:(S49-59)

6. Paul SM, Grimes-Dennis J, Burr CK, and DiFerdinando GT. 2003. Rapid diagnostic testing for HIV: Clinical implications. *Supplement to New Jersey Medicine* 100:11-14.

7. Puro V, Francisi D, Siginolfi L, Civijak R, Belfiori B, Depairs P, Roda R, Modestino R, Ghielli F, and Ippolito G. 2004. Benefits of a rapid HIV test for evaluation of the source patient after occupational exposure of healthcare workers. *Journal of Hospital Infection* 57:179-182.

8. Rouet F, Ekouevi DK, Inwoley A, Chaix ML, Bugard M, Bequet L, Viho I, Leroy V, Simon F, Dabis F, and Rouzioux C. 2004. Field evaluation of a rapid human immunodeficiency virus (HIV) serial serologic testing algorithm for diagnosis and differentiation of HIV type 1 (HIV-1), HIV-2, and dual HIV-1 -- HIV-2 infections in west African pregnant women. 2004. *Journal of Clinical Microbiology* 42:4147-4153.

9. Owusu-Ofori S, Temple J, Sarkodie F, Anokwa M, Candotti D, and Allain JP. 2005. Predonation screening of blood donors with rapid tests: Implementation and efficacy of a novel approach to blood safety in resource-poor settings. *Transfusion*. 45:133-139.

10. Ferreira OC, Ferreira C, Riedel M, RegineaM, Widolin V, Crippen P, Junior AB, and Brady B. Evaluation of rapid tests for anti-HIV detection in Brazil. (accepted for publication in *AIDS*).

11. Guidelines for appropriate evaluations of HIV testing technologies in Africa (CDC/WHO/APHL) The URL for this document is: www.phppo.cdc.gov/dls/pdf/HIV%20Test%20Guidelines%20Africa.pdf.

12. Treating Three Million by 2005, Making it Happen. The WHO Strategy. 2003. ISBN 9241591129. The URL for this document is: www.who.int/3by5/en/.

13. Interpretation and use of the Western blot assay for sero-diagnosis of Human Immunodeficiency Virus type 1 infection. 1989. *MMWR*. Supplement 38:1-7. The URL for this document is: www.cdc.gov/mmwr/preview/mmwrhtml/00001431.htm

14. Jones WK. 1998. HIV and Laboratory Science. (Keynote address presented at the 1998 Conference on the Laboratory Science of HIV). The URL for this presentation is: www.phppo.cdc.gov/mlp/pdf/labhiv/WJones.pdf

15. HIV Simple/Rapid Assays: Operational Characteristics (phase 1) Report 12. 2002. Whole Blood Specimens, Blood Safety and Clinical Technology. (World Health Organization, UNAIDS).

16. HIV Simple/Rapid Assays: Operational Characteristics (phase 1) Report 14. 2004. World Health Organization, UNAIDS and the WHO Collaborating Centre for HIV/AIDS Diagnostic and Laboratory Support (Dept. of Microbiology, Institute of Tropical Medicine, Antwerp, Belgium). ISBN 9241592168.

17. Rapid HIV tests: guidelines for use in HIV testing and counseling services in resource-constrained settings. 2004. WHO ISBN 92 4 159181 1 The URL for this document is: www.who.int/hiv/pub/vct/en/rapidhivtestsen.pdf

18. DeCock KM, Marum E, and Mbori-Ngacha D. A sero-status approach to HIV/AIDS prevention and care in Africa. 2003. *Lancet* 362:184-48.

19. Downing RG, Otten RA, Marum E, et al**.** Optimizing the delivery of HIV counseling and testing services: the Uganda experience using rapid HIV antibody test algorithms**.** 1998. *Journal of Acquired Immune-Deficiency Syndrome and Human Retrovirology* 18(4): 384-388.

20. Potts M, Walsh J. Tackling India's HIV epidemic: Lessons from Africa. 2003. *British Medical Journal.* 326:1389-92.

21. Malonza IM, Richardson BA, Kreiss JK, Bwayo JJ, and John Stewart GC. The effect of rapid HIV-1 testing on uptake of perinatal HIV-1 interventions: a randomized clinical trial. 2003. *AIDS* 17:113-118.

22. Perez F, Mukotekwa T, Miller A, Orne-Gliemann J, Glenshaw M, Chitsike I, and Dabis F. 2004. Implementing a rural programme of prevention of mother-to-child transmission of HIV in Zimbabwe: first 18 months of experience. *Tropical Medicine and International Health* 9(7):774-83.

23. Gayle H, Curbing the global AIDS epidemic. 2003. *New England Journal of Medicine.* 348:1802-05**.**

24. Farmer P, Leandre F, Mukherjee JS, Claude M, Nevil P, Smith-Fawzi MC, Koenig SP, Castro A, Becerra MC, Sachs J, Attaran A, Kim JY. 2001. Community-based approaches to HIV treatment in resource-poor settings. *Lancet.* 358:404-09.

25. Evaluation of three rapid HIV assays. 2001. Caribbean Epidemiology Centre (CAREC/PAHO/WHO) Internal CAREC report, available from CAREC laboratories).

26. HIV rapid test training package: Training for quality HIV testing in an era of expanding services. 2005. Materials jointed prepared by HHS/CDC/GAP, WHO and USAID. (Package from meeting held 27-28 January 2005 in Atlanta, GA)

27. Information about Genie 1.5 mm retractable lancets is available at this URL: www.bd.com/ca/pdfs/safety/products/collection/lancets/genie_lancet_wallchart_VS5422.pdf

28. Verification panels for HIV rapid testing are available from BBI. Panel VRZ602 contains both HIV 1 and 2 antibodies. Information the panels recommended for use by MOH TT is available at this URL:

13. Timeline

Item	Description	Proposed Date	Staff
1	Prepare working draft of implementation plan for TT	5/5/05	Cynthia
2	Meet with implementation committee	5/6/05	Ingrid, Violet, Claire, Robin, Rosemary, Lynette, David, Miriam, Peggy
3	Prepare checklist for assessment	5/10/05	Robin
4	Prepared revised draft of implementation plan for TT	5/10/05	Cynthia
5	Visit George Street Clinic with Dr. Phillips	5/12/05	(entire committee)
6	Obtain support for revised plan from committee	5/12/05	(entire committee)
7	Request Dr. Hospedales review of implementation plan	5/13/05	Lynette
8	Assess George Street Clinic as pilot site	5/20/05	Claire, Robin, Cynthia
9	Meet with Dr. Hospedales to receive his response	5/27/05	Lynette, Noreen, Violet, Robin, Claire, Arlene, James, Cynthia
10	Conceive solution to Dr. Hospedales criticisms of the implementation plan	7/6/05	Violet, Noreen, Peggy, Claire, Robin, Cynthia
11	Obtain Dr. Hospedales support for the revised plan	7/21/05	Violet, Noreen, Lynette, Robin
12	Finalize MOH support for implementation plan	7/05	Violet
13	Determine format of HIV records (paper vs electronic) at pilot site	7/05	David, Harry, Noreen, Cynthia
14	Initiate invitation for Loris Hughes to assist with training during the week of 19 Sept 05	7/21/05	Cynthia
15	Order supplies	7/05	Robin, Rosemary, Violet
16	Begin collecting Stat-Pak data using amended PMTCT protocol	7/05	PMTCT testers
17	Customize TT HIV rapid test training materials	8/05	Robin, Rosemary, Peggy, Cameile, Cynthia, Loris
18	Identify HIV testing quality manager for MOH TT	8/05	Violet, Rosemary, Robin
19	Add Stat-Pak data for TT to implementation plan	9/05	Cynthia
20	Provide diagnostic HIV rapid test training in TT	9/21-23/05	Robin, Loris, Rosemary
21	Begin same-visit diagnostic testing at pilot site	9/26/05	David, Rosemary, Robin
22	Evaluate HIV rapid testing process at pilot site	10/05	Rosemary, Robin, Cynthia
23	Expand HIV rapid test result reporting to additional sites	11/05	Ingrid, Miriam, Claire, Violet, Noreen, Robin

Appendix 1.

MOH TT HIV Rapid Test Algorithm

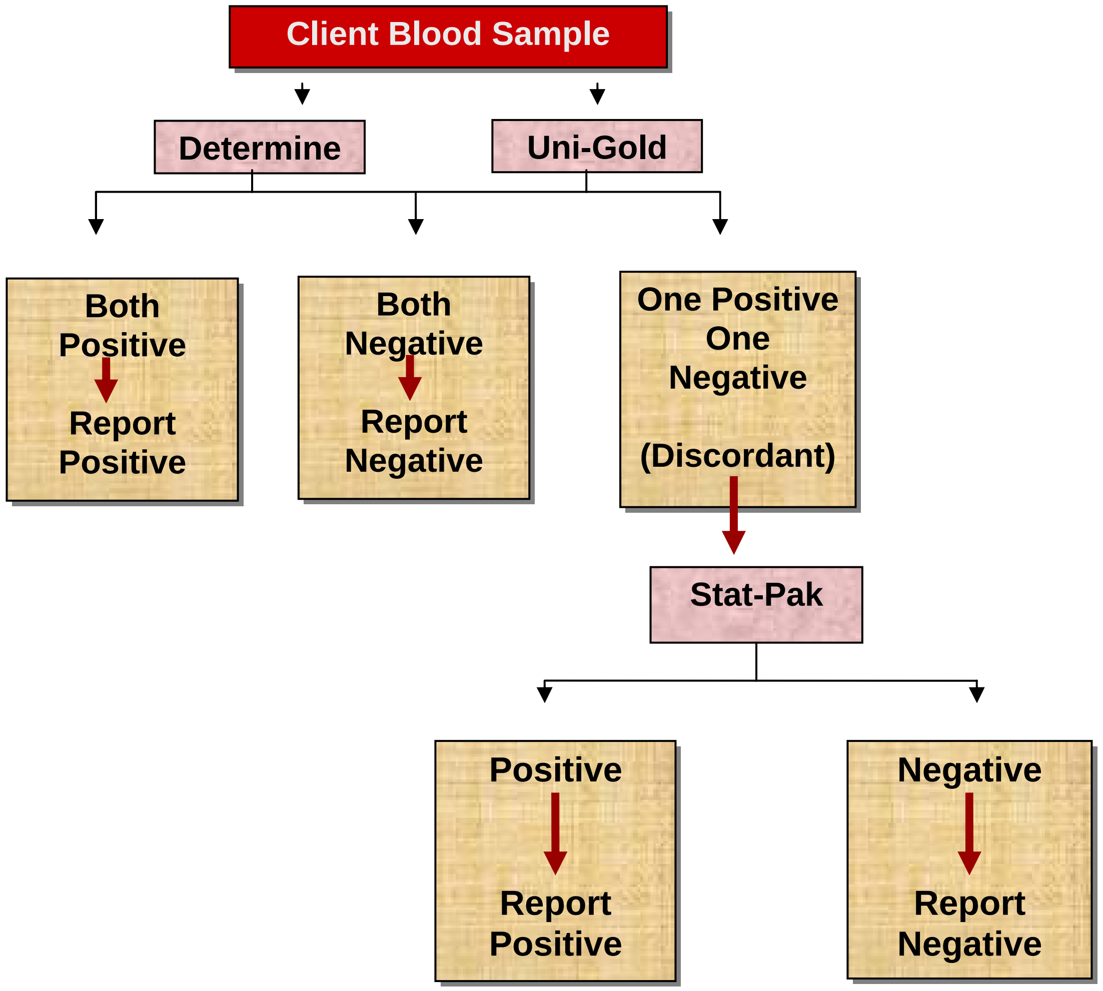

MOHTT-certified diagnosis of infection requires use of this algorithm. This algorithm permits testers to report HIV status to clients who want to "know their status" for HIV infection.

To receive their HIV status test report, each client must have two valid concordant test results.

Appendix 2.

Temperature Log: Testing Area

Record temperature each business day. Temperature in storage area should not exceed 28° C.

Date	Temperature		Date	Temperature

Appendix 3.

Temperature Log: Rapid Kit Storage Area

Record temperature each business day. Temperature in storage area should not exceed 28° C.

Date	Temperature		Date	Temperature

Appendix 4

Temperature Log: QC Sample Storage Area

Record temperature each business day. Temperature in storage area should be between 2-8° C.

**Date**	**Temperature**		**Date**	**Temperature**

Appendix 5

Determine® HIV-1/2 SOP

Summary of the manufacturer's working protocol for Determine® Rapid Tests.

Procedure

1. Check the expiration date. Do not use expired kits.
2. Remove the protective kit cover from the test device.
3. Label test device with the appropriate patient/client identification.
4. Using the Uni-Gold pipette, apply two drops of whole blood to the sample pad marked by an arrow symbol.
5. Wait one minute until blood is absorbed into the sample pad.
6. Apply 1 drop of Chase Buffer to the sample pad.
7. Wait at least 15 minutes (up to 60 minutes) and read the result.
8. Record results on the worksheet

Interpretation of Test Results (Only three results are possible with this test):

Positive (Two bands)

Red bands appear in both the control window (labeled 'C') and the patient window (labeled 'P') of the strip. Any visible red color in the patient window should be interpreted as positive.

Negative (One Band)

One red band appears in the control window of the strip (labeled 'C') and no red band appears in the patient window of the strip (labeled 'P').

Invalid (No Band)

If there is no band in the control window of the strip the test is invalid. Even if a red band is present in the patient window of the strip, the result is invalid and should be repeated.

Appendix 6

Uni-Gold™ HIV SOP

Summary of the manufacturer's working protocol for Uni-Gold™ HIV Rapid Tests.

Procedure

1. Check the expiration date. Do not use expired kits.
2. Remove the protective kit cover from test device.
3. Label test device with the appropriate patient/client identification.
4. Apply two drops of whole blood to the sample well.
5. Apply 2 drops of Wash Reagent to the sample well.
6. Read results after 10 minutes (up to 20 minutes).
7. Record results on the worksheet.

Interpretation of Test Results (Only three results are possible with this test):

Positive (Two bands)

Red bands appear in both the control area (labeled 'C') and the test area (labeled 'T') of the device. Any visible red color in the test area should be interpreted as positive.

Negative (One Band)

One red band appears in the control area of the device (labeled 'C') and no red band appears in the test area of the device (labeled 'T').

Invalid (No Band)

If there is no band in the control area of the device, the test is invalid. Even if a red band is present in the test area of the device, the result is invalid and should be repeated.

Appendix 7

HIV1/2 Stat Pak SOP

Summary of the manufacturer's working protocol for HIV1/2 Stat-Pak Rapid Tests.

Procedure

1. Check the expiration date. Do not use expired kits.
2. Remove the protective kit cover from the device.
3. Label the device with the appropriate patient/client identification.
4. Collect blood sample with the 5 µl loop provided.
5. Touch the loop to the center of the sample well and wait 3 seconds.
6. Slowly add three drops of buffer to the sample well.
7. Read results after 10 minutes (up to 20 minutes).
8. Record results on the worksheet.

Interpretation of Test Results (Only three results are possible with this test) :

Positive (Two bands)

Red bands appear in both the control area (labeled 'C') and the test area (labeled 'T') of the device. Any visible red color in the test area should be interpreted as positive.

Negative (One Band)

One red band appears in the control area of the device (labeled 'C') and no red band appears in the test area of the device (labeled 'T').

Invalid (No Band)

If there is no band in the control area of the device, the test is invalid. Even if a red band is present in the test area of the device, the result is invalid and should be repeated.

Appendix 8

QC SAMPLE LOG

Date and Time	Tester	QC samples Lot #	Determine®			Uni-Gold™			Stat-Pak		
			Lot #	Ex Date	Result	Lot #	Ex Date	Result	Lot #	Ex Date	Result
		Pos									
		Neg									
		Pos									
		Neg									
		Pos									
		Neg									
		Pos									
		Neg									
		Pos									
		Neg									
		Pos									
		Neg									
		Pos									
		Neg									
		Pos									
		Neg									
		Pos									
		Neg									
		Pos									
		Neg									

Appendix 9

HIV RAPID TEST REPORT

Client Name:________________________

Client ID:____________________________

Client DOB ___________________

Client Gender: M (   )   F (   )

Tester Name: _________________________

Testing Location: _____________________

Rapid test	**Determine®**	**Uni-Gold™**	**Stat-Pak**
Lot #			
Exp date			
Result			
Reported Result			

Appendix 10

Finger-Stick Blood Draw Protocol

1. **Wear gloves.**
2. **Open all equipment before taking client's hand.**
3. **Scrub the middle or ring finger with alcohol swab.**
4. **Blot the sampling area once with dry gauze pad and massage hand to increase blood flow.**
5. **Puncture the side of the finger with a sterile lancet. Be sure to get a good stick.**

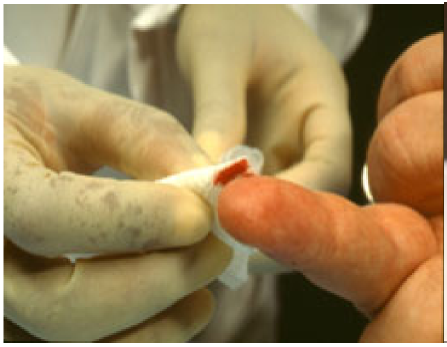

Step 7

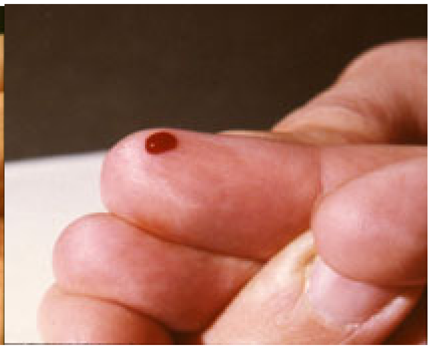

Step 8

6. **Immediately turn the client's hand over to permit blood drop forming toward the floor. (Gently massaging the finger will promote better blood flow.)**
7. **Absorb the first drop of blood with the corner of gauze pad. (Do not wipe).**
8. **Wait for new blood droplet to form.**
9. **Collect blood with pipette.**

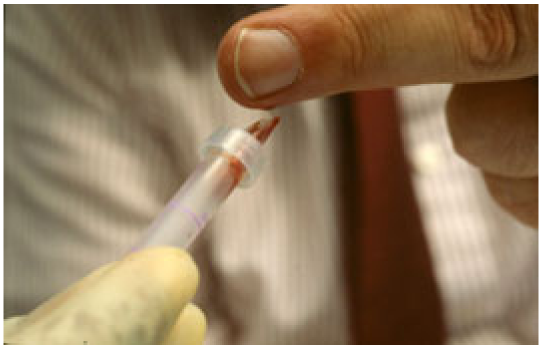

Step 9

11. **Place used lancets in sharps container.**

Appendix 11 May 2005

We, the undersigned, were invited to contribute to this **Protocol for Implementation of Diagnostic HIV Rapid (Same-Visit) Testing: Trinidad and Tobago**. Our contributions have been incorporated in ways that encourage us to endorse the protocol for MOH adoption and implementation.

Dr. Lynette Berkeley	CAREC Laboratory Manager	
Dr. Violet Duke	Liaison NACC-MOH	
Ms. Rosemary Gonzales	NWRHA Operations	
Ms. Miriam Gordon	POSGH	
Dr. Noreen Jack	CCPRI	
Ms. Peggy Mitchell	TPHL	
Dr. David Musa	George Street Clinic	
Ms. Ingrid Neckles	NAP	
Dr. Randolf Phillips	CMOH St. George West	
Ms. Sheila Sameil	CAREC-FPATT Project Manager	
Ms. Claire Sandy-Robinson	PMTCT-MOH Coordinator	
Dr. Harry Smith	CAREC-CDC/GAP	
Dr. Cynthia Warner	CAREC-CDC/GAP	
Ms. Robin Weaver	CAREC-SPSTI Lab Advisor	
