# Supplementary material for: Same-visit HIV testing in Trinidad and Tobago
Source: BMC Public Health. 2010 Apr 9;10:185. doi: 10.1186/1471-2458-10-185 (PMC2858728; doi:10.1186/1471-2458-10-185)
Supplement: Additional file 2 — Classroom training materials. This document includes the classroom-training materials used to train non-laboratory personnel to use rapid tests to provide "same-visit" HIV test results. [file 1471-2458-10-185-S2.PDF]

Participant Manual

MOH TT Certified "Same-Visit" HIV Testing Workshop

29 Nov-1 Dec 2005

TABLE OF CONTENTS

List of Participants

Module 1: Status of HIV/AIDS, Trinidad & Tobago

Module 2: Integration of HIV Rapid Testing in HIV Programs

Module 3: Overview of HIV Testing Technologies

Module 4: HIV Testing Strategies and Algorithms

Module 5: Quality of HIV Rapid Testing

Module 6: Safety at the HIV Rapid Testing site

Module 7: Preparing for Testing – Supplies and Kits

Module 8: Blood Collection – Finger Puncture

Module 9: Performing HIV Rapid Tests

Module 10: Inventory

Module 11: Refrigerators

Module 12: Quality Control

Module 13: Quality Monitoring

Module 14: (This module is not included)

Module 15: Documents and Records

Module 16: Professional Ethics

Materials Used in this Workshop

The materials used in this workshop were originally introduced at a meeting in Atlanta, Georgia on January 27-28, 2005. The meeting was entitled "*Coordinating a Systematic Roll-Out of an HIV Rapid Test Training Package: Training for Quality HIV Rapid Testing in an Era of Expanding Services".* CAREC was represented at the meeting by Ms. Wendy Kitson-Piggott, Ms. Carol Trotman and Dr. Lynette Berkeley.

The materials introduced in Atlanta resulted from efforts coordinated among WHO, PAHO, CDC and USAID. The original materials were intended for customization to "site-specific" use. Site-specific materials must suit the training needs of national HIV rapid testing programs. In September 2004, The Ministry of Health of Trinidad and Tobago (MOH TT) requested CAREC technical assistance with introduction of "same visit" HIV testing. The customized materials in this binder are part of the CAREC response to that request.

The materials in your participant manual were customized during a series of conference calls. The goal of the customization process was to create materials optimally useful to the MOH TT "same-visit" rapid testing implementation. Those involved in the customization process are listed below. This workshop is the first use of the training package in the Caribbean Region.

The original materials included 16 Modules. This participant binder contains customized version of the Modules in their original order. Module 14 is intentionally omitted.

Customization Committee

Ms. Cameile Ali (consultant)
Dr. Lynette Berkeley (CAREC)
Ms. Rosemary Gonzales (NWRHA)
Dr. Stacy Howard (CDC)
Dr. Loris Hughes (CDC)
Ms. Peggy Mitchell (TPHL)
Dr. Cynthia Warner (CDC)
Ms. Robin Weaver (CAREC)
Dr. Katy Yao (CDC)

Status of HIV/AIDS
Trinidad & Tobago

Dr Nilesh Buddha

UN Volunteer, National Surveillance Unit

Ministry of Health, Trinidad & Tobago

Challenges of tracking HIV/AIDS

- HIV diseases can be asymptomatic for years
- Many infected do not know, are not counted
- Reported data reflect testing/ care seeking behaviour & access to testing/ treatment
- Stigma, fear of loss of confidentiality and of fatal disease – create barriers
- HIV & AIDS represent same viral infection, but are reported differently – challenging to standardize reports & analysis

Challenges…(contd)

- Different sources report HIV infections, AIDS cases & deaths
- Sensitive personal data, confidential, encoding-decoding, chances of duplication
- Marginalized populations (don’t want to be identified)
- Overseas/ private sector

Limitations

- HIV positive are *only* those cases which are reported/confirmed by TPHL/CAREC
- AIDS cases are
  - picked-up by NSU Surveillance Nurses from 5 public hospitals
  - classified by Medical Epidemiologist from TPHL forms
- Surveillance data do not represent HIV Incidence and Prevalence

Regional HIV and AIDS statistics and features, end of 2004

	Adults & children living with HIV	Adults & children newly infected with HIV	Adult prevalence	Adult & child deaths due to AIDS
Sub-Saharan Africa	25.4 million [23.4 – 28.4 million]	3.1 million [2.7 – 3.8 million]	7.4 [6.9 – 8.3]	2.3 million [2.1 – 2.6 million]
North Africa & Middle East	540 000 [230 000 – 1.5 million]	92 000 [34 000 – 350 000]	0.3 [0.1 – 0.7]	28 000 [12 000 – 72 000]
South and South-East Asia	7.1 million [4.4 – 10.6 million]	890 000 [480 000 – 2.0 million]	0.6 [0.4 – 0.9]	490 000 [300 000 – 750 000]
East Asia	1.1 million [560 000 – 1.8 million]	290 000 [84 000 – 830 000]	0.1 [0.1 – 0.2]	51 000 [25 000 – 86 000]
Latin America	1.7 million [1.3 – 2.2 million]	240 000 [170 000 – 430 000]	0.6 [0.5 – 0.8]	95 000 [73 000 – 120 000]
Caribbean	440 000 [270 000 – 780 000]	53 000 [27 000 – 140 000]	2.3 [1.5 – 4.1]	36 000 [24 000 – 61 000]
Eastern Europe & Central Asia	1.4 million [920 000 – 2.1 million]	210 000 [110 000 – 480 000]	0.8 [0.5 – 1.2]	60 000 [39 000 – 87 000]
Western & Central Europe	610 000 [480 000 – 760 000]	21 000 [14 000 – 38 000]	0.3 [0.2 – 0.3]	6 500 [<8 500]
North America	1.0 million [540 000 – 1.6 million]	44 000 [16 000 – 120 000]	0.6 [0.3 – 1.0]	16 000 [8 400 – 25 000]
Oceania	35 000 [25 000 – 48 000]	5 000 [2 100 – 13 000]	0.2 [0.1 - 0.3]	700 <1 700
TOTAL	39.4 million [35.9 – 44.3 million]	4.9 million [4.3 – 6.4 million]	1.1 % [1.0 - 1.3%]	3.1 million [2.8 – 3.5 million]

HIV/AIDS in T&T

Daily in 2004	4 new cases of HIV
Total HIV (1983 – 2004)	14536
Male:Female	60:40
Total AIDS	5365
Total deaths	3273
Median age of HIV +	Male – 35, Female – 29
15 – 24 year olds	15 %
Estimated PLWHA	3.2 % adults (28,000)

HIV/AIDS in T&T

- 70 % of infections in 15-49 age group
- 45 % of new infections occur in females
- In the 15-24 age group, 70 % of the new infections occur in females

HIV/AIDS Surveillance in T & T

- Major reporting units: MRC, POSGH, EWMSC, QPCC/C, Pvt, SFGH, Tobago
- Less than 5% of total HIV/AIDS cases have data on occupation, education, condom usage, partner info, etc
- Data on ethnic origin ?

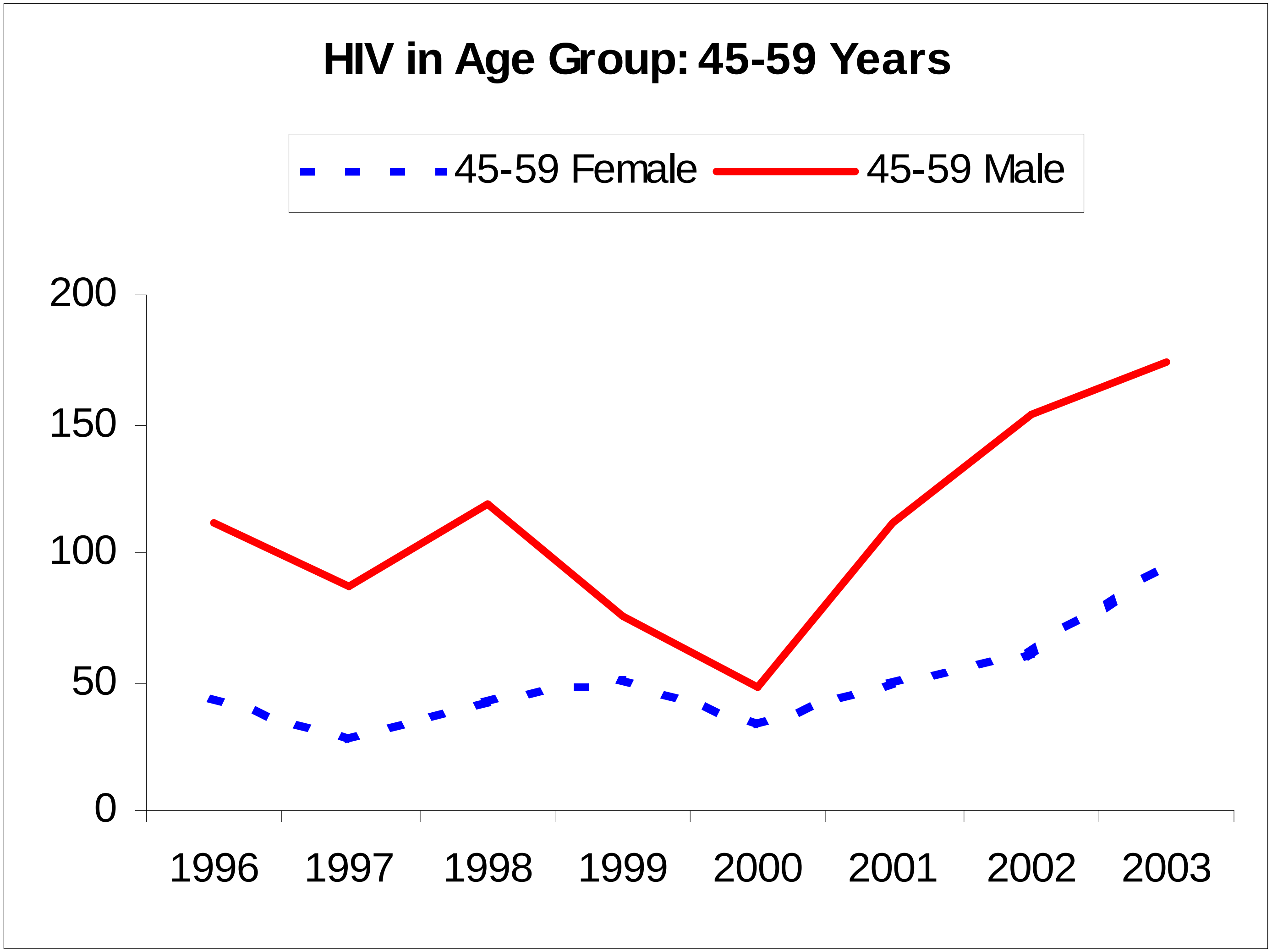
HIV in Age Group: 45-59 Years
45-59 Female
45-59 Male
200
150
100
50
0
1996
1997
1998
1999
2000
2001
2002
2003

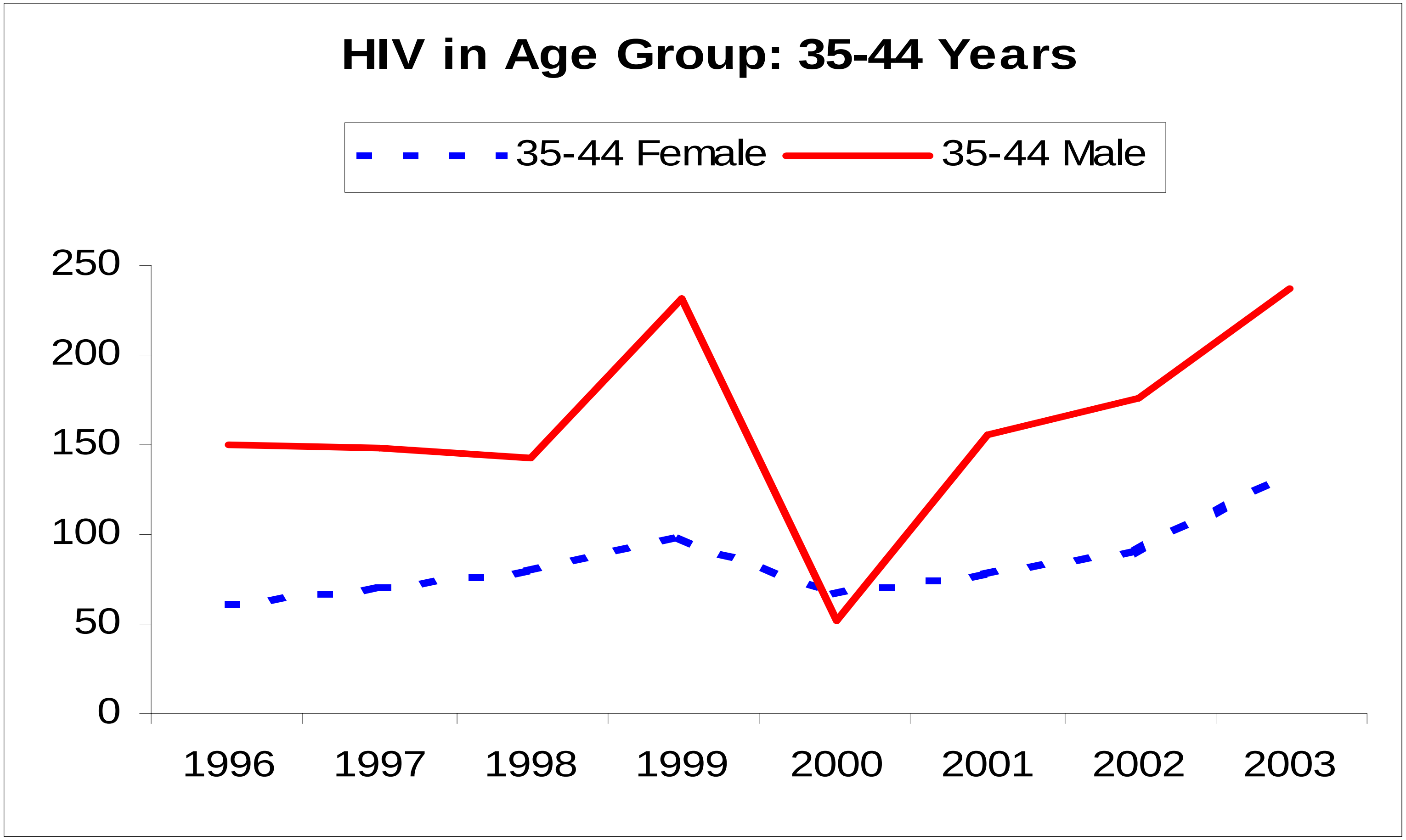

HIV in Age Group: 35-44 Years
35-44 Female
35-44 Male
250
200
150
100
50
0
1996
1997
1998
1999
2000
2001
2002
2003

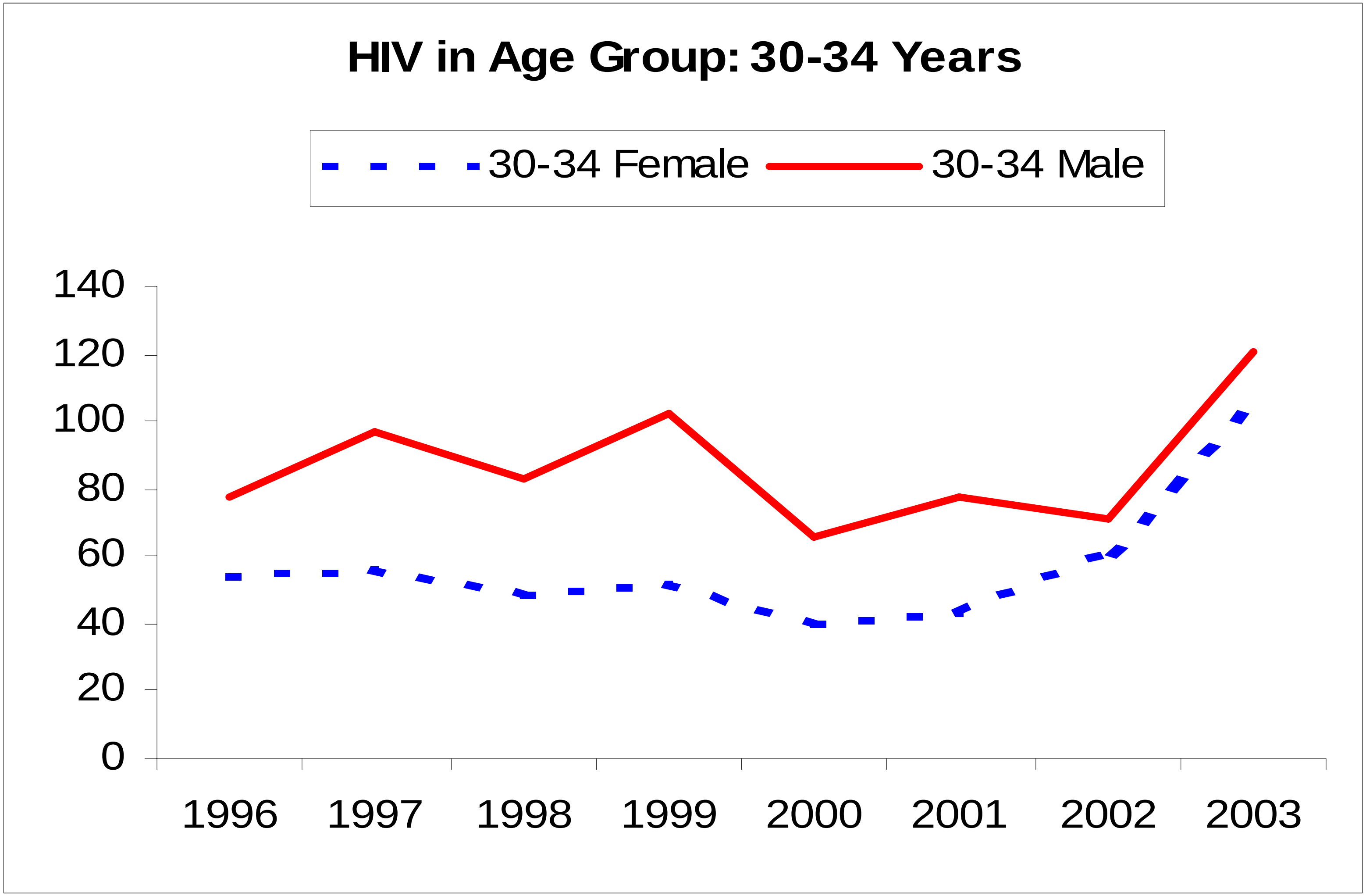
HIV in Age Group: 30-34 Years
30-34 Female
30-34 Male
140
120
100
80
60
40
20
0
1996
1997
1998
1999
2000
2001
2002
2003

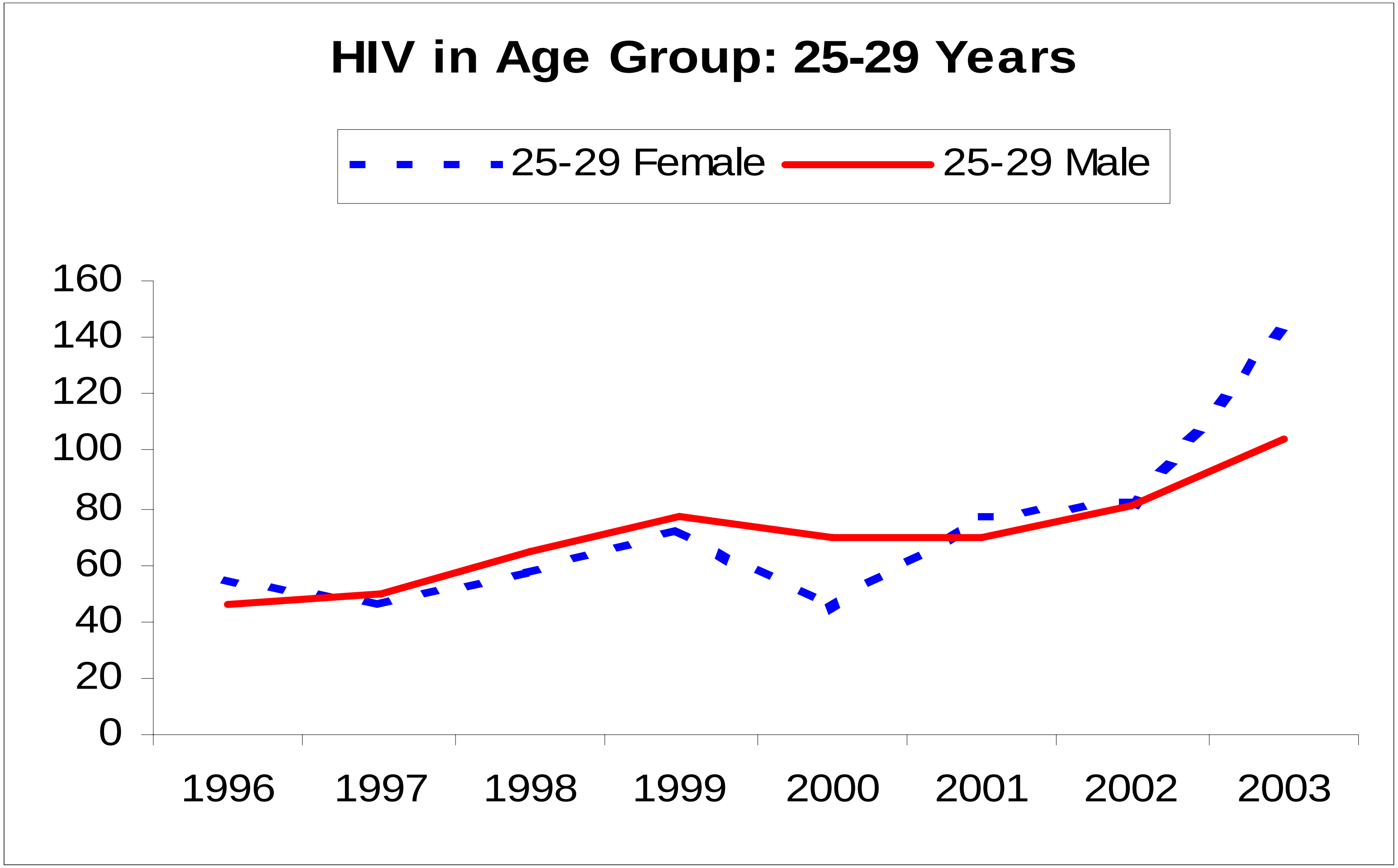
HIV in Age Group: 25-29 Years
25-29 Female
25-29 Male
160
140
120
100
80
60
40
20
0
1996
1997
1998
1999
2000
2001
2002
2003

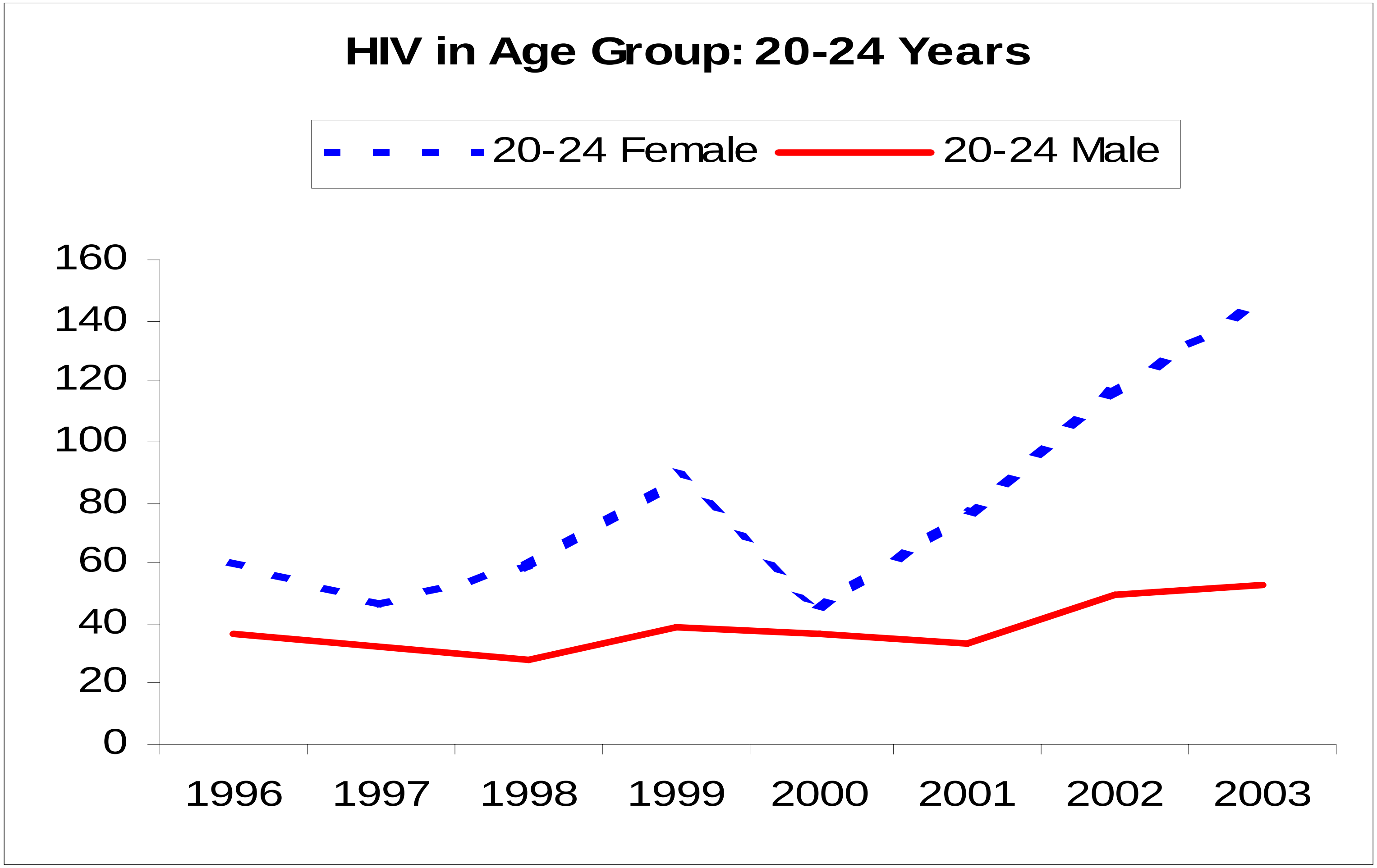
HIV in Age Group: 20-24 Years
20-24 Female
20-24 Male
0
20
40
60
80
100
120
140
160
1996
1997
1998
1999
2000
2001
2002
2003

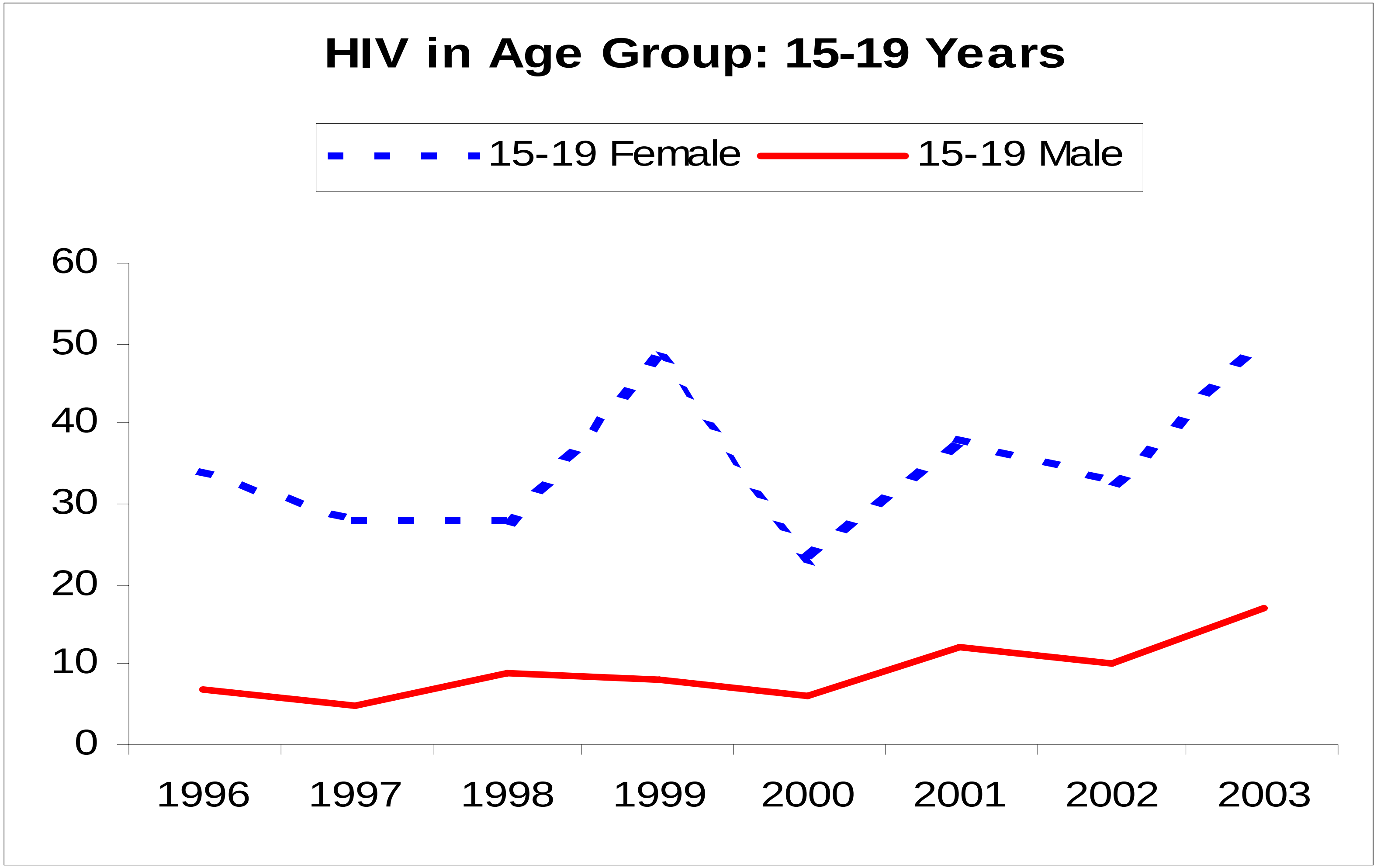

HIV in Age Group: 15-19 Years
15-19 Female
15-19 Male
60
50
40
30
20
10
0
1996
1997
1998
1999
2000
2001
2002
2003

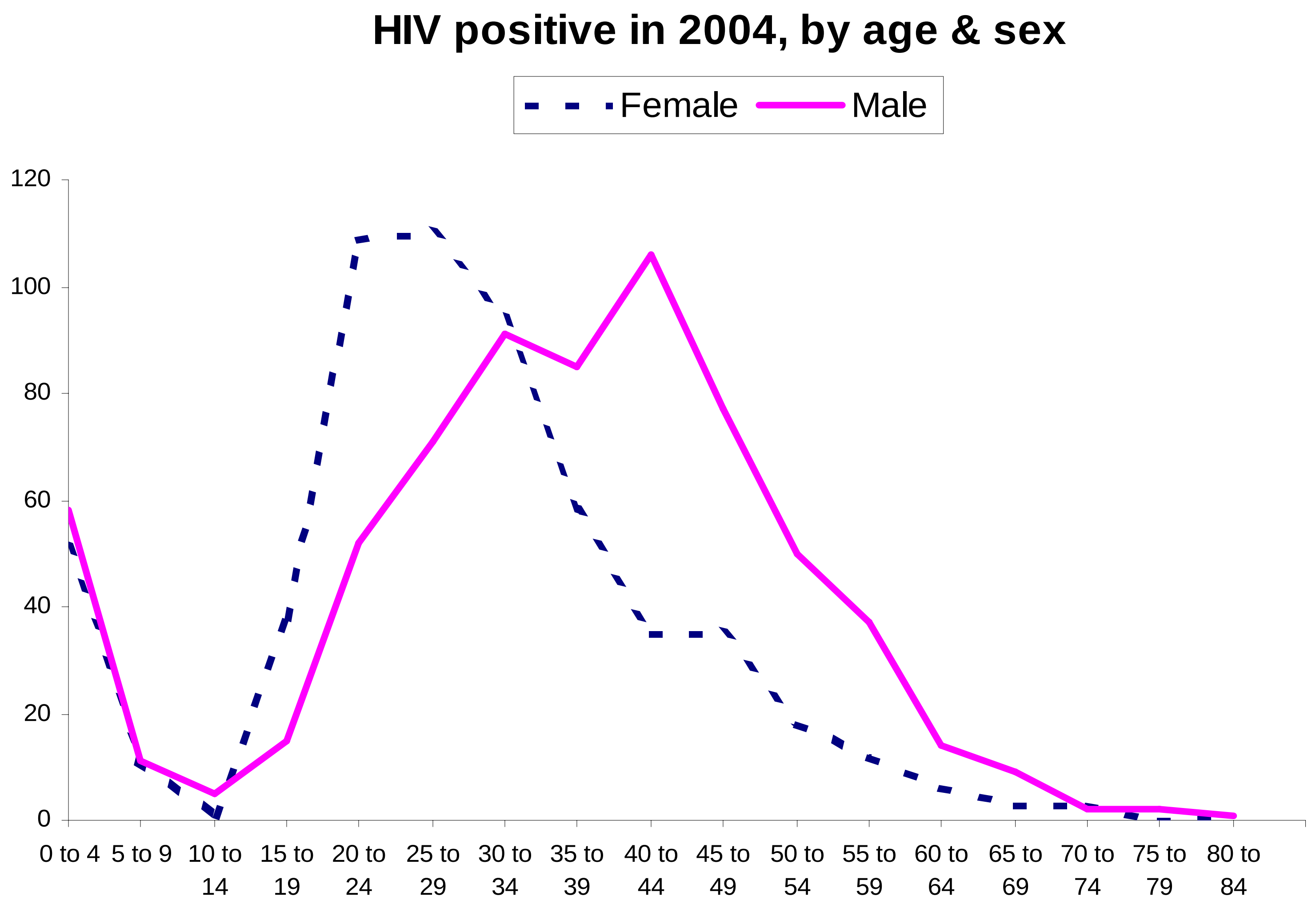

Dr Nilesh Buddha, NSU, MoH, T&T

Cumulative AIDS cases, by age & sex (1983 - 2003)

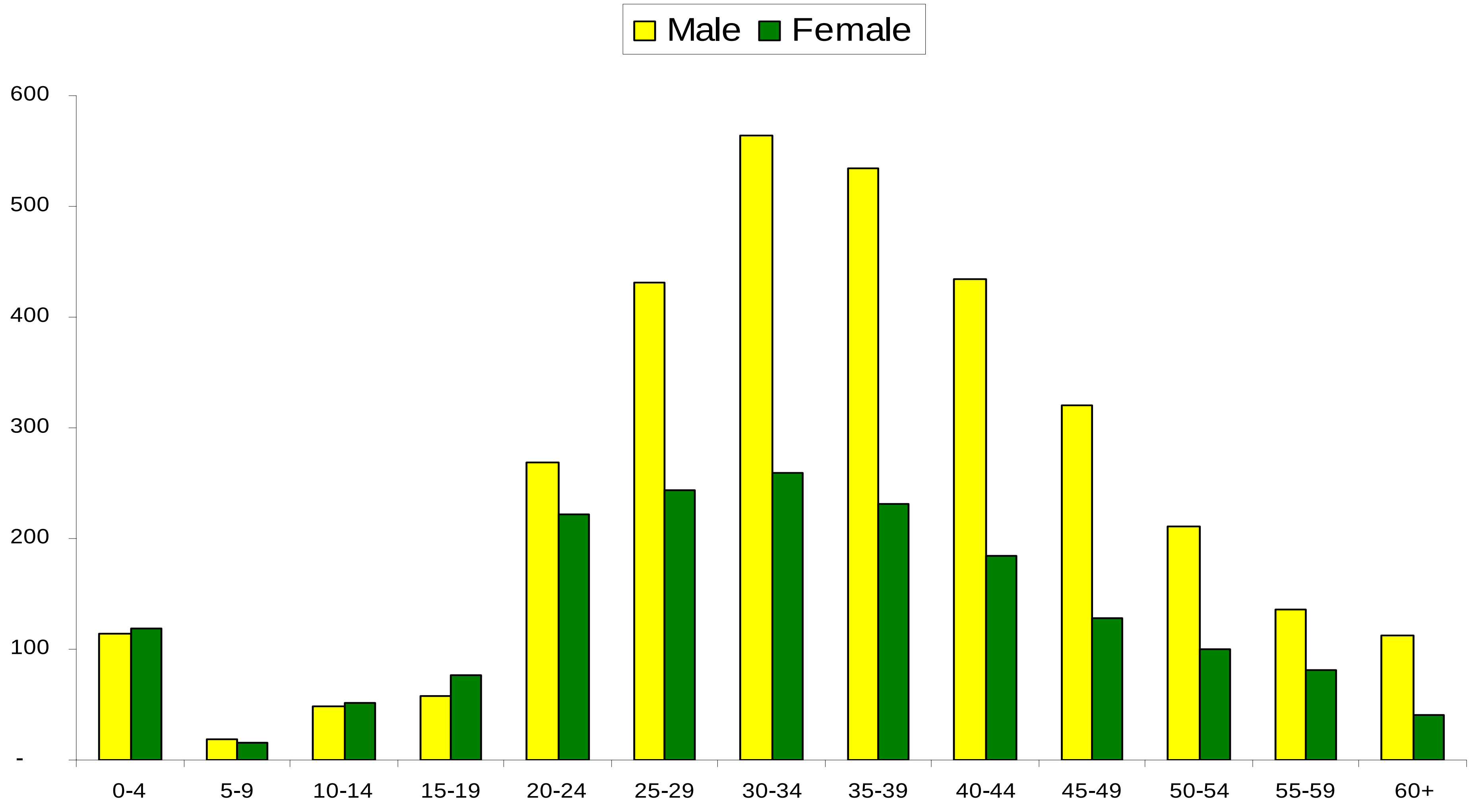

Dr Nilesh Buddha, NSU, MoH, T&T

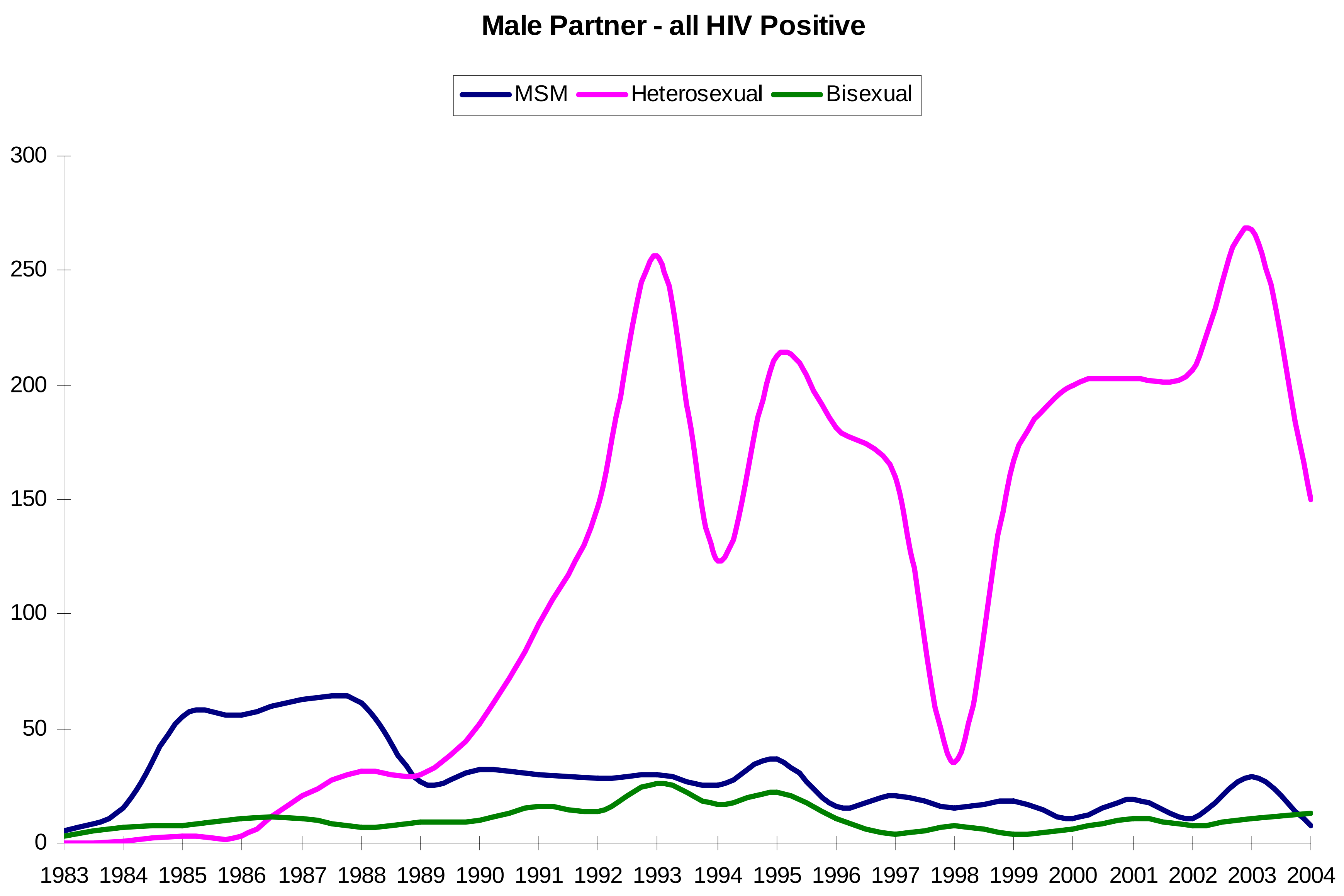

Dr Nilesh Buddha, NSU, MoH, T&T

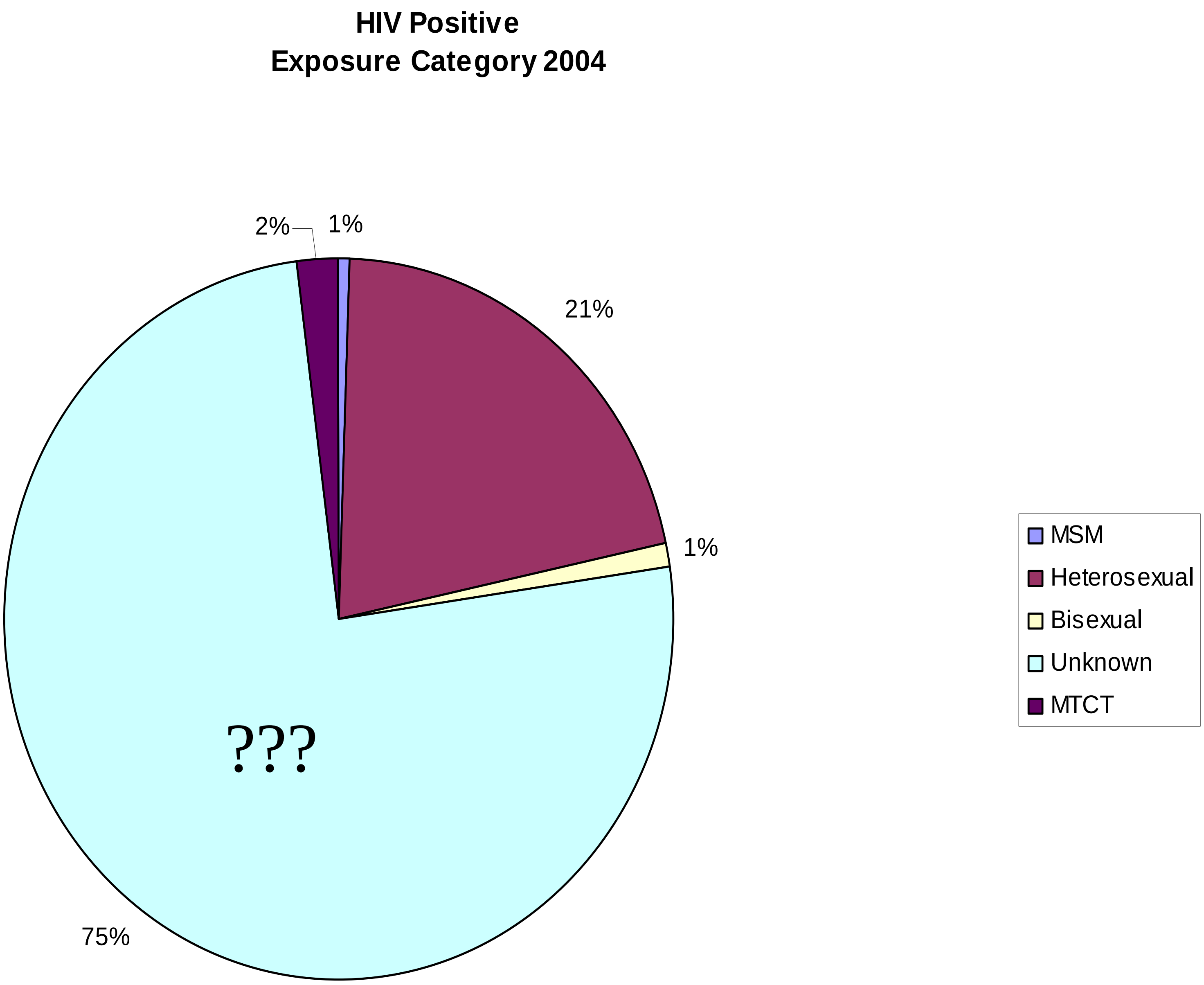

Dr Nilesh Buddha, NSU, MoH, T&T

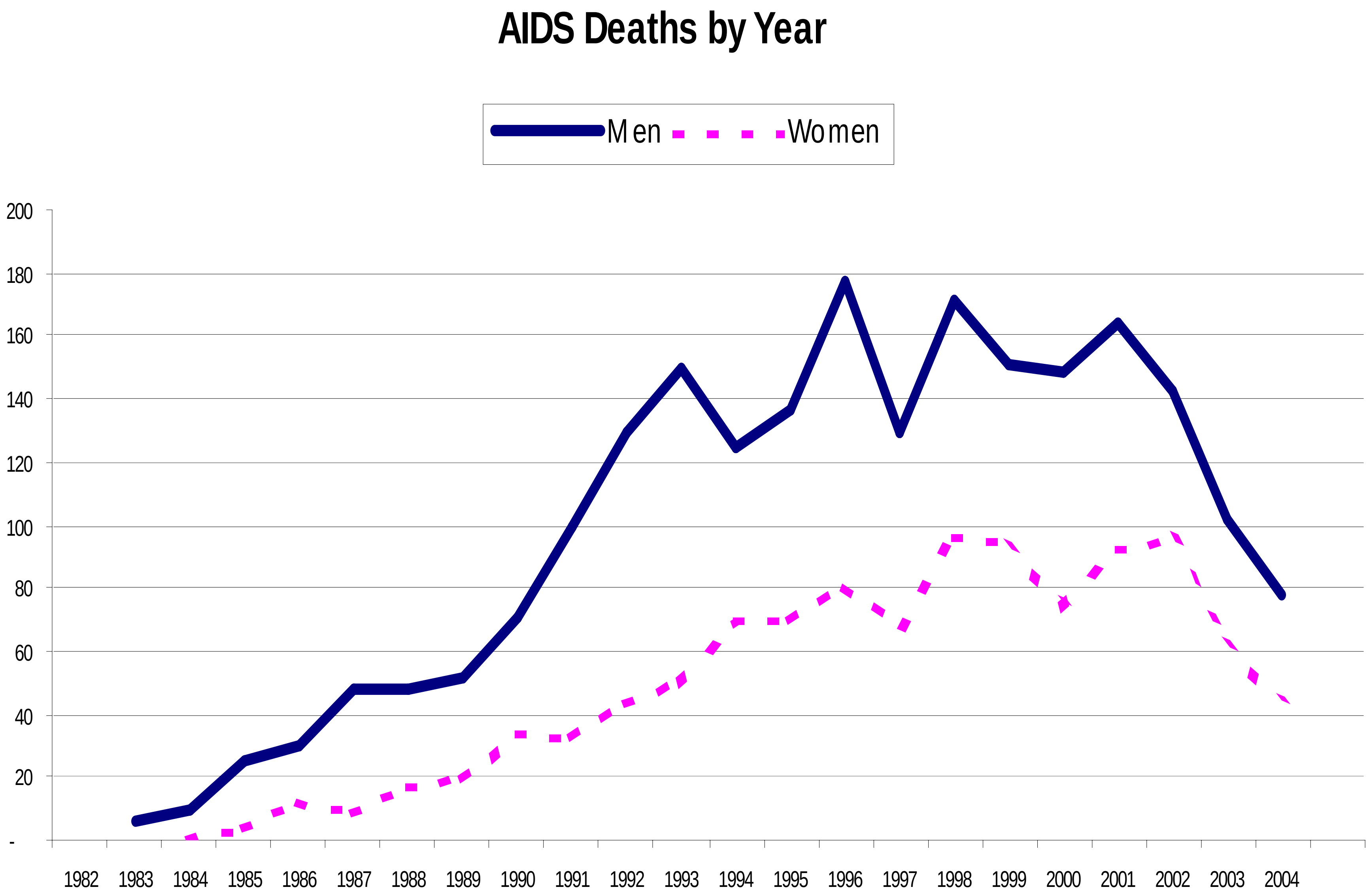

Dr Nilesh Buddha, NSU, MoH, T&T

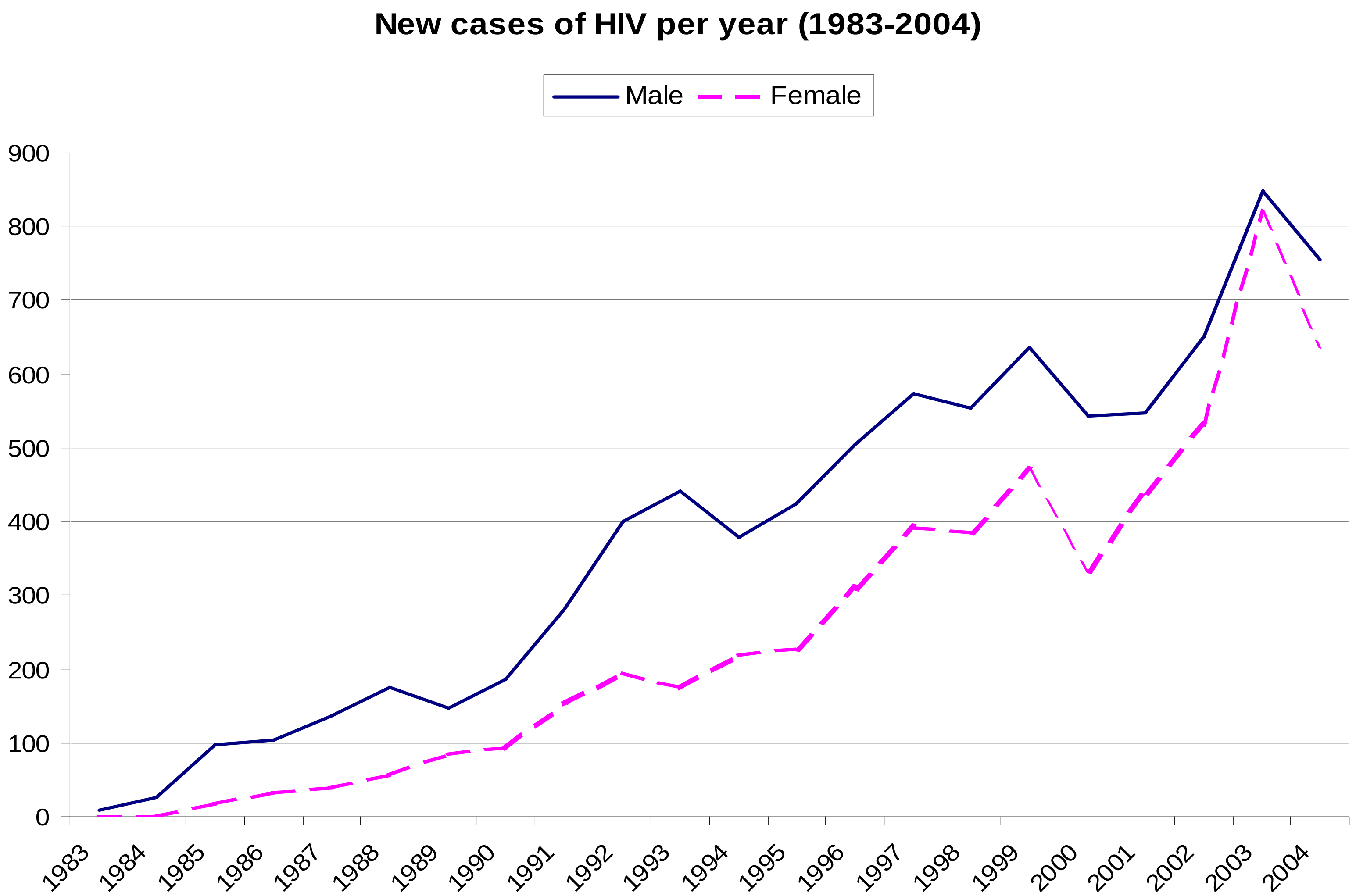

Dr Nilesh Buddha, NSU, MoH, T&T

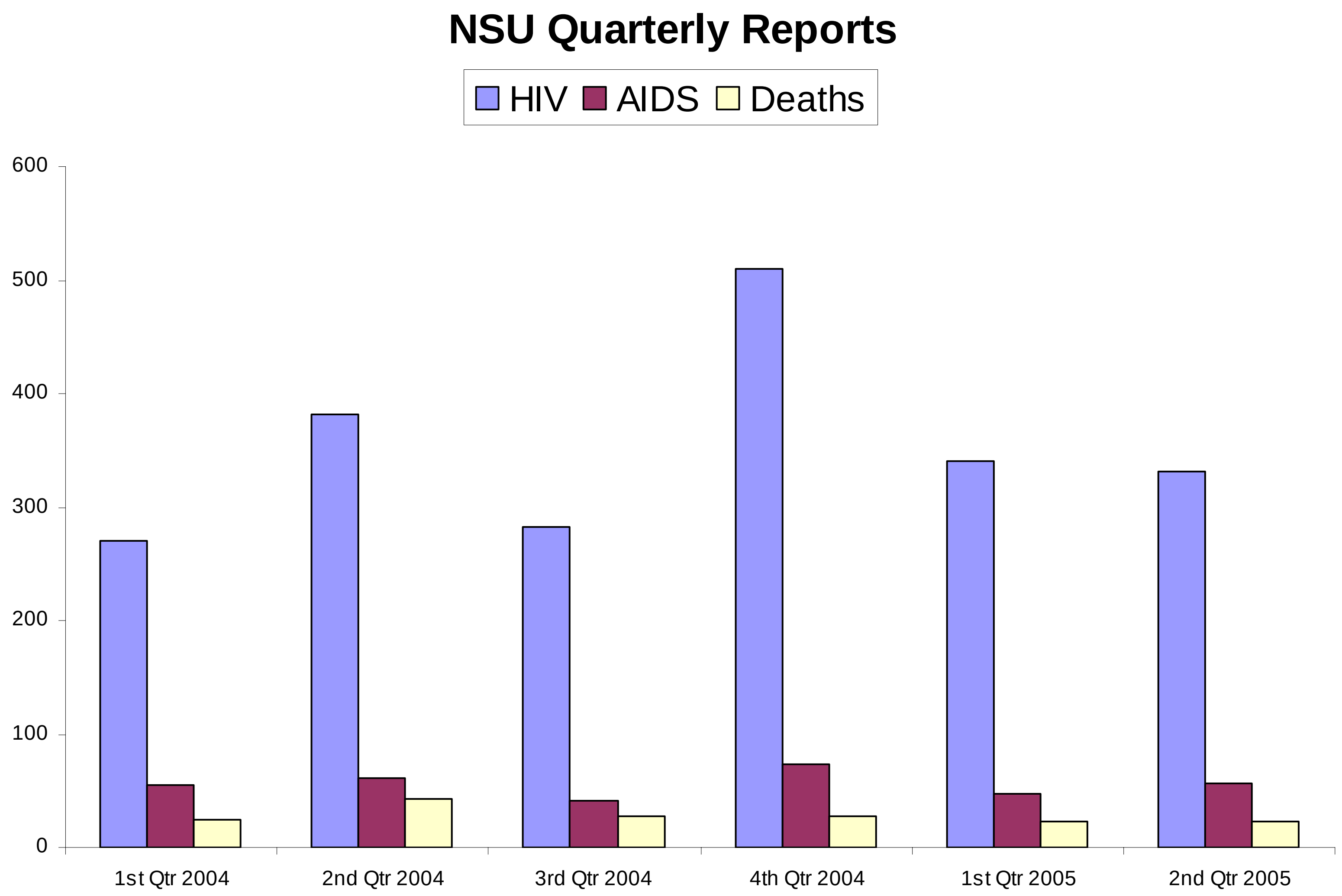

Dr Nilesh Buddha, NSU, MoH, T&T

Incidence

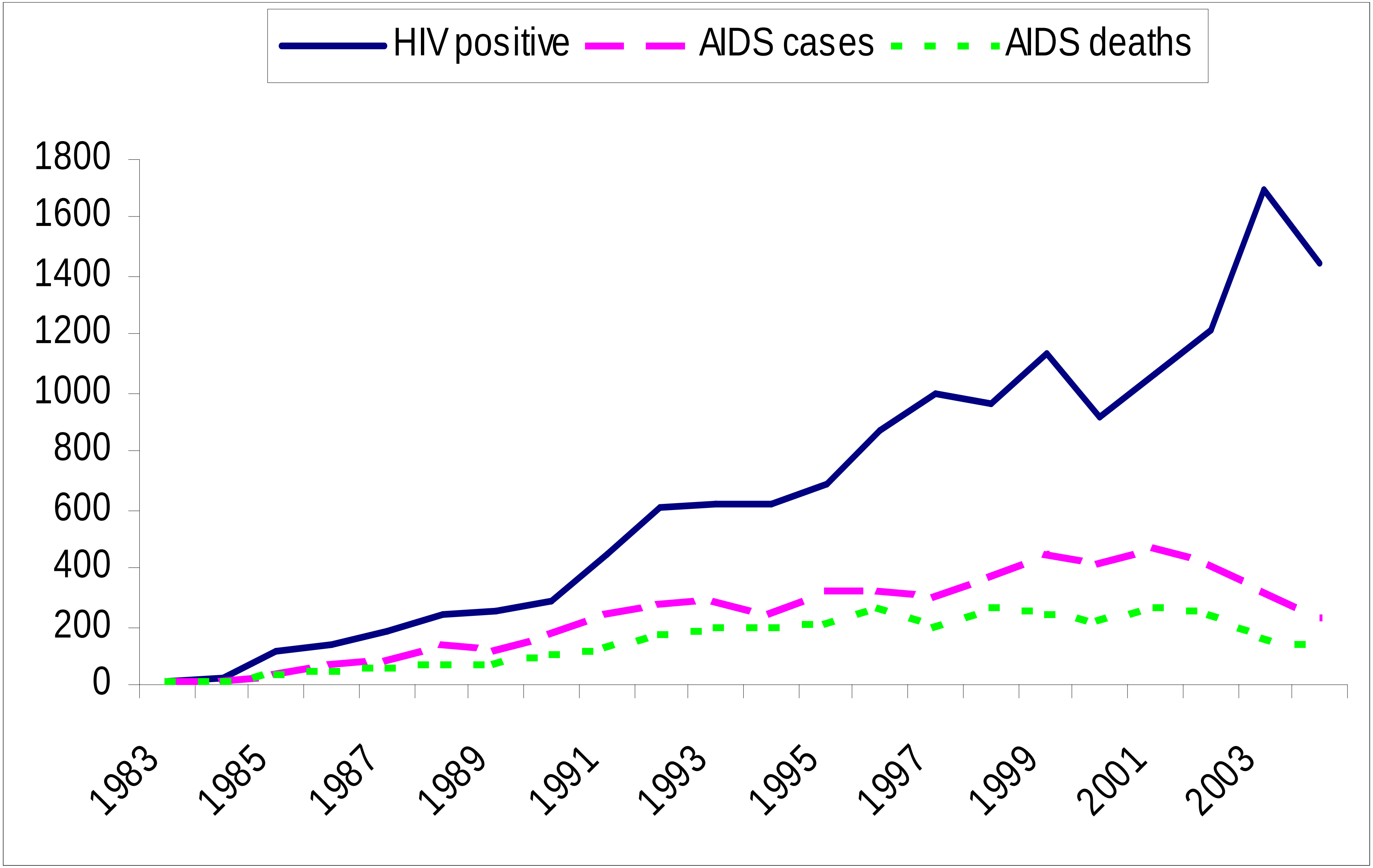

Dr Nilesh Buddha, NSU, MoH, T&T

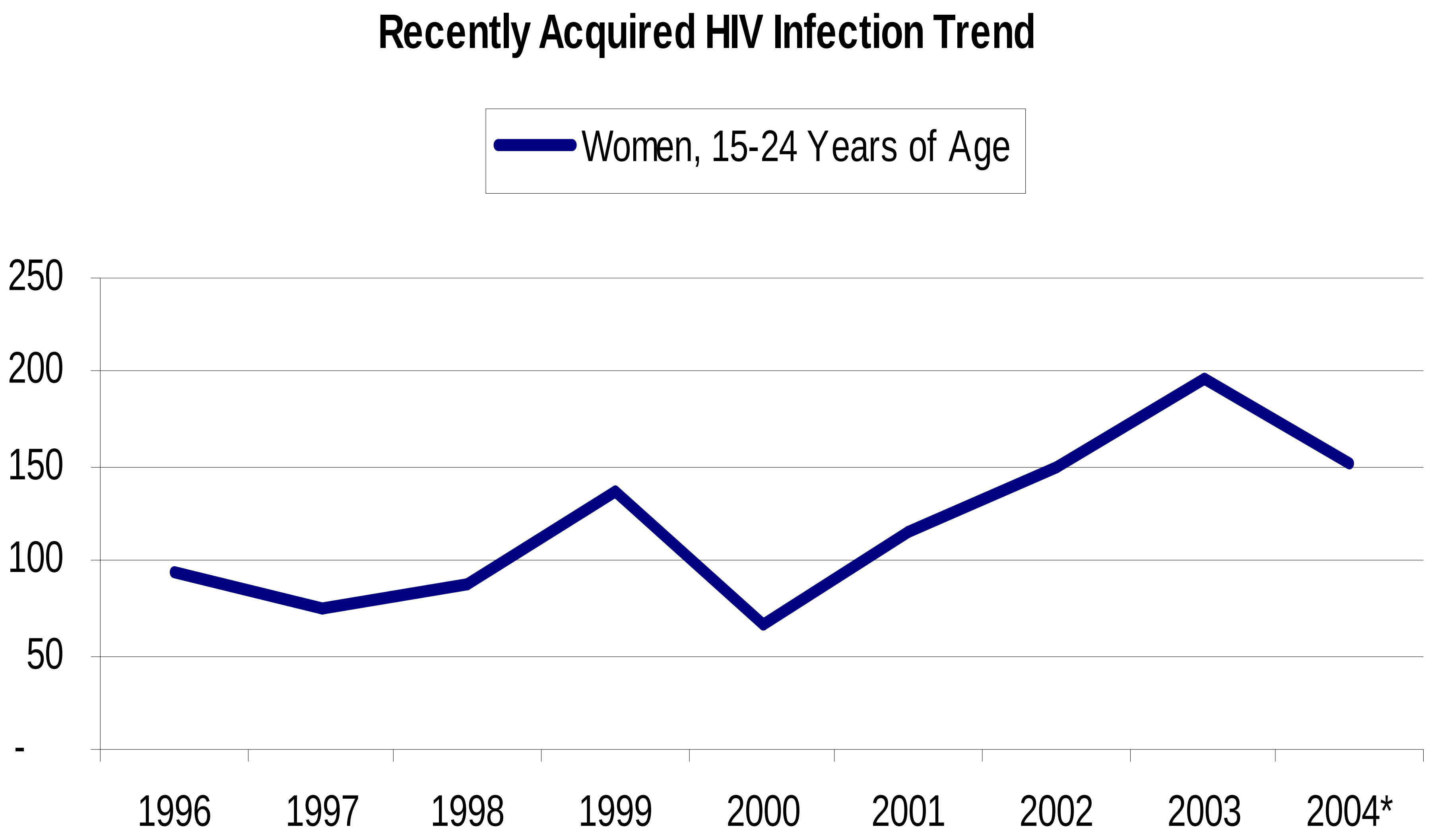

Recently Acquired HIV Infection Trend
Women, 15-24 Years of Age
250
200
150
100
50
-
1996
1997
1998
1999
2000
2001
2002
2003
2004*

Cumulative (1983 - 2004), by age

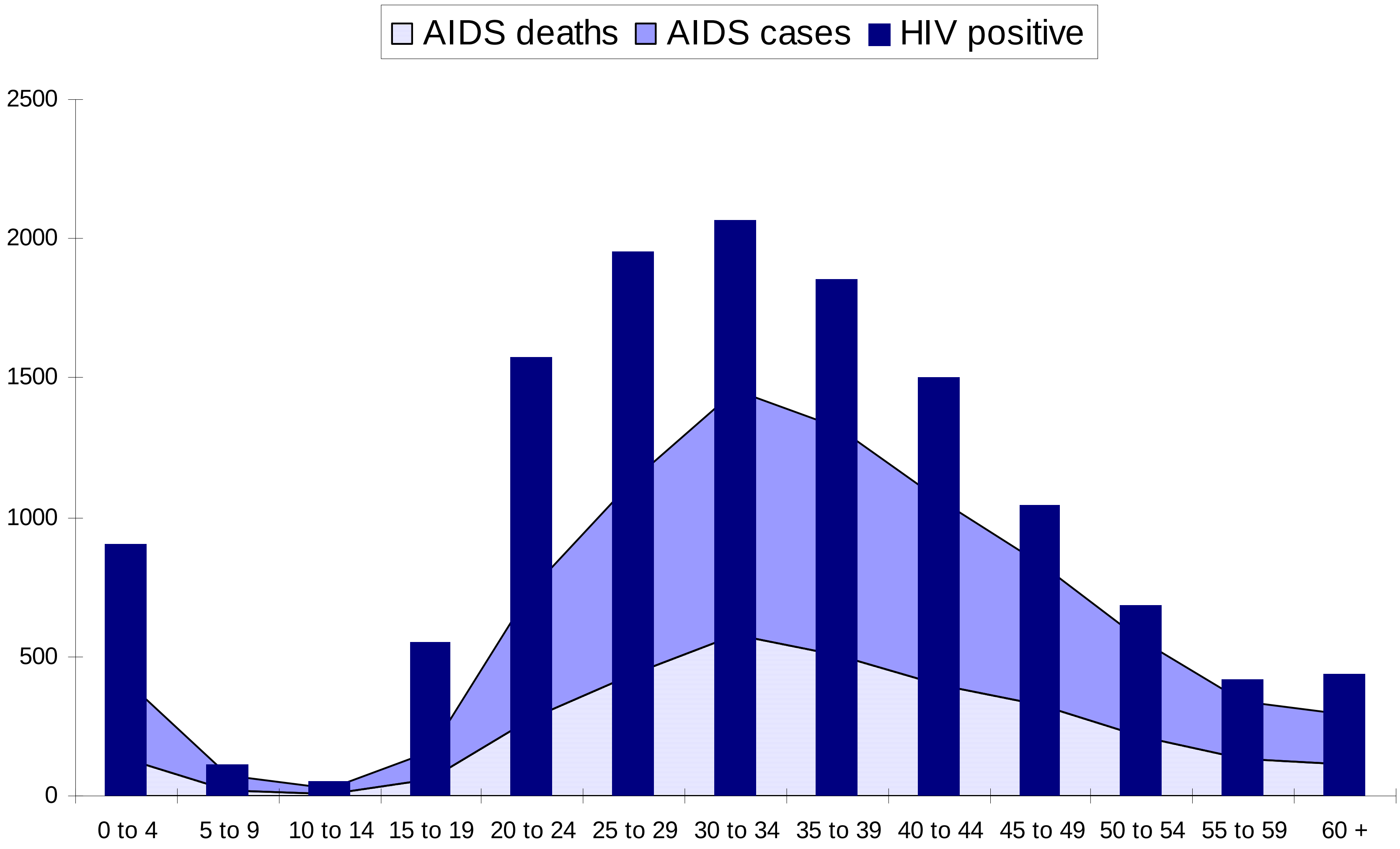

Dr Nilesh Buddha, NSU, MoH, T&T

Counti-wise Incidence, 2004

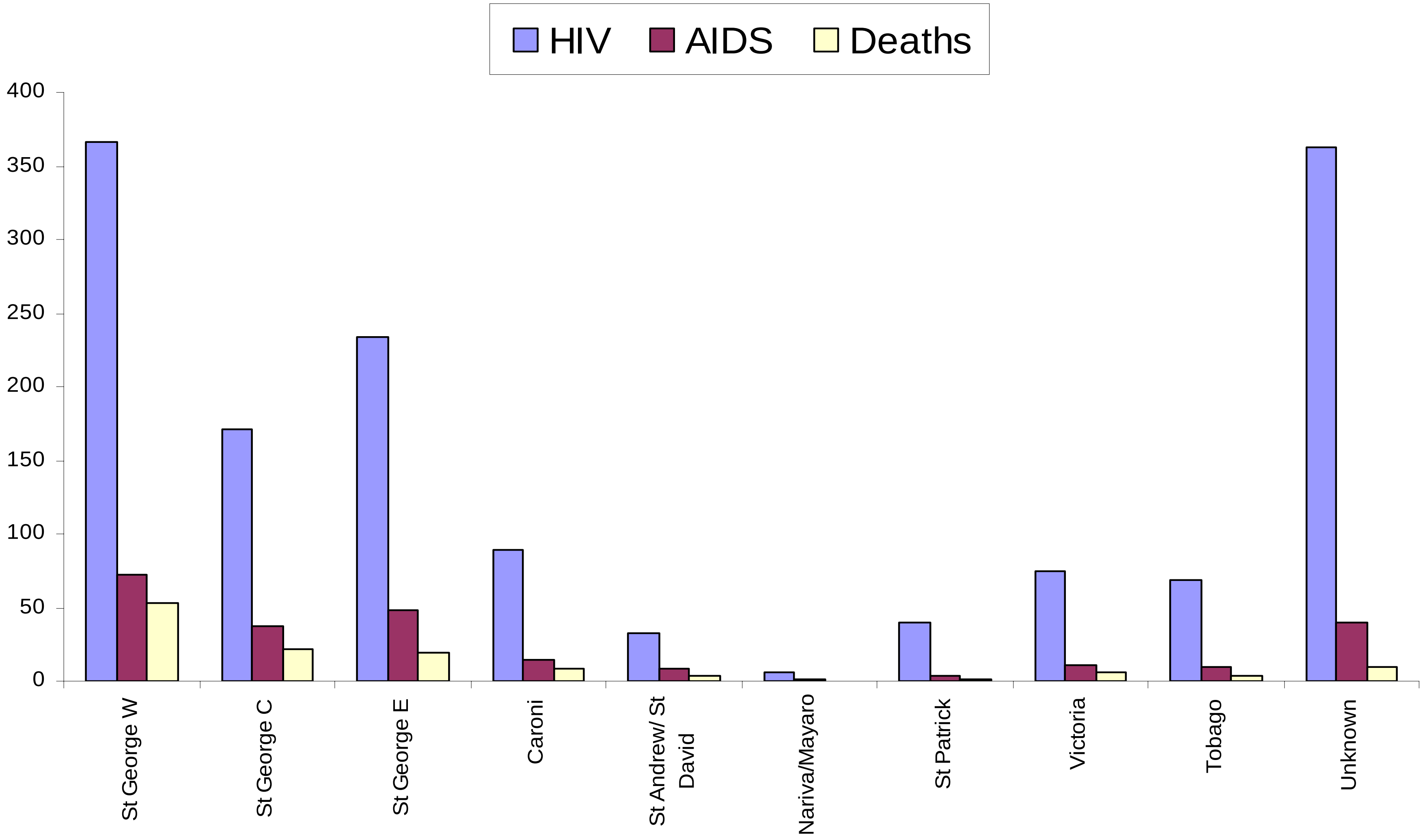

Dr Nilesh Buddha, NSU, MoH, T&T

HIV Positivity rate

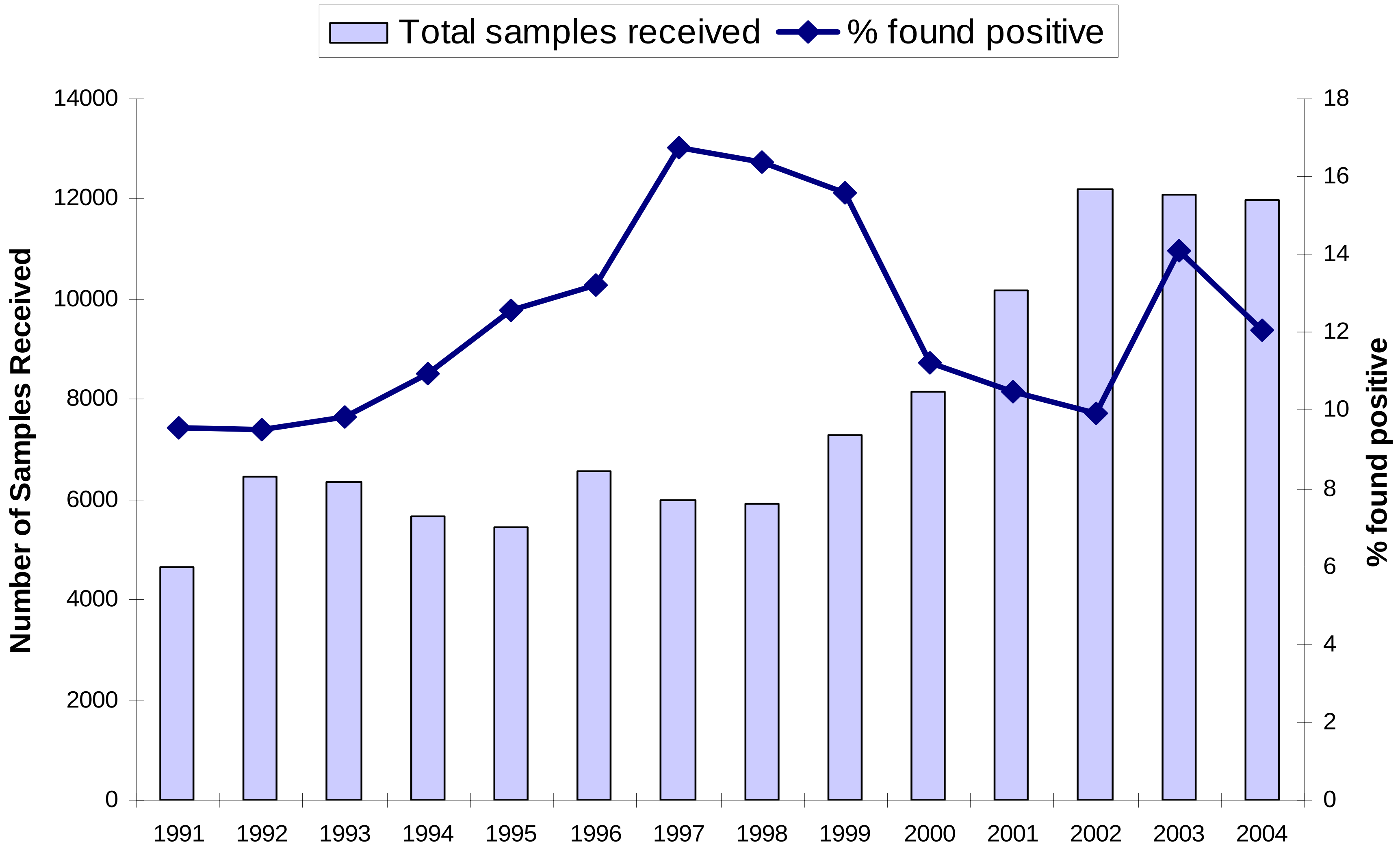

HIV Positive

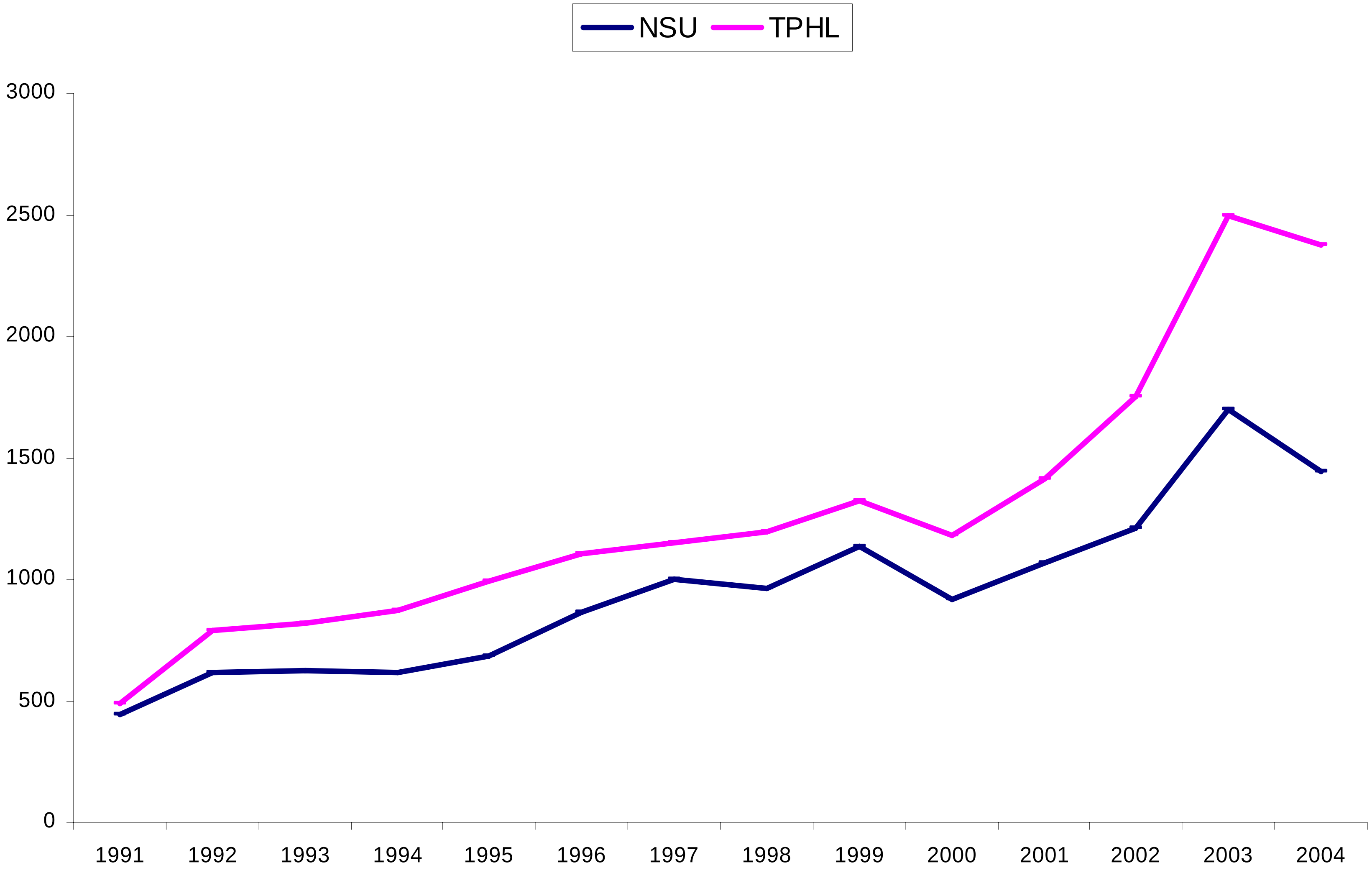

Dr Nilesh Buddha, NSU, MoH, T&T

HIV Positive Duplicates identified at NSU

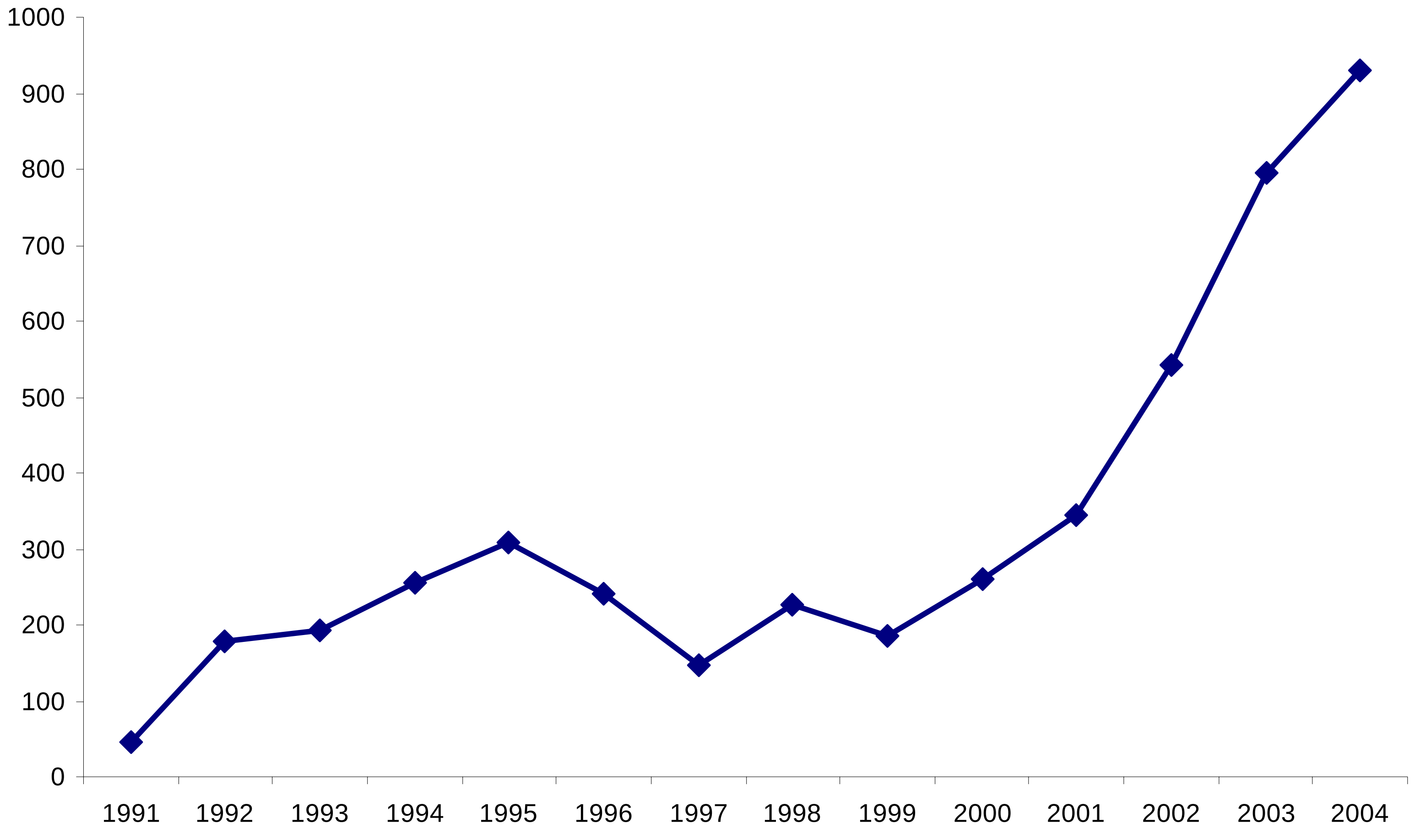

Dr Nilesh Buddha, NSU, MoH, T&T

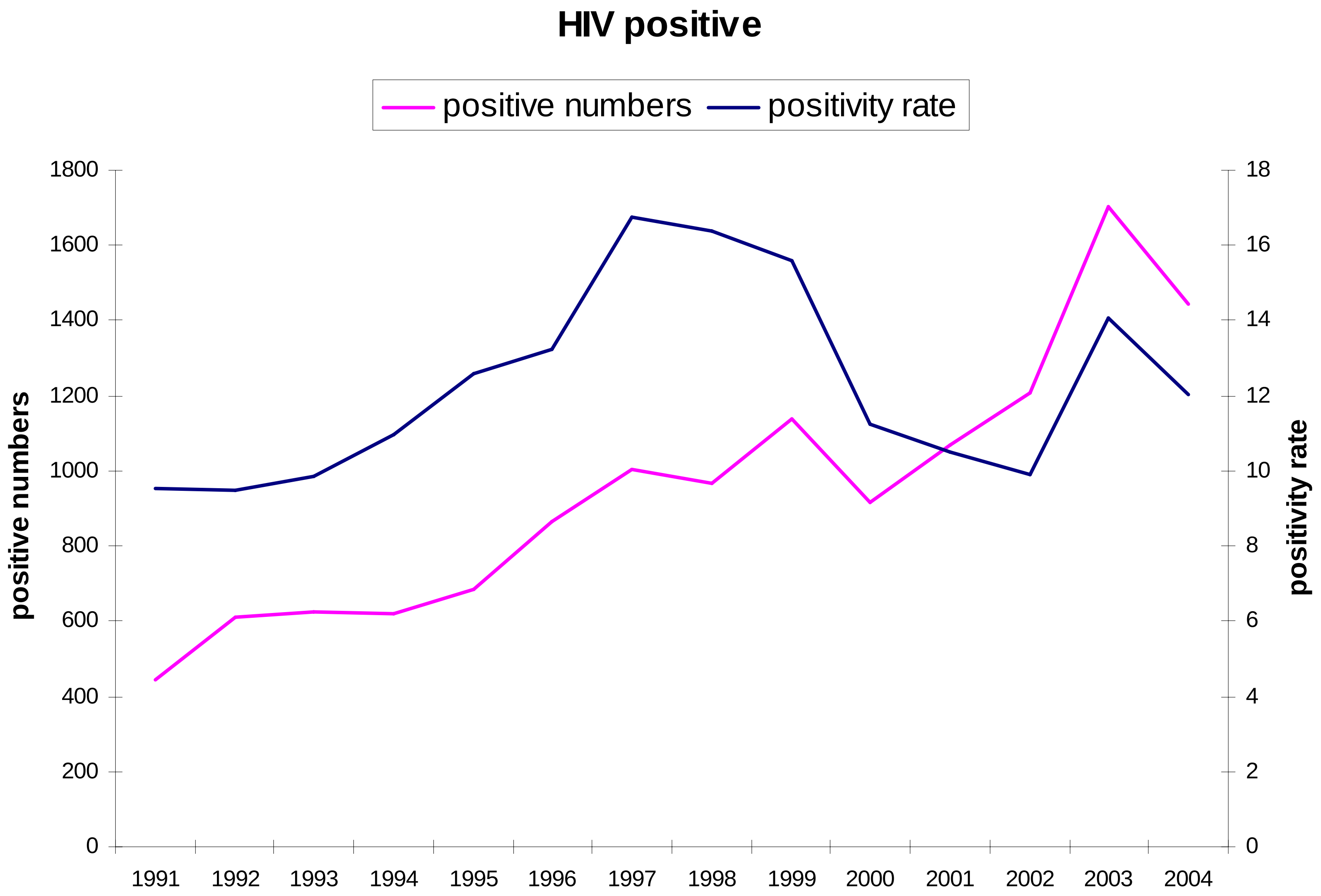

Dr Nilesh Buddha, NSU, MoH, T&T

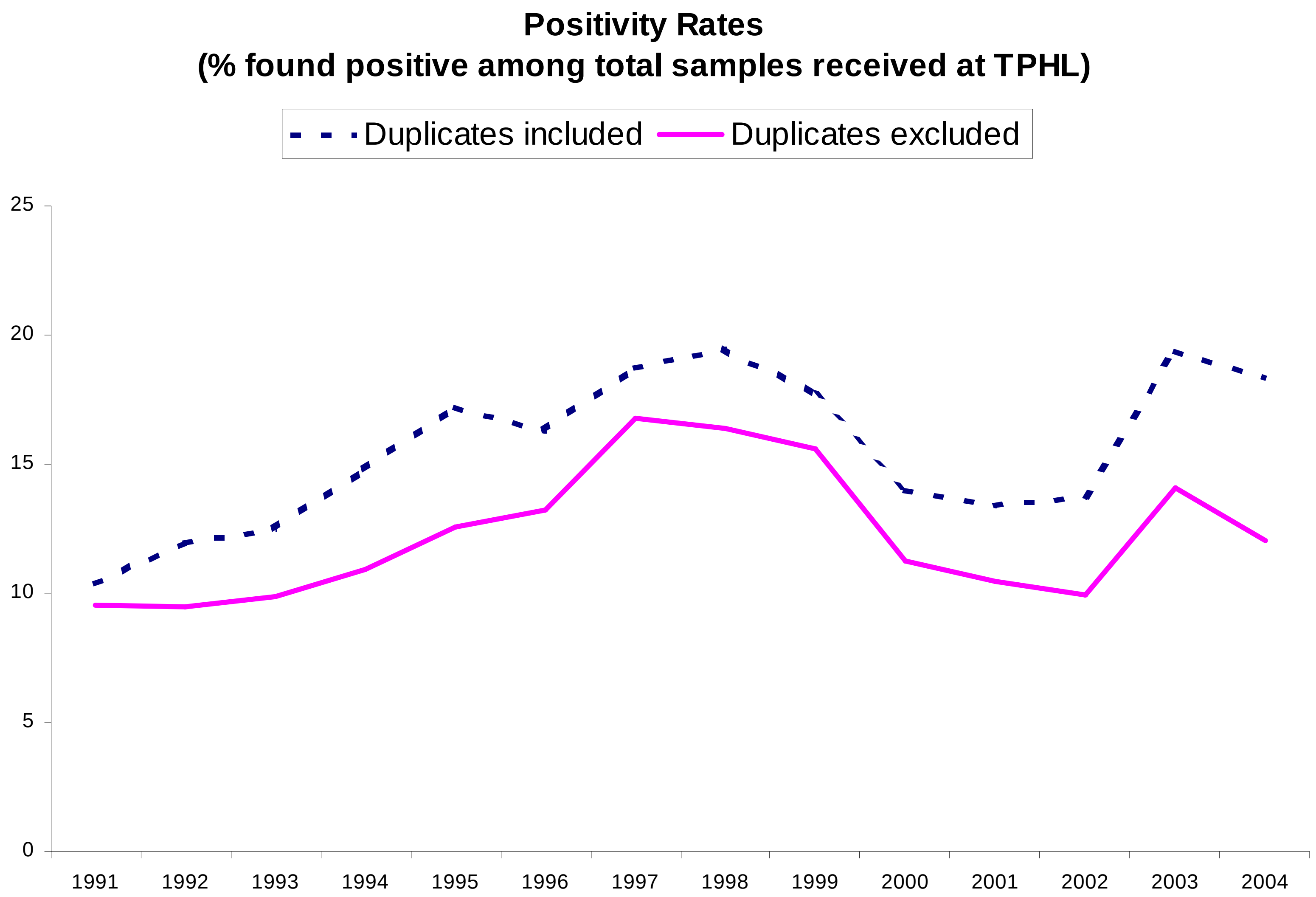

Dr Nilesh Buddha, NSU, MoH, T&T

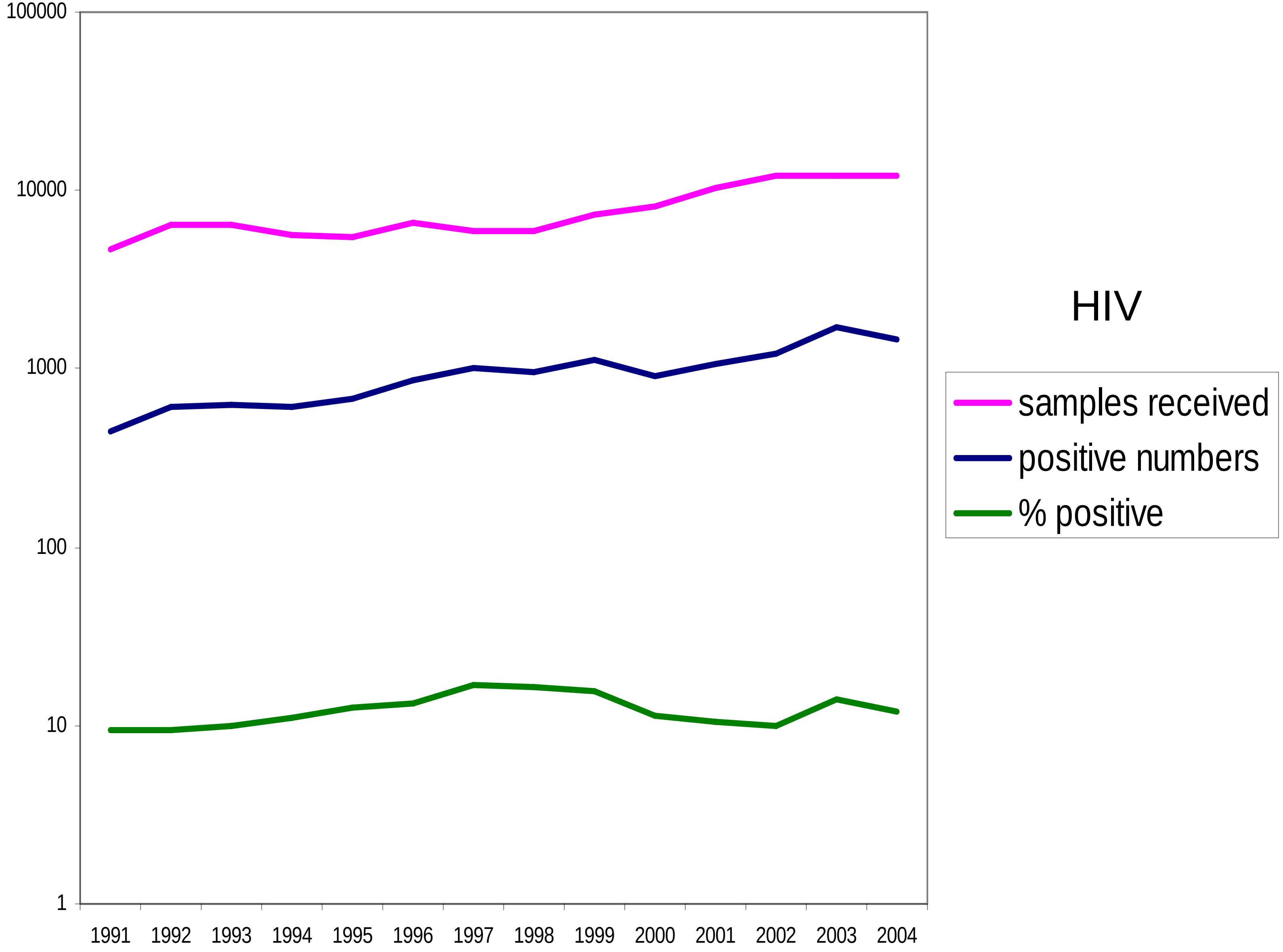

Dr Nilesh Buddha, NSU, MoH, T&T

2004, Major Providers (more than 80% of all tests performed & positive tests)

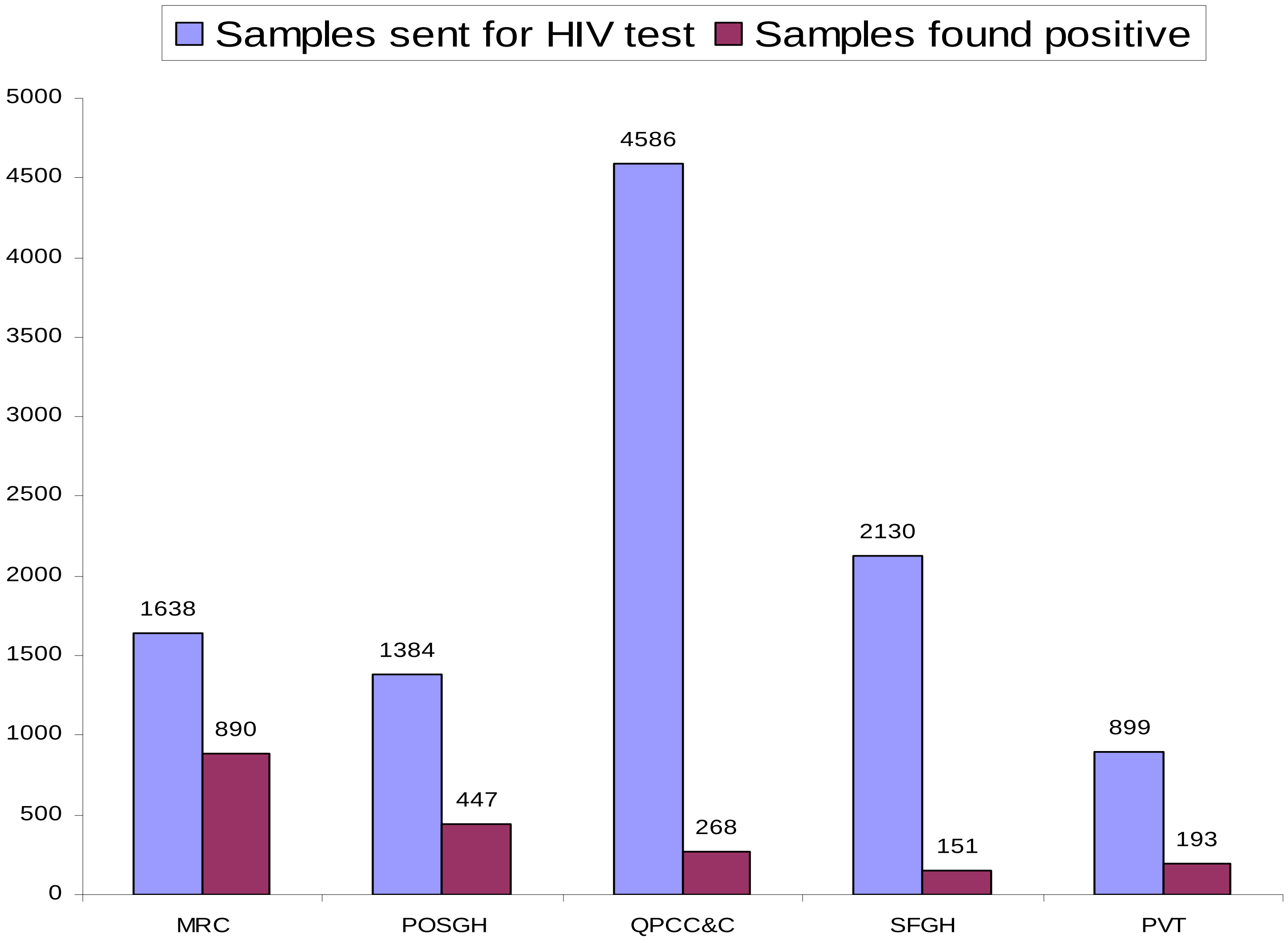

Dr Nilesh Buddha, NSU, MoH, T&T

% positive of total samples sent to TPHL for HIV testing in 2004

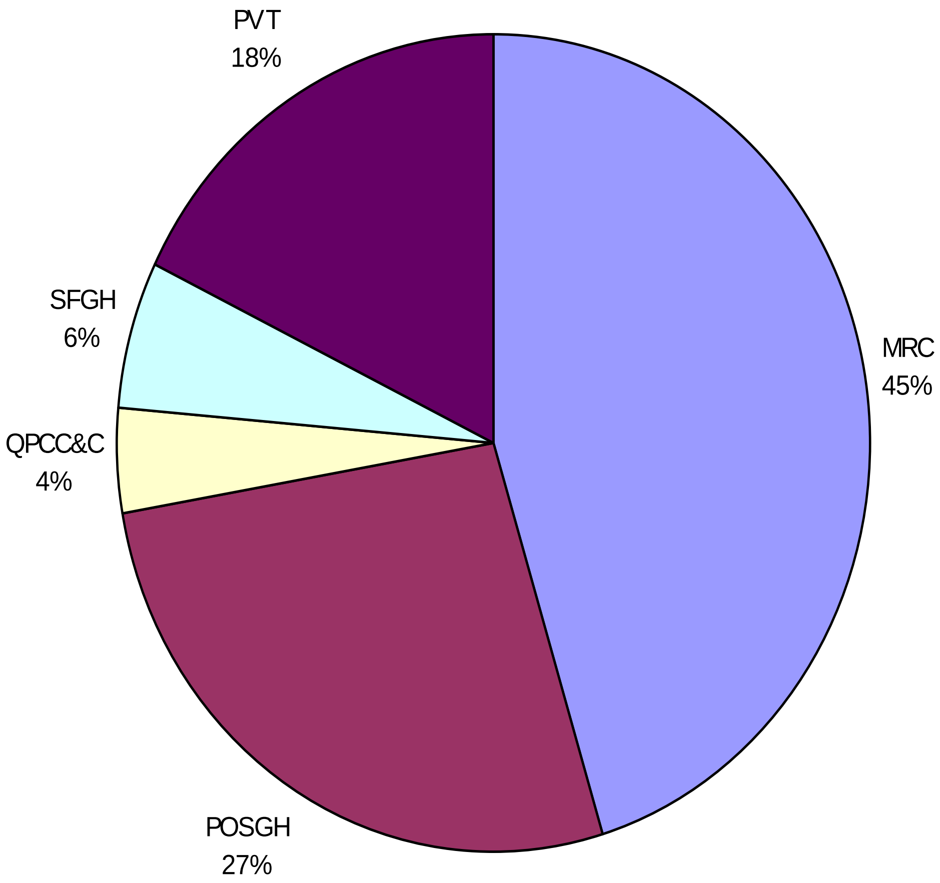

Total >80% of all positive in 2004

Dr Nilesh Buddha, NSU, MoH, T&T

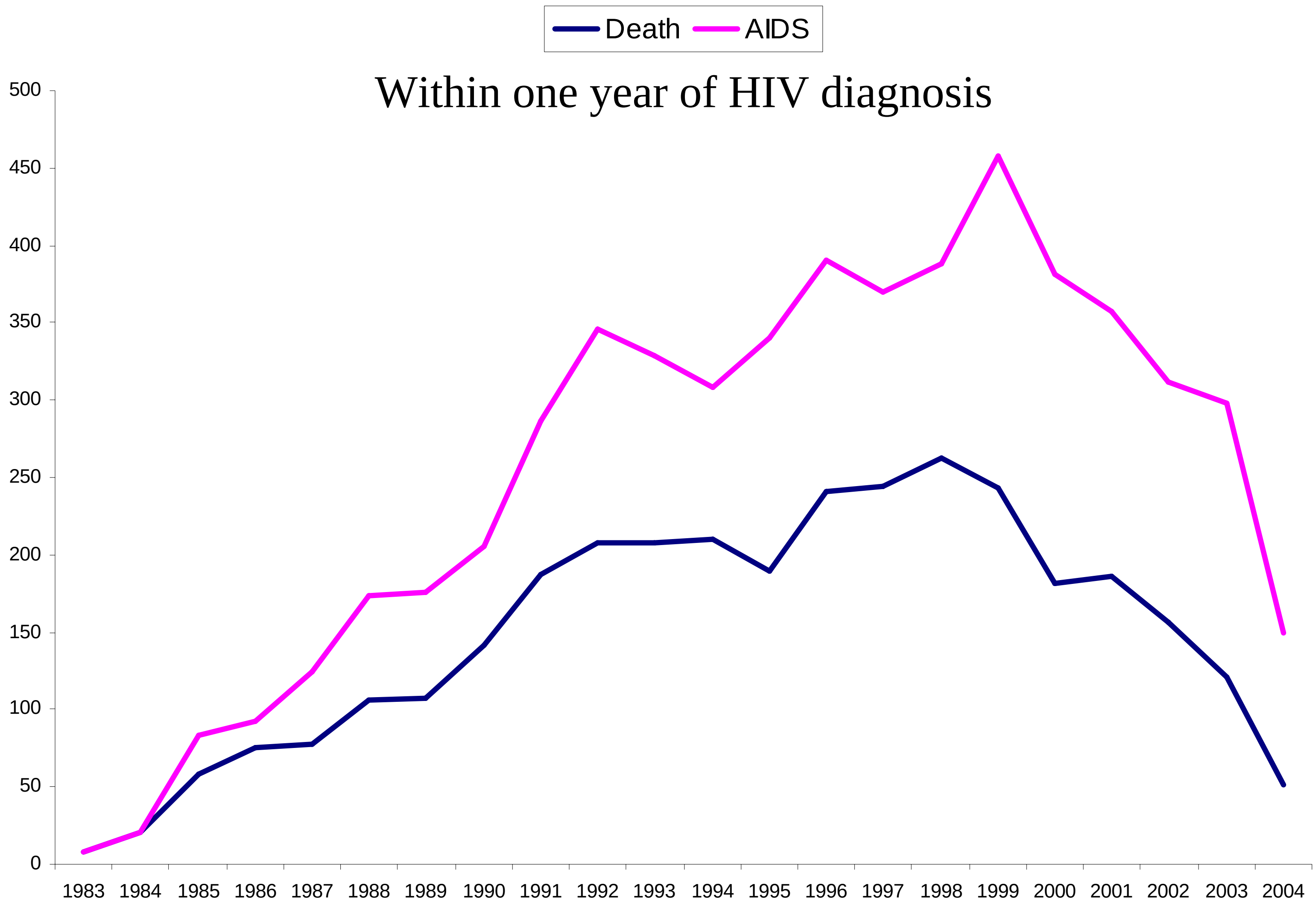

Dr Nilesh Buddha, NSU, MoH, T&T

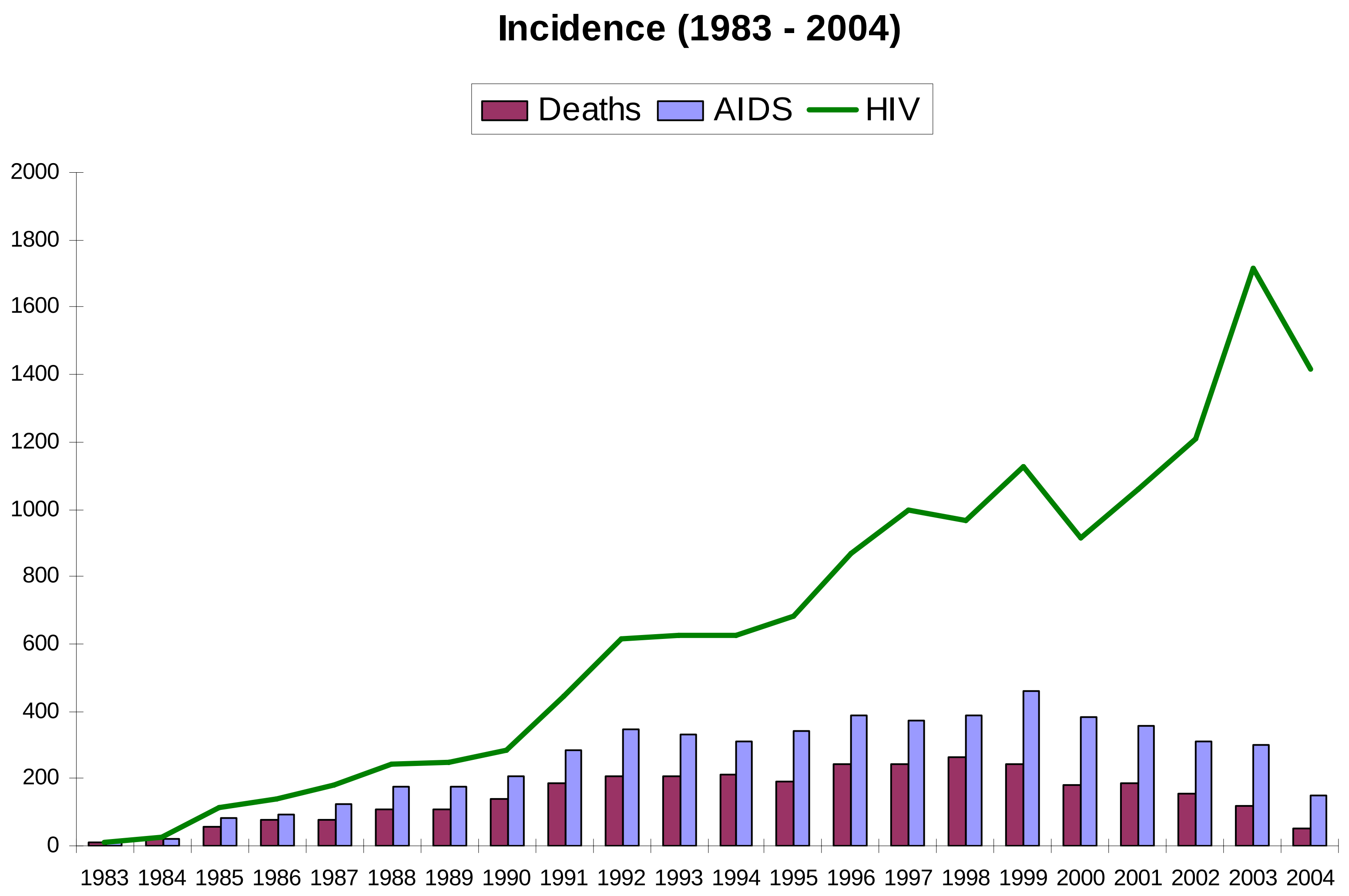

Dr Nilesh Buddha, NSU, MoH, T&T

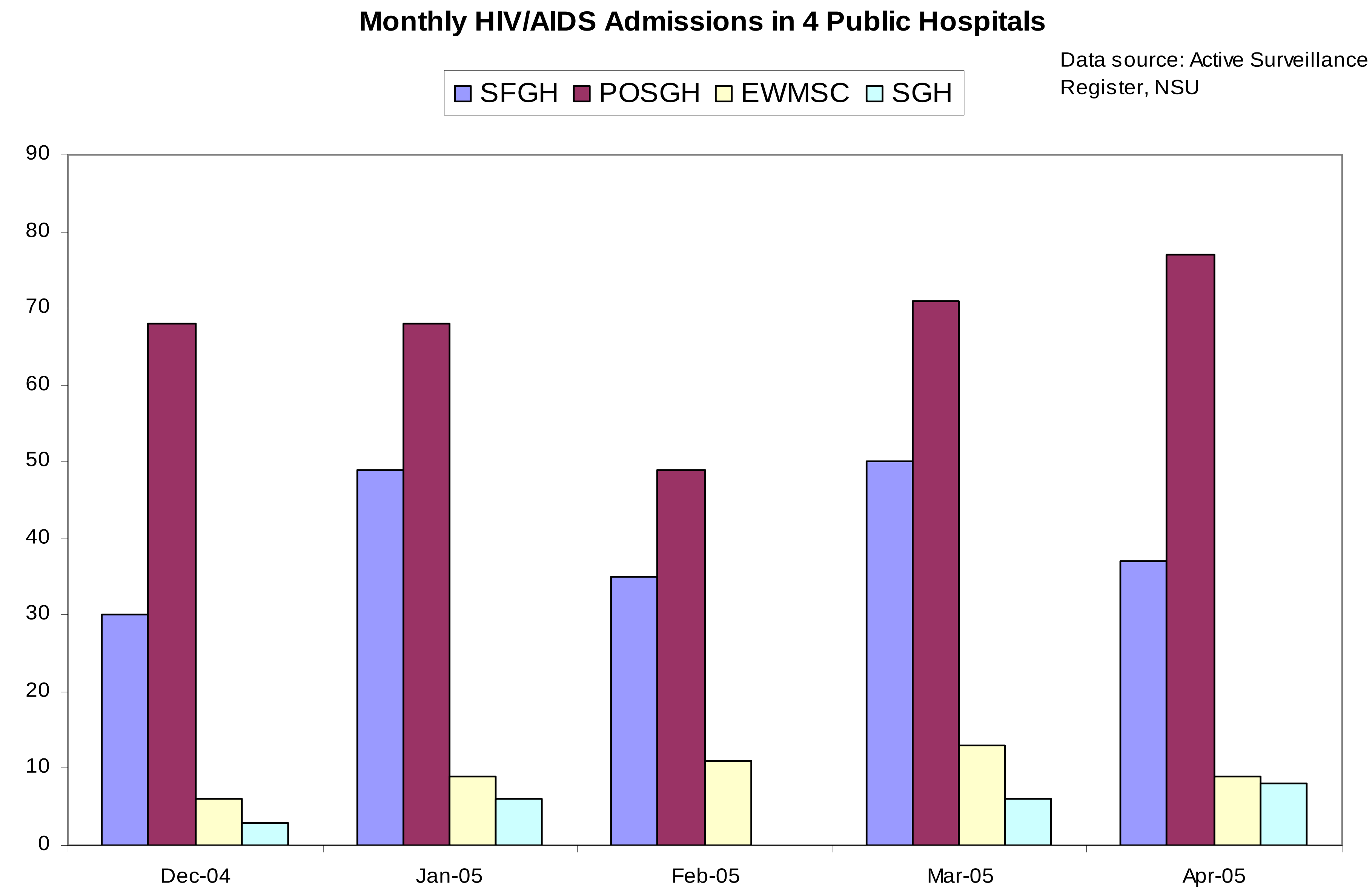

Dr Nilesh Buddha, NSU, MoH, T&T

Features

- Principle mode of transmission – Heterosexual, multiple sexual partners, unsafe sex
- Principle age at transmission – young females, adult males in all age groups
- Young men missed-out in VCT – remain continued source of infection
- Condom usage ? Substance abuse ? Ethnicity ?
- Occupation, education, socio-economic status ?
- ARV works, more resources will be required for sustained Care & Treatment

Features (contd)

- HIV/AIDS scenario at very crucial stage in 2005; sustained efforts in Prevention, Care & Treatment will yield very good returns
- Proximal causes – risky behaviours
- Solution – behavioral change communication (population-wide & high-risk targeted)
- Underlying causes – socio-economic (deprived, poor, disrupted families, uneducated, unemployed)
- Ultimate solution – social change, socio-economic rehabilitation of the marginalized

How much persuasive?

- If u can’t find a woman, take a man
- Deputy is essential
- Rum till I die

Public Health Axiom

- Population average determines numbers of deviant individuals (e.g., alcohol, blood pressure, smoking, high risk behaviour)
- We are all responsible for all
- Let’s think out of box

Population average has to change to reduce the high risk/ deviant individuals

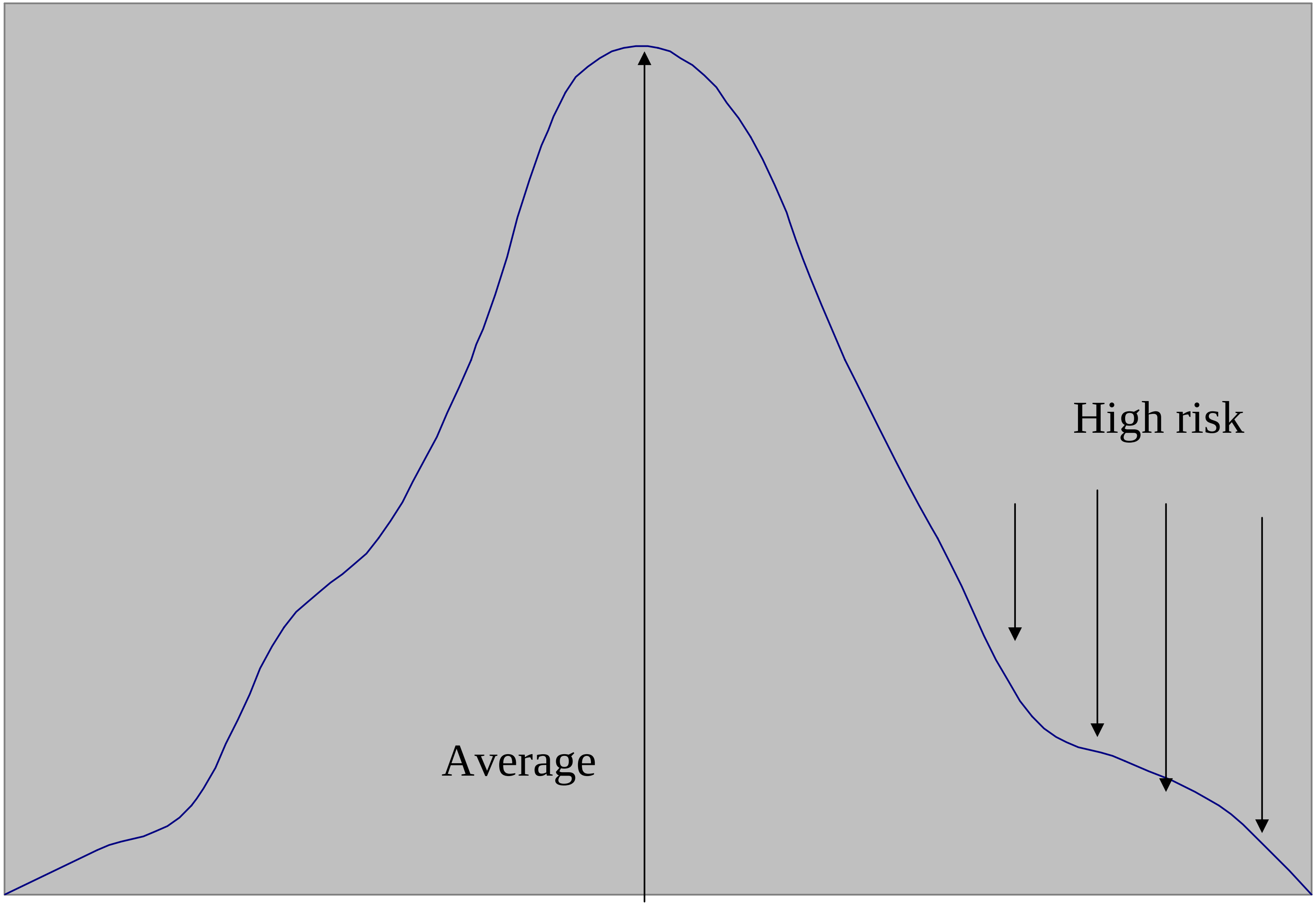

Small steps, big rewards

- Post-test Form of HIV+ at MRC
- Encoding Vs name-based, confidential reporting
- Ethnic data
- TPHL data analysis
- Explore possible synergies to avoid
  - Duplication & wastage of resources

Big steps, big rewards

- AIDS notifiable disease
- Private sector involvement
- Behavioural studies, surveys
- TB & STD: co-morbidity

Surveillance Operational Guidelines

- Roles & responsibilities
  - National, Region, County
- Reporting
  - Who, whom, when, what
  - Flow chart & formats
- Data management
  - Security, confidentiality, coding

Salient features

- Provider requests test
  - Public & private sector including laboratories
- TPHL reports positive test results to
  - Provider, CMOH and NSU
- County gets the post-test form filled-out and sends to NSU on weekly basis
- County reports monthly summary to RHA
- RHA plans & implements programmes
- NSU reports quarterly & annually

Salient features

- NSU nurses fills-out post-test form of the AIDS cases found in the 4 major hospitals during active surveillance
- RHA to assume above responsibilities once its surveillance unit is functional
- Important: confidentiality & security at all levels

Module 2:
Integration of HIV Rapid Testing in HIV Programs

Learning Objectives

- Recognize need for HIV counseling and testing in HIV prevention programs
- Recognize need for "same-visit" HIV testing in prevention programs
- Know the value of "same-visit" HIV testing for some sites (VCT, PMTCT etc.)
- Know where "same-visit" HIV testing will be used in T&T

Content Overview

- Need for expanded access to HIV testing
- Need for more people to “know their status”
- Need to integrate testing into counseling and referral programs
- Need to integrate testing into prevention, care and support services

HIV/AIDS Program Strategy

"Innovative solutions must be found to dramatically increase the number of individuals who are tested and know their status."

- Implementation and evaluation of new and effective programmes
- Scale-up of current testing, counseling and referral services

Current Status of HIV Testing

- 95% of those infected with HIV do not "know their status"
- Only 5-10% of all people have ever had an HIV test
- Less than 10% of pregnant women have had an HIV test
- Less than 10% of health care facilities offered testing and counseling in 2002

HIV Testing Occurs in a Variety of Settings

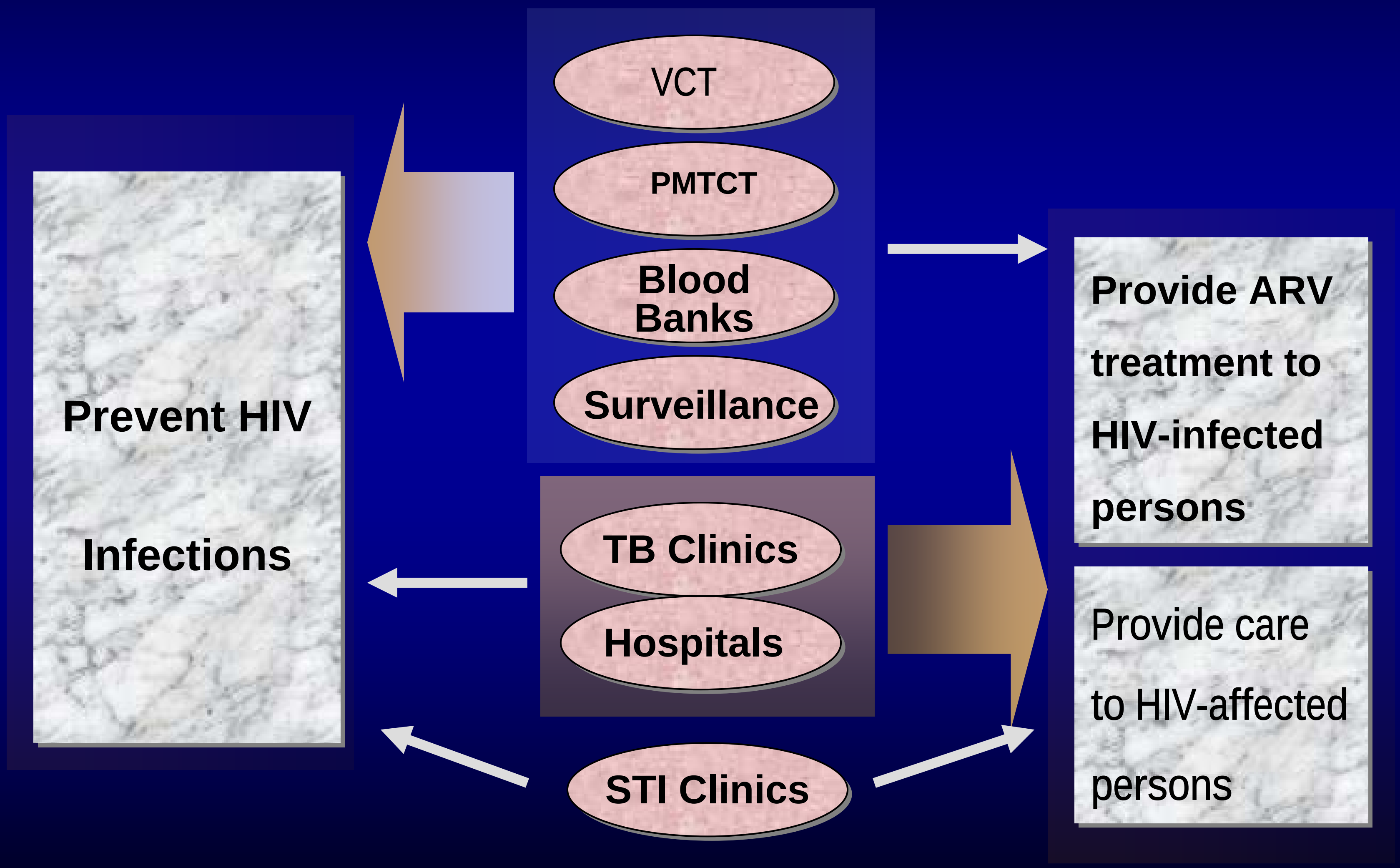

- Lab workers
- Health workers
- Counselors

HIV Testing as an “Entry Point” to other HIV Services

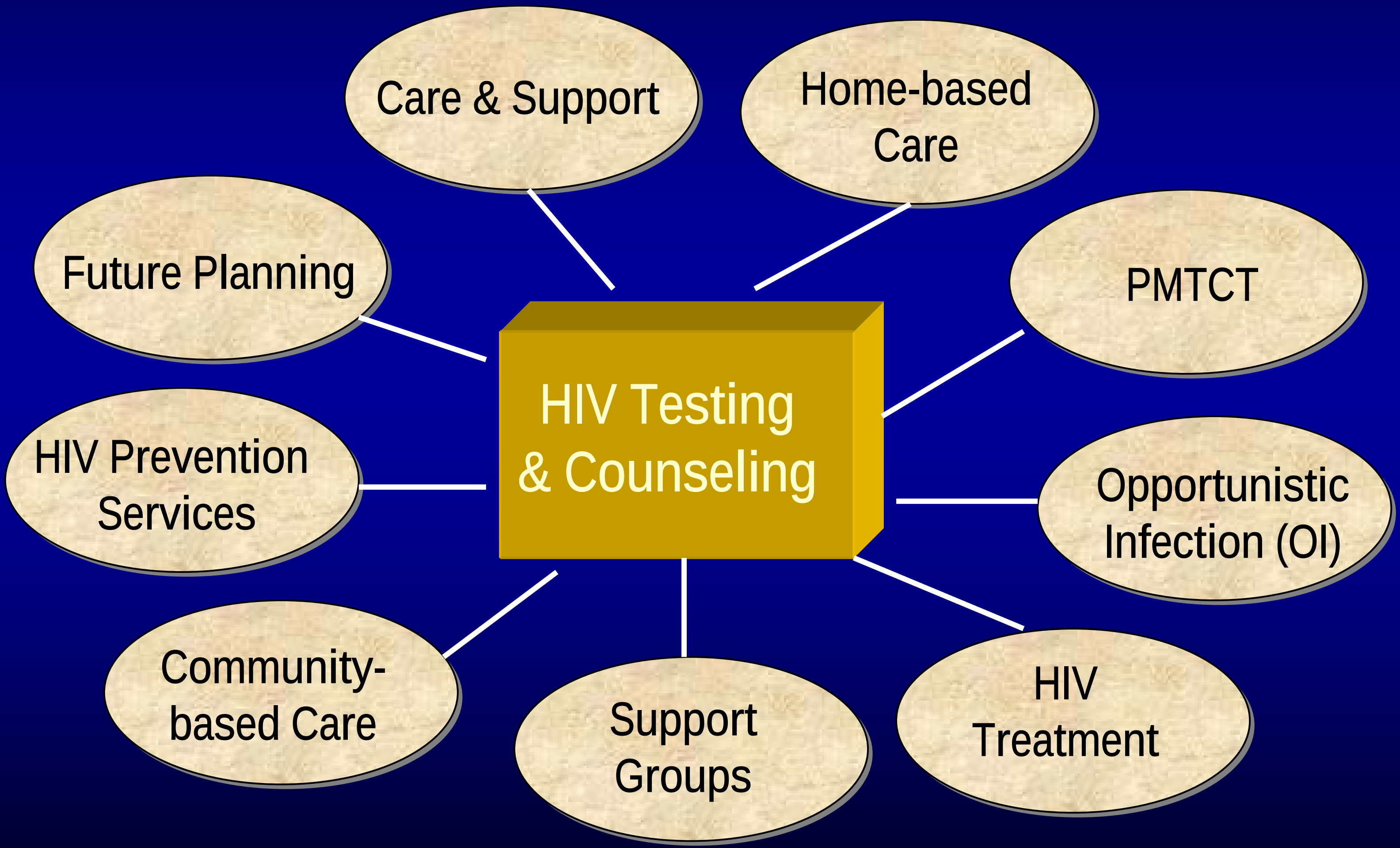

- **Lab workers**
- **Health workers**
- **Counselors**

Community-Based Counseling and Testing

- Members of the community are trained to provide counseling and testing
- Challenges: supervision; quality; confidentiality; linkage to care and treatment
- Support of community leaders is essential for success

Couples Counseling and Testing

- Facilitates disclosure and joint planning for risk reduction
- Increases utilization of HIV/AIDS services
- Allows for better family planning and care of children

Preventing Mother to Child Transmission (PMTCT)

- Prevention of HIV infection in pregnant women
- Prevention of unwanted pregnancies in HIV-infected women
- Prevention of HIV transmission from mothers to infants
- Provision of treatment and support to infected women and their families

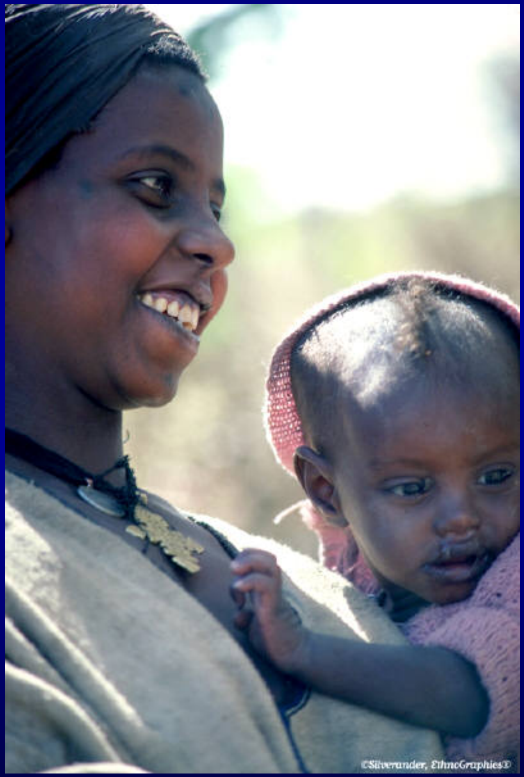

Core Interventions for PMTCT

- "Same-visit" HIV testing and counseling
- Antiretroviral treatment
- Safer delivery practices
- Safer infant-feeding practices

Reasons for “Same-Visit” HIV Testing

- Clients can be tested on site
- More clients receive test results
- Immediate intervention (ART) is possible
- Reduce HIV transmission to infants
- Eliminate need to wait for lab test results
- Reduce chance of specimen “mix-up”

International Recommendations for Counseling and Testing in PMTCT

- Group pre-test information at ANC
- Individual pre-test counseling at ANC
- Routine HIV testing at ANC and at L&D
- "Same-day" testing at ANC and at L&D
- Individual post-test counseling
- Encouraging partner testing

Source: WHO's draft CT for PMTCT (2003), CDC's MMWR 2002; CDC's Dear Colleague letter (2003); Institute of Medicine. Reducing the odds. Preventing perinatal transmission of HIV in the United States. Washington, DC: National Academy Press, 1999

Summary

- Where can HIV counseling and testing be offered?
- What are the advantages of using “same-visit” HIV testing?
- What are special advantages of using HIV rapid testing in PMTCT?
- How does HIV testing help to support access to treatment ?

Module 3:
Overview of HIV Testing Technologies

Learning Objectives

At the end of this module, you will be able to:

- Discuss integration of HIV testing into other HIV/AIDS programmes and services
- Discuss the various testing technologies for HIV
- Explain the advantages and disadvantages of HIV rapid tests
- Recognize individual HIV test results as positive, negative or invalid

Content Overview

- Expansion of HIV rapid testing
- Types of HIV tests
- Challenges in HIV testing
- Advantages and disadvantages of HIV rapid testing
- Types of HIV rapid tests
- Reading individual test results

HIV Testing Occurs in a Variety of Settings

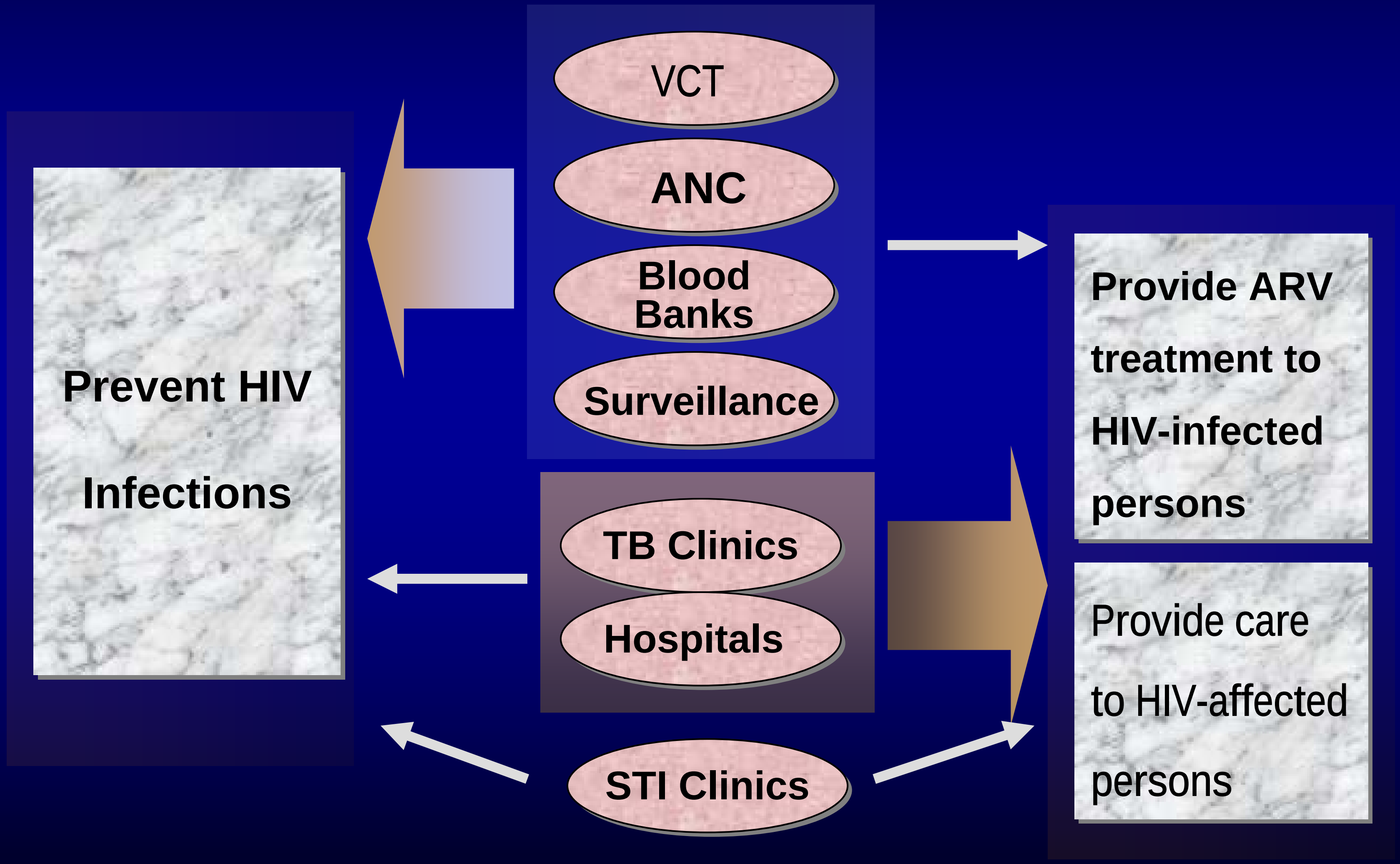

Lab workers Health workers Counselors

Expansion of Testing Services

- Integrate HIV testing services into national health systems
- Introduce testing into non-traditional settings

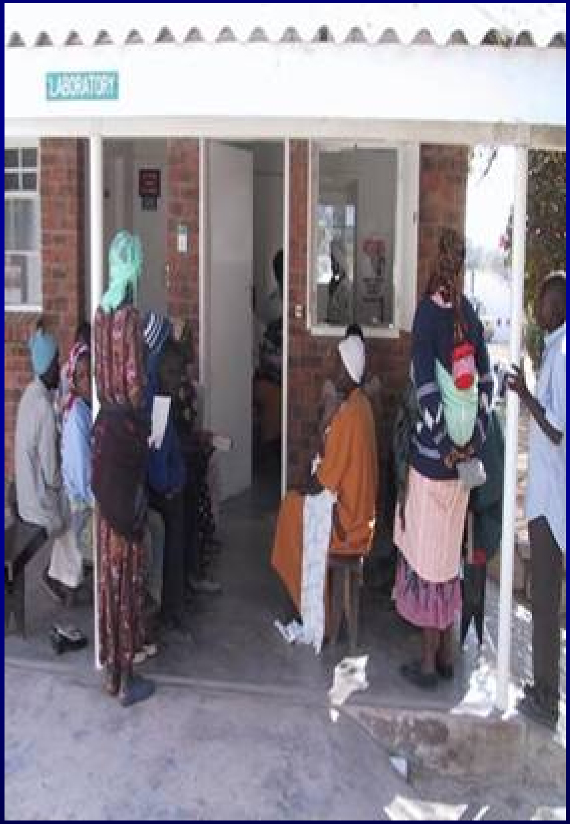

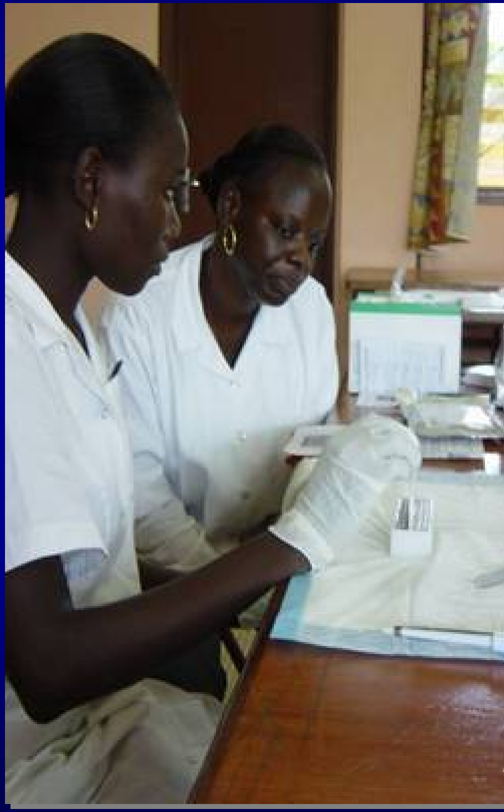

Lab workers   Health workers   Counselors

Use of HIV Testing in the Continuum of Care

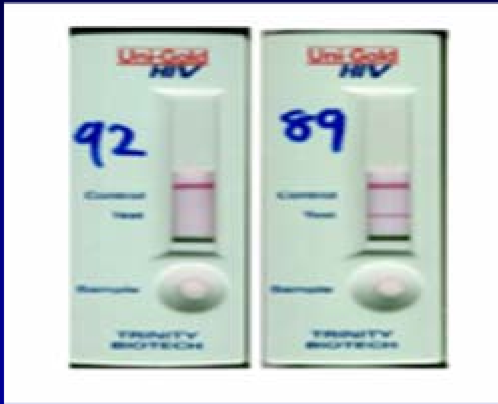

Use HIV testing to diagnose HIV infection

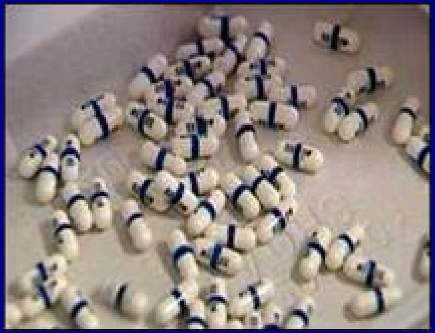

Initiate treatment with ARVs

Monitor effectiveness of ARVs with diagnostics (viral load, CD4)

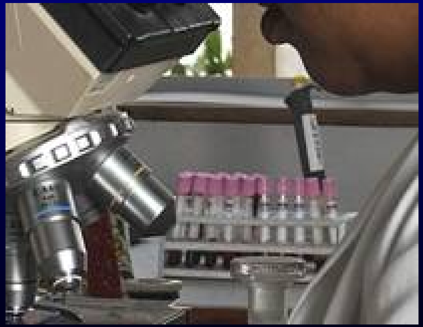

- **Lab workers**
- **Health workers**
- **Counselors**

Types of HIV Tests

- Serology (Antibody/Antigen testing)
  - Enzyme Immunoassays (EIAs)
  - Rapid tests
  - Western blot (WB)
- Lymphocytes
  - CD4 counts
  - CD8 counts
- Nucleic Acid
  - Viral Load
  - PCR

Challenges in HIV Testing

- Maternal antibody present in infants
- “Window” between infection and antibody appearance
- Not all HIV subtypes can be detected with a single test
- Other health conditions
- Laboratory requirements
- Technical skill

Enzyme Immunoassays (EIAs)

- Detect HIV antibodies to both HIV-1 and HIV-2
- Detect both HIV antibody and HIV antigen (close window period)
- Require sophisticated laboratory
- Require well trained lab personnel

Enzyme ImmunoAssays (EIAs)

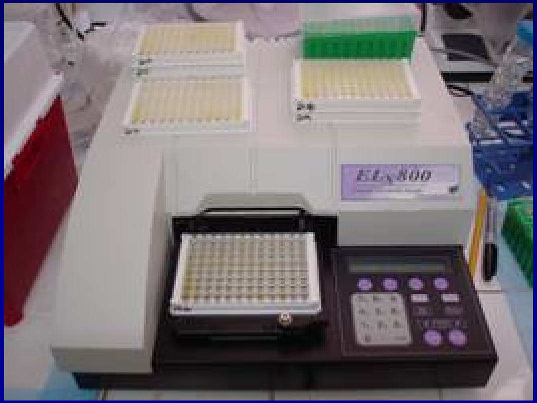

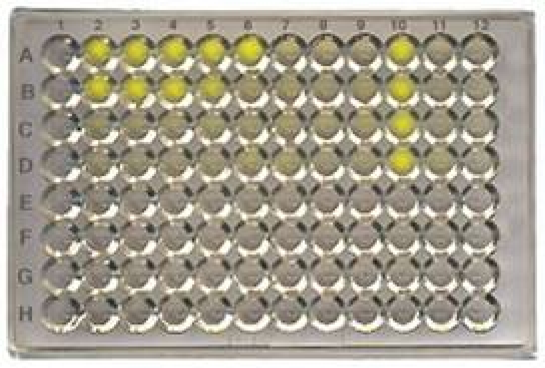

Samples placed in individual wells for testing

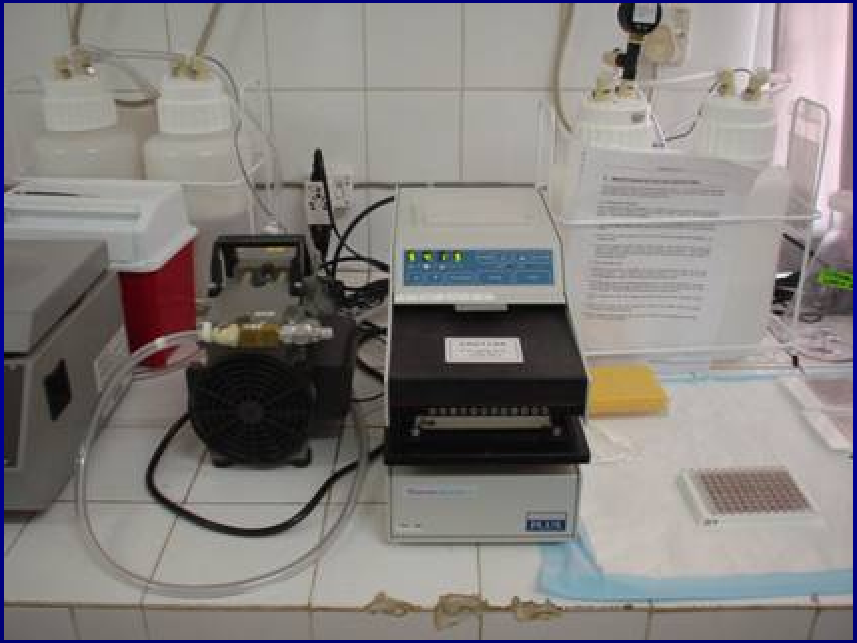

Automated reader determines result of test

Lab workers   Health workers

HIV Rapid Tests

- Detect HIV antibodies to HIV 1 and HIV 2
- Results comparable to EIA results
- Suitable for non-lab settings
- Non-lab personnel can be trained to test using these devices

Western Blot Assays

- Detects antibodies to specific HIV antigens
- Difficult to interpret
- Multiple standards for performance in use
- Expensive
- Limited availability

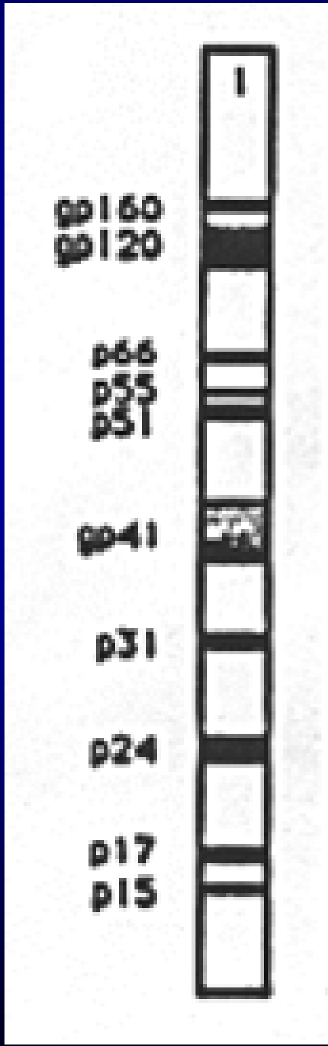

◊ Lab workers ◊ Health workers

CD4 Cell Counts

- Follow clinical progress
- Monitor therapy
- Require trained staff
- Require sophisticated equipment

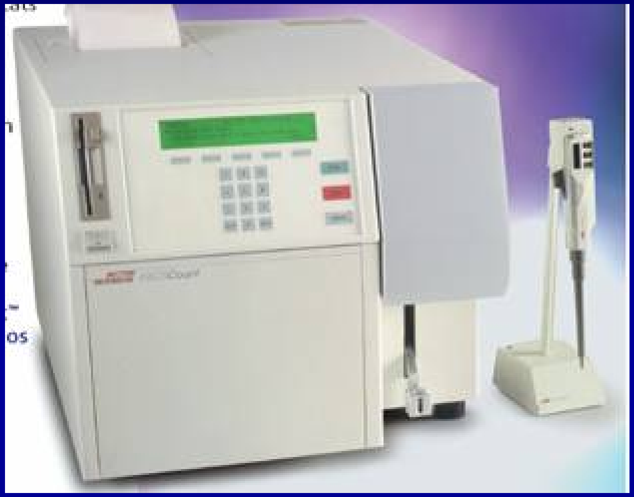

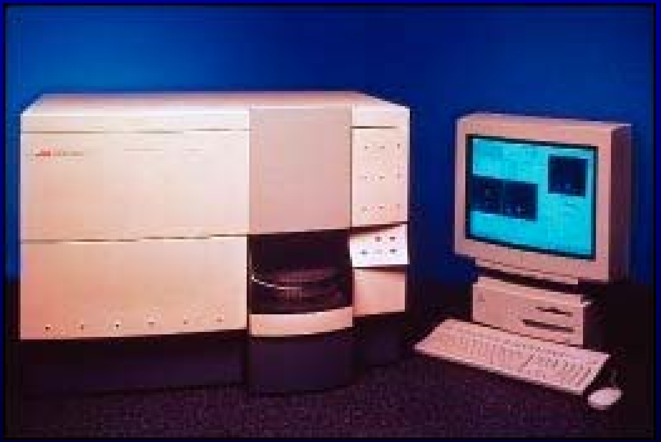

◇ Lab workers ◇ Health workers

HIV Viral Load

- Molecular assay to measure HIV levels
- Used to monitor disease progression
- Used to monitor ARV response
- Requires sophisticated lab facility
- Requires highly trained personnel
- Expensive

HIV Test Complexity*

- Level 1: No laboratory facility or experience needed
- Level 2: Multi-step process is required; Centrifuge or other equipment required
- Level 3: Specific laboratory skills such as diluting are required
- Level 4: Sophisticated equipment and well-trained laboratory staff are required

*WHO Reports

HIV Rapid Testing permits expansion of services

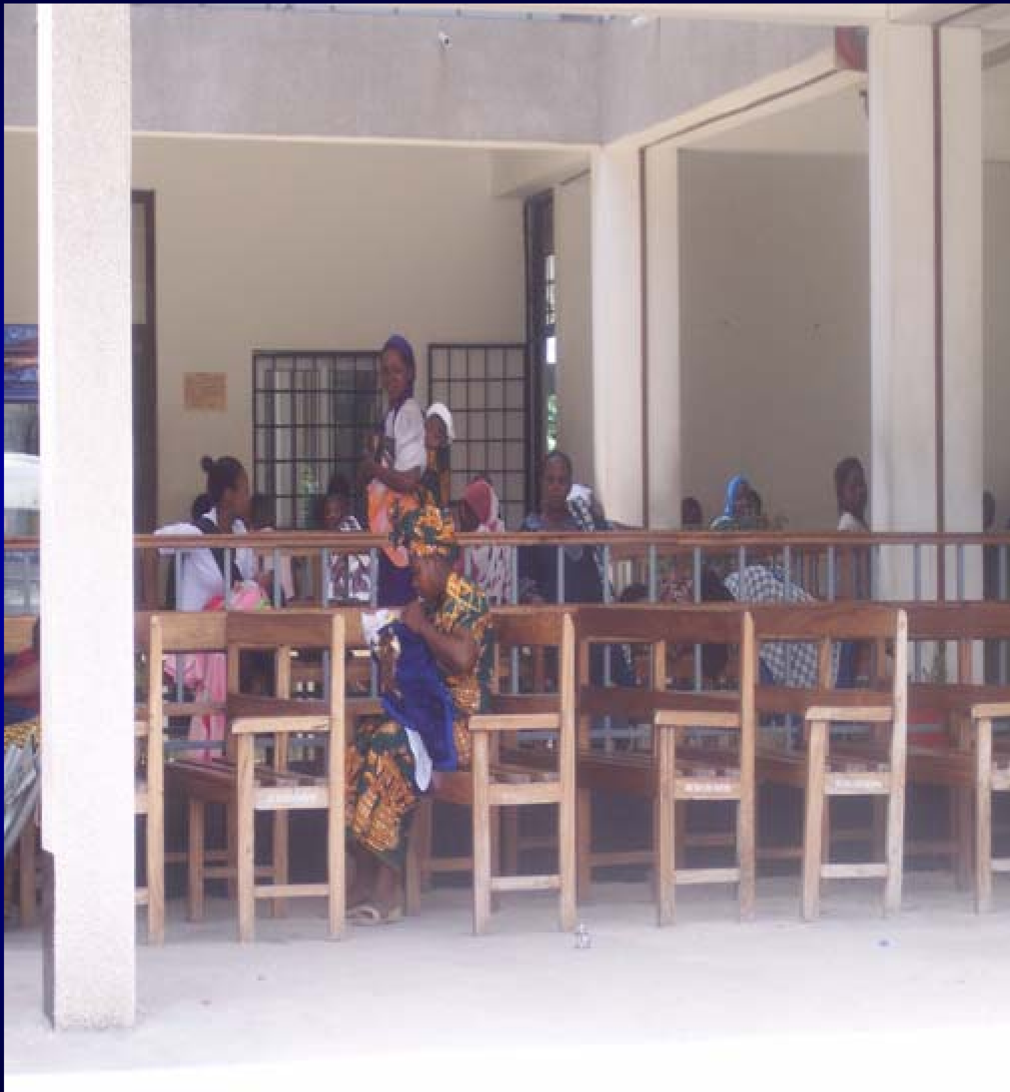

HIV Rapid Tests: Advantages

- Increased access to testing
- Expanded numbers of testing sites
- "Same-visit" HIV test results
- Permits testing in non-lab settings
- No refrigerated storage required
- WHO Level 1 test complexity
- Non-lab staff can be trained to use the tests

HIV Rapid Tests: Disadvantages

- Monitoring quality of testing is difficult
- Testing may be seen as “deceptively easy”
- Designing training for non-lab testers is challenging
- Positioning rapid testing within national medical systems is challenging

Body Fluids Used for HIV Rapid Testing

- Serum
- Plasma
- Whole blood
- Oral fluids

Three Formats of HIV Rapid Tests

- Immunoconcentration (flow-through device)
- Immunochromatography (lateral flow)
- Particle agglutination

Immunoconcentration Principle

HIV-antibody links to bound HIV-antigens forming colored spots

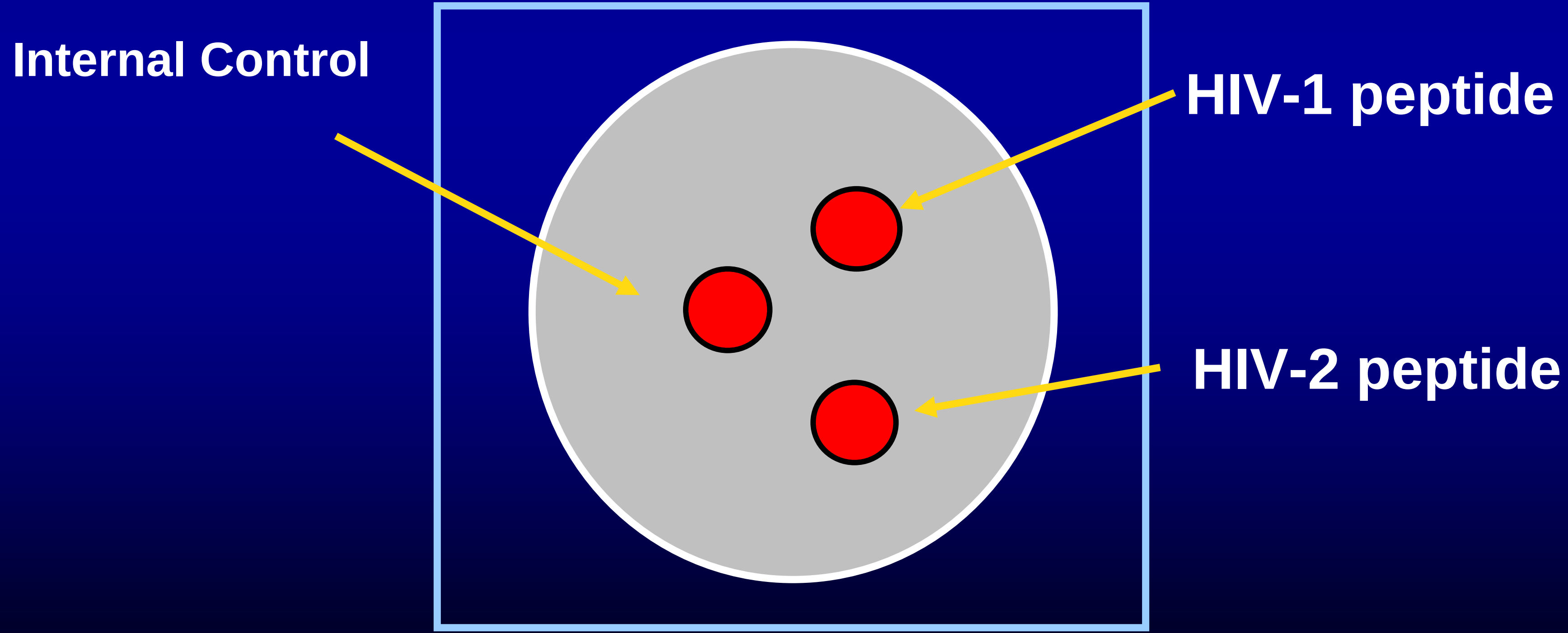

Immunoconcentration Tests

These devices are also called “Flow-Through” Devices.

Two brand names are:

- Multi-Spot
- Genie II

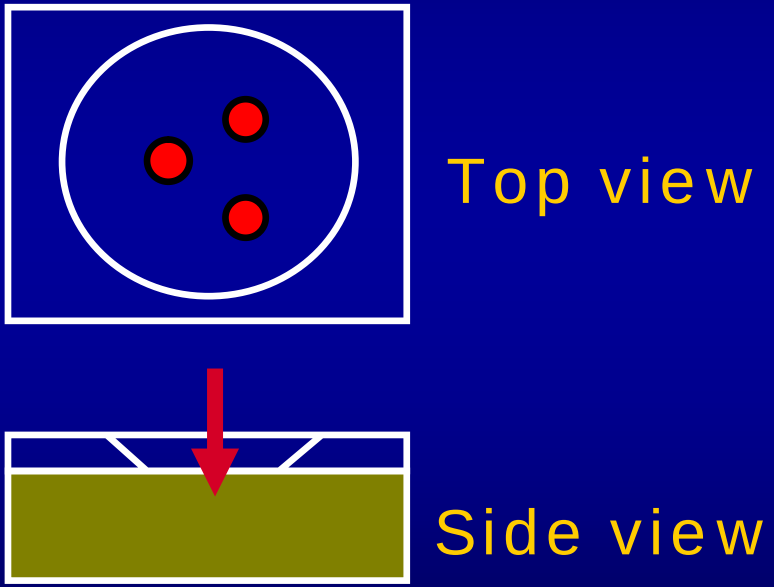

Reading Results: Genie II

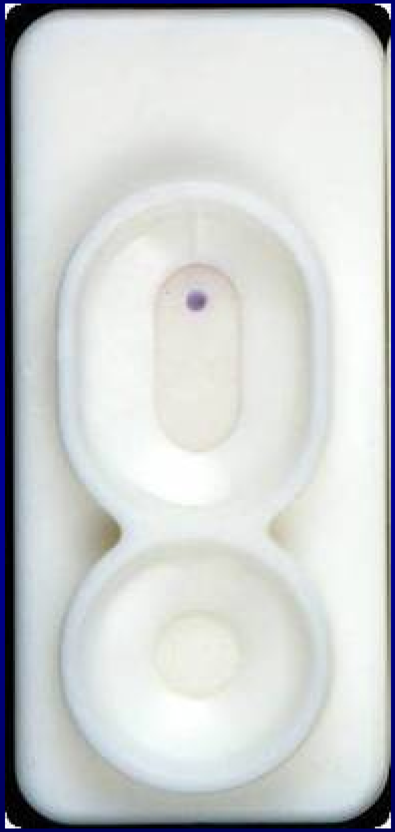

Negative

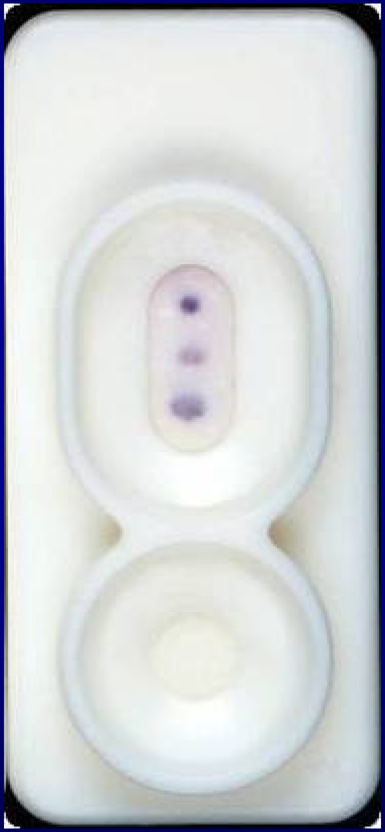

Positive

Immunochromatography Principle

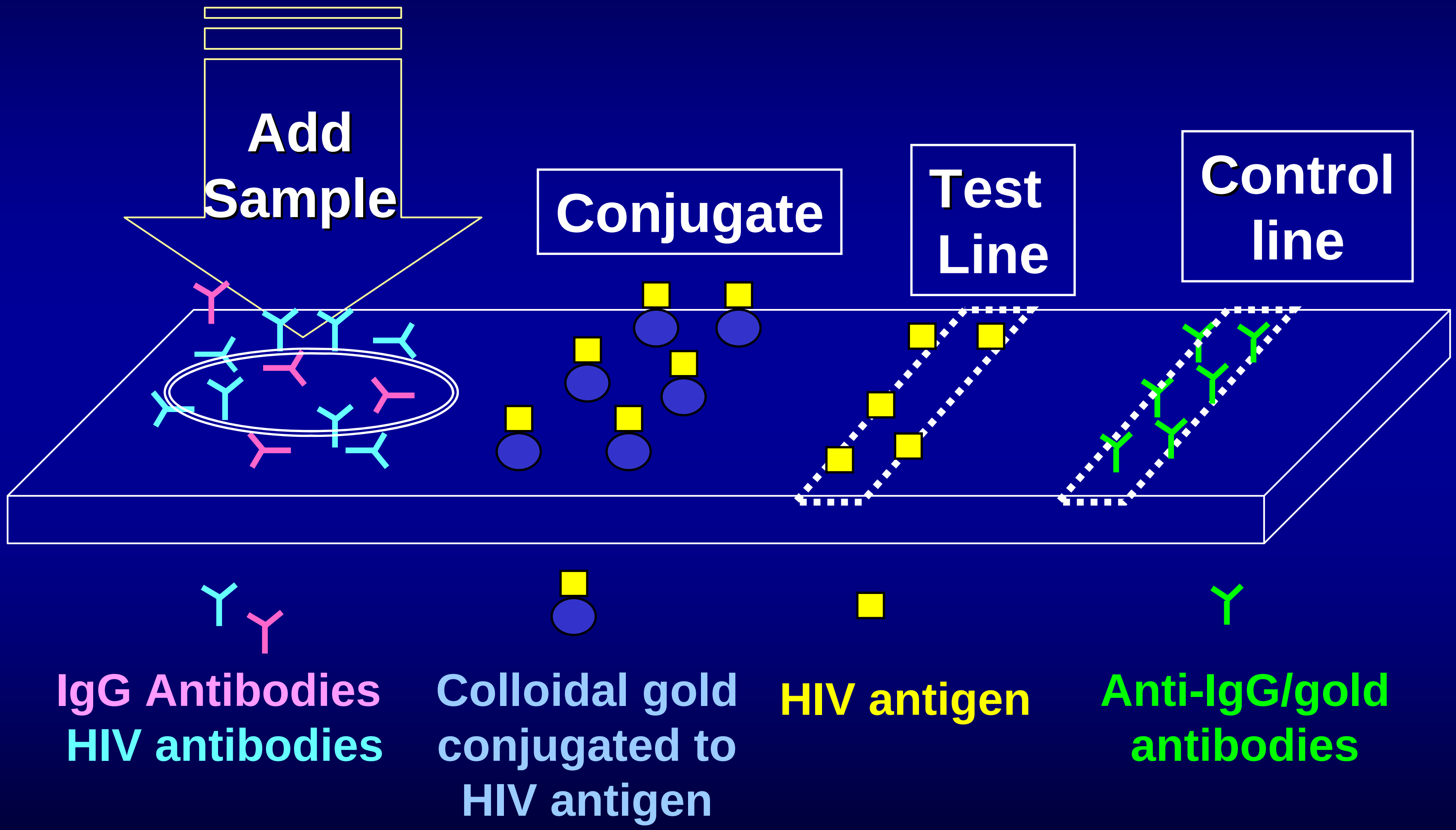

Lab workers   Health workers

Immunochromatography Tests

Lateral Flow Devices

- Determine
- Hema-Strip
- OraQuick
- Unigold
- Stat-Pak

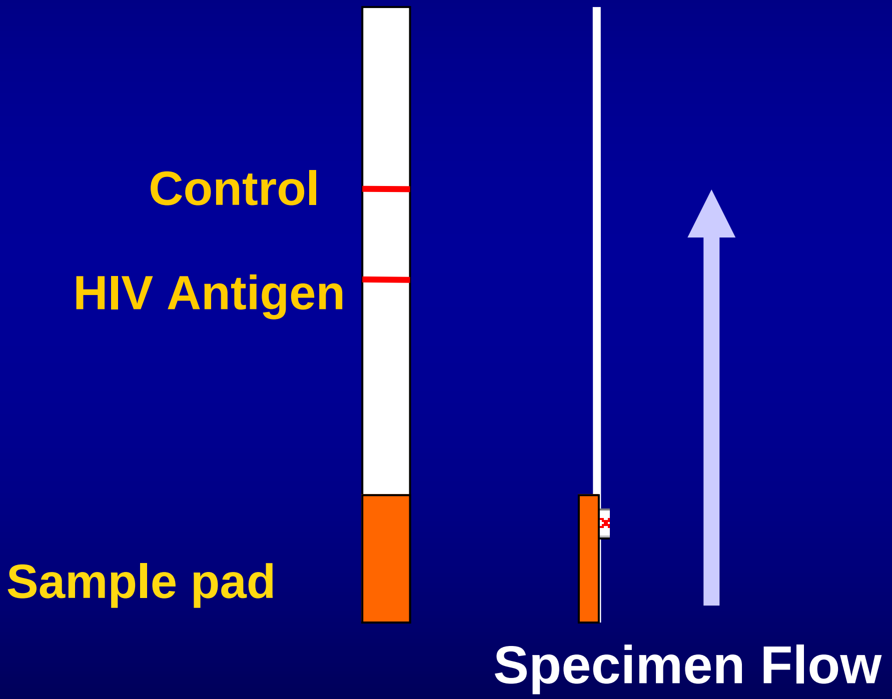

Reading Results: Determine

Negative

Positive

Positive

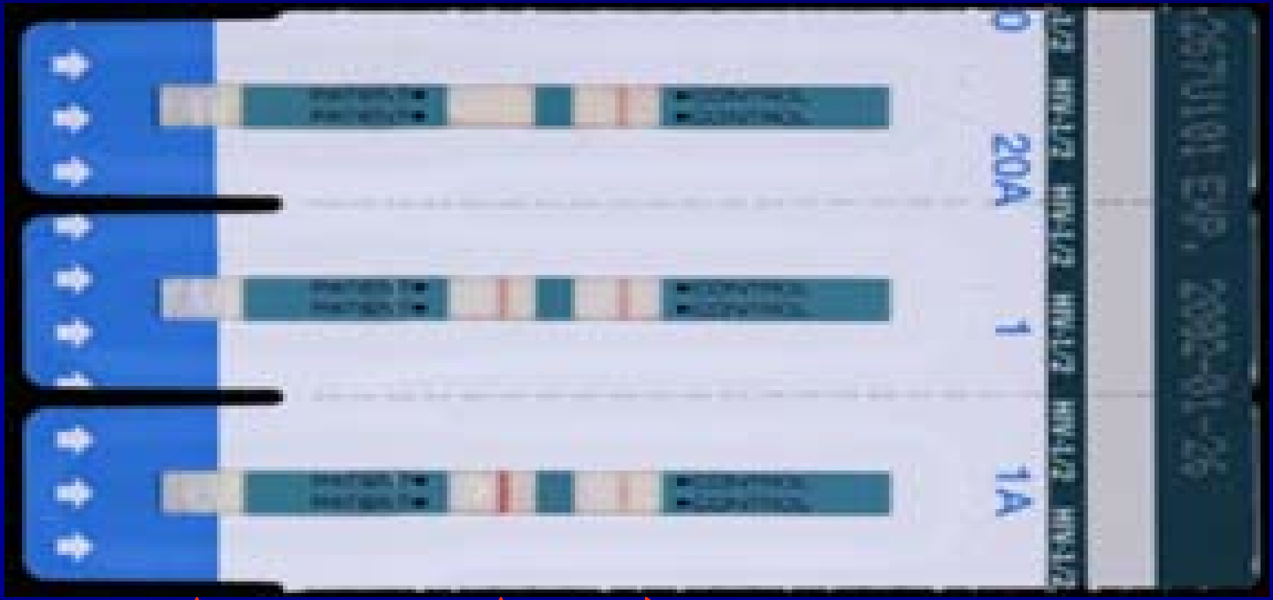

Sample Pad Test line Control line

Reading Results: OraQuick

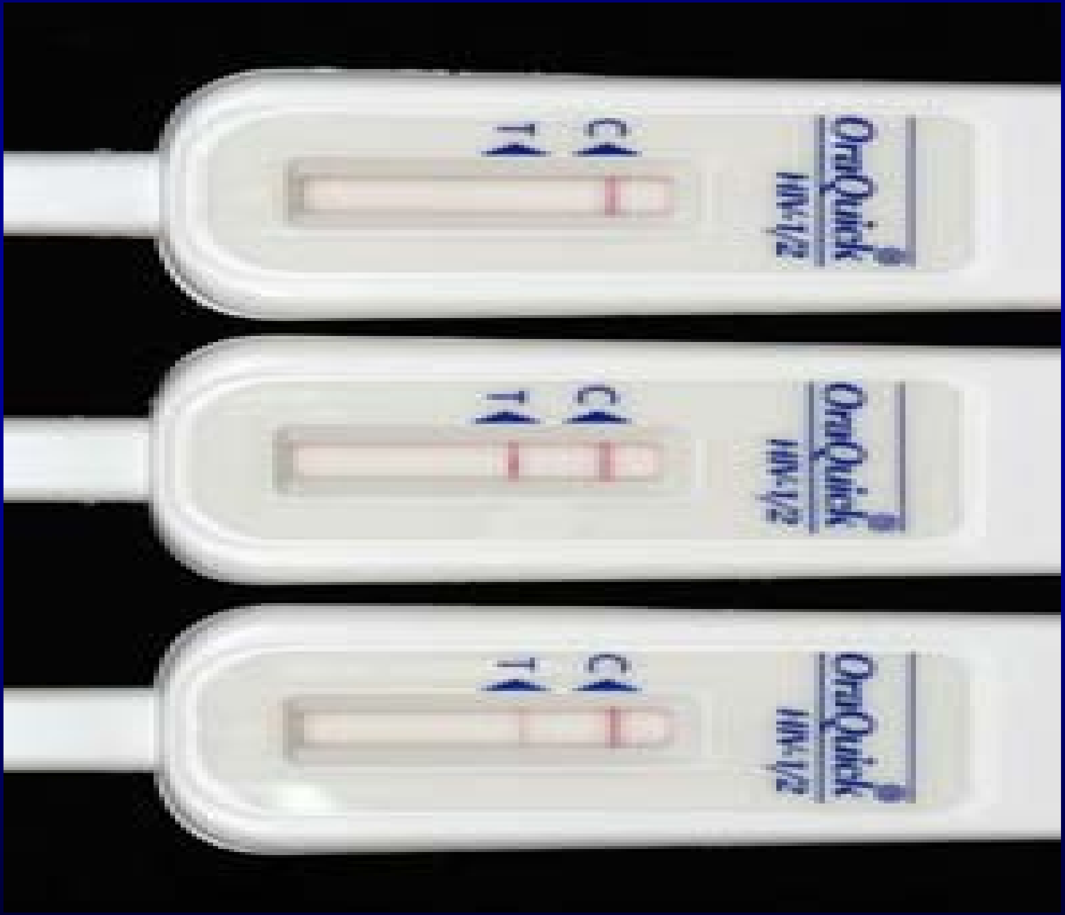

Negative

Positive

Positive

Particle Agglutination Principle

Anti-HIV antibodies bind to the antigen-coated latex particles.

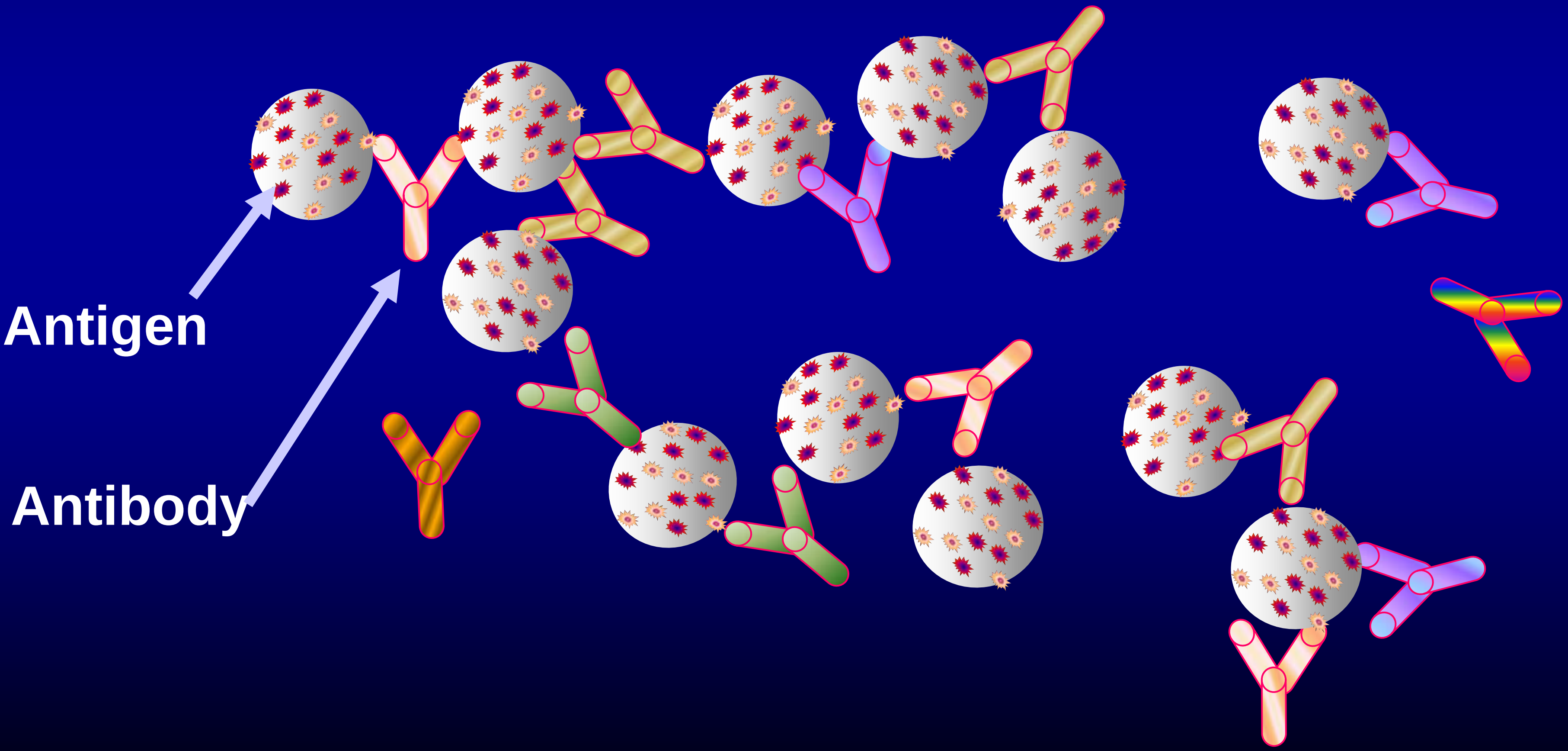

Agglutination Tests

- Capillus
- Serodia

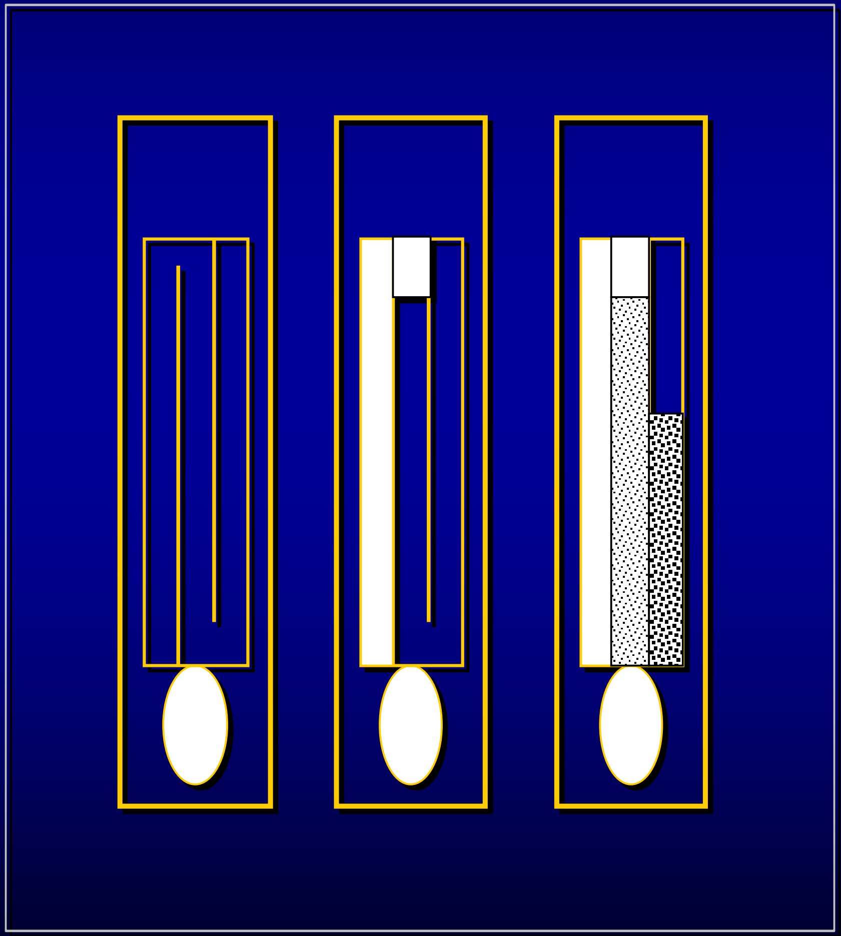

Reading Results: Capillus

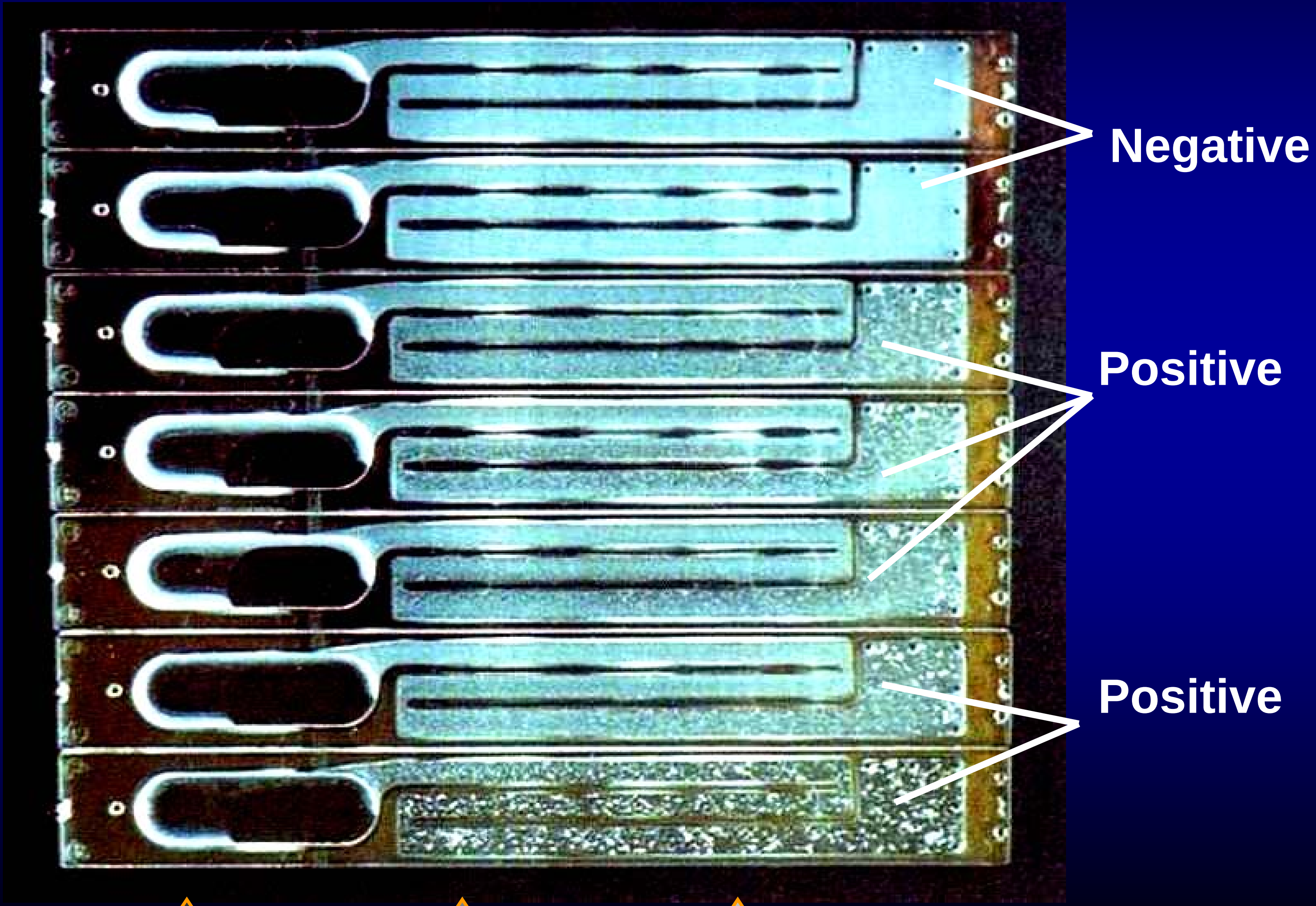

Results for HIV Rapid Tests

Positive

- Test band
- Control band

Negative

- Control band only

Invalid

- No control band present
- Repeat with new device.

Exercise: Interpreting Individual HIV Rapid Test Results

- *Refer to Participant Manual*
- *Work alone to determine individual test results*
- *3 Minutes*

Key Messages

- HIV rapid tests are reliable
- HIV rapid tests require training, supervision, and monitoring
- No single test result can be used to diagnose HIV infection

Summary

- What are rapid tests?
- List three advantages of HIV rapid testing.
- What is WHO Level 1 test complexity?
- Why are HIV viral load tests needed?
- How many types of HIV rapid test results are possible?

Module 4:
HIV Testing Algorithms

Learning Objectives

At the end of this module, you will be able to:

- Define parallel and serial testing algorithms
- Define national testing algorithms
- Explain test selection for the MOH TT "same-visit" HIV rapid testing algorithm
- Describe the MOH TT testing algorithm
- Define HIV status using an algorithm

Content Overview

- Testing strategies
- Testing algorithms
- National algorithms
- MOH TT national testing algorithm
- Interpreting HIV test results

Strategies and Algorithms

- Strategy – Testing approach used:
  - Blood Safety
  - Surveillance
  - Diagnosis

- Algorithm – combination and sequence of tests used in a given strategy

HIV Test Performance

- No test is 100 % sensitive
- No test is 100 % specific
- No single HIV test result is sufficient to diagnose HIV infection
- A combination of results (an algorithm) is essential to diagnose HIV infection

Diagnostic Algorithms

- All diagnostic HIV testing is based on at least 2 test results*
- When the first two tests are concordant, the result can be reported
- When the first two tests are discordant, a third test must be performed
- The result of the third test is the algorithm result

*WHO recommendations

Ideal Algorithm Tests are:

- Highly sensitive
- Highly specific
- Based on different antigens
- Routinely available
- Easy to use
- Stored at room temperature

National Testing Algorithms

Advantages:

- Country-wide standardization
- Simplified procurement
- Simplified supply management
- Uniform training
- Quality monitoring

National Algorithm Development

- Select type of algorithm needed*
- Review available information
- Select tests for inclusion
- Develop protocol
- Pilot test selected algorithm
- Expand to national scale
- Review testing algorithms annually

*WHO recommendations

MOH TT Algorithm Selection*

- Algorithm should provide “same-visit” HIV test results
- Algorithm should be suitable for use in “non-lab” locations
- “Same-visit” test results can be obtained using “diagnostic” algorithms*
- Diagnostic algorithms require three tests
- Non-lab locations require use of HIV rapid tests

*WHO recommendations

MOH TT Algorithm Test Selection

- WHO evaluation
- CAREC evaluation
- Guyana evaluation
- Brazil evaluation
- Temperature for shipment/storage
- Ease of use
- Type of specimen (whole blood)
- Cost
- Procurement reliability

The Algorithm Is Important

Always follow the algorithm!

HIV Diagnostic Algorithms*

- Serial algorithms are suitable where prevalence of HIV exceeds 10%
- Parallel algorithms are needed where prevalence of HIV is less than 10%
- In TT, HIV prevalence is less than 10% and diagnosis requires a parallel algorithm

*WHO definitions

Serial Testing Algorithm

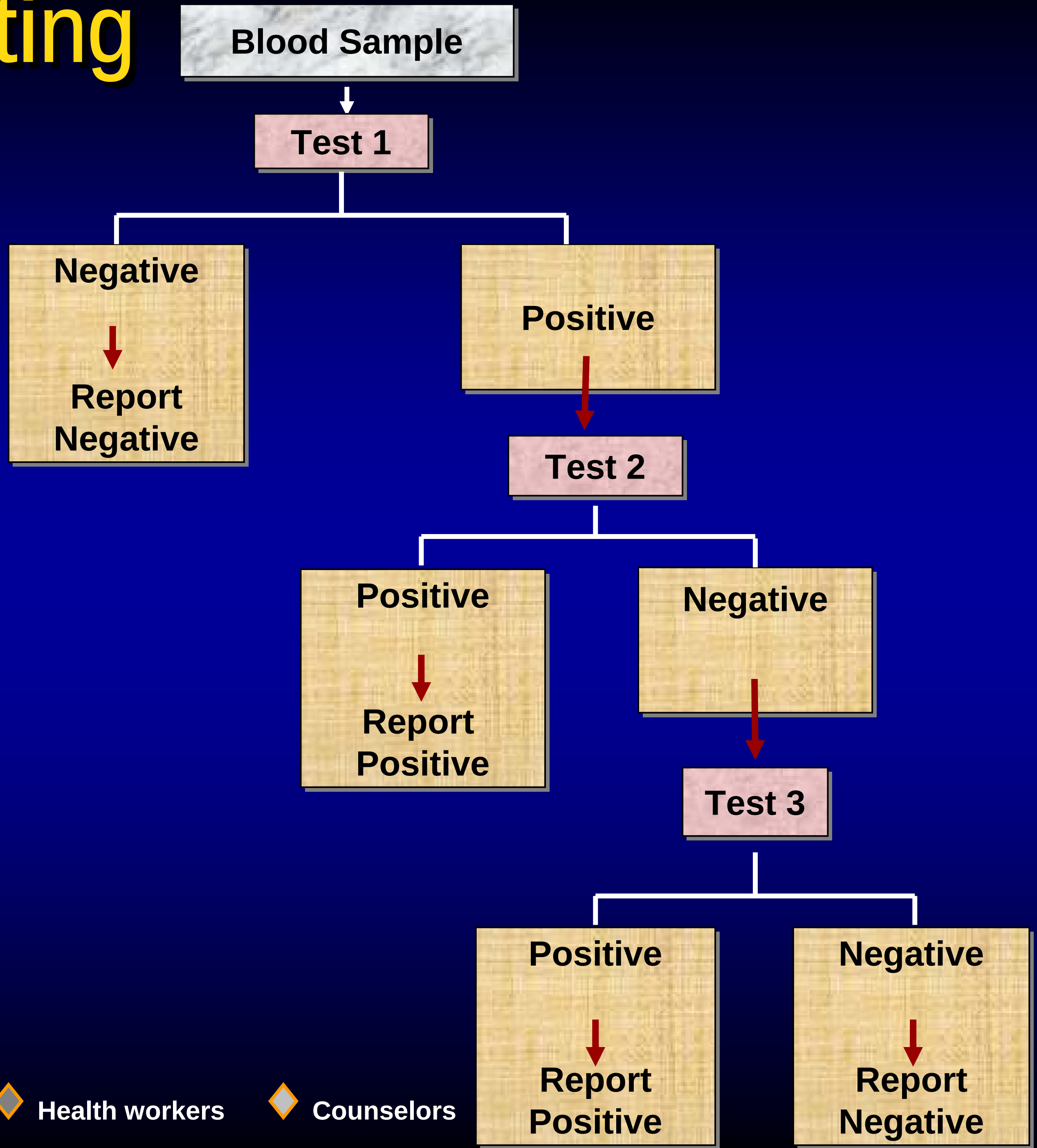

- Lab workers
- Health workers
- Counselors

Parallel Testing Algorithm

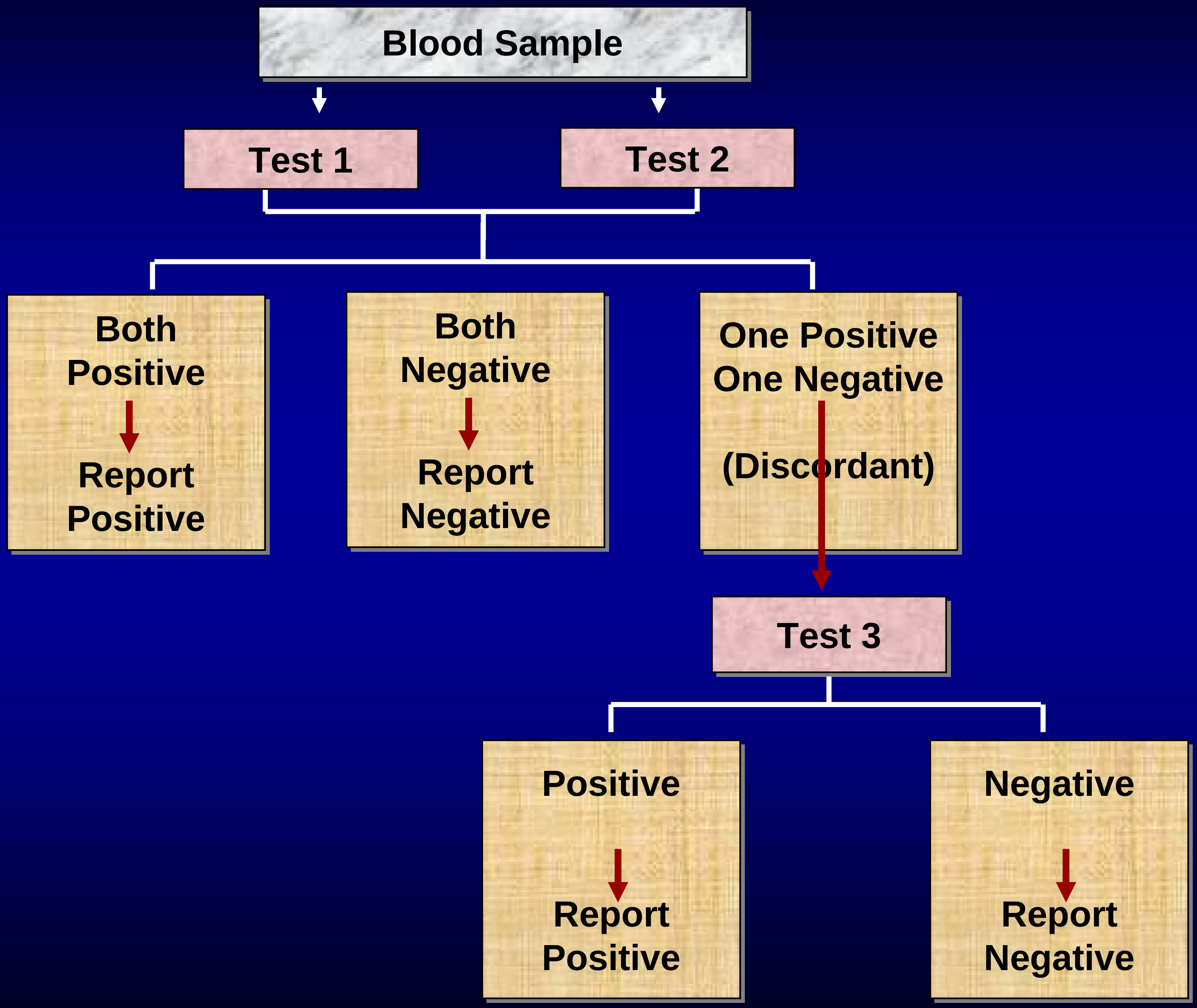

Lab workers Health workers Counselors

MOH TT Algorithm

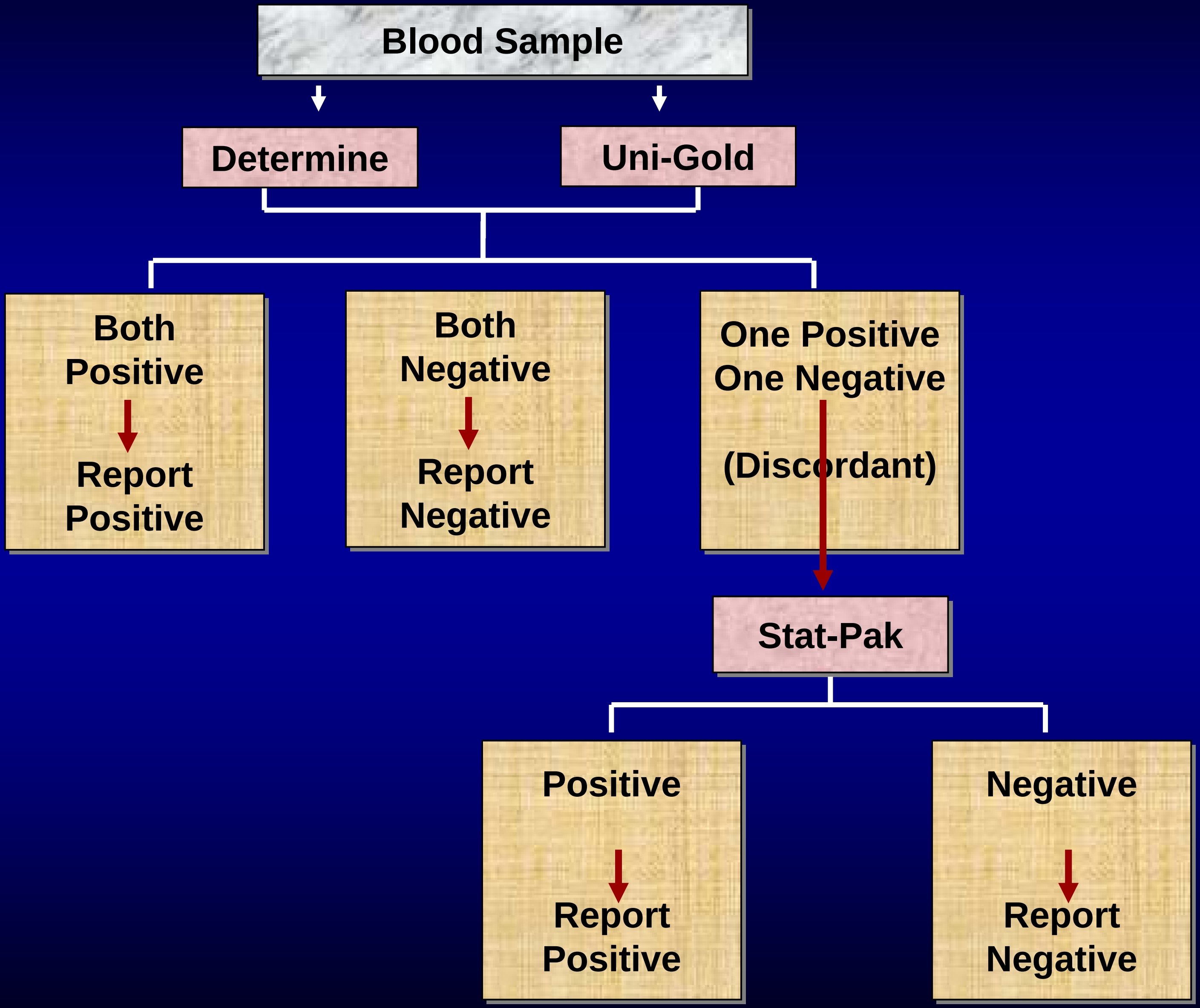

Lab workers Health workers Counselors

HIV Testing Results: Parallel Algorithm

TEST 1	TEST 2	TEST 3	HIV Status
Negative	Negative		

HIV Testing Results: Parallel Algorithm

TEST 1	TEST 2	TEST 3	HIV Status
Negative	Negative		Negative

HIV Testing Results: Parallel Algorithm

TEST 1	TEST 2	TEST 3	HIV Status
Positive	Positive		

Lab workers   Health workers   Counselors

HIV Testing Results: Parallel Algorithm

TEST 1	TEST 2	TEST 3	HIV Status
Positive	Positive		Positive

Lab workers Health workers Counselors

HIV Testing Results: Parallel Algorithm

TEST 1	TEST 2	TEST 3	HIV Status
Negative	Positive	Negative	

HIV Testing Results: Parallel Algorithm

TEST 1	TEST 2	TEST 3	HIV Status
Negative	Positive	Negative	Negative

HIV Testing Results: Parallel Algorithm

TEST 1	TEST 2	TEST 3	HIV Status
Positive	Negative	Positive	

Lab workers Health workers Counselors

HIV Testing Results: Parallel Algorithm

TEST 1	TEST 2	TEST 3	HIV Status
Positive	Negative	Positive	Positive

Lab workers   Health workers   Counselors

HIV Testing Results:
Parallel Algorithm

TEST 1	TEST 2	TEST 3	HIV Status
Negative	Positive	Positive	

HIV Testing Results: Parallel Algorithm

TEST 1	TEST 2	TEST 3	HIV Status
Negative	Positive	Positive	Positive

Questions?

- When can HIV be diagnosed with a single test?
- What is an algorithm?
- Why is the MOH TT algorithm parallel?
- What does it mean when test results are concordant? Discordant?
- What tests are in the MOH TT algorithm?

Module 5: Quality of HIV Rapid Testing

A Systems Approach to Quality

Learning Objectives

At the end of this module, you will be able to:

- Explain the systems approach to quality
- Identify the elements of quality in HIV rapid testing
- Recognize factors that compromise quality
- Describe your role in assuring quality in HIV testing

Content Overview

- How to achieve quality
- How to maintain quality
- Essential elements of a quality system
- Quality assurance in HIV rapid testing
- Your role in quality of HIV rapid testing

What Is "Quality"?

What Is “Quality”?

- Reliable
- Reproducible
- Accurate
- Provides satisfaction
- Meets expectations

A Systems Approach to Quality

- Considers all components within a system
- Identifies the connections and relationships among the components

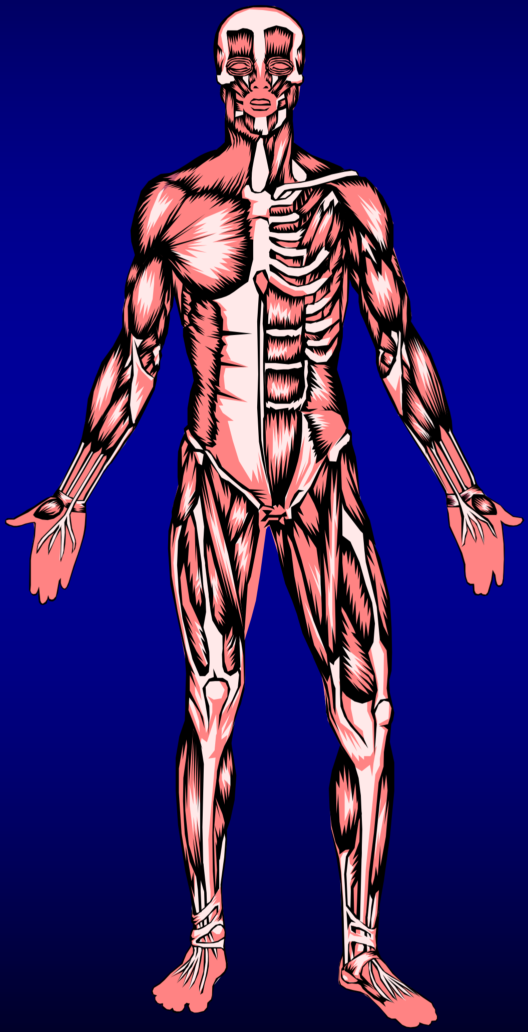

Example: the human body system

A pain in one component may be caused by a disorder in another component

Benefits of Quality Systems

- Monitors all components of testing
- Detects and corrects errors
- Improves reproducibility between testing sites
- Helps contain costs

T&T Quality Health Care System

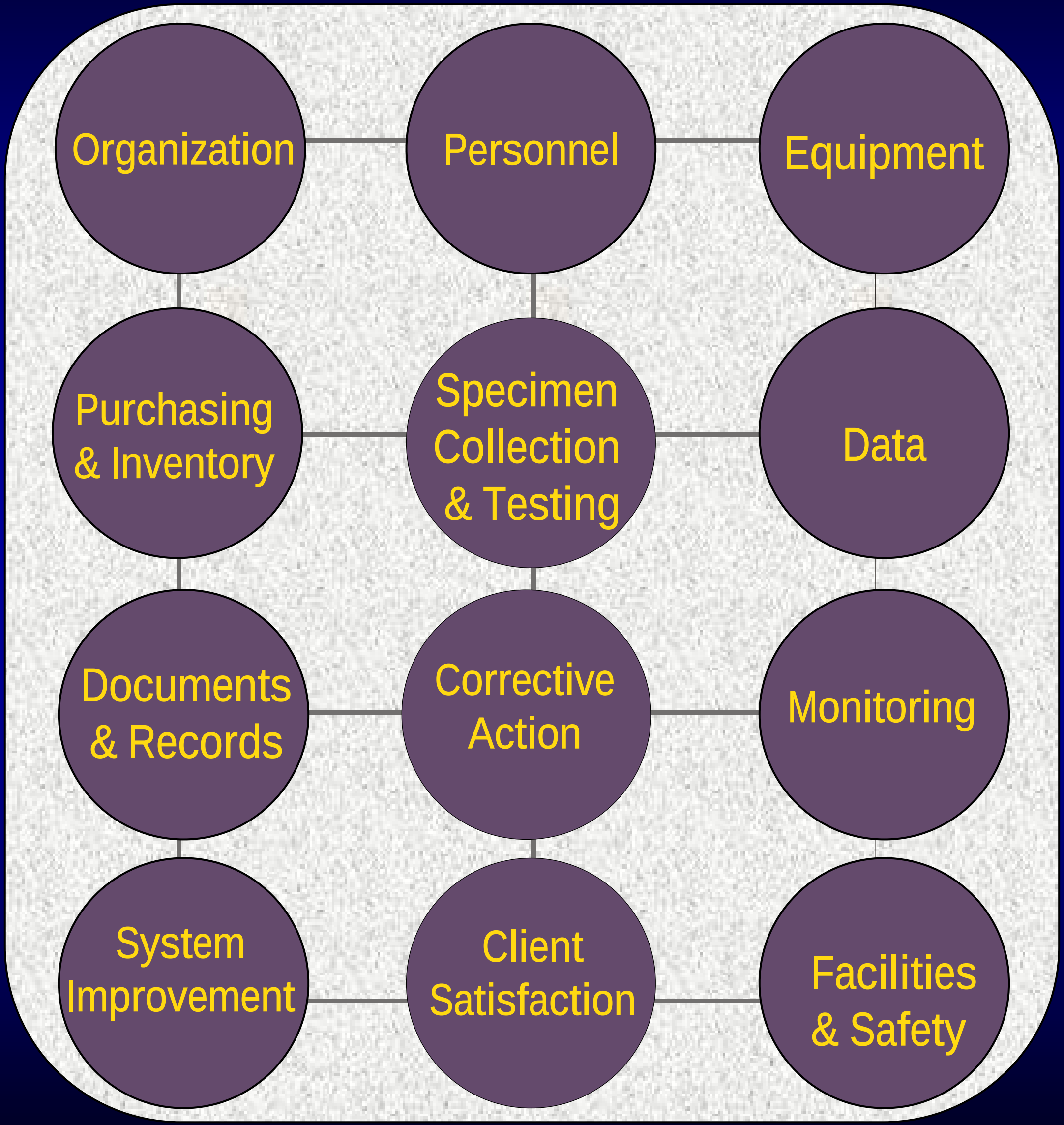

Lab workers Health workers Counselors

Organization

Ministry of Health

Regional Health Authorities

Non-Governmental Organizations

Policy

Budget

Resources

Personnel

Planning

Hiring

Retention

Training

Supervision

Performance

Equipment

Selection

Maintenance

Security

Purchasing and Inventory

- Ordering
- Receiving
- Storing
- Tracking
- Records

Spec Collection & Testing

Specimen Management

Standard Procedures

Training

Quality Control

Result Reporting

Lab workers   Health workers   Counselors

Data

Collection

Management

Client Confidentiality

Computer skills

Lab workers   Health workers   Counselors

Documents and Records

Standardized Forms

Document Approval

Document Distribution

Document Storage

Document Destruction

Corrective Action

Error Identification

Troubleshooting

Improvement

Record Keeping

Monitoring

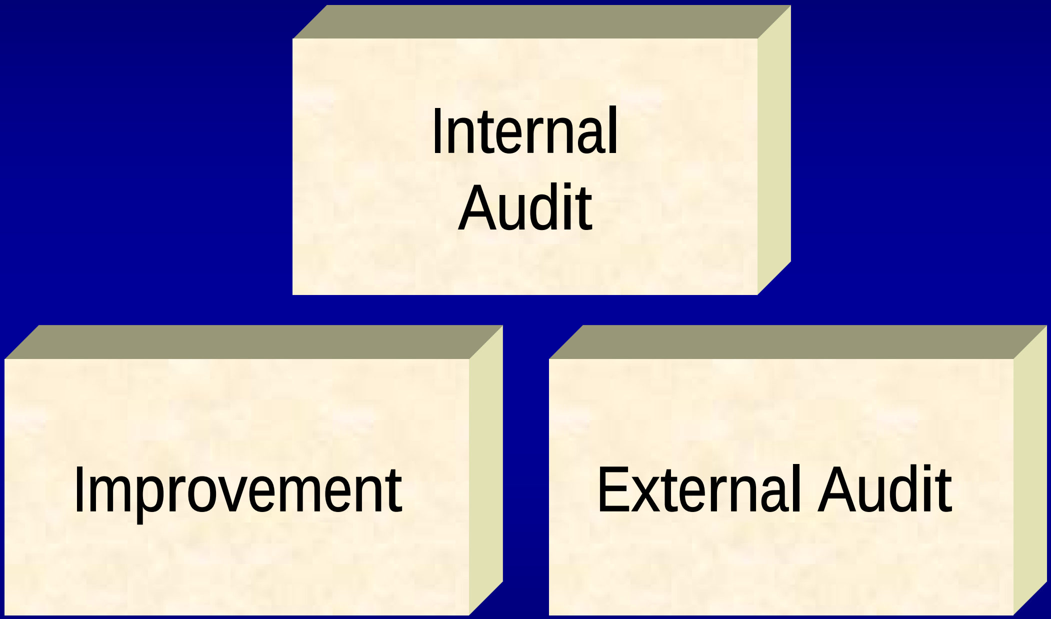

System Improvement

Data Collection

Data Review

Data Utilization

Client Satisfaction

Feedback

Troubleshooting

Resolution

Facilities & Safety

Universal Precautions

Safety Policy

Confidentiality

Standard Procedures

Records

Quality Testing Systems

Quality system components include all activities, supplies, and personnel that contribute to test results.

Quality HIV Testing System

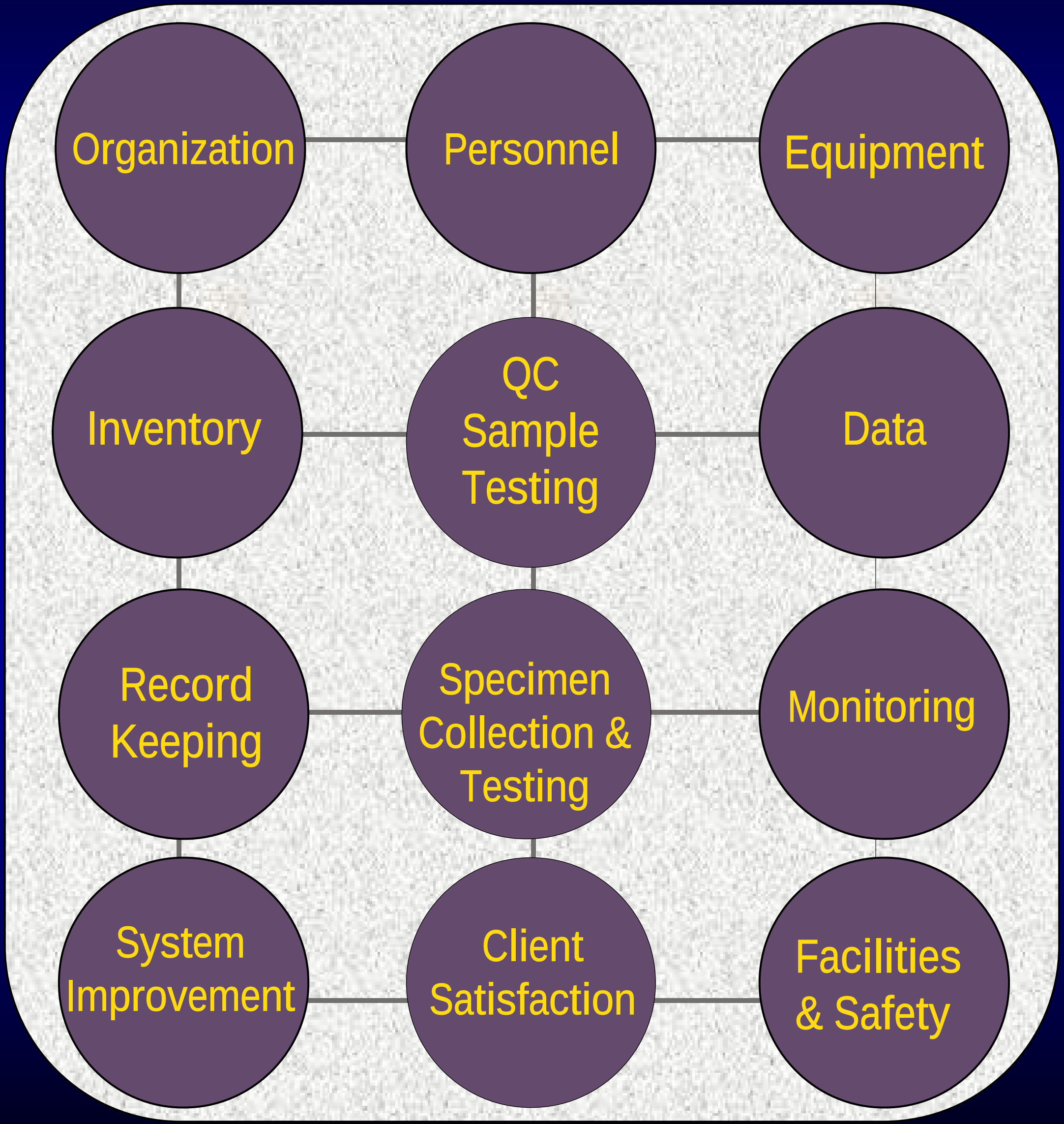

Lab workers Health workers Counselors

Activity: Are You “Positive” or “Negative?”

Participants take turns tossing the cabbage ball. When you catch the ball,

- Peel a statement off the ball
- Read out loud your statement to the group
- Based on the statement, go to:
  - The Positive Circle or
  - The Negative Circle

Who Is Responsible for Quality?

EVERYONE!

- Laboratory and program staff establish quality procedures.
- Test site personnel implement quality procedures.

Why Do Errors Occur?

- Responsibilities unclear
- Written procedures lacking
- Written procedures not followed
- Training incomplete
- Records improperly monitored
- Supplies stored improperly
- QC samples not tested

Preventing and Detecting Errors

- Record temperatures
- Review inventory
- Test QC samples
- Maintain QC Sample Log
- Use designated testing space
- Follow SOPs
- Complete records for each client

Testing Errors

- Algorithm not followed
- Individual test SOPs not followed
- Incorrect time for reading test
- Wrong buffer used for testing
- Expired reagents used for testing
- Finger-stick fails to give enough blood
- Incorrect information recorded
- Improper waste disposal

Quality in HIV Rapid Testing

- Makes test results uniform among testing sites
- Sets a standard for HIV testing
- Meets/exceeds customer expectations
- Provides approach to correcting problems
- Provides data for improvement of testing
- Reduces costs

No test is foolproof!

Summary

- Why do errors occur?
- What errors could occur in HIV rapid testing?
- Where is quality in a testing site?
- How can you reduce errors in testing?
- How will errors impact clients?

Module 6: Safety at the HIV Rapid Testing Site

Learning to “Think like a lab person” (Part 1)

Quality HIV Testing System

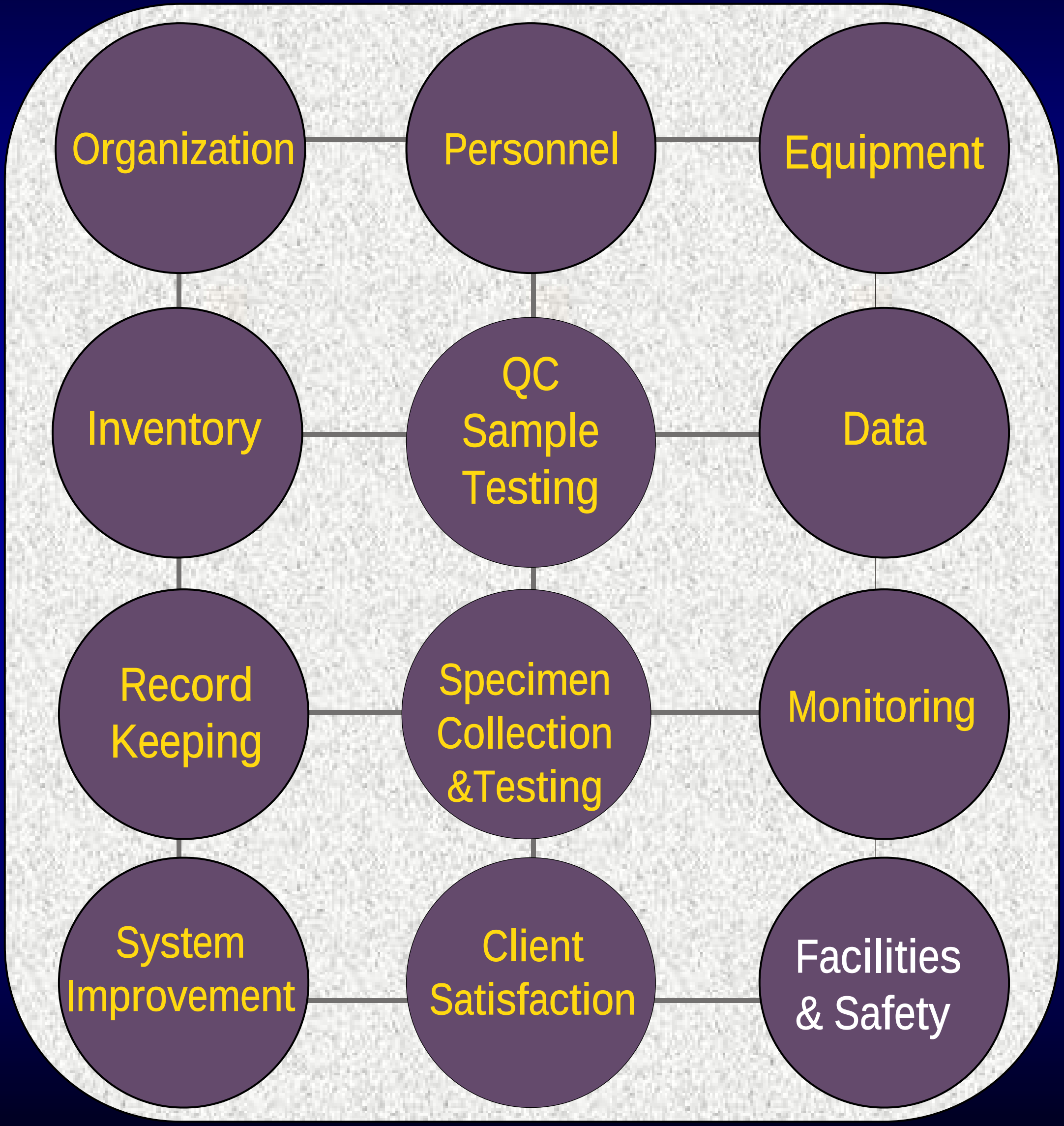

Lab workers Health workers Counselors

Learning Objectives

At the end of this module, you will be able to:

- Follow personal health and safety practices
- Maintain a clean and organized workspace
- Dispose of infectious materials
- Describe what to do in the case of an accident
- Follow safety procedures and keep safety records

Content Overview

- Safety practices include how to:
  - Develop safety habits
  - Properly dispose of waste
  - Keep safety records

What is Safety?

Safety is taking precautions to protect you, the client and others against infection, accident or injury.

Why Is Safety Important?

We cannot wait until a serious accident or illness occurs before we implement safe practices

Who Needs to be safe?

- The client
- The tester
- Others

Universal Safety Precautions

Every specimen should be treated as though it is infectious

Practice Personal Safe Work Habits

- Wash hands before and after testing each client
- Change gloves for each client
- Wear lab coat or apron
- Dispose of lancets in designated container
- Do not retrieve anything out of the disposal container/bag

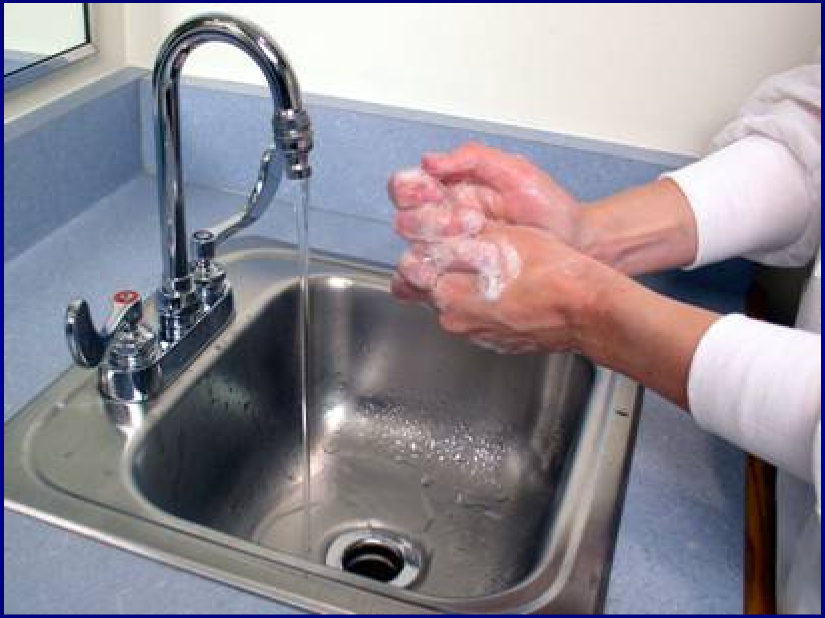

Practice Personal Safe Work Habits (Cont'd)

- Do not put pens/pencils in your mouth. It is *strictly forbidden*
- Never eat, drink ,apply make-up, remove or insert contact lenses, or smoke at the test site
- Do not store food/drink in the QC sample refrigerator

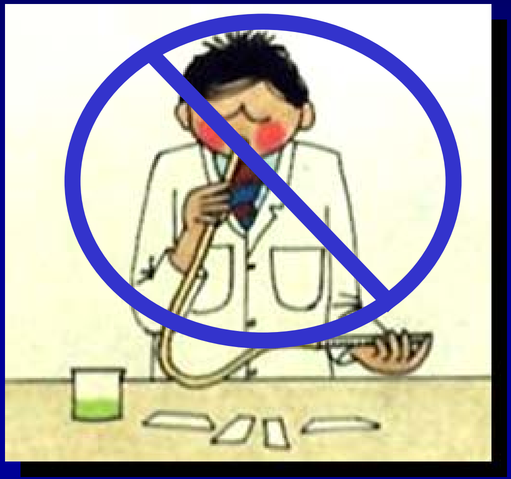

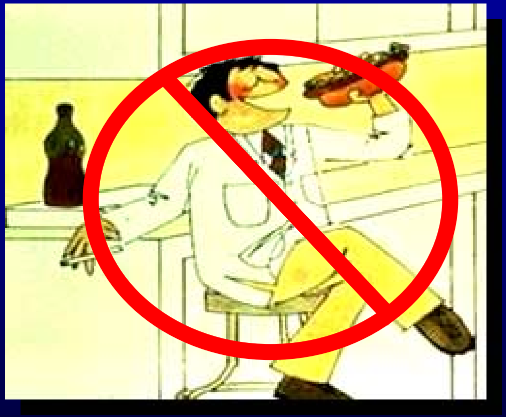

What is a Bio-Hazard?

An organism or substance capable of causing infection, disease or death.

Maintain Clean & Orderly Work Space

- Keep work areas uncluttered and clean
- Restrict or limit access when working
- Keep supplies locked in a safe and secure area

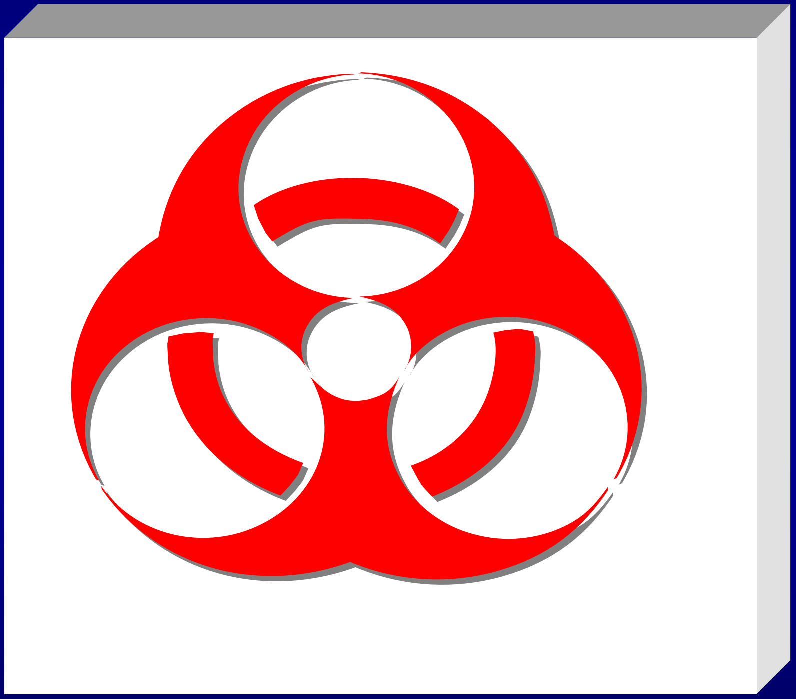

Biohazard

Drop Used Lancets in Special Containers

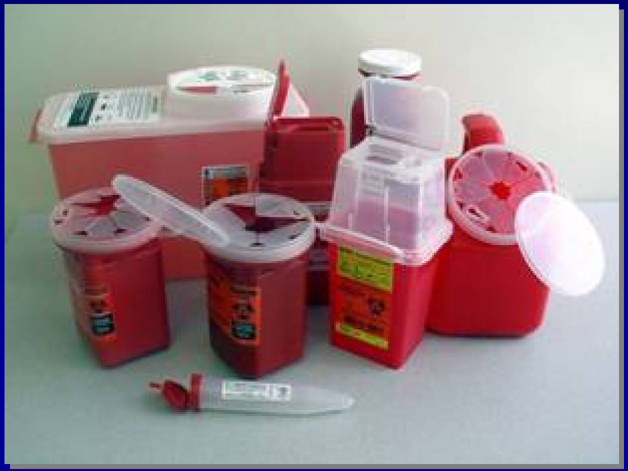

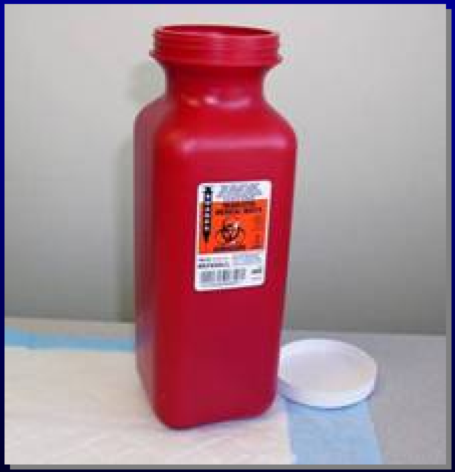

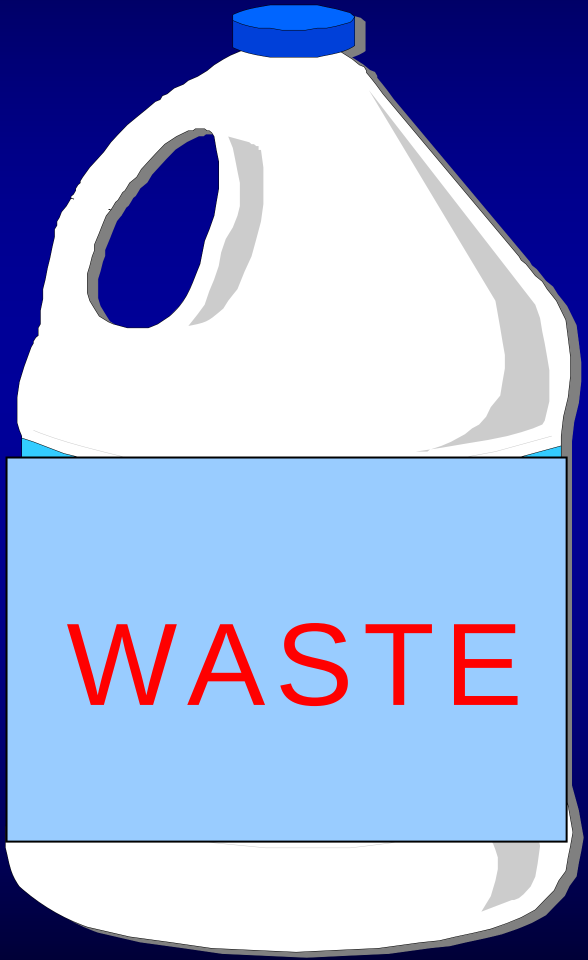

Disinfect Work Areas with Bleach

Disinfection

- Kills germs and pathogens
- Keeps work surface clean
- Prevents cross-contamination
- Reduces risks of infection

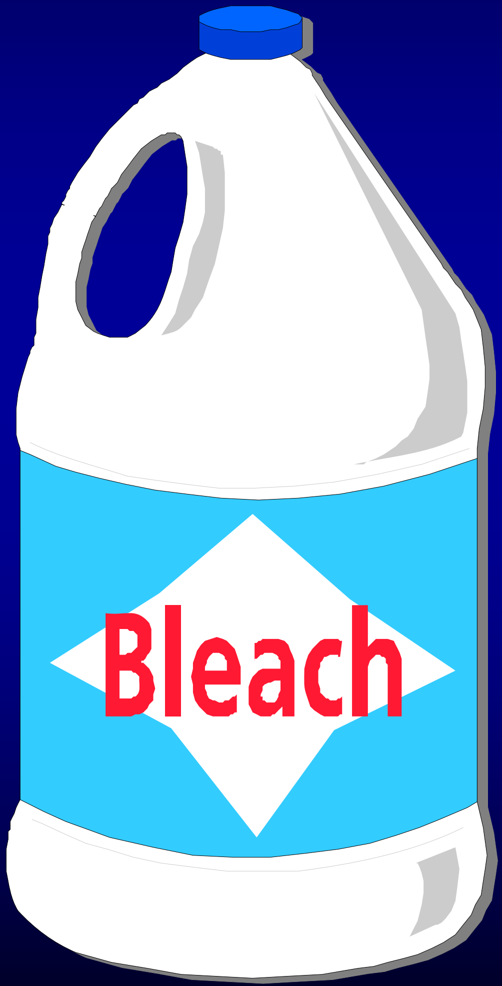

Different Cleaning Jobs Require Different Bleach Solutions*

General lab use - *Hypochlorite Solutions*

Spills	General Disinfection
**10%**<br>**(1 part + 9 parts)**	**1%**<br>**(1 part + 99 parts)**

You should have 10% bleach readily available at your test site.

* WHO Laboratory Biosafety Manual

Making a 10% Bleach Solution

Referred to as a 1/10, 1:10

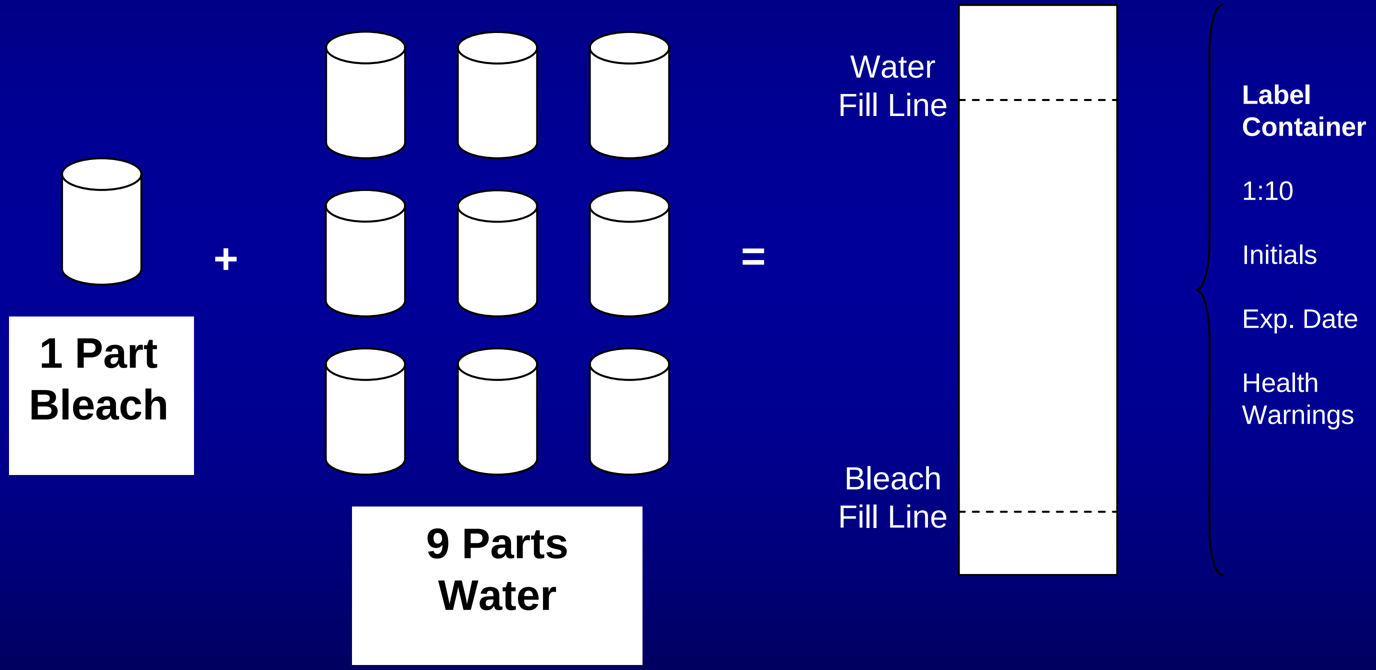

In Case of a Spill or Splash

- Wear clean disposable gloves
- Large spills- Cover with paper towels and soak with 10% household bleach and allow to stand for at least 5 minutes
- Small spill - Wipe with paper towel soaked in 10% bleach
- Discard contaminated towels in infectious waste containers

Incineration of Waste

- Incineration is burning of contaminated waste to destroy and kill micro-organisms.
- Incineration:
  - Prevents re-use
  - Protects environment
  - Must be conducted by authorized institutions

In Case of an Accident

What should you do?

- Assess & take action
- Report to manager immediately
- Record on standard form

Action Plan for Implementing Safety Practices

- Identify hazards
- Establish safety policies
- Implement safety procedures
- Keep proper safety records

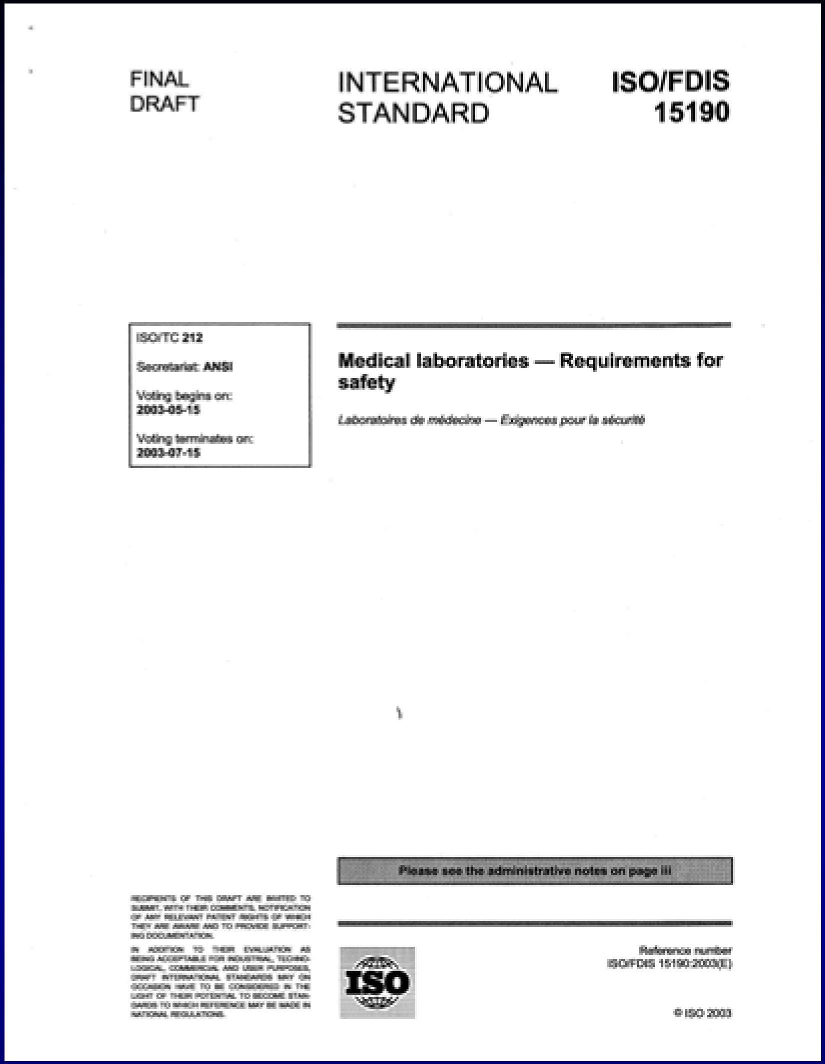

FINAL DRAFT

INTERNATIONAL STANDARD

ISO/FDIS 15190

ISO/TC **212**

Secretariat: **ANSI**

Voting begins on: **2003-05-15**

Voting terminates on: **2003-07-15**

Medical laboratories — Requirements for safety

Laboratoires de médecine — Exigences pour la sécurité

Please see the administrative notes on page iii

RECIPIENTS OF THIS DRAFT ARE INVITED TO SUBMIT, WITH THEIR COMMENTS, NOTIFICATION OF ANY RELEVANT PATENT RIGHTS OF WHICH THEY ARE AWARE AND TO PROVIDE SUPPORTING DOCUMENTATION.

IN ADDITION TO THEIR EVALUATION AS BEING ACCEPTABLE FOR INDUSTRIAL, TECHNOLOGICAL, COMMERCIAL AND USER PURPOSES, DRAFT INTERNATIONAL STANDARDS MAY ON OCCASION HAVE TO BE CONSIDERED IN THE LIGHT OF THEIR POTENTIAL TO BECOME STANDARDS TO WHICH REFERENCE MAY BE MADE IN NATIONAL REGULATIONS.

Reference number
ISO/FDIS 15190:2003(E)

© ISO 2003

Safety References

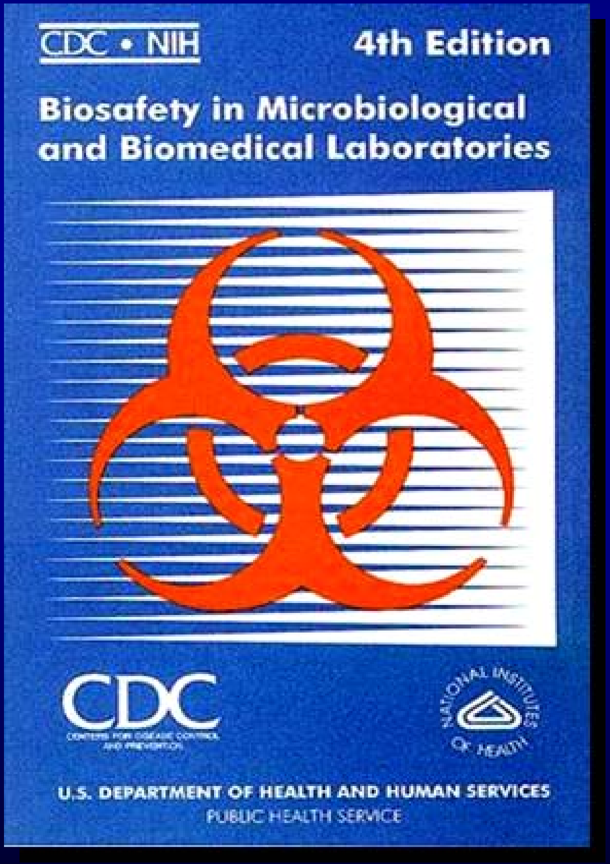

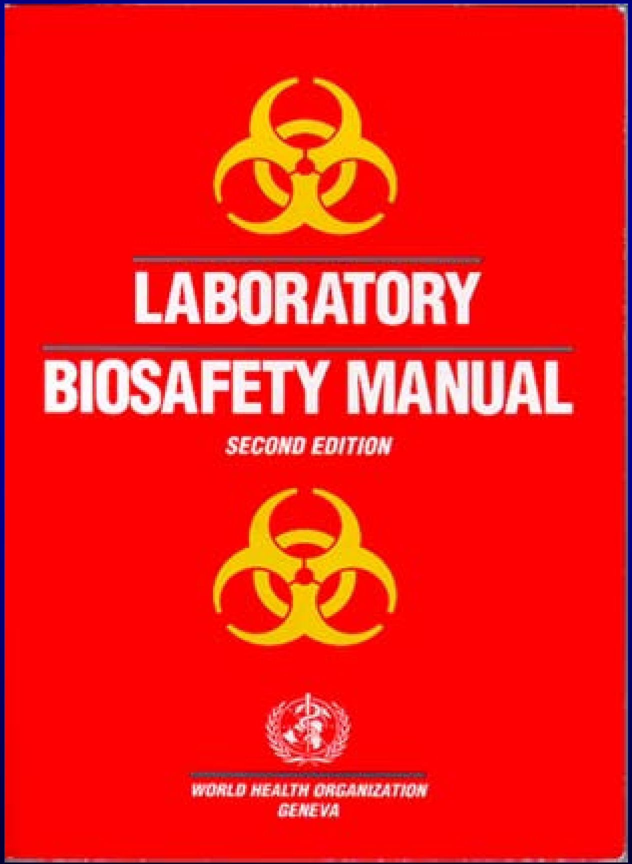

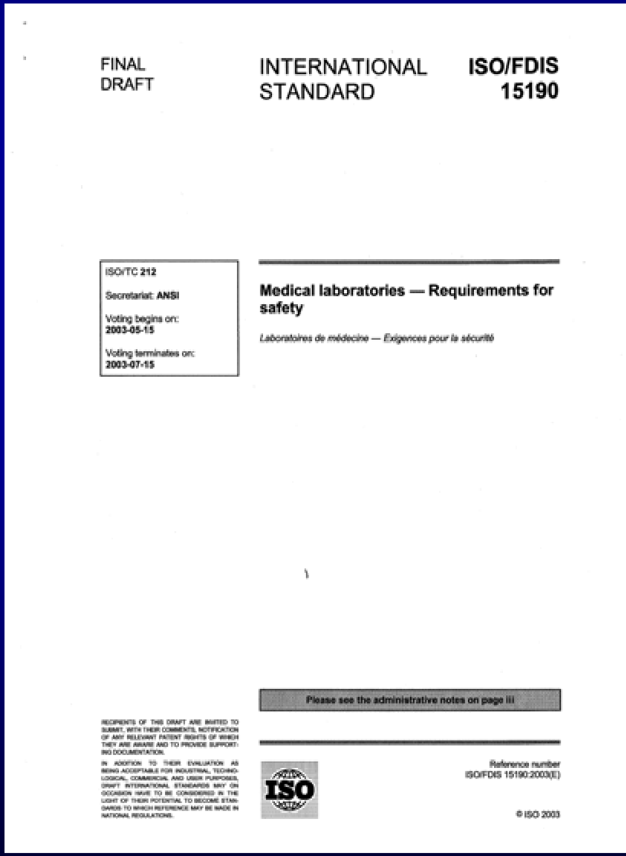

Lab workers Health workers Counselors

Module 7:
Preparing for Testing – Supplies and Kits

Learning Objectives

At the end of this module, you will be able to:

- List and identify all the supplies required for HIV rapid testing
- List and identify all the components of test kits for HIV rapid testing

Memory Game: Supplies & Materials

How To Play:

- Spend 3 minute browsing through the display and learn the items
- Spend 1 minute writing down as many items as possible (without looking)
  - Tally up the number of items you list
  - The person with the most items wins.

Materials & Supplies Checklist (Part 1)

- HIV rapid test kits
- Alcohol swabs
- Cotton
- Retractable Lancets
- Leak-proof bag for waste incineration
- Disinfectant
- Pen for writing
- Pen for labeling
- Timer or watch with minute hand
- Paper towels (for cleaning, hand washing and work area)
- Soap for hand washing

Materials & Supplies Checklist (Part 2)

- One positive QC sample
- One negative QC sample
- QC sample log
- Standard Operating Procedures
- Band Aids / plasters
- Thermometers
- Register for recording results

Gloves

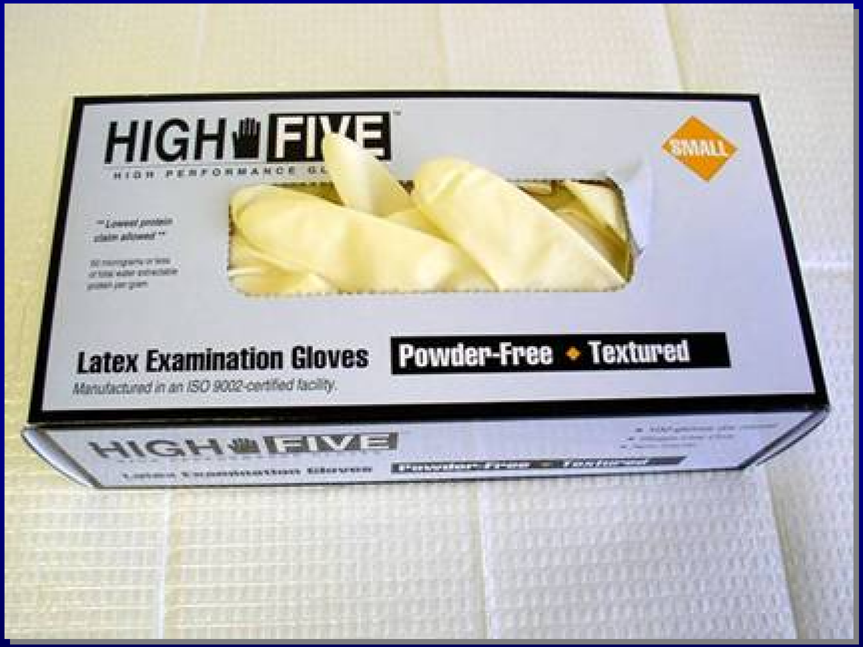

- Single use disposable gloves
- Latex or polypropylene
- Without evidence of holes or tearing

Alcohol

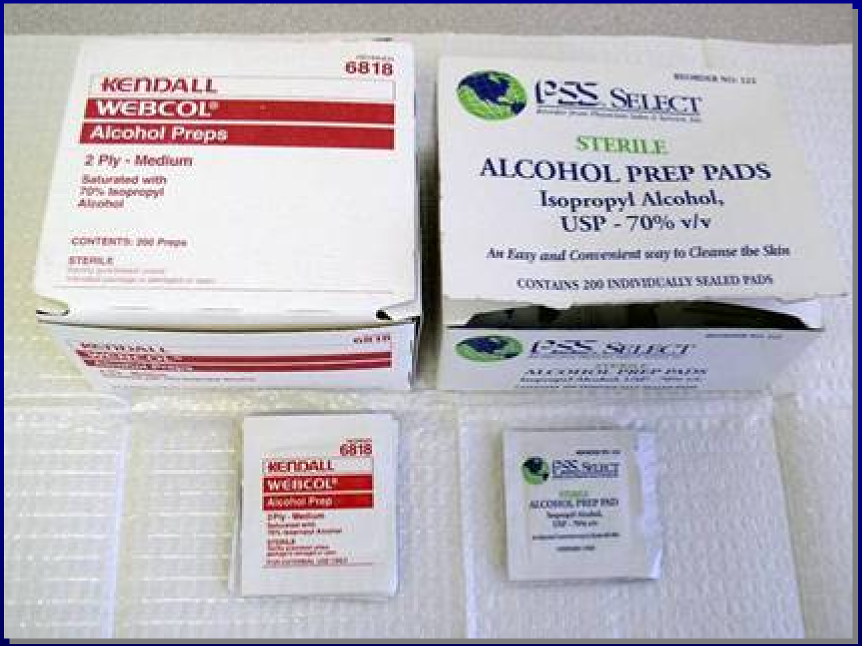

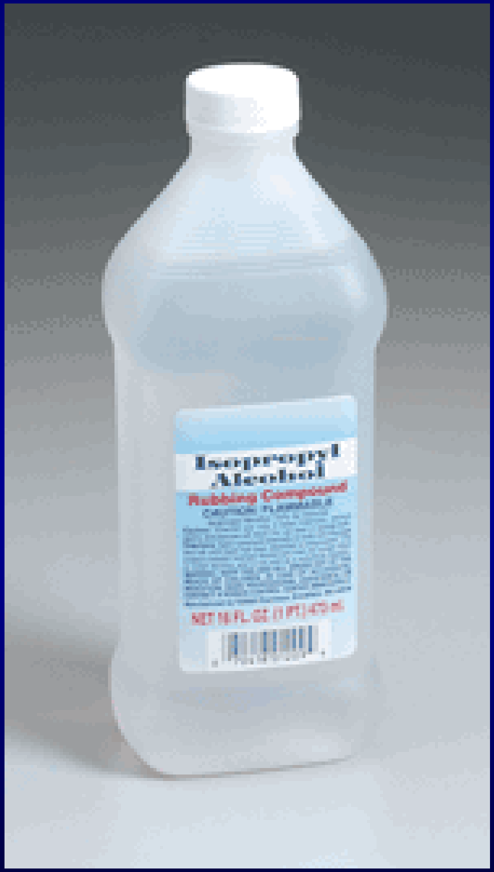

Lab workers Health workers Counselors

Cotton Gauze or Cotton Balls

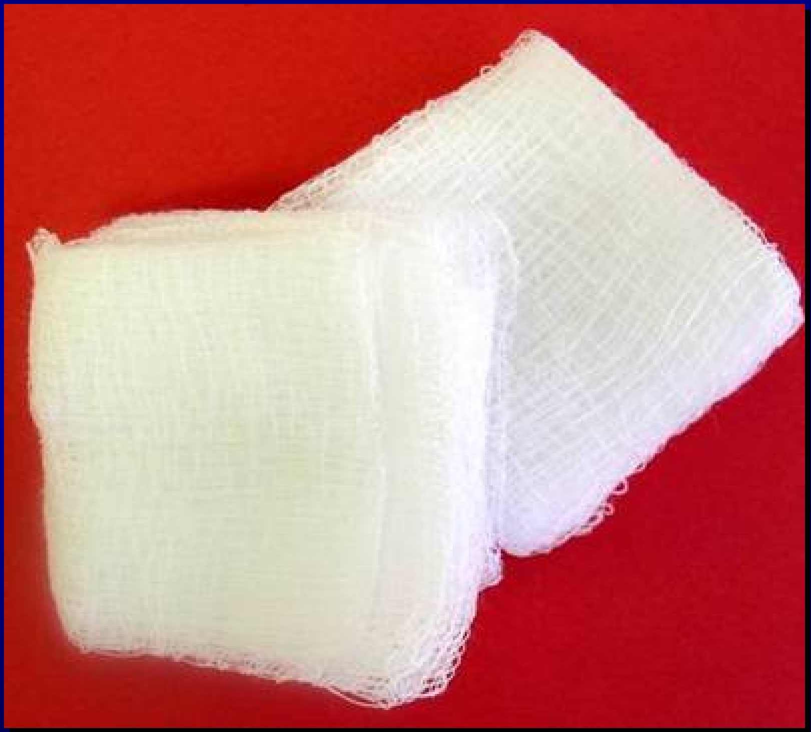

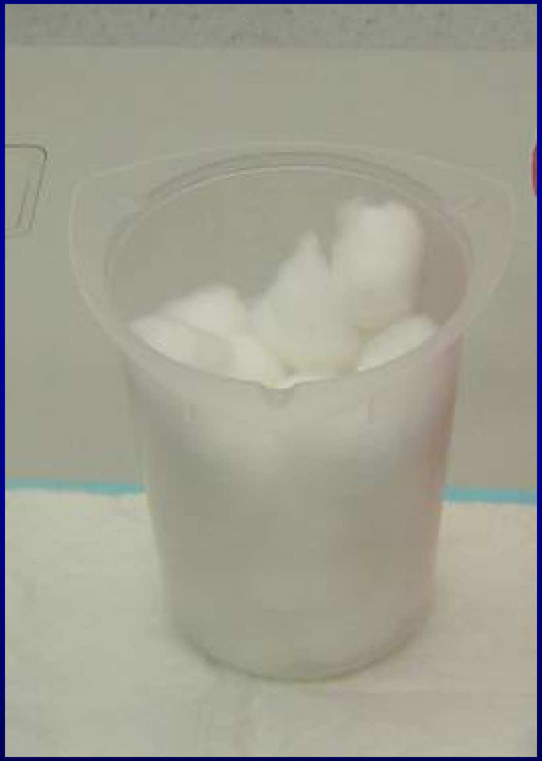

Single-use, hazardous waste disposal

Retractable Safety Lancets

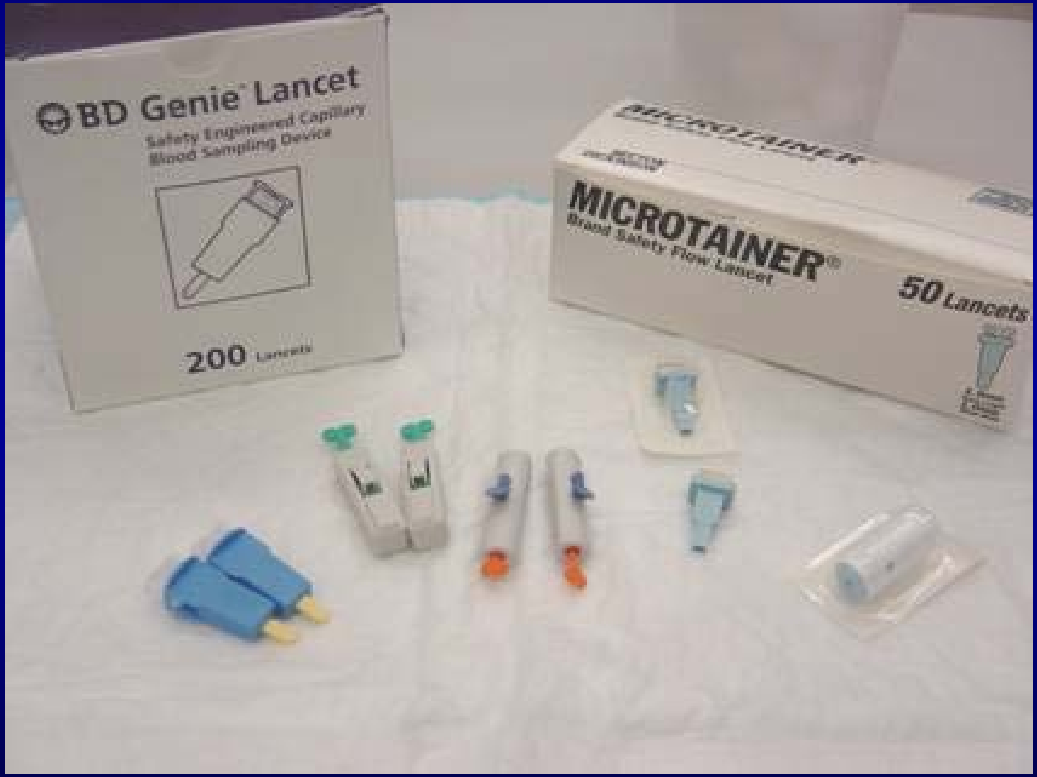

◆ Lab workers ◆ Health workers ◆ Counselors

Timers

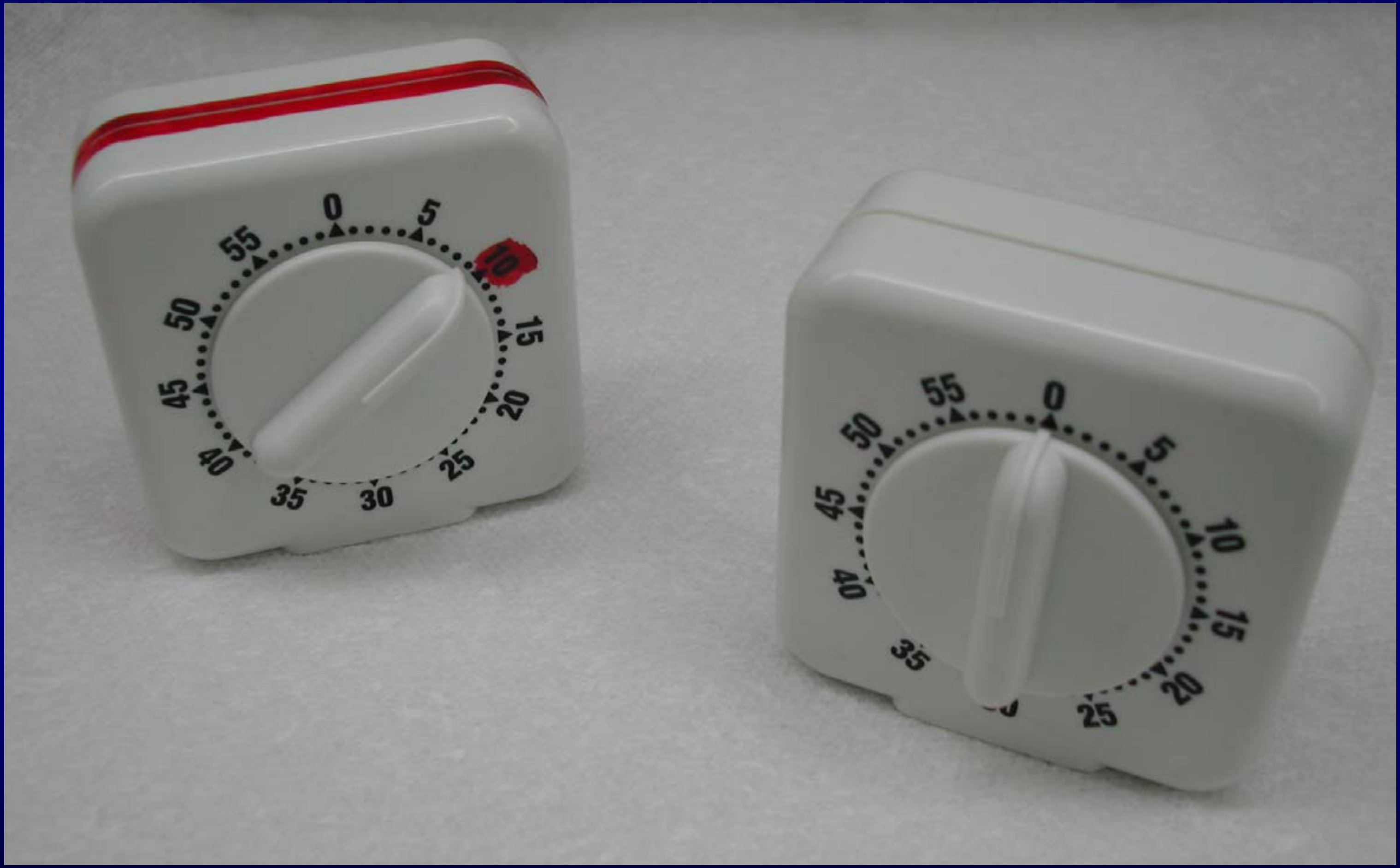

Standard Operating Procedures and Forms

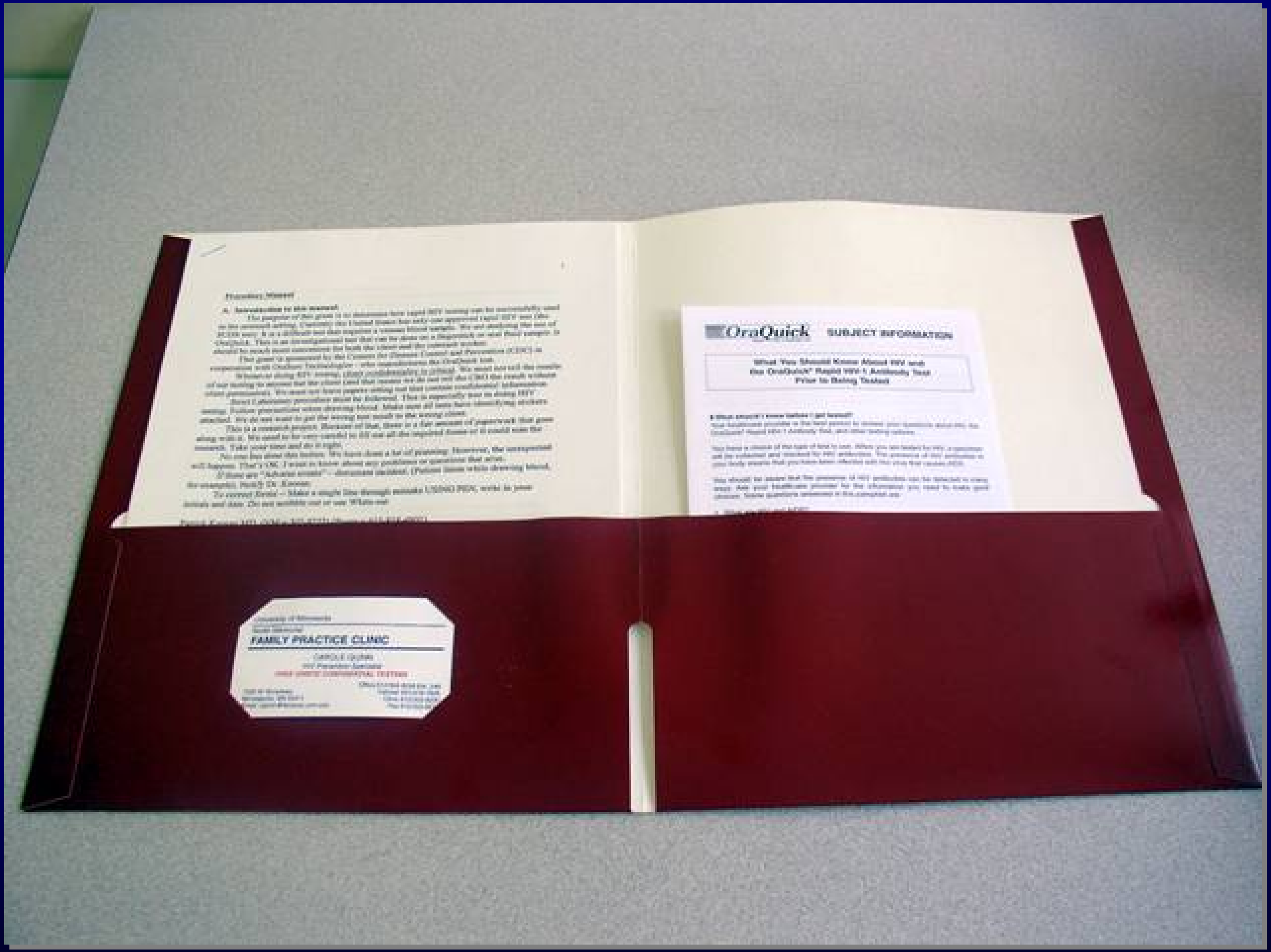

- Lab workers
- Health workers
- Counselors

Labeling Pens and Writing Pens

- Labeling Pens

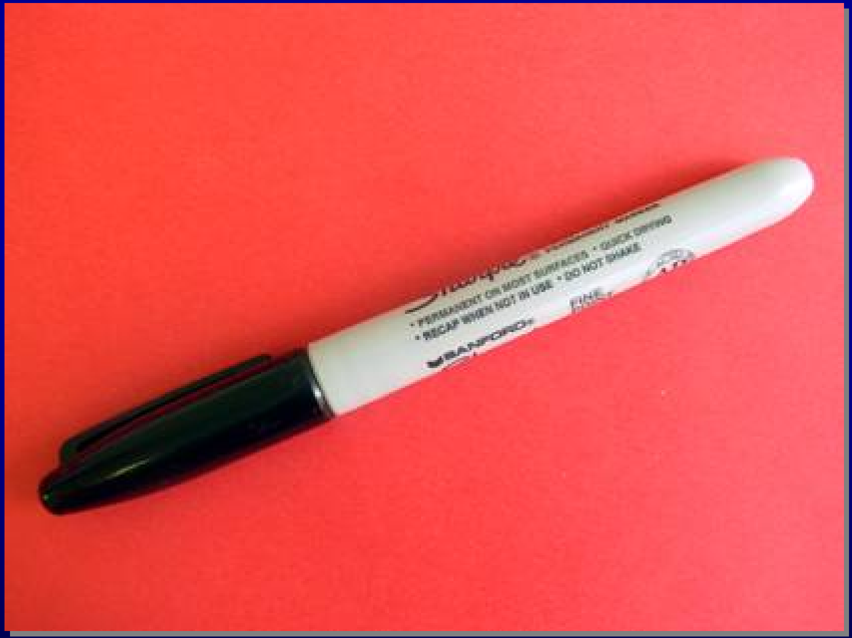

- Writing Pens

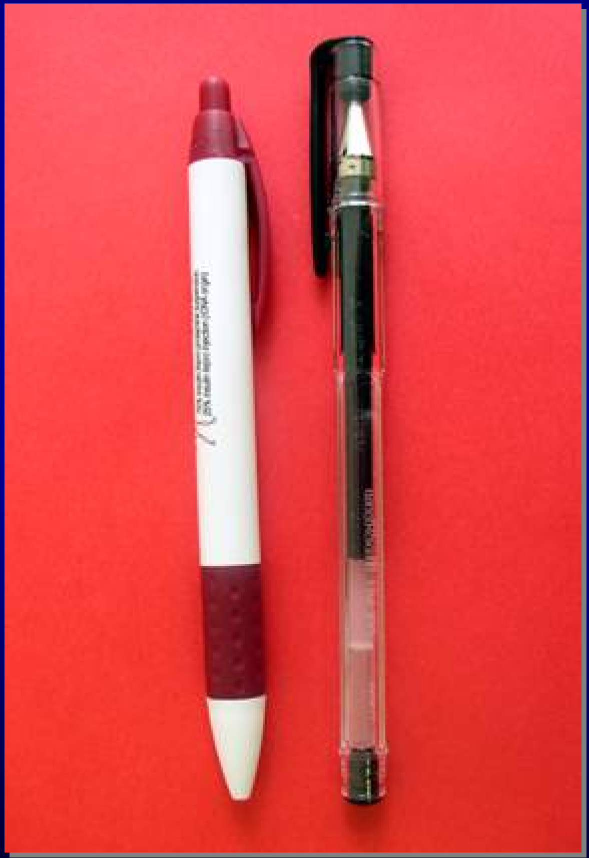

Waste Disposal

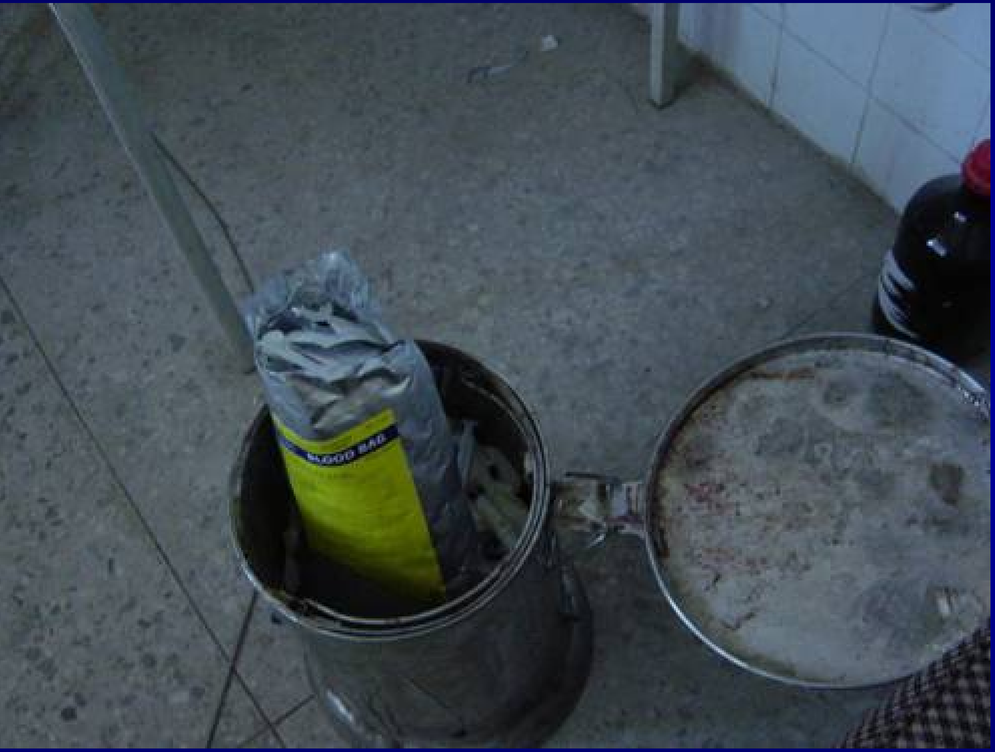

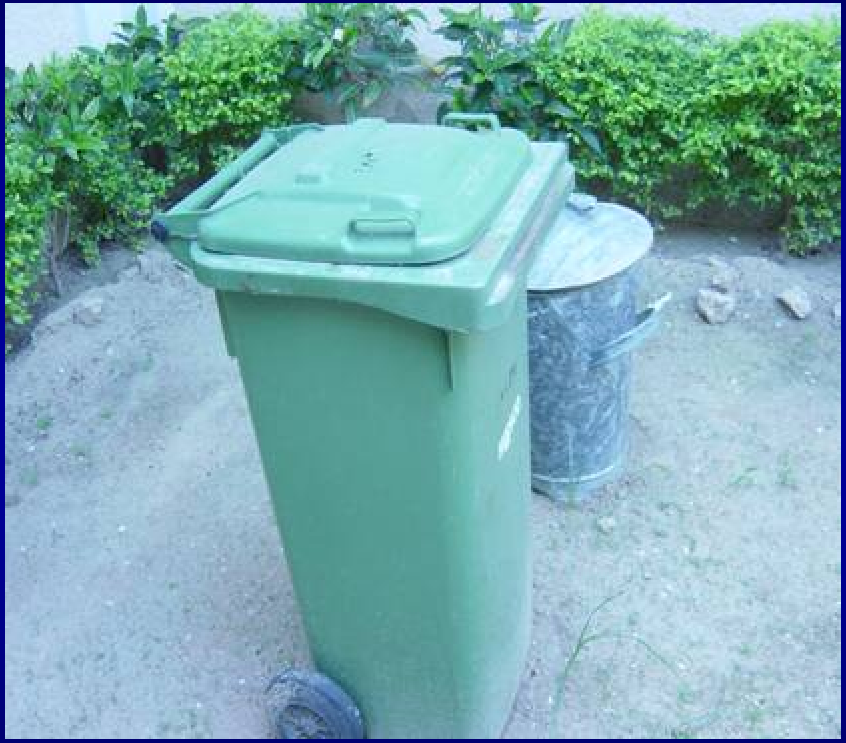

Examine Test Kits

- Display test kits used in-country
- Examine the different components found in each of the Rapid Test kits:
  - Desiccant packet – This is not used when performing the test.
  - Buffer solution – Required for whole blood testing.
  - Transfer pipette or loop
  - Testing device

Organize Your Work Area

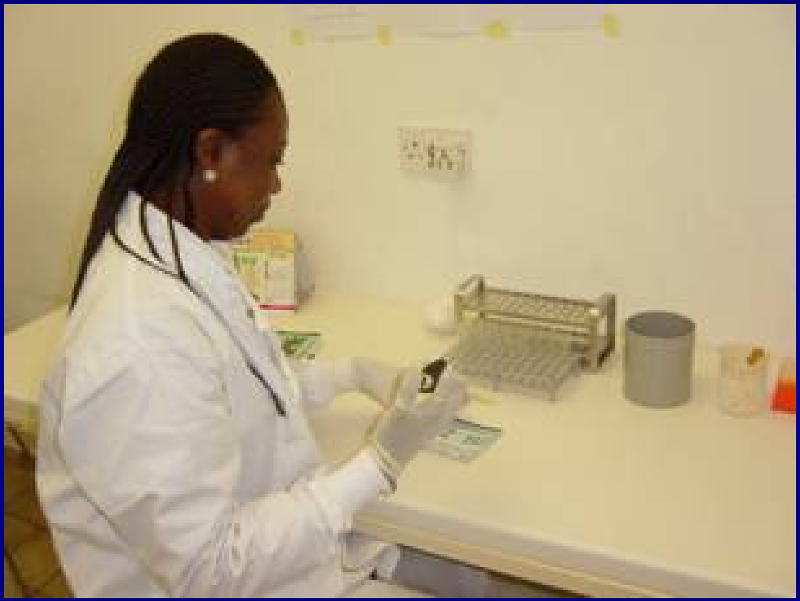

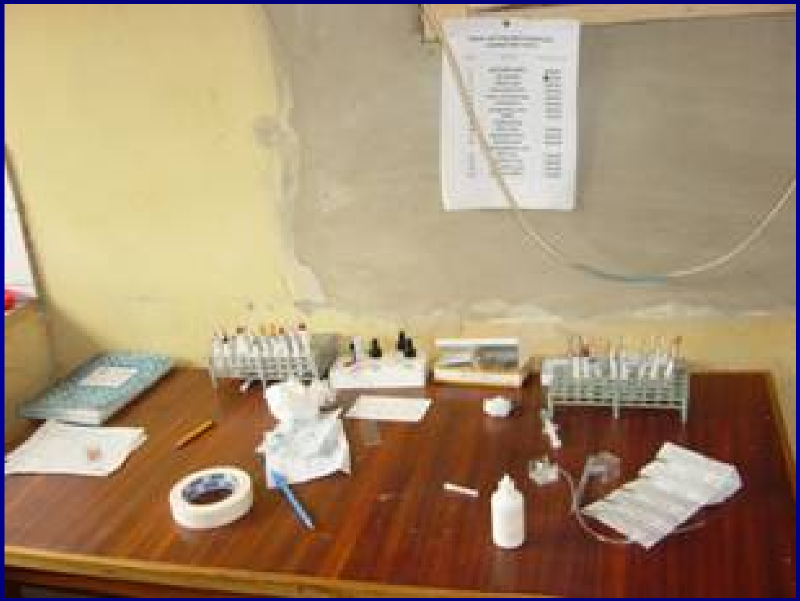

Summary

- What type of lancets will be used for MOH TT "same-visit" testing?
- What are desiccant packs used for?
- What type of bag is needed for waste disposal?
- Which kit uses a loop for sample collection?
- How many QC samples are needed?

Module 8:
Blood Collection – Finger Puncture

Learning Objectives

At the end of this session you will be able to:

- Apply the principle of the finger puncture
- Select the appropriate finger for the procedure
- Perform and collect finger puncture blood accurately and confidently

Content Overview

- Preparing for testing
- Educating your client
- Performing a finger puncture

Puncture Site Selection

Finger selection:

- Middle finger or Ring finger

Area of finger:

- Central fleshy portion of the finger, slightly to the side of the centre and perpendicular to the whorls of the fingerprint

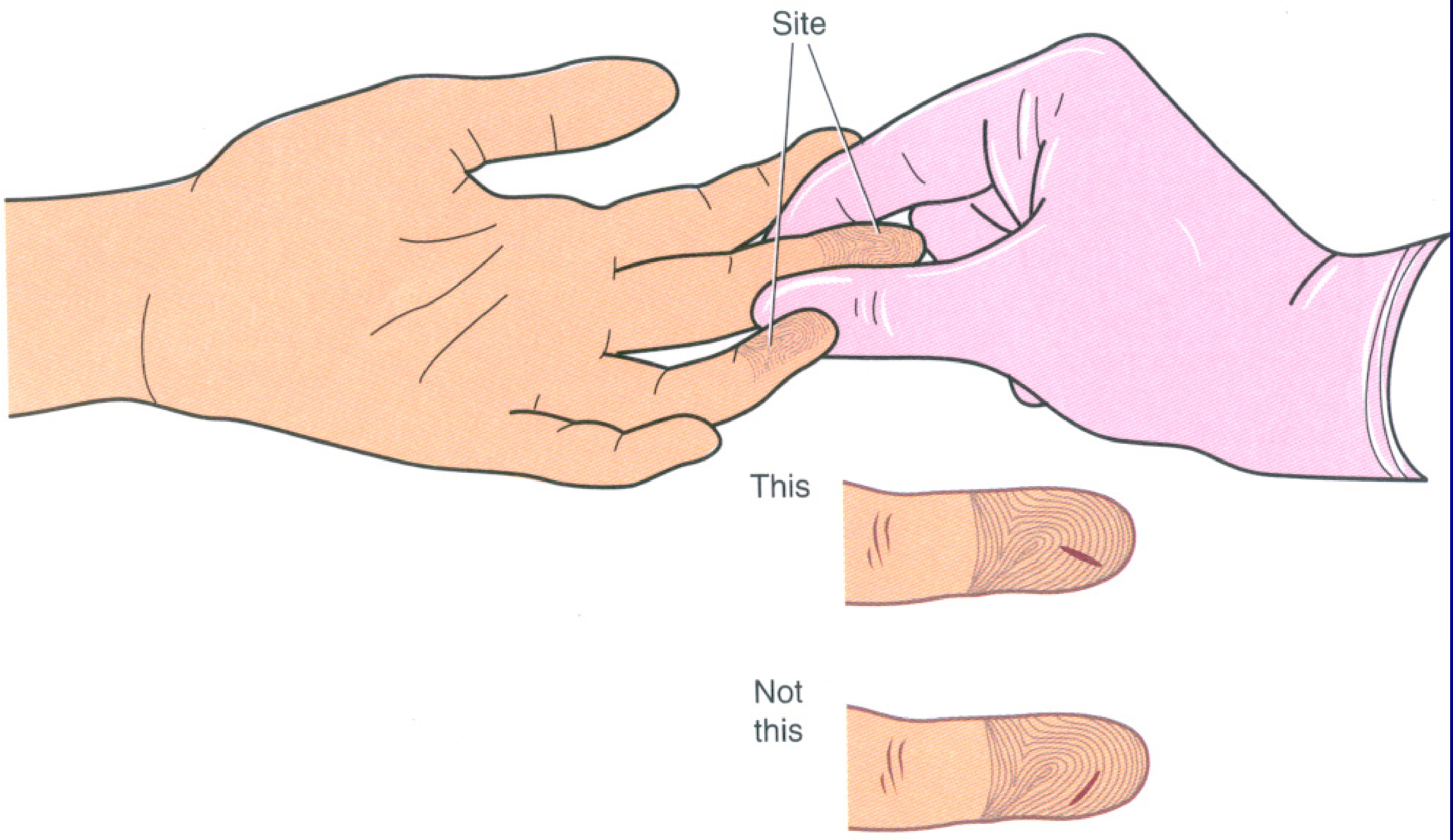

FIGURE 10-9

Recommended site and direction of finger puncture.

Don’ts of Finger Puncture

- Do not puncture the side or the tip of the finger
- Do not puncture parallel to the grooves of the fingerprint
- Do not puncture the index finger
- Do not puncture the little finger
- Do not puncture the fingers of infants or very young children

Finger Puncture Procedure

Finger Puncture – Getting Started

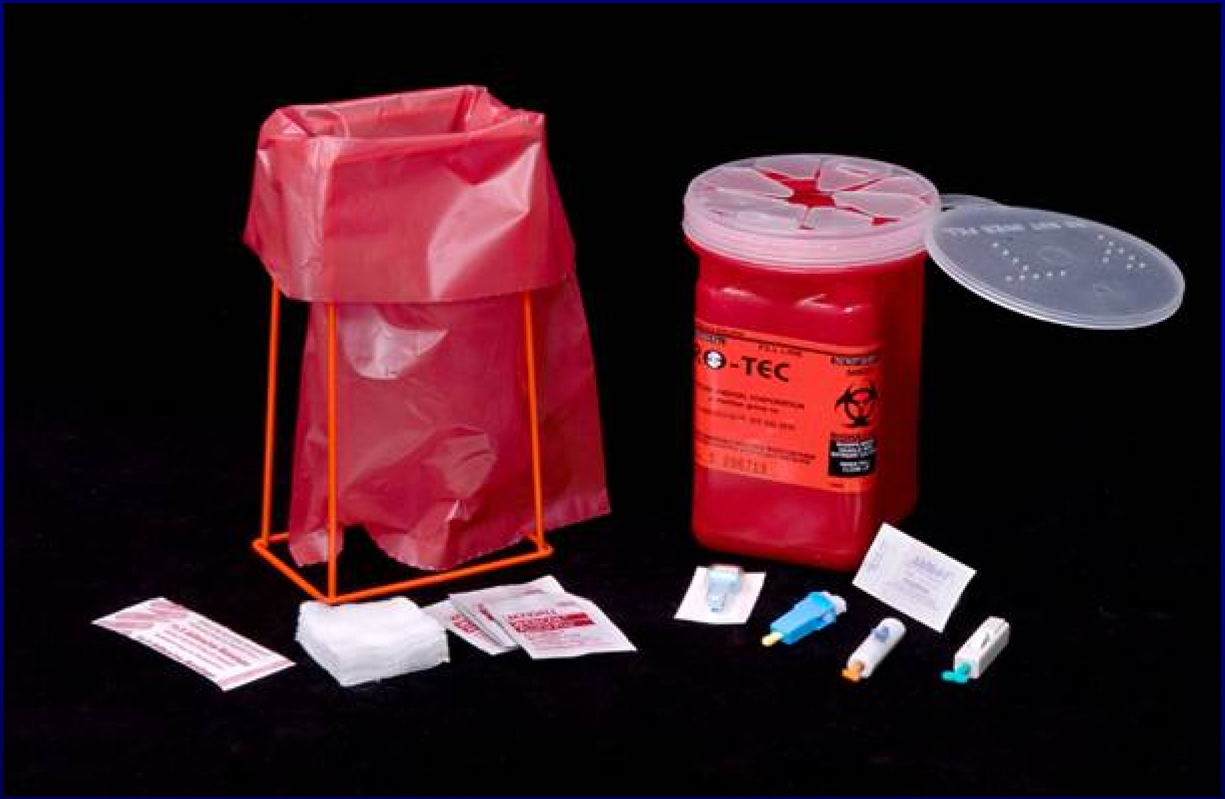

1. Collect supplies

Lab workers   Health workers   Counselors

Finger Puncture – Finger Preparation

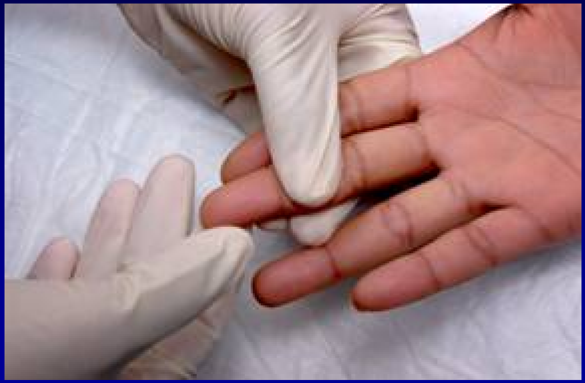

1. Choose whichever finger is least calloused.

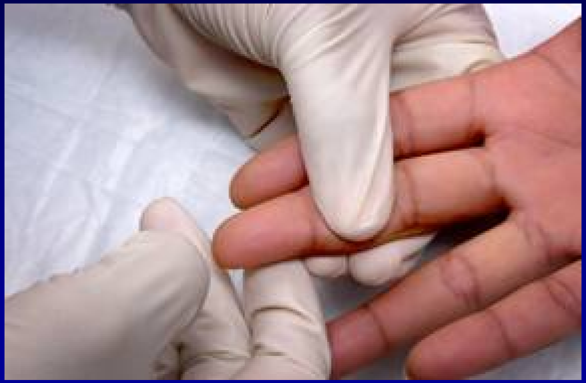

2. Massage finger to increase blood flow.

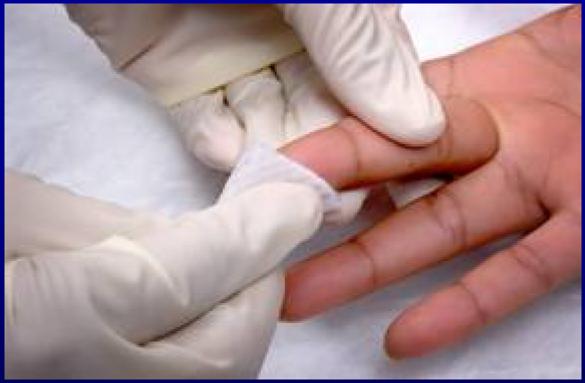

3. Clean the fingertip with alcohol. Work from the middle out to reduce contamination. Allow the area to dry.

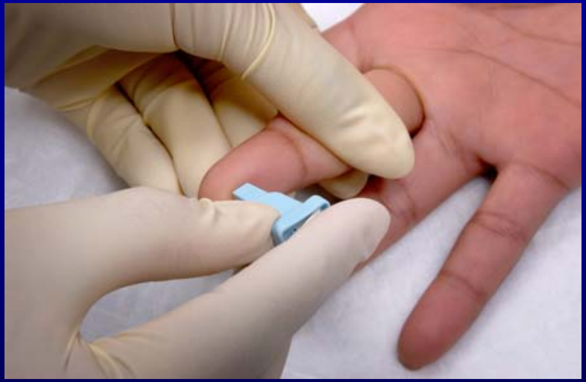

4. Grasp the finger and place a new sterile lancet on the side of the fingertip.

Finger Puncture – Collecting Blood

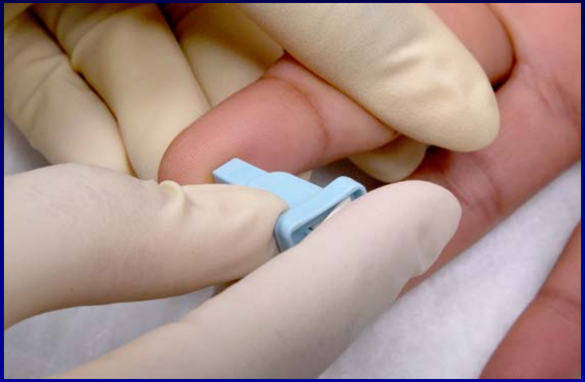

5. Firmly press the lancet to puncture the fingertip

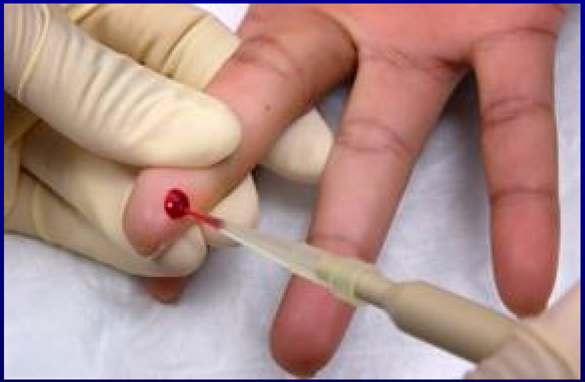

6. Collect the sample. Blood may flow best when the finger is held lower than the elbow.

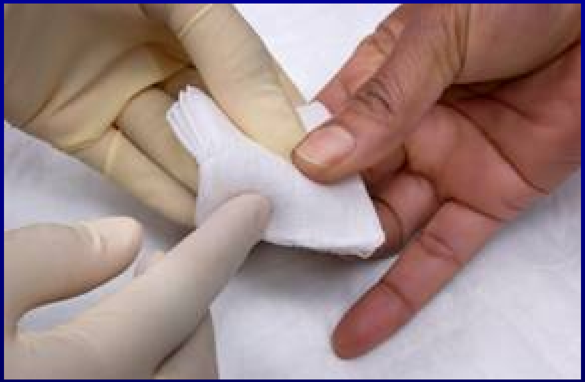

7. Apply a gauze pad or cotton ball to the puncture site until the bleeding stops

Lab workers Health workers Counselors

Finger Puncture - Proper Disposal

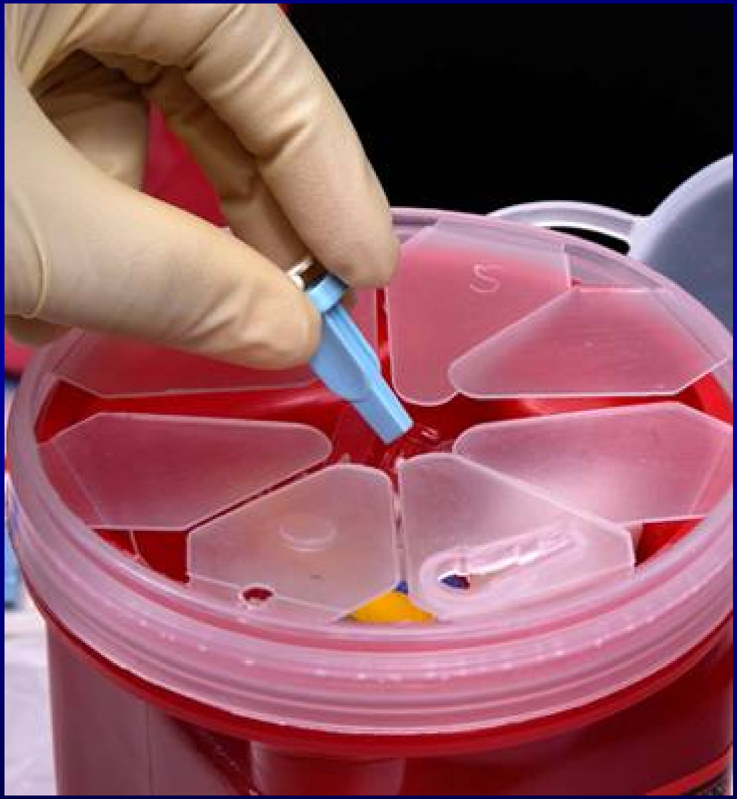

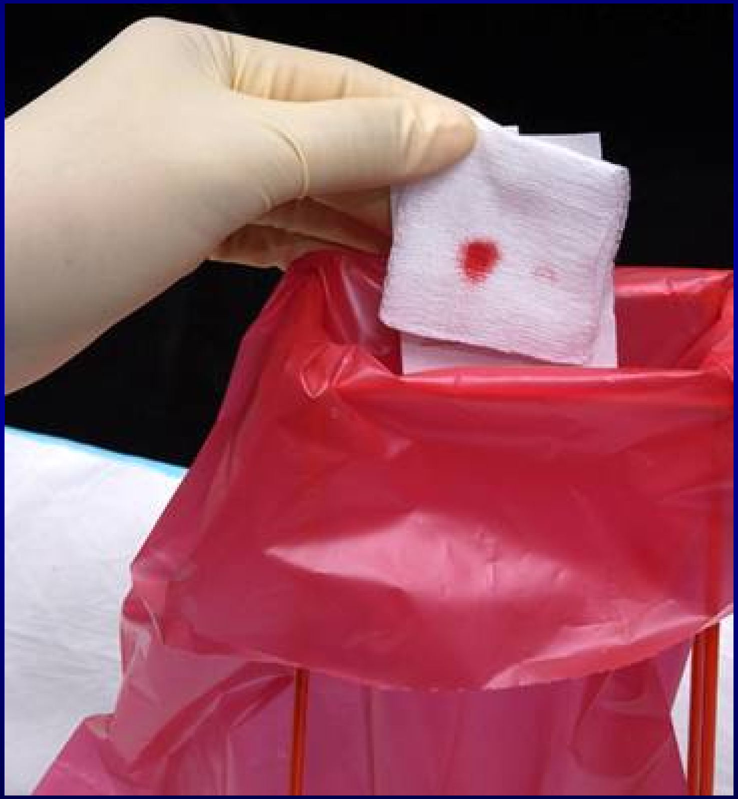

8. Properly dispose of all contaminated supplies

Lab workers Health workers Counselors

Instructor-Led Demonstration

Summary

- How do you put a client at ease while collecting blood?
- What supplies do you need for a finger puncture?
- What are the steps when performing a finger puncture?
- What safety precautions should you follow?

Module 9:
Performing HIV Rapid Tests

Demonstration and Practice

Learning Objectives

- At the end of this module, you will be able to:
  - Define "Standard Operating Procedure" (SOP)
  - Perform MOH TT algorithm tests according to SOPs
    - Uni-Gold
    - Determine
    - Stat-Pak
  - Perform two tests simultaneously
  - Accurately interpret individual test results
  - Accurately determine HIV status

Content Overview

Overview of Testing Procedures

Workspace Setup

Demo + Practice (individual tests on known samples)

Video Presentation and Discussion

Practice
MOH TT algorithm with
blood and/or plasma samples

Lab workers Health workers Counselors

Predictive Value: Single HIV Test

Test Specificity 99.8%

HIV Prevalence	True +	False +	Positive Predictive Value
10%	100	2	98%
5%	50	2	96%
2%	20	2	91%
1%	10	2	83%
0.5%	5	2	71%
0.2%	2	2	50%
01.%	1	2	33%

Determine

Determine: Getting Ready

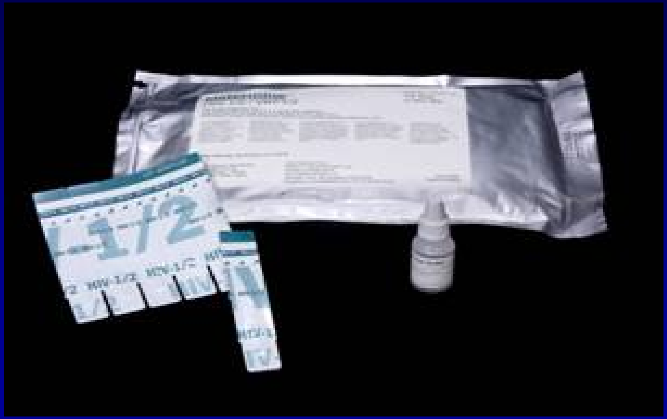

1. Collect test items

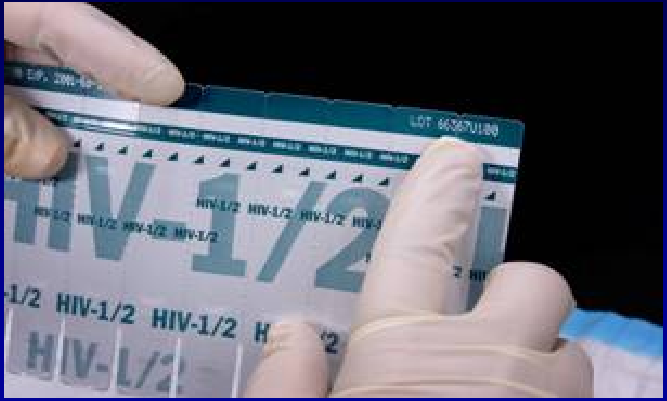

2. Use 1 strip per test
   Preserve the lot number

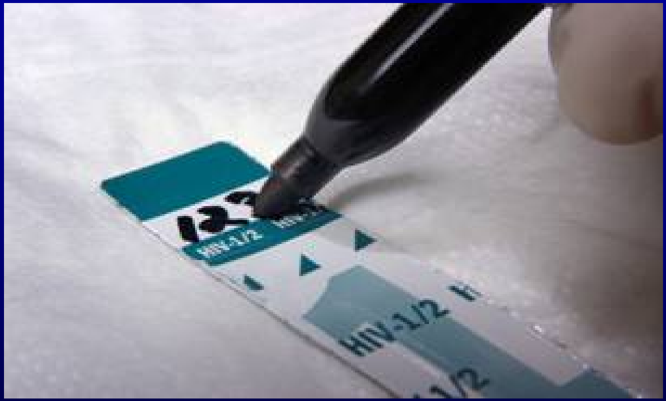

3. Label the test strip with client identification number

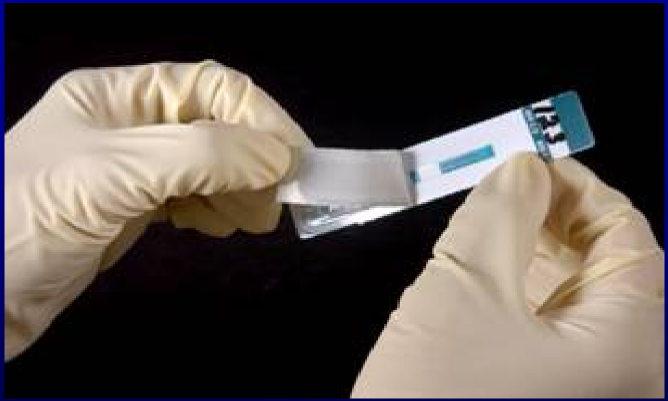

4. Remove protective foil cover

Determine: Collecting Specimen

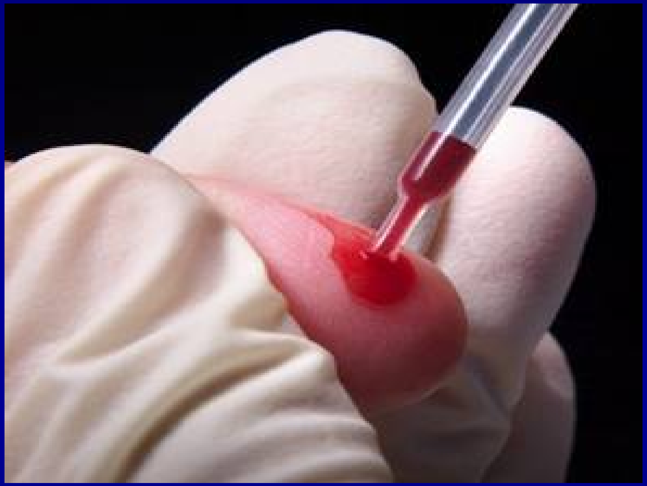

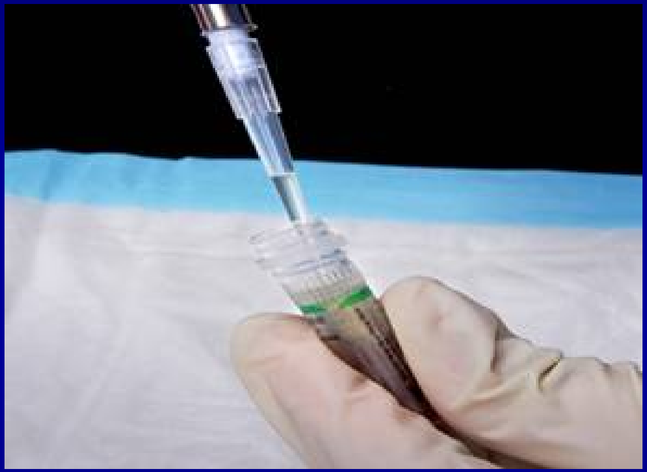

5. Collect 1 drop of blood using a plastic transfer pipette

Determine: Applying Sample and Buffer to Test Strip

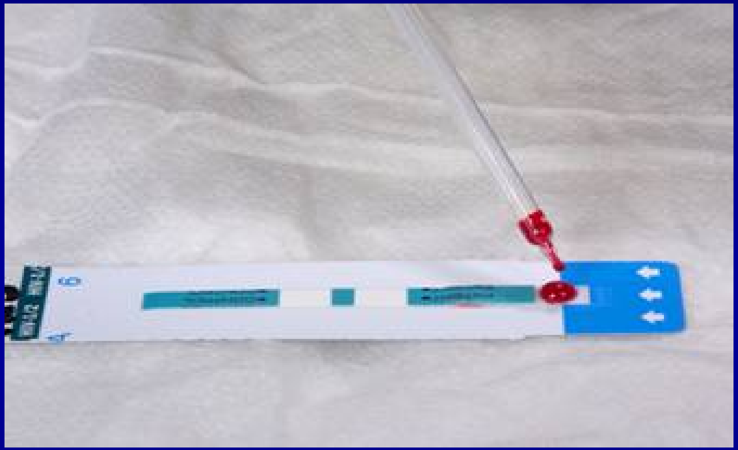

6. Apply the sample to the absorbent pad on the strip

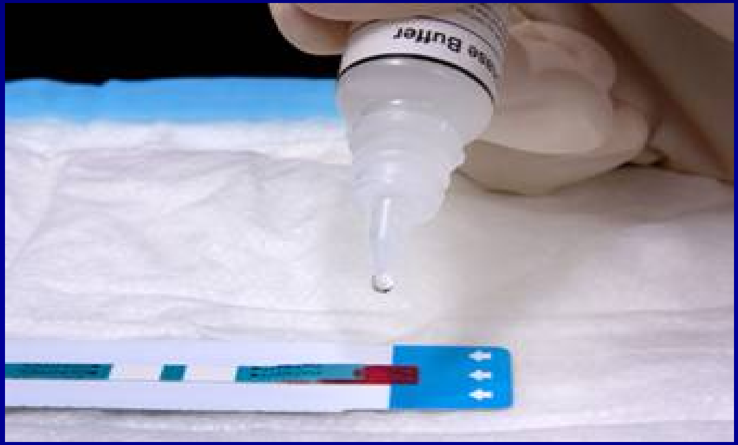

7. Add 1 drop of chase buffer to the specimen pad

Determine: Getting Results

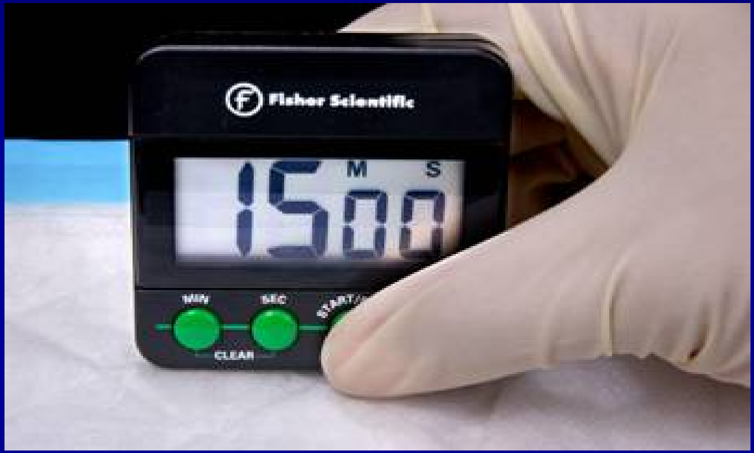

8. Wait 15 minutes before reading the results

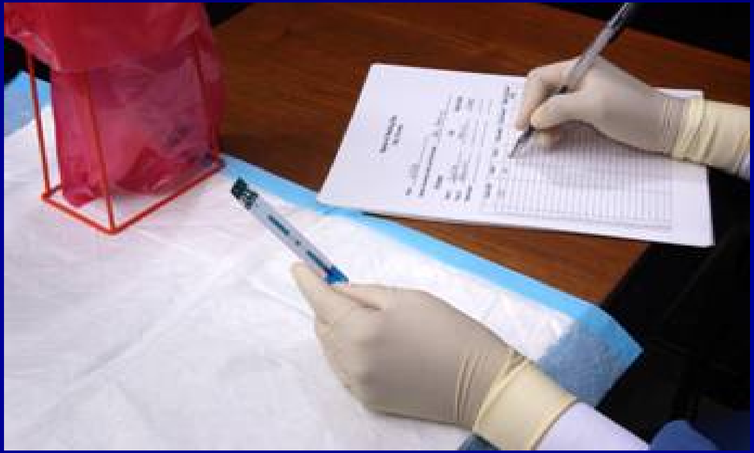

9. Read and record the results

Lab workers   Health workers   Counselors

Determine - Test Interpretation

Positive

Negative

Invalid

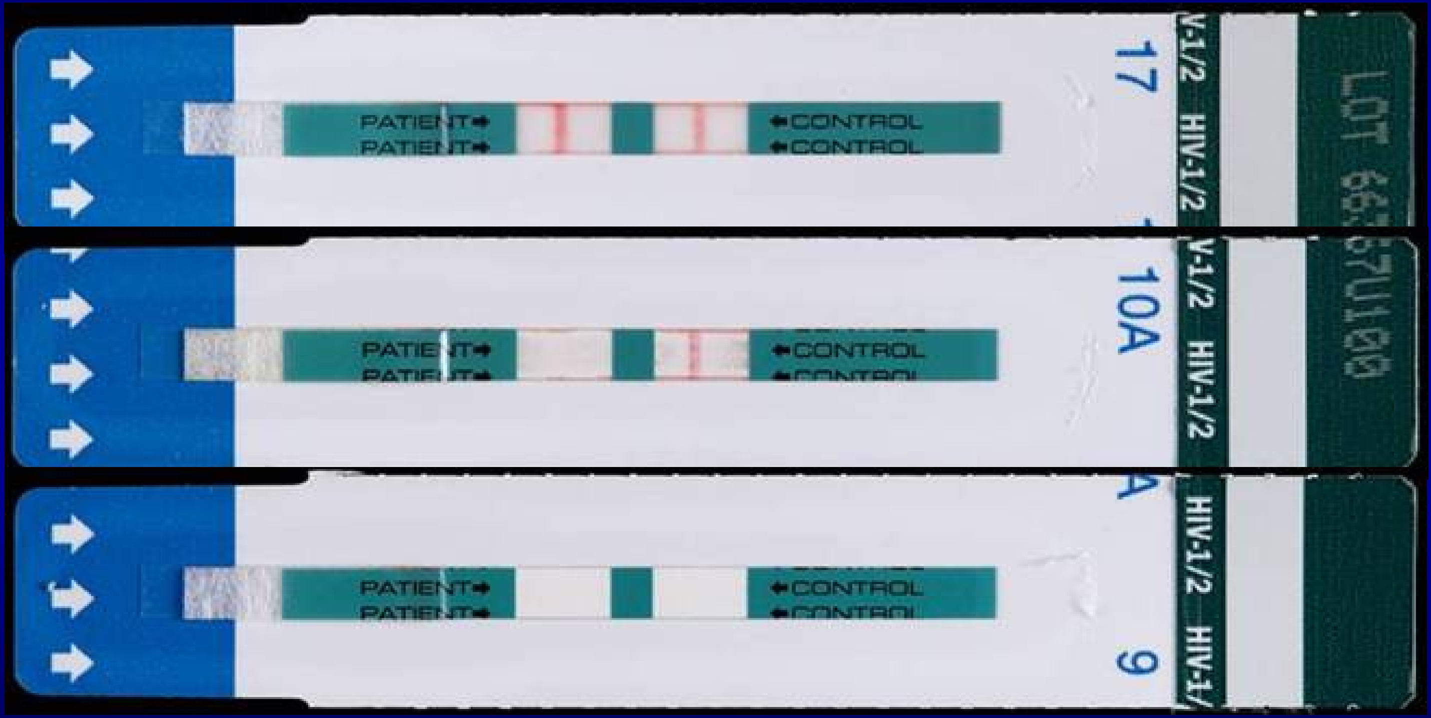

Lab workers Health workers Counselors

Uni-Gold

Uni-Gold: Getting Ready

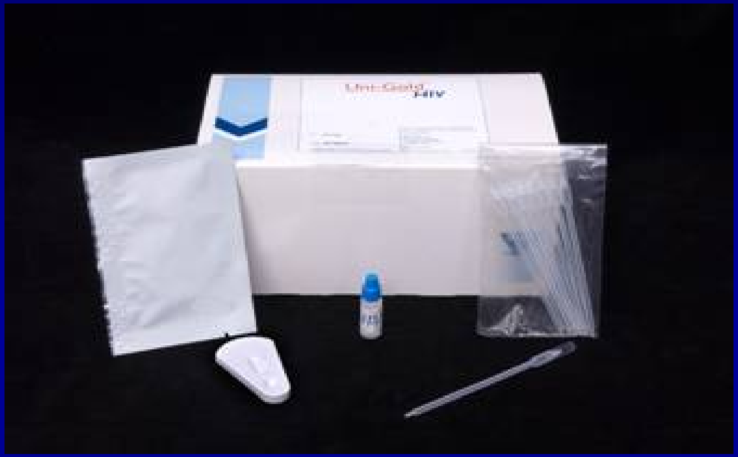

1. Collect test items

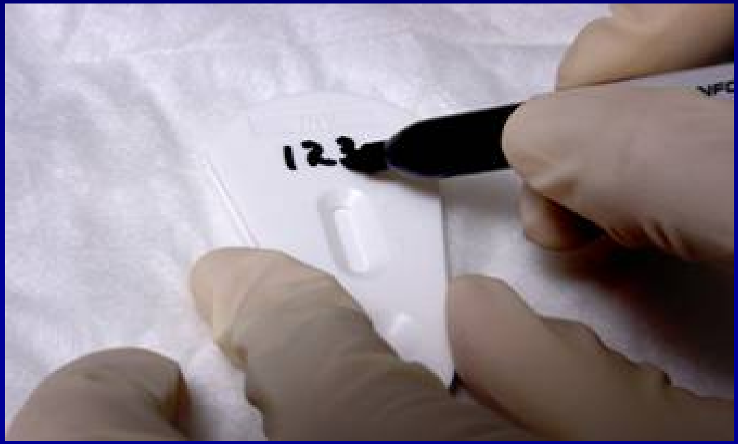

2. Remove device from package and label it

Lab workers   Health workers   Counselors

Uni-Gold: Collecting Specimen

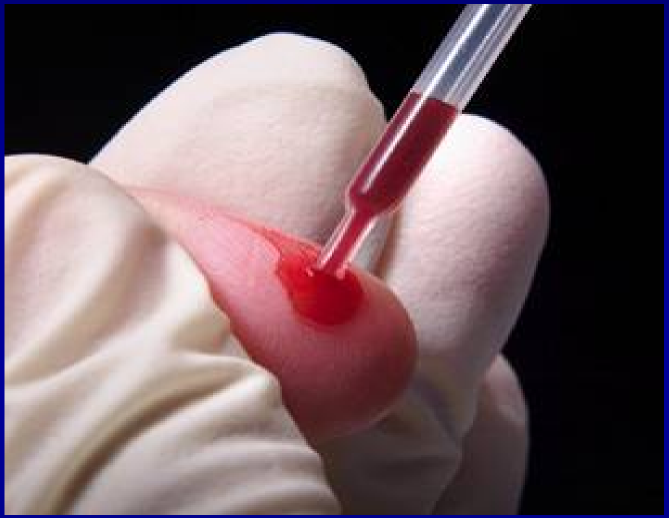

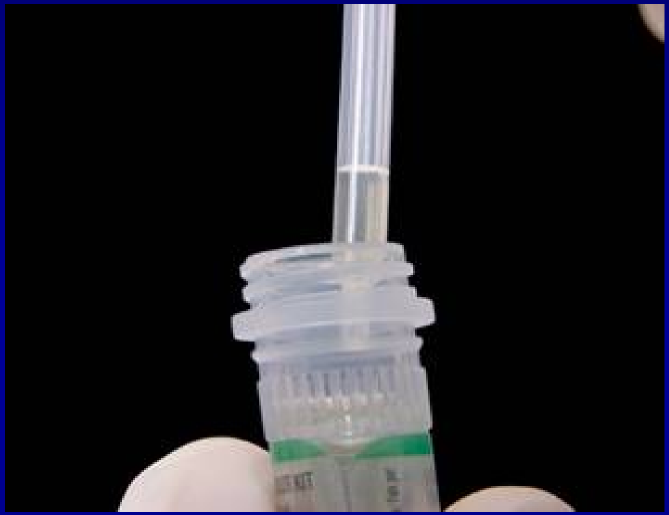

3. Collect specimen using the disposable pipette

Uni-Gold: Adding Sample and Reagent to Test Device

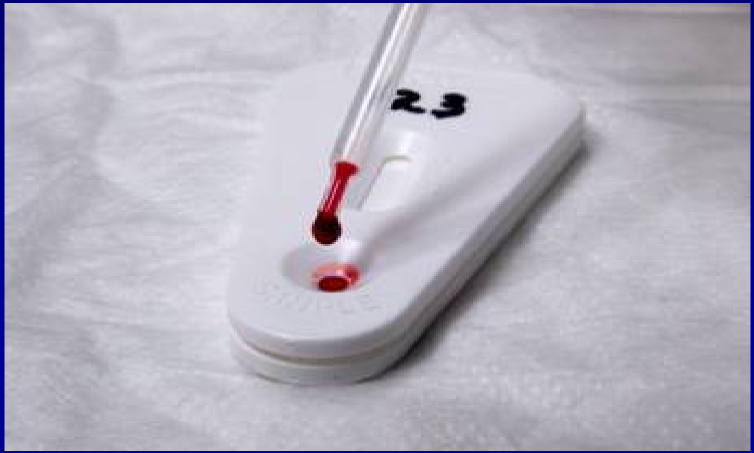

4. Add 2 drops of sample to the sample port

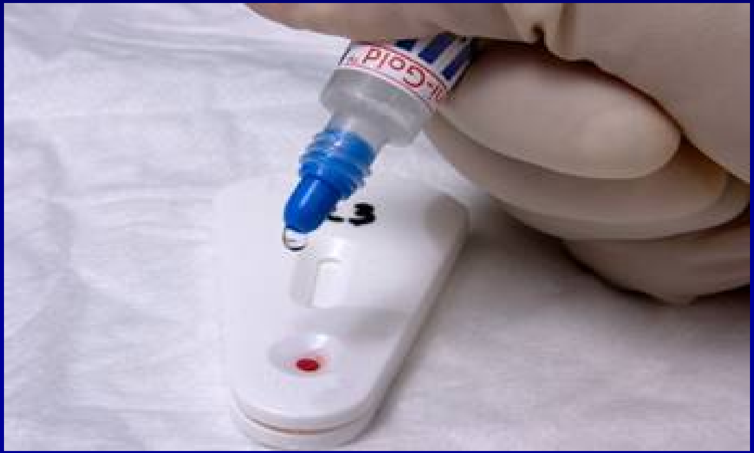

5. Add 2 drops of wash buffer to sample port

Lab workers Health workers Counselors

Uni-Gold: Getting Results

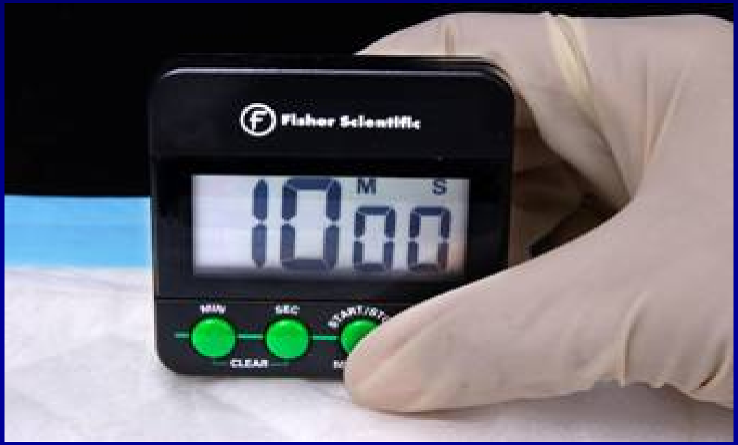

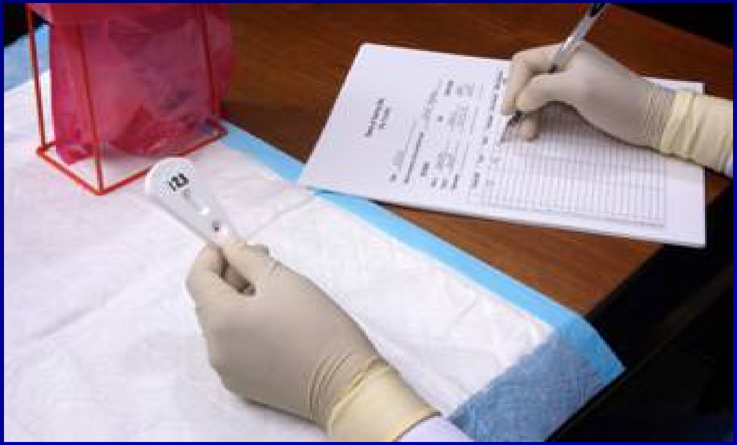

6. Wait for 10 minutes to read the results

7. Read and record the results

Uni-Gold: Test Interpretation

Positive	Negative	Invalid
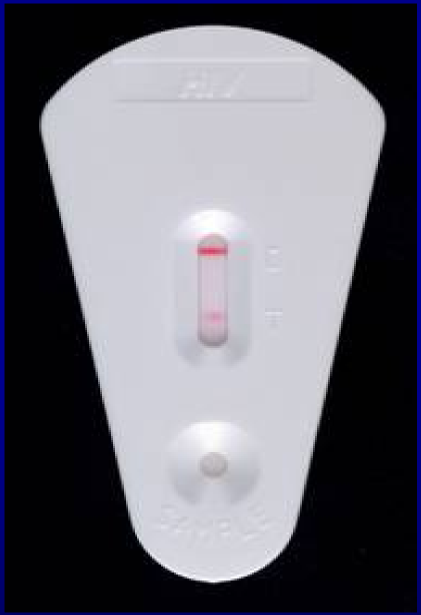	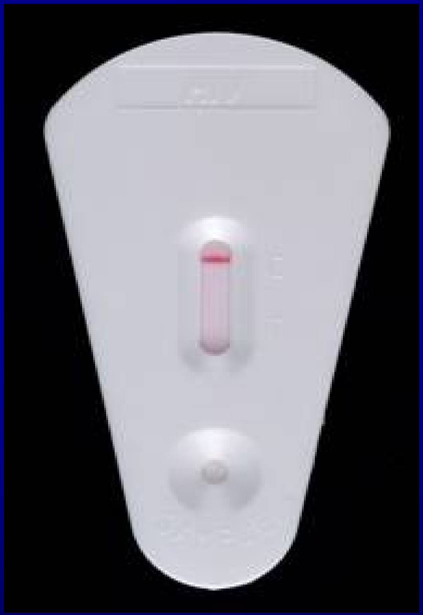	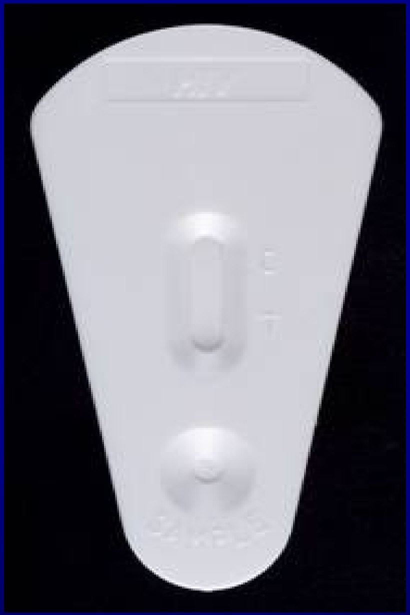

Lab workers   Health workers   Counselors

Stat-Pak

Stat-Pak: Getting Ready

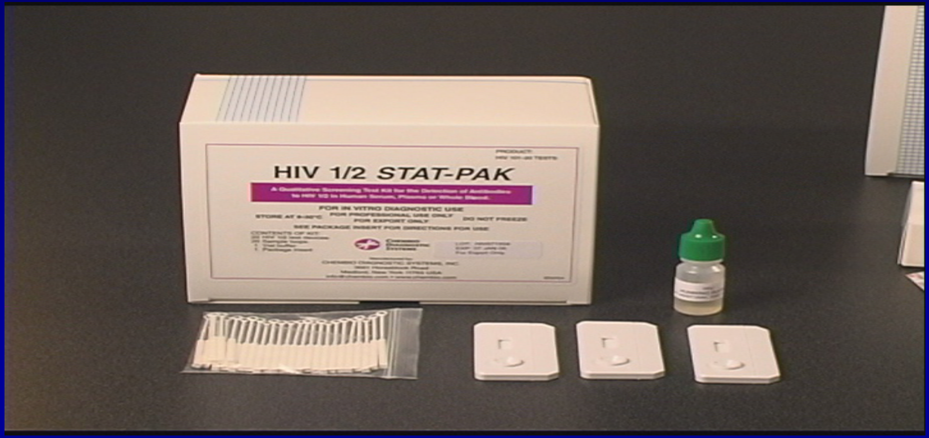

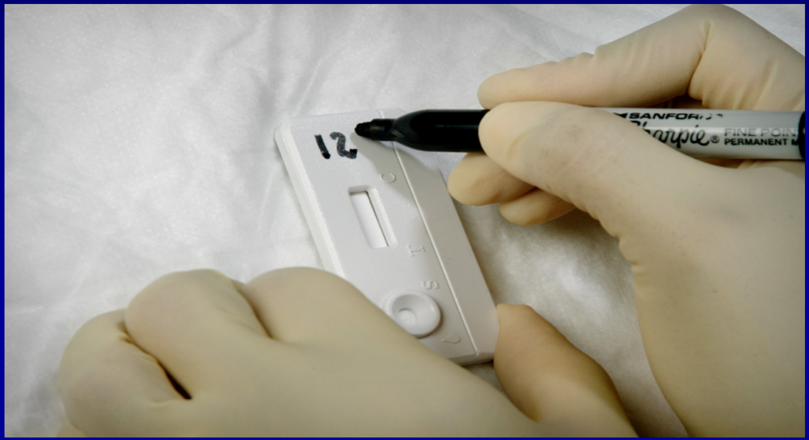

1. Collect test items

2. Remove device from package and label it

Lab workers   Health workers   Counselors

Stat-Pak: Collecting Specimen

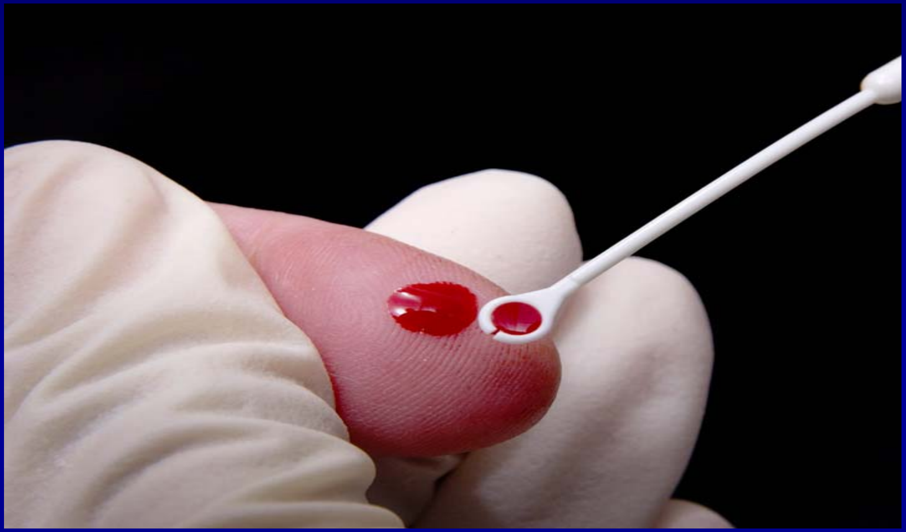

3. Collect specimen using the calibrated loop

Stat-Pak: Adding Specimen and Reagent to Test Device

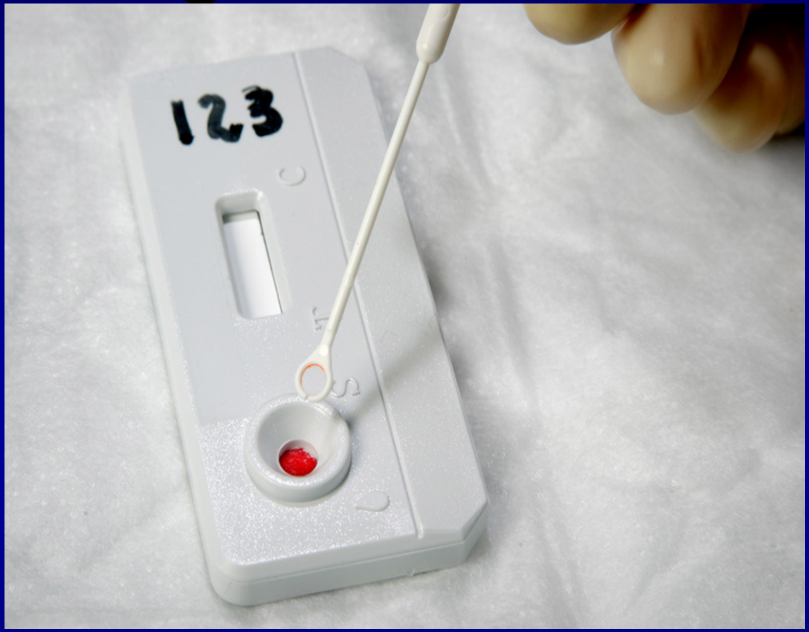

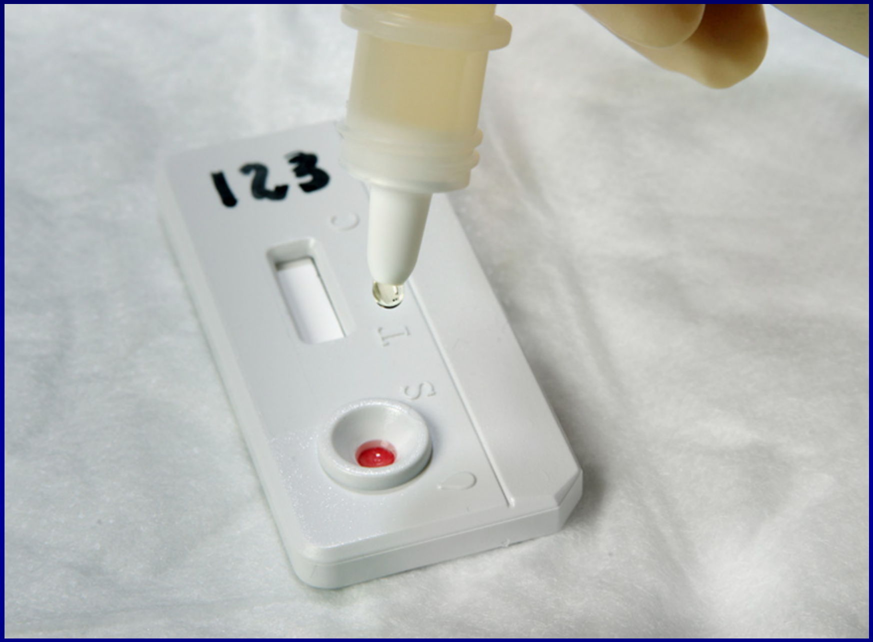

4. Add sample to center of SAMPLE well.

5. Add 3 drops of buffer. Drop buffer directly over SAMPLE well.

Lab workers Health workers Counselors

Stat-Pak: Getting Results

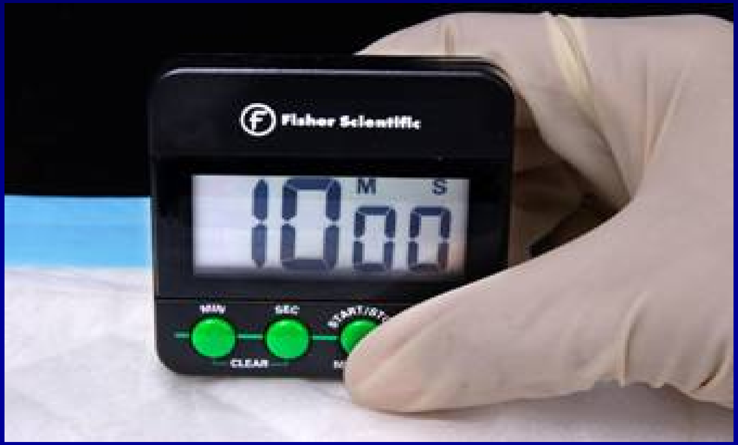

6. Wait for 10 minutes to read the results

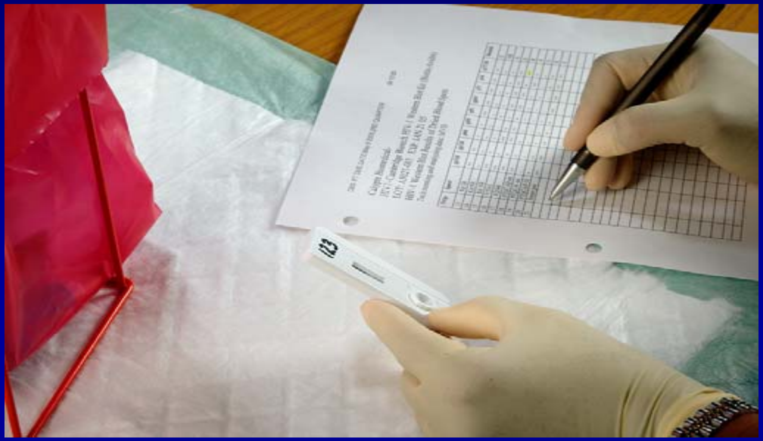

7. Read and record the results

Stat-Pak: Test Interpretation

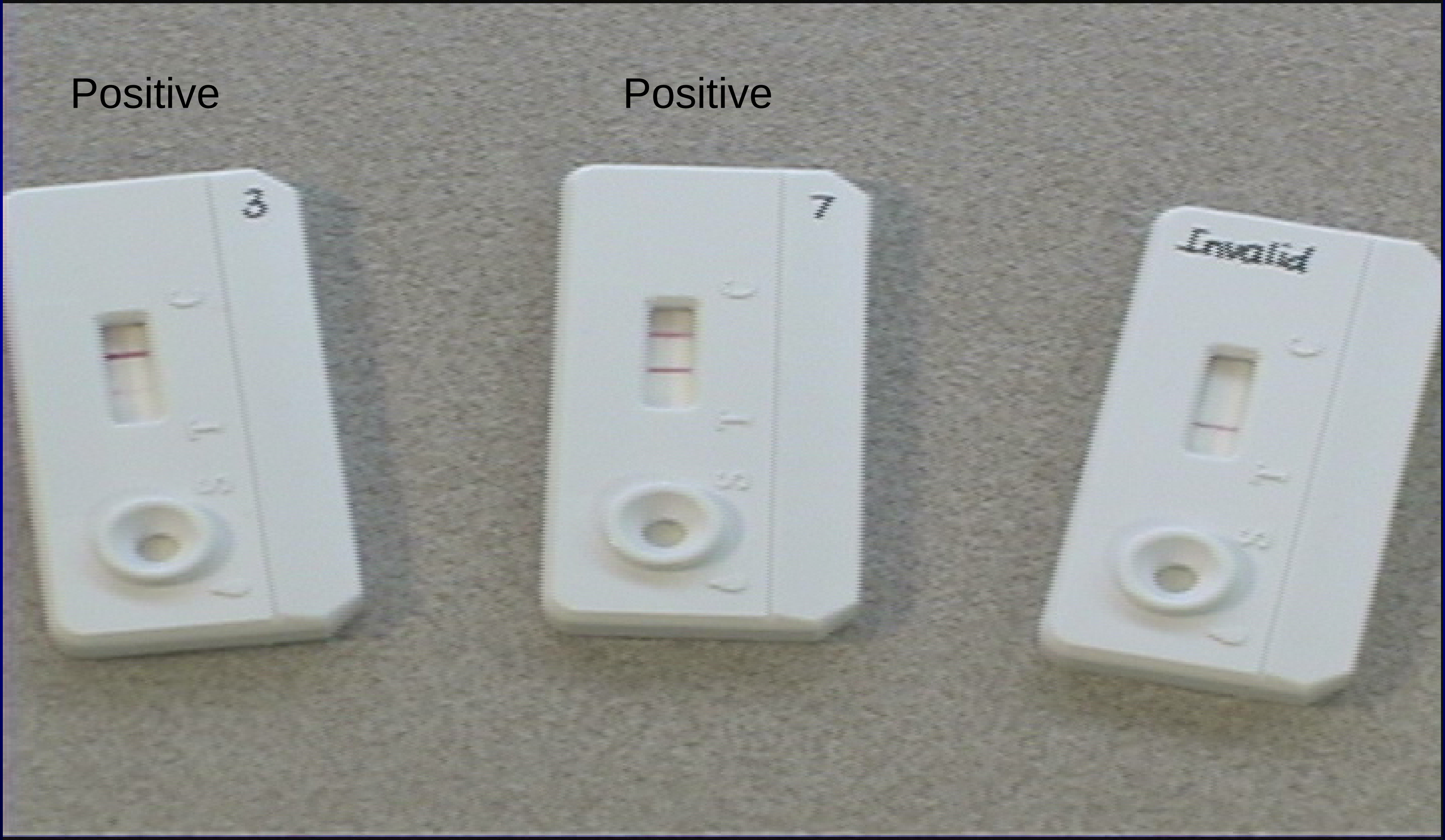

Lab workers   Health workers   Counselors

Stat-Pak: Test Interpretation

Positive

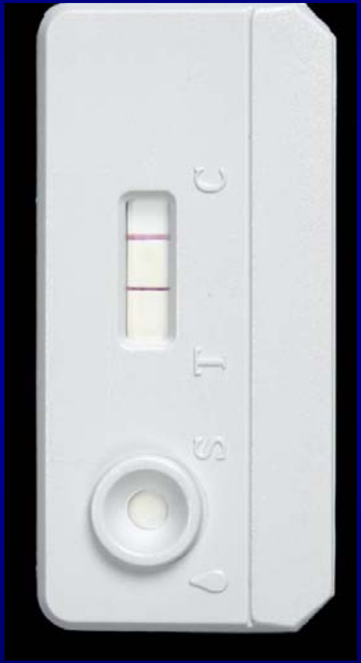

Lines of any intensity in **both** control and test areas.

Negative

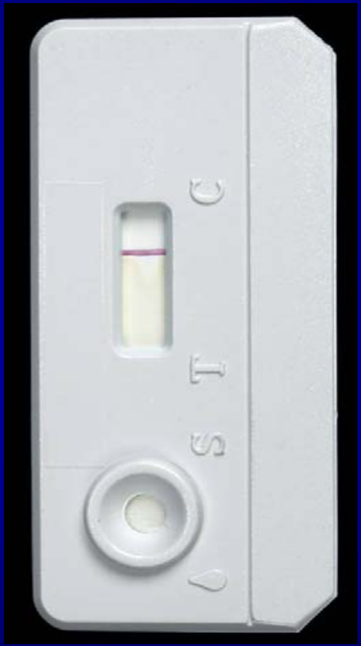

Line in control area only.

Invalid

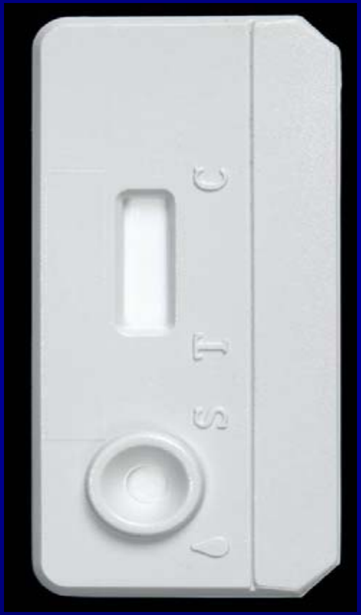

No line in control area. Do not report invalid results.

Activity: Workspace Setup

- Identify your workspace
- Gather test kits and supplies
- Obtain positive and negative samples from instructor
- Arrange all items at your work station

- Activity time: 10 minutes

Hands-On Practice: Individual Tests

Instructions:

- Use safety precautions
- Practice with samples provided by your instructor only
- Raise your hand if you need additional supplies
- Show your test results to instructor after you are done

Total time: 15 minutes per test

Video Presentation and Discussion

Video: Determine

- What preparation is required before testing?
- What are the components in the test kit?
- What must you preserve when separating test strips from the packet?
- What information needs to be recorded, and where?
- How do you collect blood? What device do you use?
- How long do you set the timer?
- How many results are possible? How do you read them?

Video: Uni-Gold

- What preparation is required before testing?
- What are the components in the test kit?
- What information needs to be recorded, and where?
- How do you collect blood? What device do you use?
- How long do you set the timer?
- How many results are possible? How do you read them?

Video: Multiple HIV Tests

- Why must you keep two test kits separate when performing both at the same time?
- Do you collect blood at the same time or separately when performing multiple tests?
- How do you set the timer when two tests require different wait time?
- When is the third test used?
- How does it determine HIV status?

MOH TT Algorithm

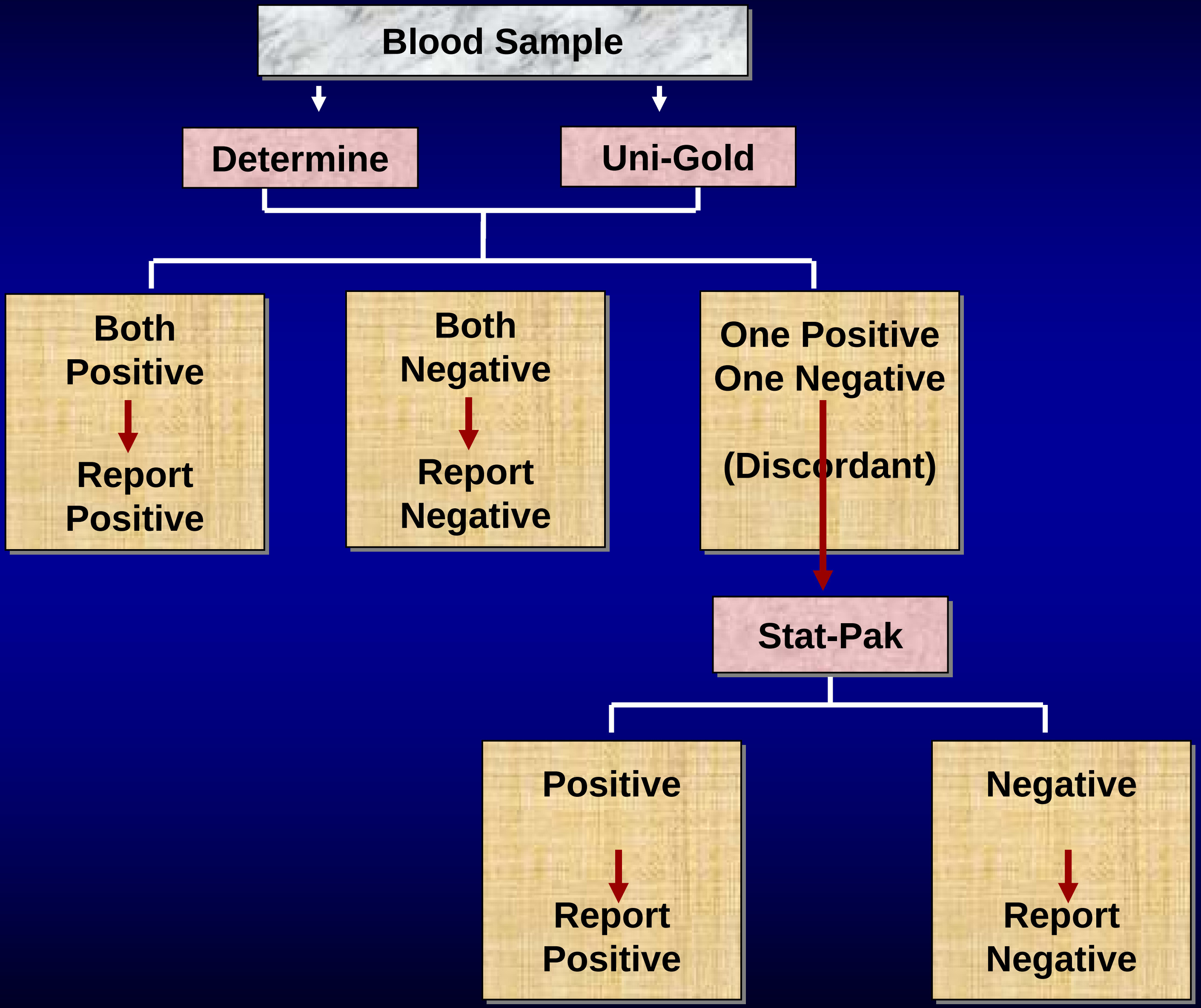

- Lab workers
- Health workers
- Counselors

Possible Outcomes in Parallel Algorithms

TEST 1	TEST 2	TEST 3	HIV Status
Negative	Negative		Negative
Positive	Positive		Positive
Negative	Positive	Negative	Negative
Positive	Negative	Negative	Negative
Negative	Positive	Positive	Positive
Positive	Negative	Positive	Positive

Hands-On Practice: Parallel Algorithm

Conduct Determine, Uni-Gold and Stat-Pak tests simultaneously following the SOPs. Record the HIV status of each sample.

Specimen		
1	→ Determine – Uni-Gold – Stat-Pak	→ HIV Status
2	→ Determine – Uni-Gold – Stat-Pak	→ HIV Status
3	→ Determine – Uni-Gold – Stat-Pak	→ HIV Status
4	→ Determine – Uni-Gold – Stat-Pak	→ HIV Status
5	→ Determine – Uni-Gold – Stat-Pak	→ HIV Status

Lab workers   Health workers   Counselors

Hands-On Practice: Parallel Algorithm

- Collect supplies
- Obtain panel of blind specimens
- Organize your workspace
- Complete one algorithm before starting the next
  - Use safety precautions
  - Practice only on samples provided
  - Raise your hand for additional supplies
- Record results on worksheet
- Do not discard test devices - instructor will check results

Summary

- Describe the key learning from performing:
  - (Uni-Gold)
  - (Determine)
  - (Stat-Pak)

- Describe the key learning from performing the multi-test algorithm

Module 10: Inventory

Managing Stock at the HIV Rapid Testing Site

Quality HIV Testing System

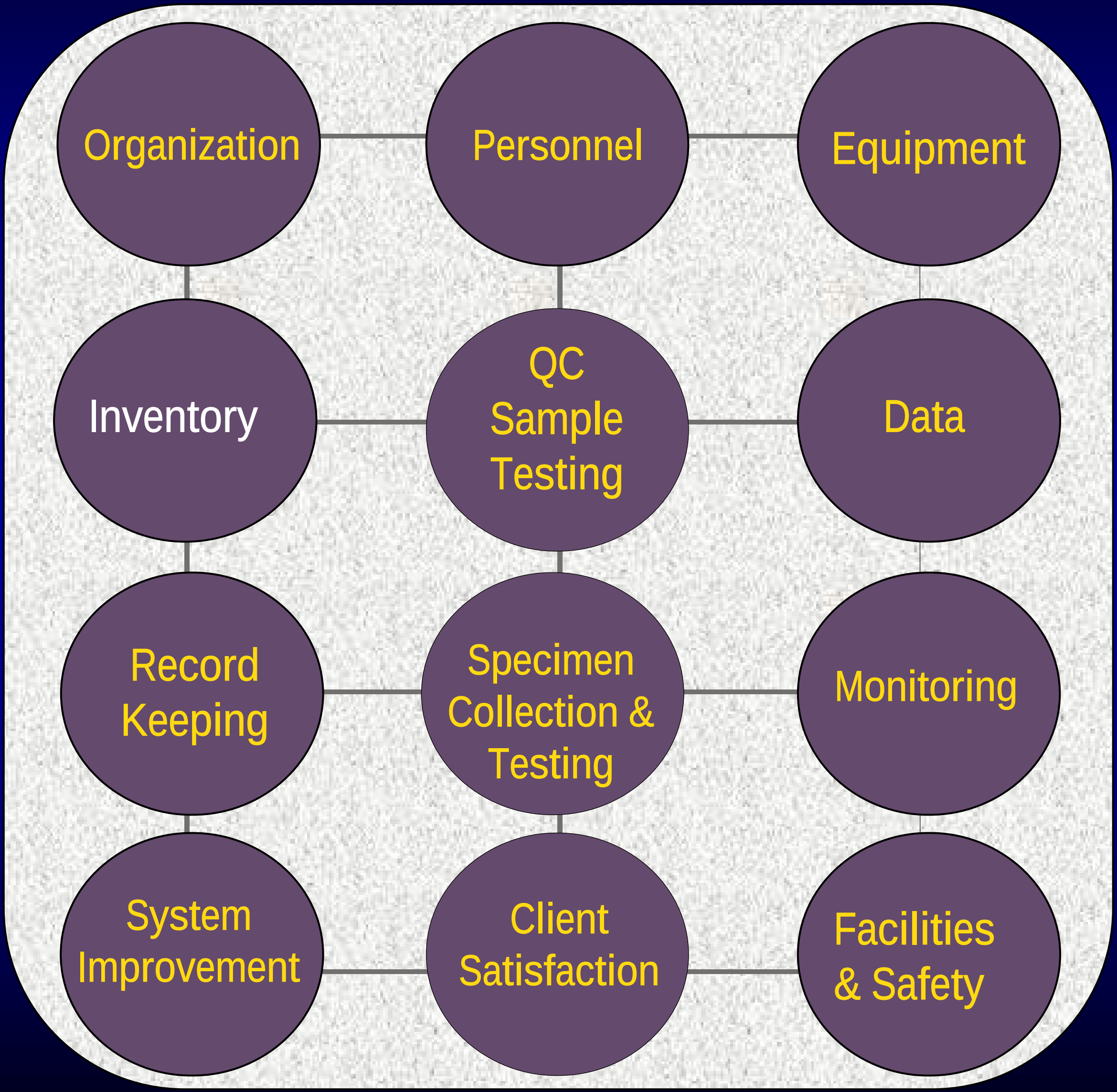

- Lab workers
- Health workers
- Counselors

Learning Objectives

At the end of this module, you will be able to:

- Maintain proper records
- Maintain proper level of consumables
- Use first-expiry-first-out concept when managing stock – Is “first in - first out” better?
- Inspect delivery of supplies before acceptance
- Identify lot numbers and expiry dates
- Store kits and supplies properly

Content Overview

What is stock management?

- Keeping record
- Re-ordering as needed
- Receiving consumables
- Storing consumables

Stock Management Means...

Properly maintaining adequate supplies to provide uninterrupted service

Stock at Rapid Test Site Includes…

Inventory in Storage Area

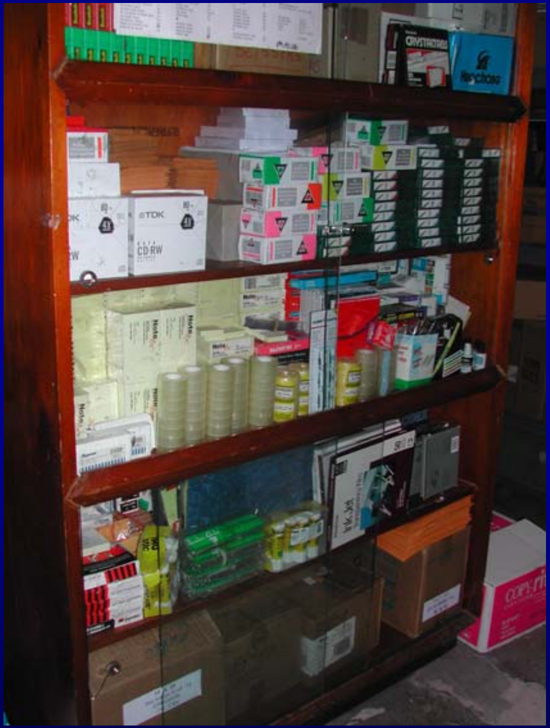

and

Supplies at Workstation

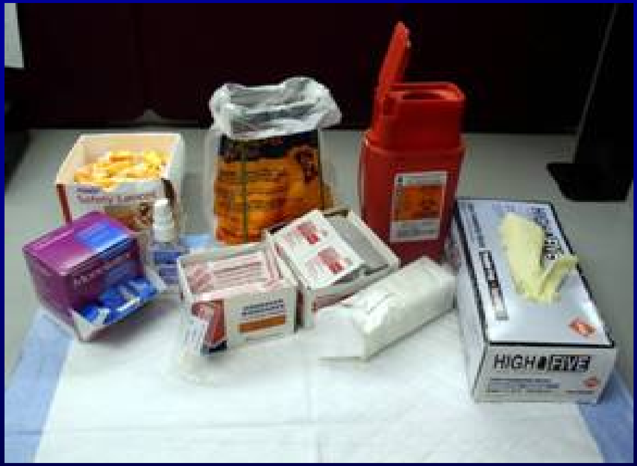

Lab workers Health workers Counselors

Stock Management Leads to High Quality Testing

- Guarantees availability of supplies
- Reduces costs
- Avoids the use of expired kits
- Reduces waste

How Do You Manage Stock?

You own a doubles cart. Your wife prepares the doubles you sell them for a profit. To make money, you must always have what your customers want.

What must you do to maintain adequate stocks?

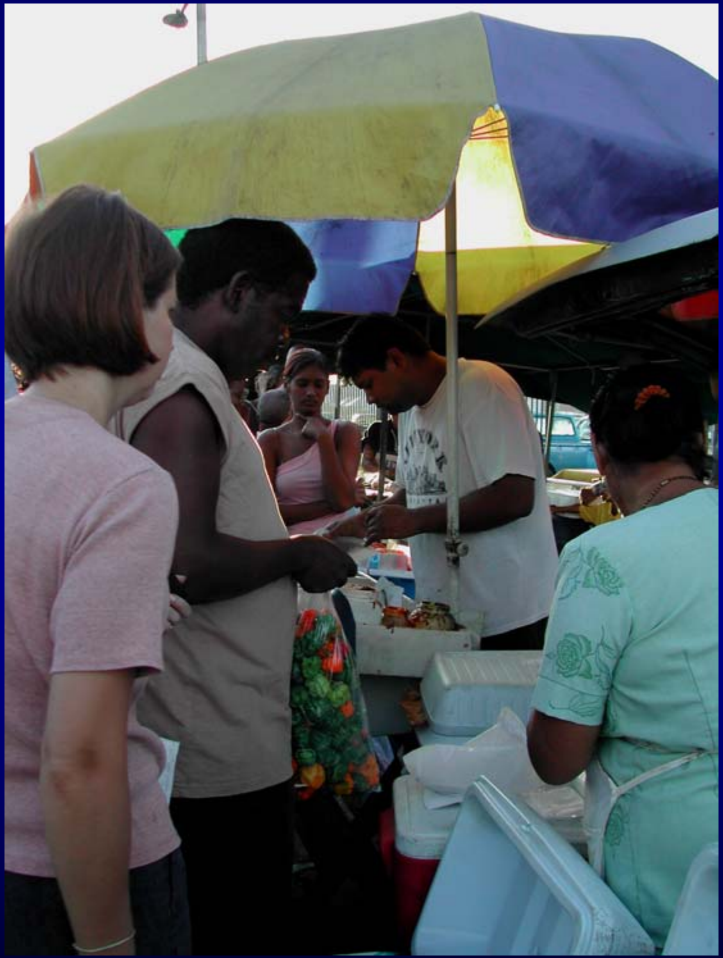

Stock Management Involves Knowing...

- What items you have.
- How many of each item you have.
- When to order.
- What you have ordered.
- How long it has been since you ordered.
- Where to store your stock.
- When new stock was received.
- Where new stock is located
- Who received the new stock.

Stock Management Involves...

- Performing a “stock count”
- Maintaining proper inventory records
- Determining when to re-order
- Determining how much to re-order
- Placing orders properly
- Inspecting delivery of new orders
- Storing stock properly

Perform a “Stock Count”

**What is it?**	Counting each item in the stock
**When is it done?**	Recommended at the beginning of each month
**Who does it?**	A designated person

All items must be accounted for. Everything that comes in and goes out must be recorded.

Maintain Proper Inventory Records

Stock Card

- Simple, heavy weight cards
- Kept for each item in stock

Stock Book

- Contains listing of all items in the store
- Is updated after physical count
- Uses information from stock cards
- Is also called a Stock Register

Stock Card: An Example

- Item Name: ____________ Unit: _____________
- Manufacturer: _____________________________
- Minimum Stock (Re-Order Level): _____________

Date	Received From	Issued to	Quantity Received	Quantity Issued	*Balance	Lot #	Signature

Stock Book: An Example

Item Name	Qty (units) Requested	Date Requested	Qty Received	Date Received	Lot #	Expiry Date

Reconciling Stock with Records

Ideal # Tests Performed = Stock Depletion

Reality # Test Performed + loss = Stock Depletion

What can be done to minimize loss?

Decide When to Re-order

Re-order when stock reaches minimum level

Terminology:

- **Minimum stock** - Amount of stock required to support testing operations until additional supplies are received
- **Lead time** – Time between placing an order and receiving it
- **Maximum usage** – number of test kits used in a given time period

Calculating Minimum Stock Level

Minimum Stock Level = Maximum lead time in weeks X Maximum Usage

Example:

Maximum lead time = 12 weeks

Maximum usage/wk = 3 kits

Minimum stock level = 12 x 3 = 36 kits

When only 36 kits are left, place an order

Exercise: Calculate Minimum Stock Level

Minimum Stock Level = Maximum lead time in weeks X Maximum Usage

- On average, you use 5 Uni-Gold kits a week.
- Usually you need 12 weeks to receive your orders.
- You should order more Uni-Gold kits when you have _____ kits left in the inventory.

Decide How Much to Re-order

Establish proper full stock level. Re-order to reach that level.

- **Review stock "use". Include stock consumed, borrowed, expired, wasted, and pilfered.**
- **Never order more than you can store.**
- **Never order more than you can use before expiry.**
- **Know maximum usage AND minimum stock level**

Decide Full Stock Level

Maintain stocks that cover
maximum usage plus minimum stock level

Maximum usage /wk = 70 tests<br># tests / month = 70 x 4<br><br>280 tests used per month<br><br>Assuming 20 tests per kit, how many kits are used per month?<br>280 /20 = 14	Lead time = 12 weeks (3 months)<br>Minimum stock level = 14 kits x 3 months = 42 kits<br>Full stock level = 42+14 = 56<br><br>You must have 56 kits in stock at the beginning of each re-order cycle.

Exercise: Decide Full Stock Level

Maximum usage /wk = 80 tests<br><br>How many tests are used per month?<br>__________ tests/month<br><br>Assuming 15 tests per kit, how many kits are used per month?<br><br>__________ kits/month	Lead time = 12 weeks or 3 months<br>Minimum stock level = ______ kits<br><br>Full stock level = _______ kits<br><br>You must always have ______ kits in stock at the beginning of each re-order cycle (3 months).

Calculate:

- What is the minimum stock level?
- How many test kits should you have in the beginning of each re-order cycle?

Place Orders Properly

- Describe current ordering system
- Provide instructions for placing orders
- Instruct trainees to complete forms needed for inventory/stock management.
- Describe contingency plan for stock shortages
- Describe communications to suppliers
  - Learn how to stop standing deliveries
  - Know how to request delivery
  - Know what information to tell central procurement

Inspect Delivery of New Orders

Upon receipt:

- Verify contents of order received with requisition
- Check integrity of received supplies
- Date each item received
- Note expiration date
- Store new shipment behind existing shipment
- Create or update records

Examine Lot Number & Expiry Date

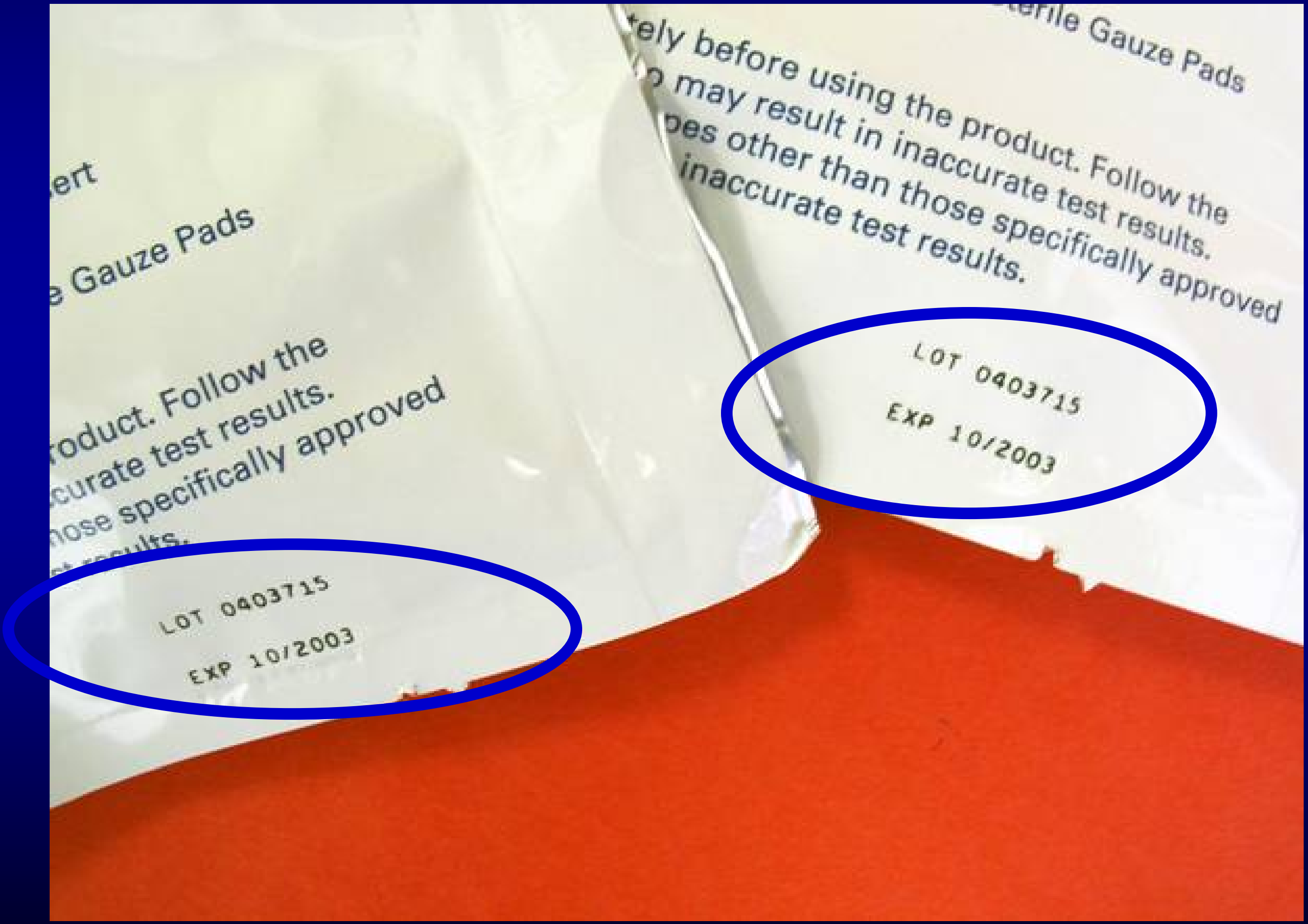

Lab workers Health workers Counselors

Proper Storage of Stock

- Store stock in clean, organized, and locked storeroom
- Store in well-ventilated space away from direct sunlight
- Store according to manufacturer's instructions
- Record storeroom temperature daily
- Place items on shelves
- Organize existing and new shipments by expiration dates

First expiry, first out

Summary

- What does inventory management mean?
- What information is recorded in inventory record-keeping?
- How do you decide minimum stock level?
- How do you decide proper full inventory level?
- What does "First Expiry First Out" mean?
- What procedure should you follow when receiving new kits and supplies?
- How should kits and supplies be stored?

Key Messages

- Maintain adequate inventory at all times.
- Do not run out of anything before re-order.
- Never order more than you can store.
- Never order more than you can use before expiry.
- NOTIFY OVERAGE-redistribute before expiry.
- Account for all items in inventory.
- Inspect new shipments before acceptance.

Module 11: Refrigerators

QC Sample Storage at HIV Testing Sites

Quality HIV Testing System

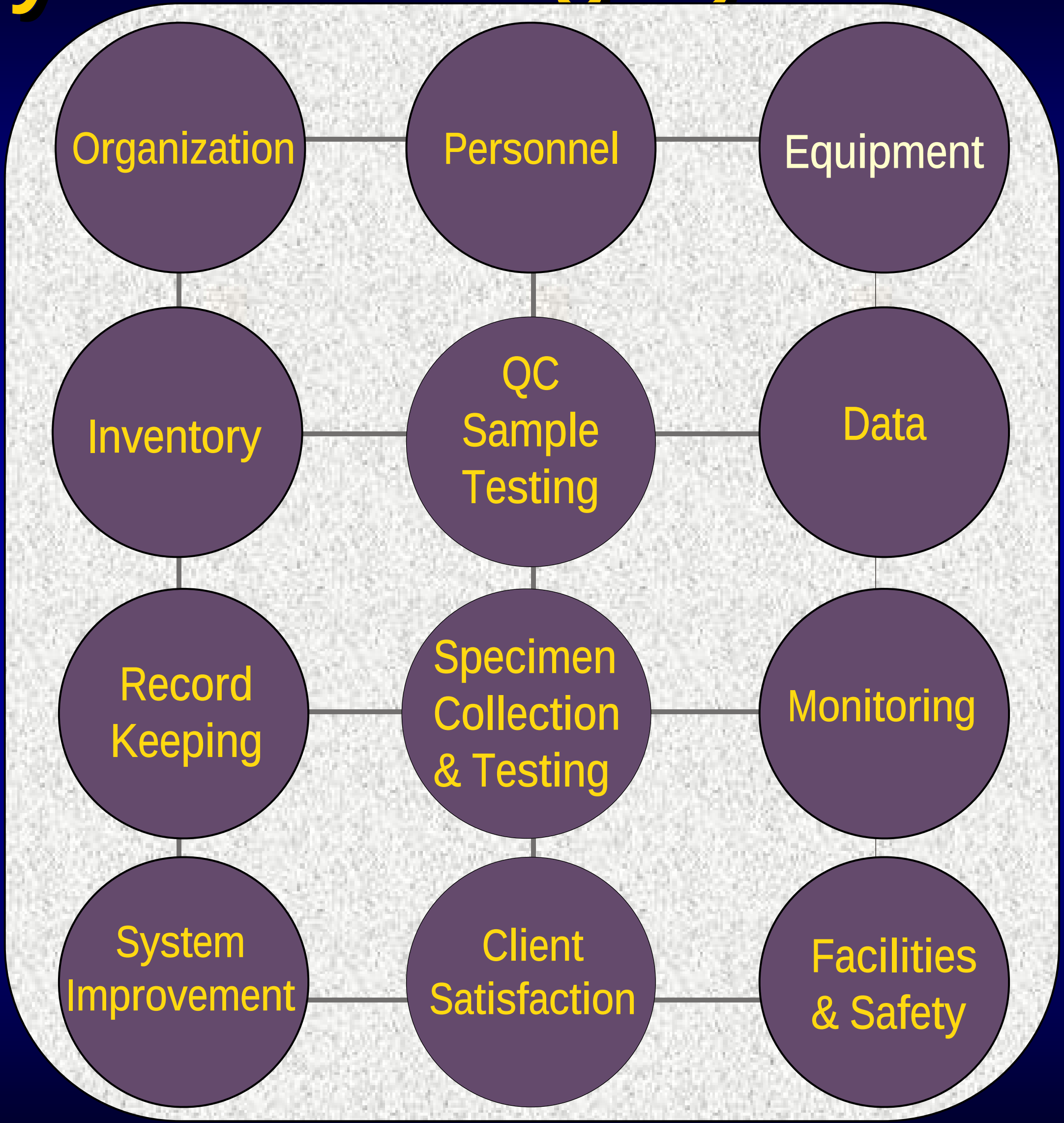

Lab workers **Health workers** **Counselors**

Learning Objectives

At the end of this module, you will be able to:

- Monitor temperatures
- Keep temperature records

Content Overview

- Rationale for equipment maintenance
- Your responsibilities for equipment
- Refrigerators at HIV rapid testing sites

Functioning Equipment is Vital to Quality Service

Functioning Equipment

Produces reliable test results

Lowers repair costs

Prevents delays in testing

Maintains productivity

Total Quality

Equipment at HIV Rapid Testing Sites

Refrigerator

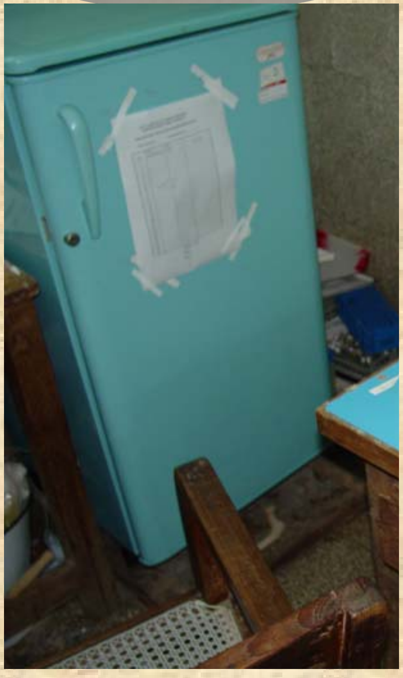

◆ Lab workers ◆ Health workers ◆ Counselors

Temperature Log

Manufacturer_______________
Model No__________________

Year ___________

QS Sample Refrigerator
Temperature Record
Range (2 to 8 ^{o}C)

	1	2	3	4	5	6	7	8	9	10	11	12	13	14	15	16	17	18	19	20	21	22	23	24	25	26	27	28	29	30	31
Jan																															
Feb																															
Mar																															
Apr																															
May																															
Jun																															
Jul																															
Aug																															
Sep																															
Oct																															
Nov																															
Dec																															

Responsibilities at Test Site

- Record refrigerator temperatures (2°C to 8°C)
- Record storage area temperature (28°C or less)
- Record Testing room temperature (28°C or less)
- Report incorrect temperatures to your supervisor
- Take corrective actions
- Keep records for each day of testing

Stop testing if refrigerator fails!

Refrigerator Care

- Keep clean
- Close completely
- Report temperatures above 8°C

  or below 2°C
- DO NOT store food or beverages in the QC sample refrigerator

Summary

- Why keep temperature records?
- What is the proper temperature for the QC sample refrigerator?
- Why not store food in the QC refrigerator?
- What is the maximal permitted temperature for the storage area?
- What should you do when you record incorrect temperatures?

Module 12: Quality Control

Content Overview

- What is Quality Control (QC)?
- Benefits of QC in rapid testing
- Internal versus external quality control
- Troubleshooting invalid results
- Quality control records

What is Quality?

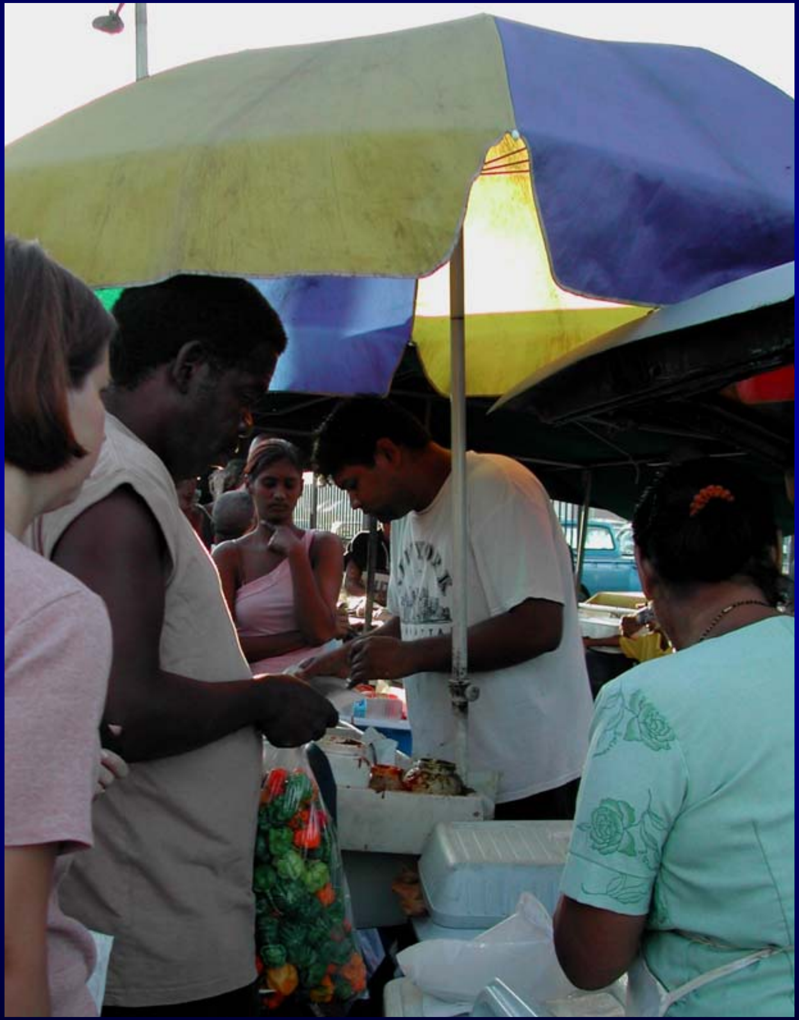

What Is Quality Control (QC)?

- Monitoring the quality of the test itself
- Reporting results that are correct

Controls for MOH TT HIV Rapid Testing

- Internal
  - Controls within the testing device
- External
  - Controls of *known* HIV status provided by an external source.

Internal and External Quality Control

Internal Control

Included in testing device

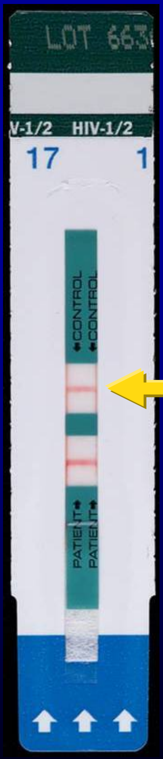

Control Band

External Control

Known positive and negative samples used to check the testing process

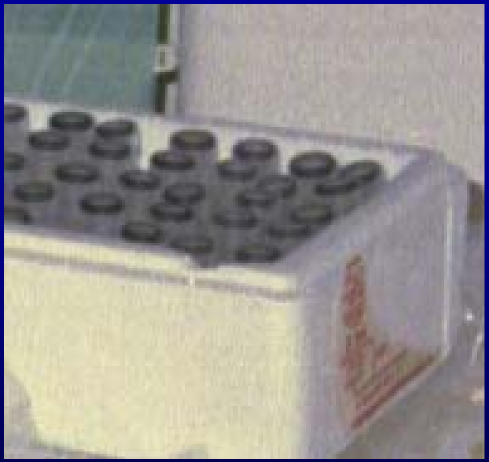

Lab workers Health workers Counselors

External Quality Control Samples

Prepared by Reference Laboratory

Commercially prepared

- Store according to instructions
- Date when opened
- Use before expiry date
- Do not contaminate

Use of External Control Samples in the MOH TT Testing System

- Each tester must test one positive and one negative control with each kit (Determine, Unigold and Stat-Pak):
  - At the start of each day of testing
  - At the beginning of a new lot number

Quality Control Record: An Example

QC SAMPLE LOG

Date and Time	Tester	QC samples Lot #	Determine®			Uni-Gold™			Stat-Pak		
			Lot #	Ex Date	Result	Lot #	Ex Date	Result	Lot #	Ex Date	Result
		Pos									
		Neg									
		Pos									
		Neg									
		Pos									
		Neg									
		Pos									
		Neg									
		Pos									
		Neg									
		Pos									
		Neg									
		Pos									
		Neg									
		Pos									
		Neg									
		Pos									
		Neg									
		Pos									
		Neg									

QC Invalid Results – What Do You Do?

- Repeat test
- If the second test is invalid, STOP testing for the day.
- Identify cause of problem
- Inform manager
- Take corrective actions

Troubleshooting Invalid Results

Problem	Potential Cause	Action
No control line or band present	• Damaged test device or controls	• Repeat the test using new device
	• Proper procedure not followed	• Follow each step of testing according to SOP<br>• Re-check buffer and/or specimen volumes
	• Expired or improperly stored test kits or controls	• Check expiry date of kits and QC samples. Do not use beyond stated expiry date<br>• Check temperature records for storage and testing area

◆ Lab workers ◆ Health workers ◆ Counselors

Troubleshooting Invalid Results – Cont'd

Problem	Potential Cause	Action
Positive reaction with negative QC sample, i.e. false positive	Mixed up samples	Re-test negative control using a new device and read results within specified time limit
Extremely faint control line	The control line can vary in intensity	No action required. Any visible line validates the results.

Exercise #1: Interpreting Rapid Test Results

- Read the test results from the teaching aids we have just handed out.
- Write your interpretation on the index card provided.

Review of Quality Control

- Review of internal control results before accepting test results
- Review of external control results by tester
- Regular review of QC sample logs and other records by the Quality Monitor (site audits)

Module 13: Quality Monitoring

On-site Review and Auditing

Content Overview

- What is quality monitoring?
- Why is it important?
- Who will provide quality monitoring for MOH TT “same visit” HIV testing?
- Test site records
- Internal and external audits
- Verification panels

Review of Quality Control

- Review of internal control results before accepting test results
- Review of external control results by tester
- Regular review of QC sample logs and other records by the Quality Monitor (site audits)

Quality Monitoring Definition

Objective review of testing by an external agency or personnel

Why Quality Monitoring?

- To compare performance and results among test sites
- To provide early warning for systematic problems in testing
- To evaluate quality of testing
- To stop testing when appropriate

Monitoring Where Testers are Counselors

- Client confidentiality is top priority
- Monitoring must be indirect
- Internal auditing is by tester
- Site auditing is by quality monitors
- Quality monitors will provide feedback *during* the site audit
- Quality monitors have authority to stop testing

MOH TT Monitoring Components

Temperature Records

QC Sample Log

Testing Records

Verification Panels

Supervisors or Managers   Testers

What is a Site Audit?

A site audit is review of the testing process to collect information needed for process improvement.

Site audits may be:

- Internal - conducted by the person who is performing testing
- External – conducted by the Quality Monitor

Components of Site Audit

- Review temperature records
- Review QC sample log
- Review client test result records
- Verify inventory control records
- Observe cleanliness of testing site
- Observe cleanliness of QC refrigerator
- Request tester to complete verification panel (external audit only)
- Discuss review observations with tester

Management Responsibilities:

- Establish quality monitoring policy
- Assign responsibilities
- Respect Quality Monitor and/or Tester decisions about testing
- Correct deficiencies
- Communicate outcomes to all involved

Summary

- Quality Monitors are responsible for site audits
- Quality Monitors have authority to stop testing
- Effective Site Managers will respect and support decisions made by the Tester and the Quality Monitor

Module 15:
Documents and Records

Learning to "Think like a lab person" (part 2)

Learning Objectives

At the end of this module, you will be able to:

- Explain differences between documents and records
- Explain the relationship between records and quality of testing
- List documents and records for quality testing at MOH TT testing sites
- Follow standard operating procedures (SOPs)
- Keep MOH TT testing site records accurately

Content Overview

- What are documents and records?
- Documents
  - Why are they important?
  - What documents should you keep?
  - Why follow SOPs?
  - What is the proper way to keep and maintain documents?
- Records
  - Why are they important?
  - What records should you keep?
  - What is the proper way to keep and maintain records?

T&T Quality Health Care System

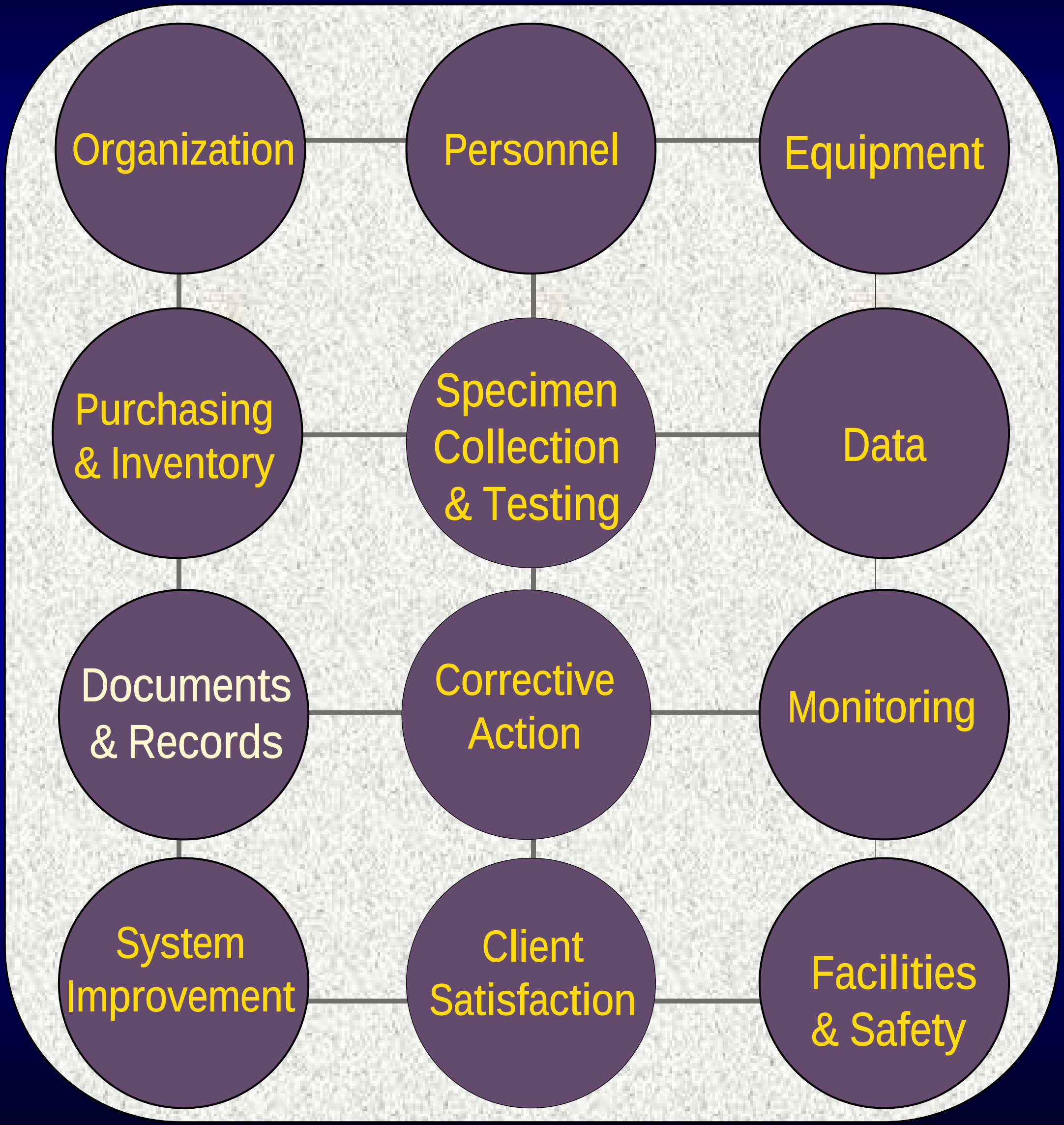

Lab workers Health workers Counselors

What Are Documents and Records?

Documents

- WRITTEN policies, process descriptions, procedures, and blank forms used to communicate information

Records

- Information on worksheets, forms, and charts (collected on documents)

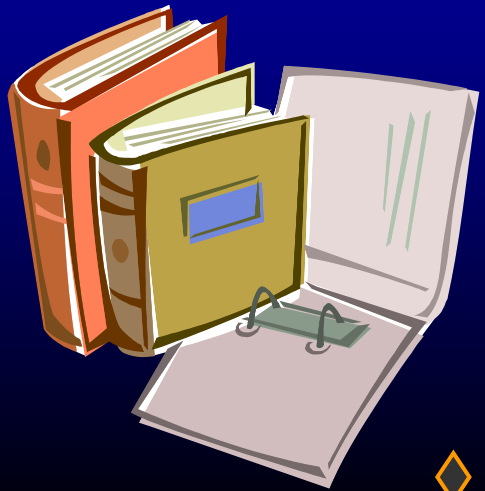

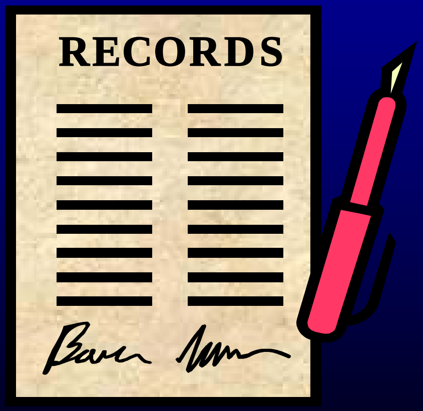

Lab workers Health workers Counselors

Which Is Which?
Documents and Records

- MOH TT testing algorithm
- Reference manual
- Temperature logs (completed)
- SOPs for individual HIV rapid tests
- Manufacturer product inserts
- Temperature logs (blank)
- QC sample log (blank)
- Inventory cards (completed)
- Client record (completed)
- Client record (blank)
- Summary of findings from a site audit

Which Is Which?

Documents and Records

- MOH TT testing algorithm
- Reference manual
- Temperature logs (completed)
- SOPs for individual HIV rapid tests
- Manufacturer product inserts
- Temperature logs (blank)
- Inventory cards (completed)
- QC sample log (blank)
- Client record (completed)
- Client record (blank)
- Summary of findings from a site audit

Lab workers Health workers Counselors

Documents Are the Backbone of the Quality System

Verbal instructions often are:

- Not heard
- Misunderstood
- Quickly forgotten
- Ignored

Policies, standards, processes, and procedures must be written down, approved, and communicated to all concerned.

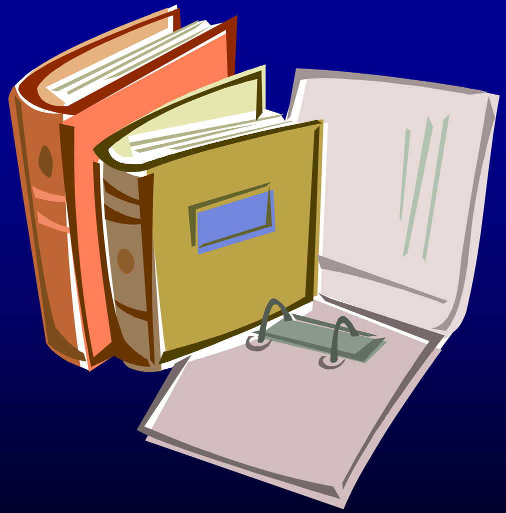

Standard Operating Procedures (SOPs) Are Documents that...

- Describe how to perform various operations in a testing site
- Provide step-by-step instructions
- Assure:
  - Reliability
  - Accuracy
  - Quality

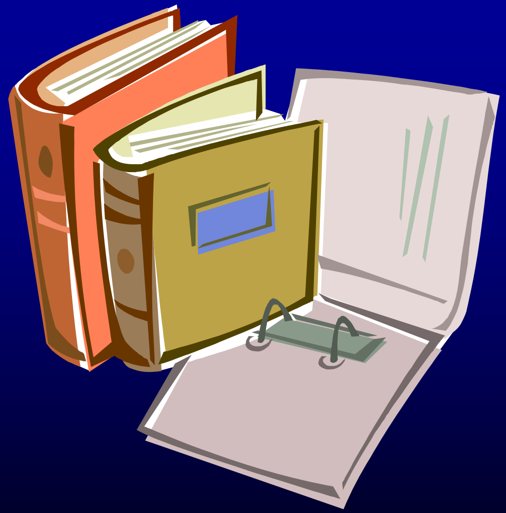

Use Test Site SOPs

- Manufacturer product inserts are not sufficient for testing.
- Test site SOPs include:
  - Materials required, but not in kit
  - Specific safety requirements
  - Sequence of tests for MOH TT testing
  - QC Samples testing procedure

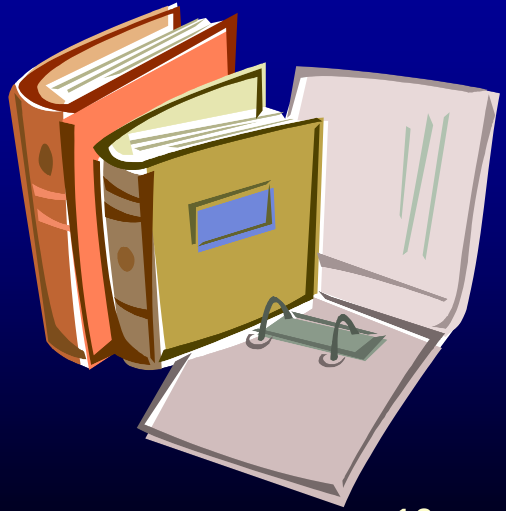

SOPs Must Be Followed

- Why is it important to follow SOPs?
- What happens if you do not?

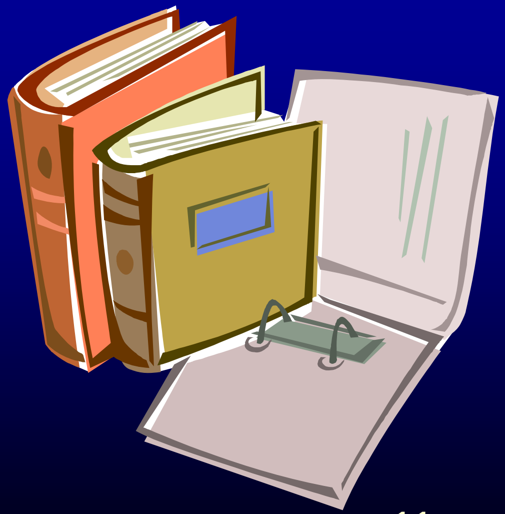

Lab workers Health workers Counselors

MOH TT Documents

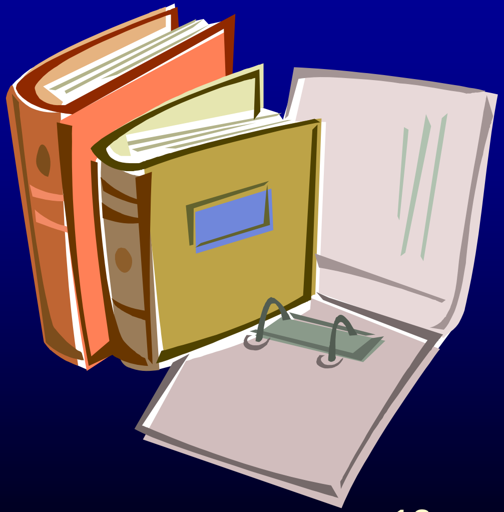

What Documents Are Needed at MOH TT Testing Sites?

- MOH TT Testing Algorithm
- Temperature Logs
- Finger-stick blood draw procedure
- QC Sample Log
- Determine SOP
- Uni-Gold SOP
- Stat-Pak SOP
- HIV Test Report
- Inventory Cards

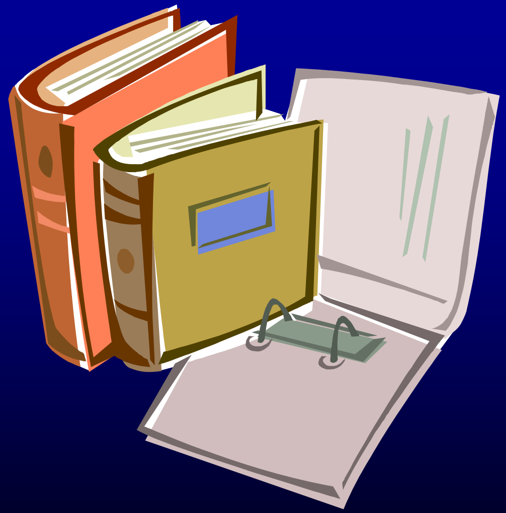

MOH TT Temperature Logs

Three Temperature Logs are needed at each MOH TT testing site:

- QC Sample Refrigerator
- Rapid Test Kit Storage Area
- Testing Area

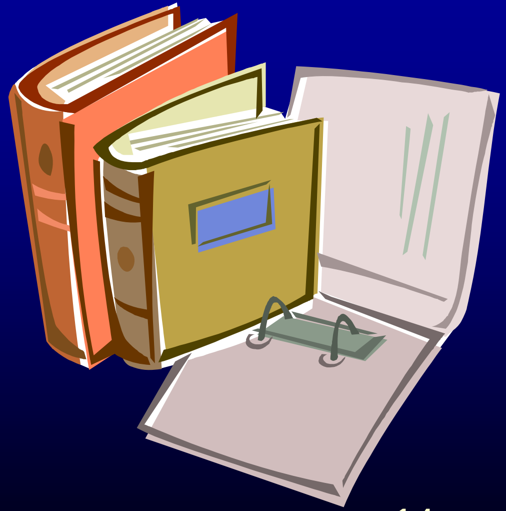

Lab workers Health workers Counselors

MOH TT Temperature Logs

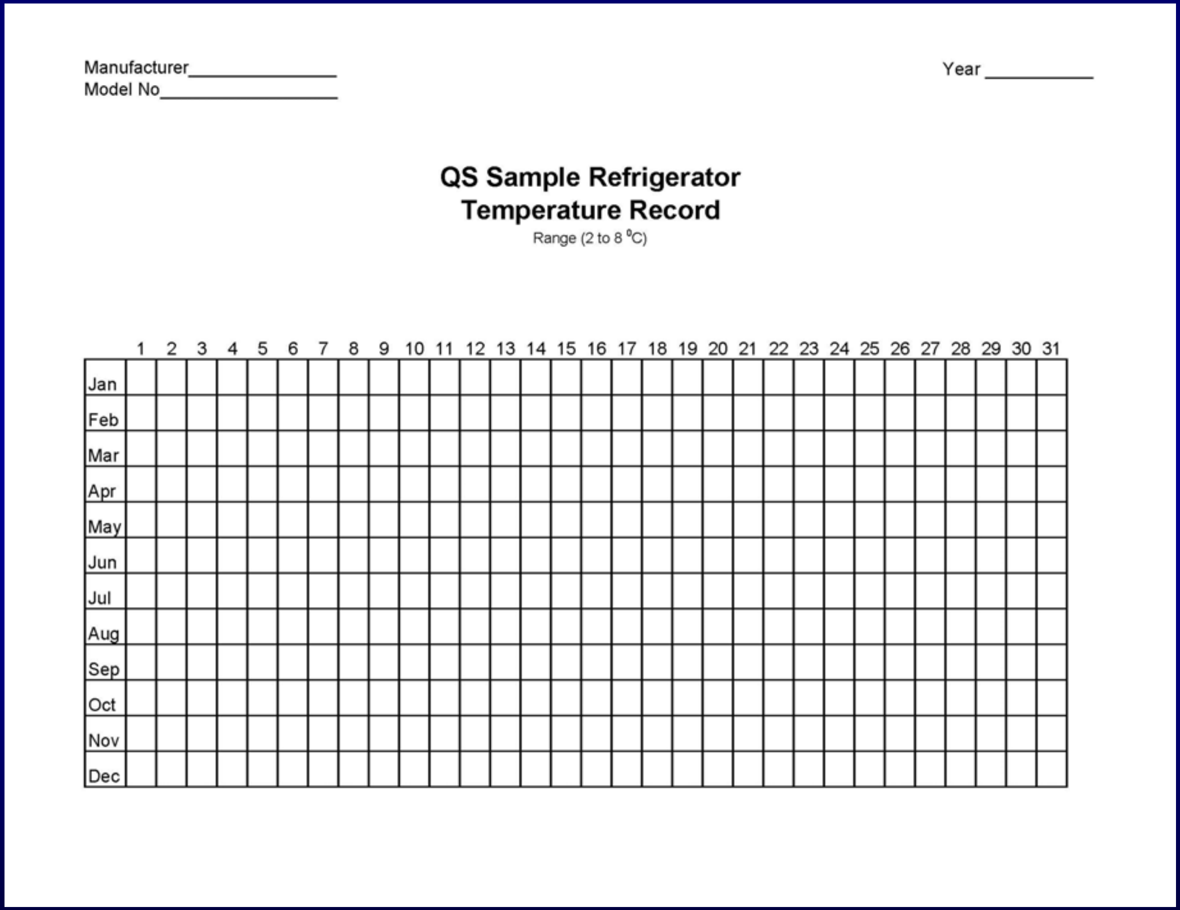

Manufacturer_______________ Year __________
Model No_______________

QS Sample Refrigerator Temperature Record

Range (2 to 8 ^{0}C)

	1	2	3	4	5	6	7	8	9	10	11	12	13	14	15	16	17	18	19	20	21	22	23	24	25	26	27	28	29	30	31
Jan																															
Feb																															
Mar																															
Apr																															
May																															
Jun																															
Jul																															
Aug																															
Sep																															
Oct																															
Nov																															
Dec																															

- Fill accurately
- Write legibly
- Date

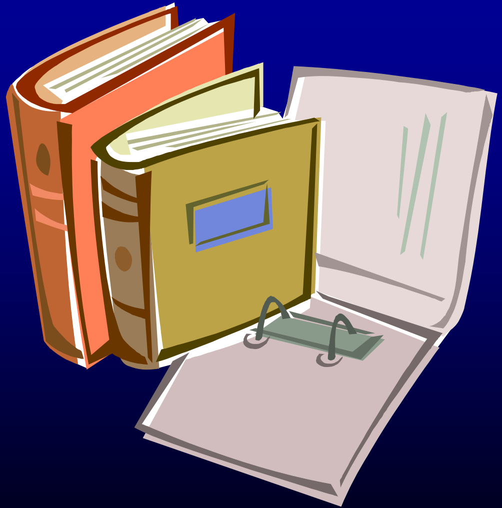

MOH TT Temperature Logs

Year ___________

Kit Storage
Temperature Record
(less than 28 ^{0}C)

	1	2	3	4	5	6	7	8	9	10	11	12	13	14	15	16	17	18	19	20	21	22	23	24	25	26	27	28	29	30	31
Jan																															
Feb																															
Mar																															
Apr																															
May																															
Jun																															
Jul																															
Aug																															
Sep																															
Oct																															
Nov																															
Dec																															

- Fill accurately
- Write legibly
- Date

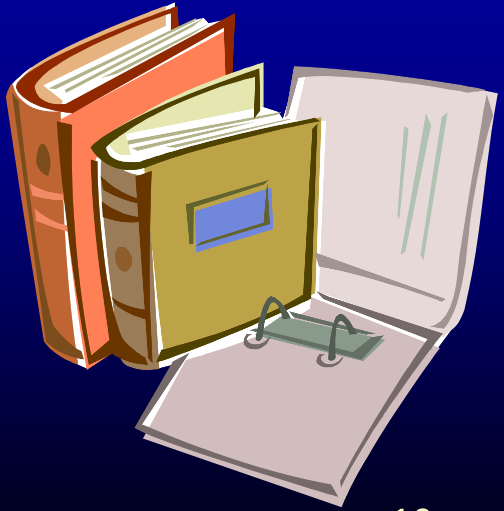

MOH TT Temperature Logs

Year __________

Testing Area
Temperature Record
(less than 28 °C)

	1	2	3	4	5	6	7	8	9	10	11	12	13	14	15	16	17	18	19	20	21	22	23	24	25	26	27	28	29	30	31
Jan																															
Feb																															
Mar																															
Apr																															
May																															
Jun																															
Jul																															
Aug																															
Sep																															
Oct																															
Nov																															
Dec																															

- Fill accurately
- Write legibly
- Date

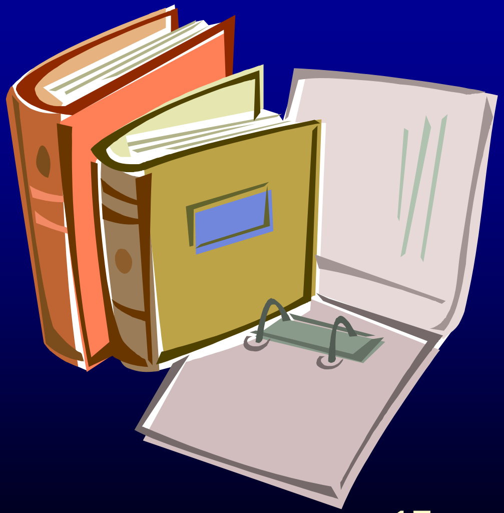

MOH TT Blood Collection Procedure

Each client will be asked to provide a sample of blood for testing.

Each blood sample should be collected the same way.

Collection should follow the Finger-Stick Blood Draw SOP

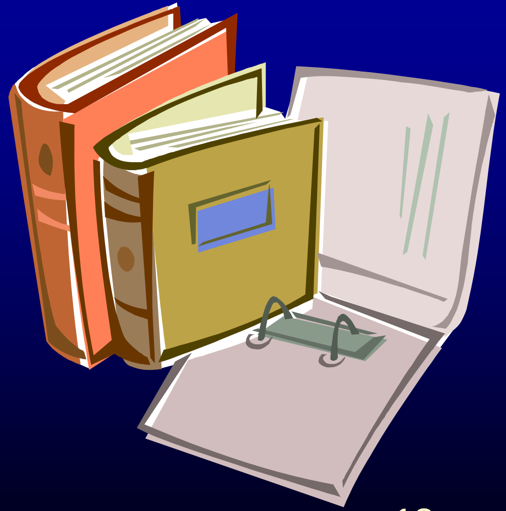

Lab workers Health workers Counselors

MOH TT Blood Collection Procedure

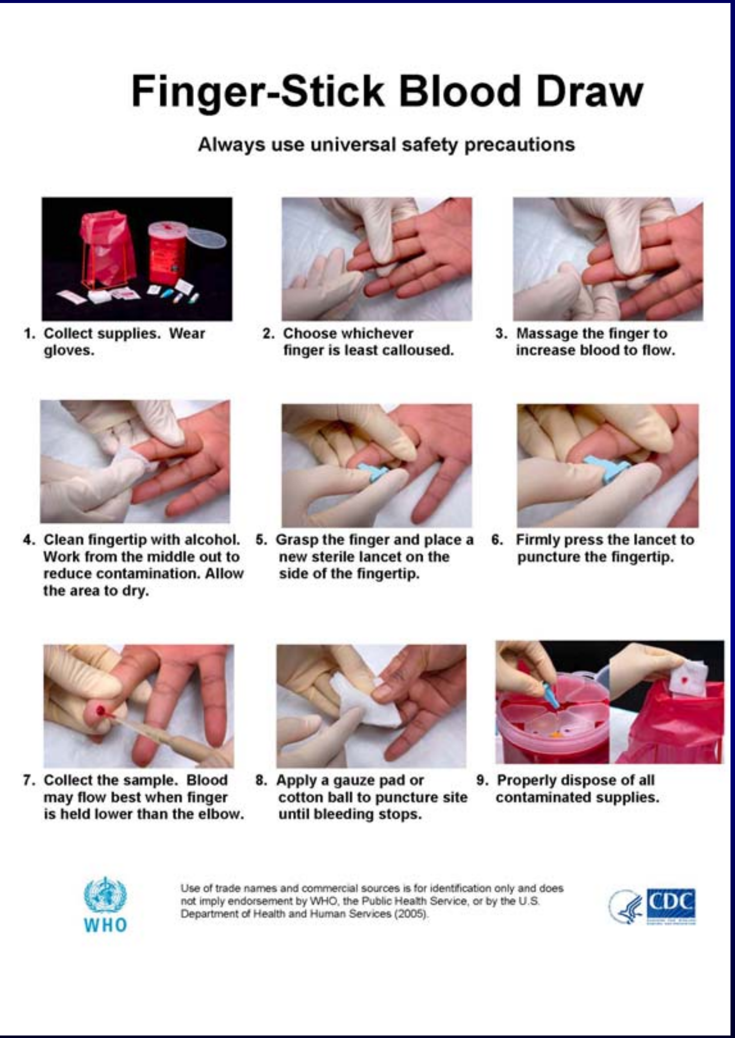

Blood collection from each client should follow the Finger Stick Blood Draw SOP

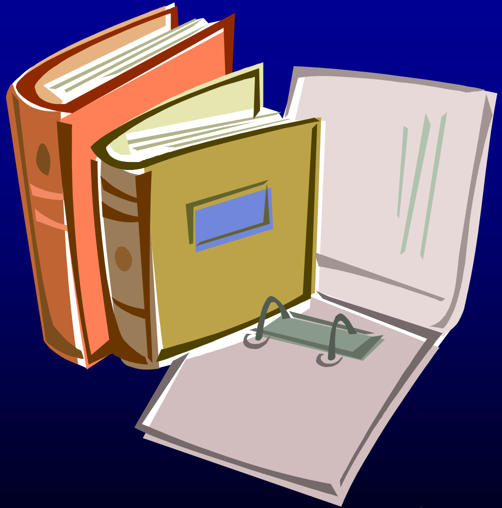

Lab workers | Health workers | Counselors

MOH TT QC Sample Log

QC SAMPLE LOG

Date and Time	Tester	QC samples Lot #	Determine®			Uni-Gold™			Stat-Pak		
			Lot #	Ex Date	Result	Lot #	Ex Date	Result	Lot #	Ex Date	Result
		Pos									
		Neg									
		Pos									
		Neg									
		Pos									
		Neg									
		Pos									
		Neg									
		Pos									
		Neg									
		Pos									
		Neg									
		Pos									
		Neg									
		Pos									
		Neg									
		Pos									
		Neg									
		Pos									
		Neg									

- Fill accurately
- Write legibly
- Date
- Sign

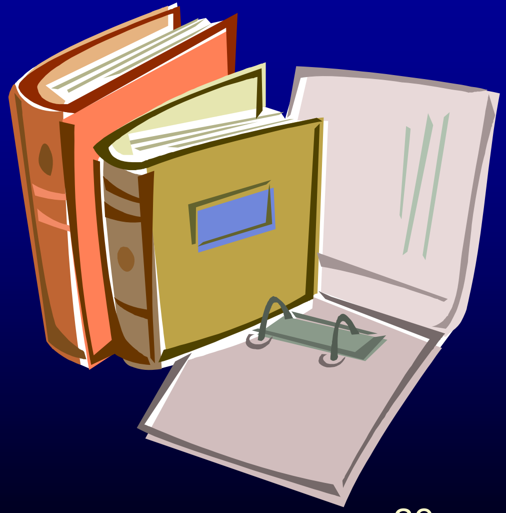

MOH TT Rapid Test SOPs

Three HIV Rapid Test SOPs are needed at each MOH TT testing site:

- Determine
- Uni-Gold
- Stat-Pak

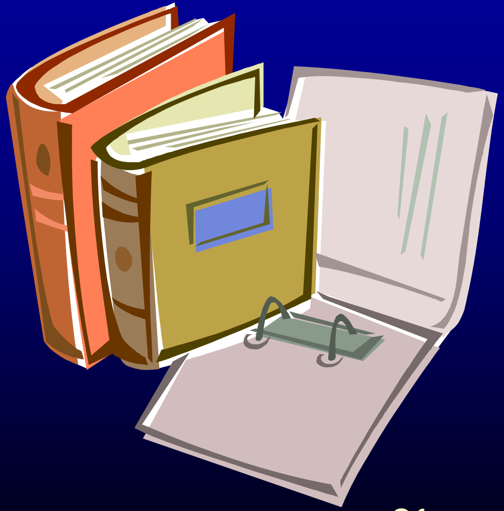

MOH TT SOP: Determine Test

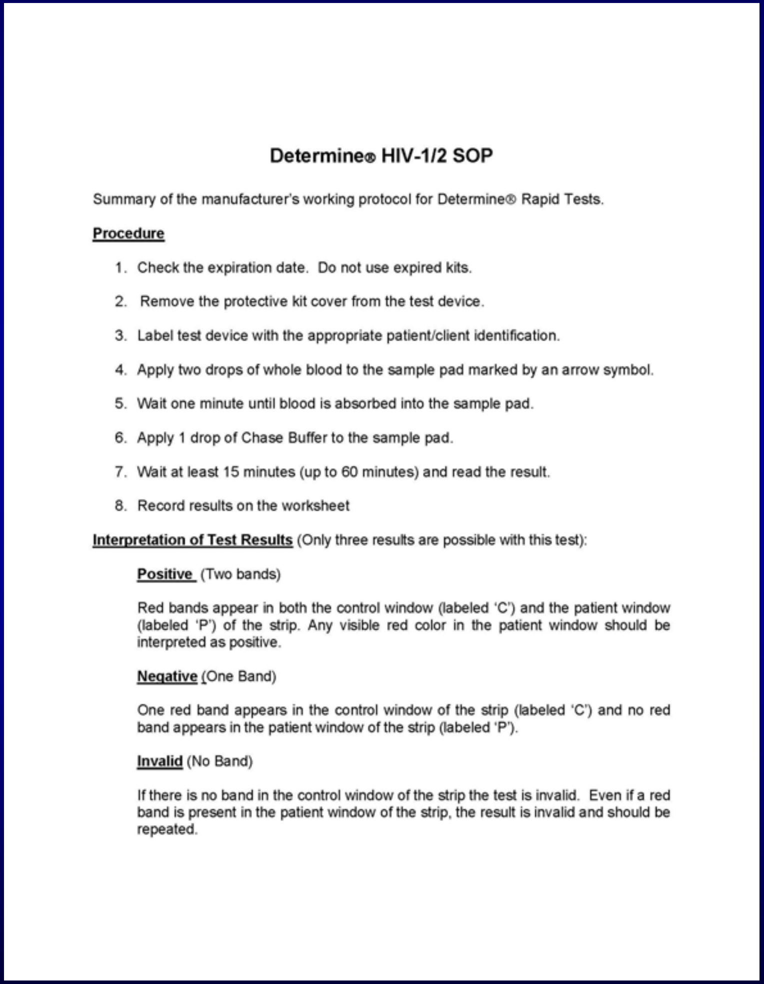

Determine® HIV-1/2 SOP

Summary of the manufacturer's working protocol for Determine® Rapid Tests.

Procedure

1. Check the expiration date. Do not use expired kits.
2. Remove the protective kit cover from the test device.
3. Label test device with the appropriate patient/client identification.
4. Apply two drops of whole blood to the sample pad marked by an arrow symbol.
5. Wait one minute until blood is absorbed into the sample pad.
6. Apply 1 drop of Chase Buffer to the sample pad.
7. Wait at least 15 minutes (up to 60 minutes) and read the result.
8. Record results on the worksheet

Interpretation of Test Results (Only three results are possible with this test):

Positive (Two bands)

Red bands appear in both the control window (labeled 'C') and the patient window (labeled 'P') of the strip. Any visible red color in the patient window should be interpreted as positive.

Negative (One Band)

One red band appears in the control window of the strip (labeled 'C') and no red band appears in the patient window of the strip (labeled 'P').

Invalid (No Band)

If there is no band in the control window of the strip the test is invalid. Even if a red band is present in the patient window of the strip, the result is invalid and should be repeated.

- Approved for use in-country
- Must be kept up-to-date
- Must be followed precisely

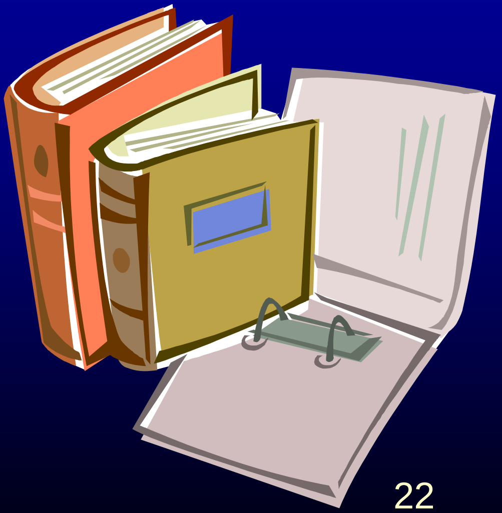

Lab workers   Health workers   Counselors

MOH TT SOP: Uni-Gold Test

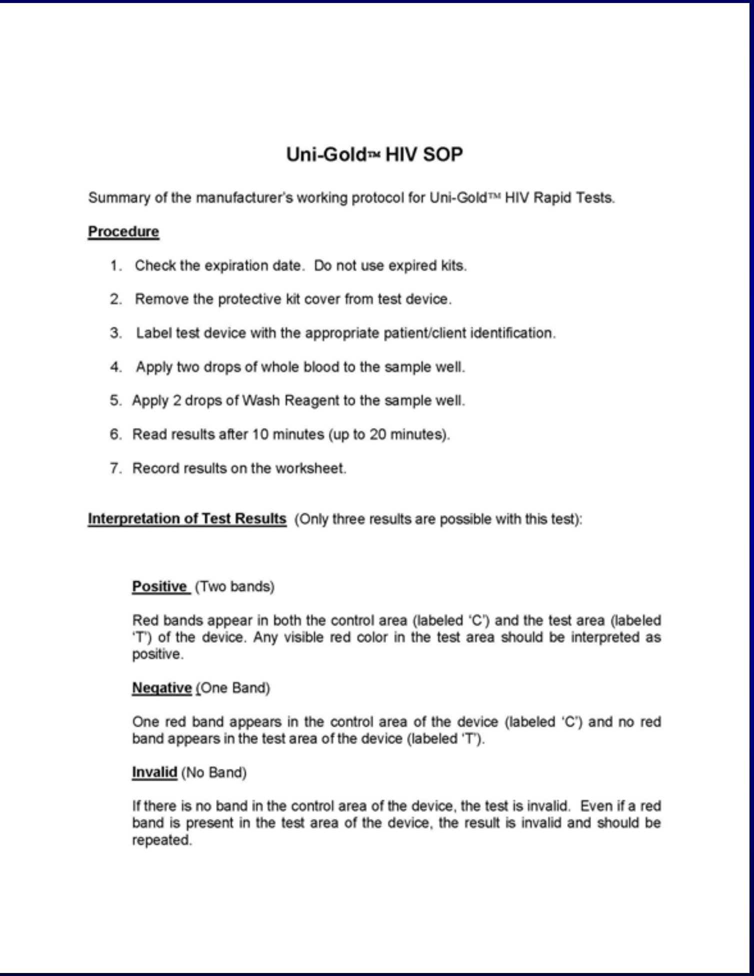

Uni-Gold™ HIV SOP

Summary of the manufacturer's working protocol for Uni-Gold™ HIV Rapid Tests.

Procedure

1. Check the expiration date. Do not use expired kits.
2. Remove the protective kit cover from test device.
3. Label test device with the appropriate patient/client identification.
4. Apply two drops of whole blood to the sample well.
5. Apply 2 drops of Wash Reagent to the sample well.
6. Read results after 10 minutes (up to 20 minutes).
7. Record results on the worksheet.

Interpretation of Test Results (Only three results are possible with this test):

Positive (Two bands)

Red bands appear in both the control area (labeled 'C') and the test area (labeled 'T') of the device. Any visible red color in the test area should be interpreted as positive.

Negative (One Band)

One red band appears in the control area of the device (labeled 'C') and no red band appears in the test area of the device (labeled 'T').

Invalid (No Band)

If there is no band in the control area of the device, the test is invalid. Even if a red band is present in the test area of the device, the result is invalid and should be repeated.

- Approved for use in-country
- Must be kept up-to-date
- Must be followed precisely

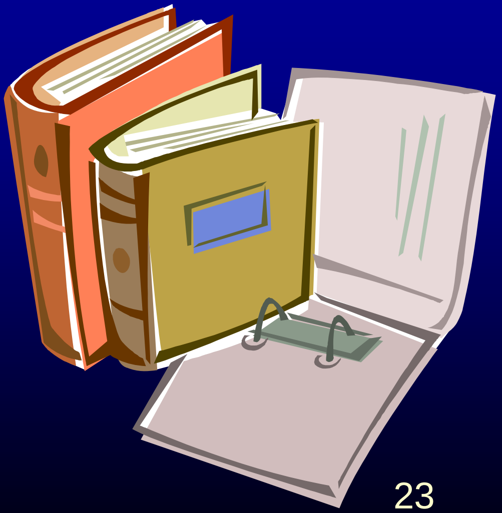

Lab workers   Health workers   Counselors

MOH TT SOP: Stat-Pak Test

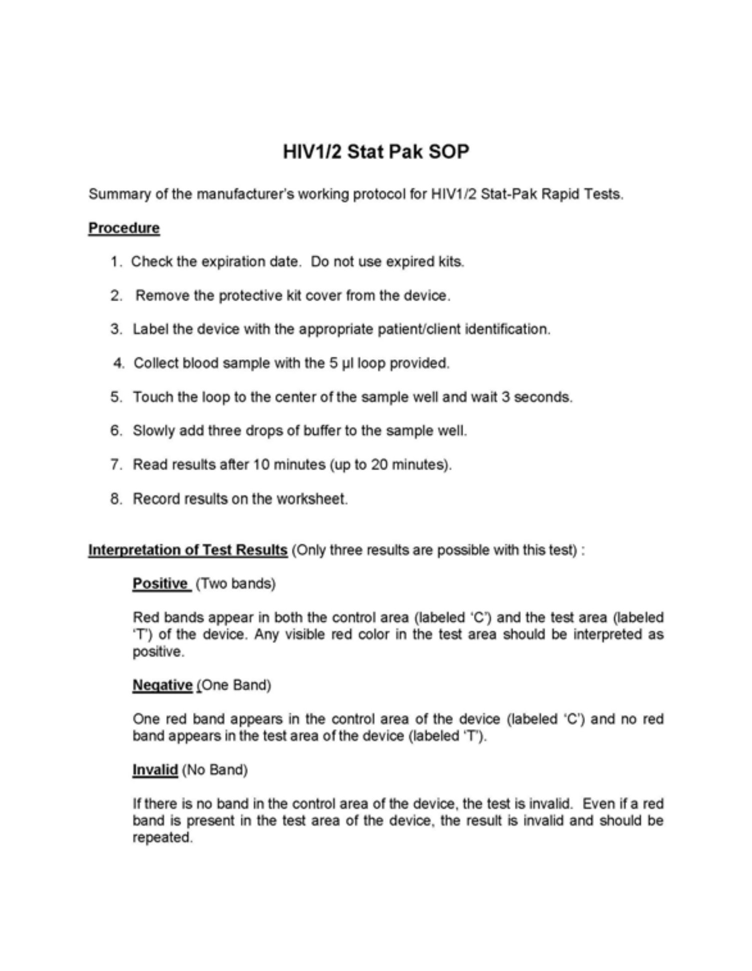

HIV1/2 Stat Pak SOP

Summary of the manufacturer's working protocol for HIV1/2 Stat-Pak Rapid Tests.

Procedure

1. Check the expiration date. Do not use expired kits.
2. Remove the protective kit cover from the device.
3. Label the device with the appropriate patient/client identification.
4. Collect blood sample with the 5 µl loop provided.
5. Touch the loop to the center of the sample well and wait 3 seconds.
6. Slowly add three drops of buffer to the sample well.
7. Read results after 10 minutes (up to 20 minutes).
8. Record results on the worksheet.

Interpretation of Test Results (Only three results are possible with this test) :

Positive (Two bands)

Red bands appear in both the control area (labeled 'C') and the test area (labeled 'T') of the device. Any visible red color in the test area should be interpreted as positive.

Negative (One Band)

One red band appears in the control area of the device (labeled 'C') and no red band appears in the test area of the device (labeled 'T').

Invalid (No Band)

If there is no band in the control area of the device, the test is invalid. Even if a red band is present in the test area of the device, the result is invalid and should be repeated.

- Approved for use in-country
- Must be kept up-to-date
- Must be followed precisely

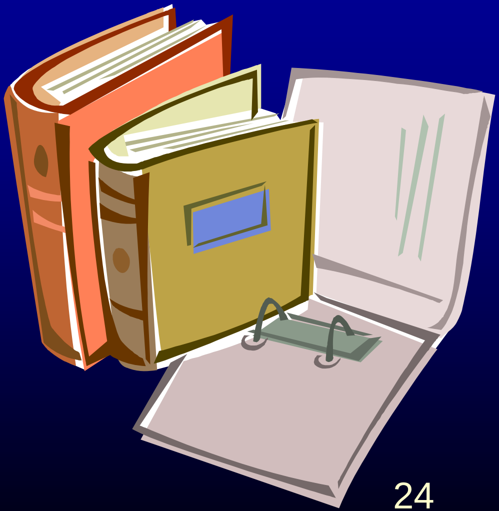

◆ Lab workers ◆ Health workers ◆ Counselors

MOH TT HIV Rapid Test Report

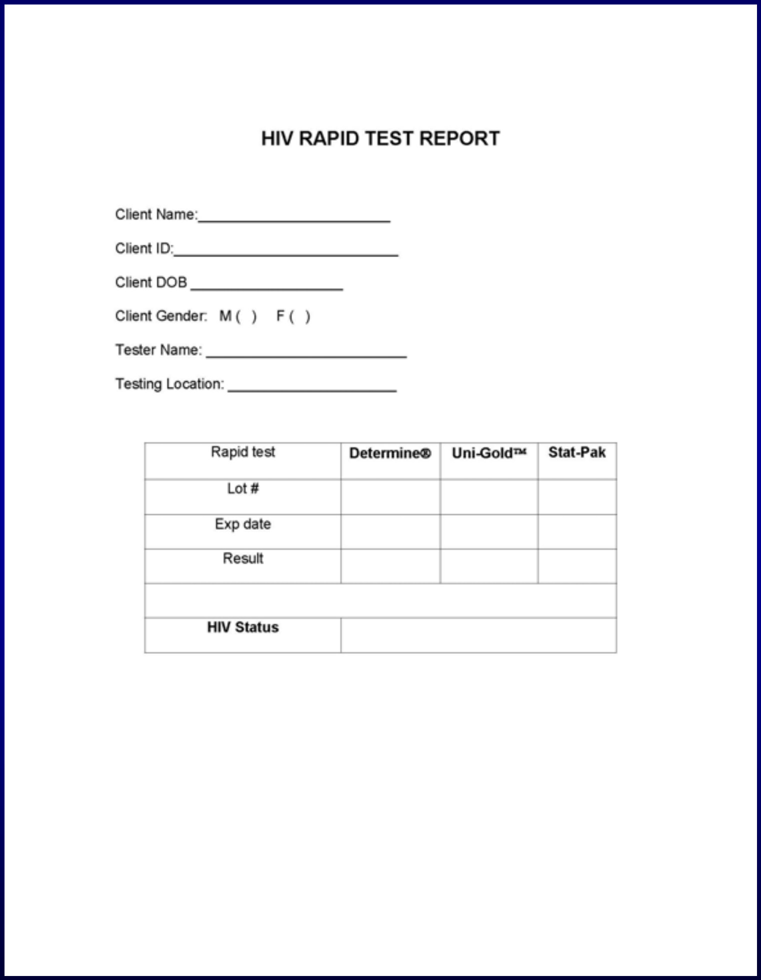

HIV RAPID TEST REPORT

Client Name:____________________

Client ID:______________________

Client DOB ________________

Client Gender: M ( ) F ( )

Tester Name: _____________________

Testing Location: __________________

Rapid test	**Determine®**	**Uni-Gold™**	**Stat-Pak**
Lot #			
Exp date			
Result			
**HIV Status**			

- Fill accurately
- Write legibly
- Date
- Sign

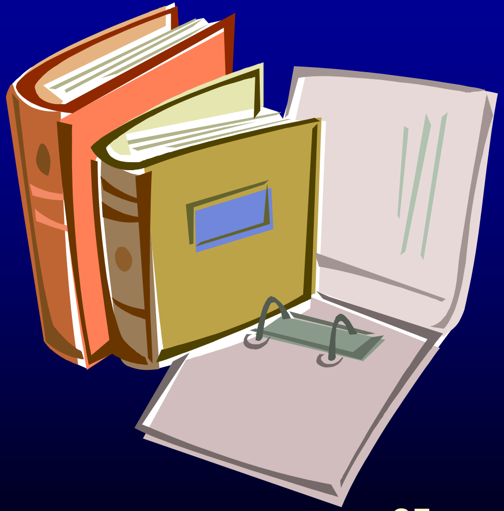

Lab workers · Health workers · Counselors

MOH TT Records

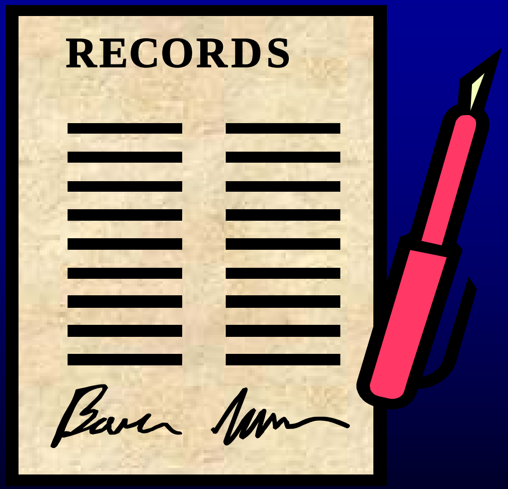

What Records Are Essential at MOH TT Test Sites?

- Temperature Logs
- QC Sample Logs
- Client test results
- Inventory records

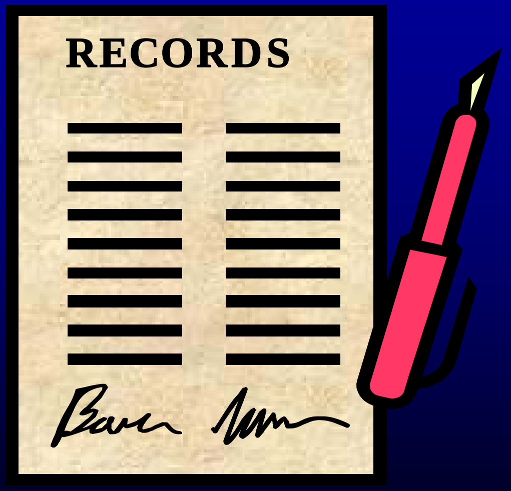

Proper Record-Keeping Results in Quality Testing

Record-keeping allows a test site to:

- Communicate accurately and effectively
- Quickly identify problems
- Minimize error
- Monitor quality system
- Assist management in:
  - Developing policy & plans
  - Monitoring and evaluating programs

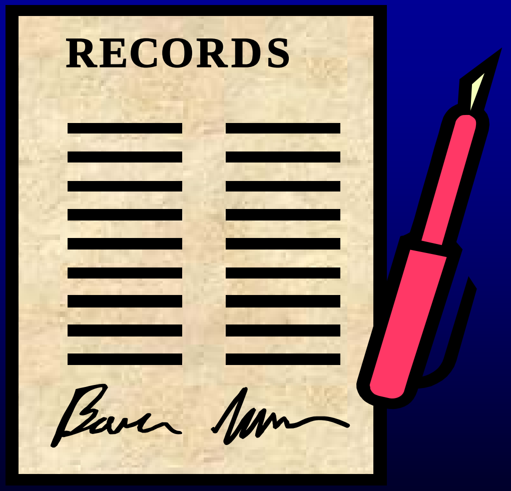

Lab workers Health workers Counselors

Tips for Good Record Keeping

- Understand the information to be collected
- Record the information every time
- Record all the information
- Record the information in the same way every time

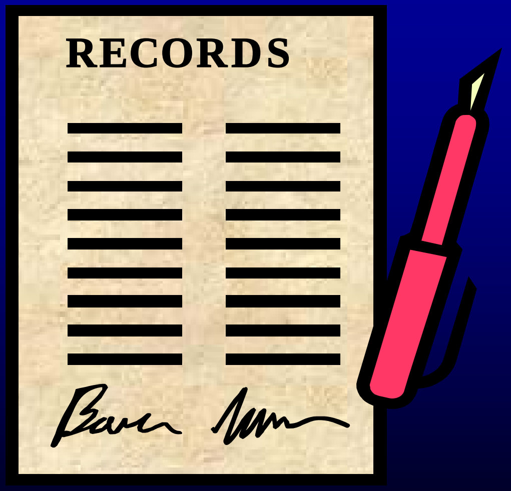

* PMTCT Generic Curriculum

How Long Should You Retain Client Test Records?

It depends on several factors:

- National policies
- Secure storage space at test site

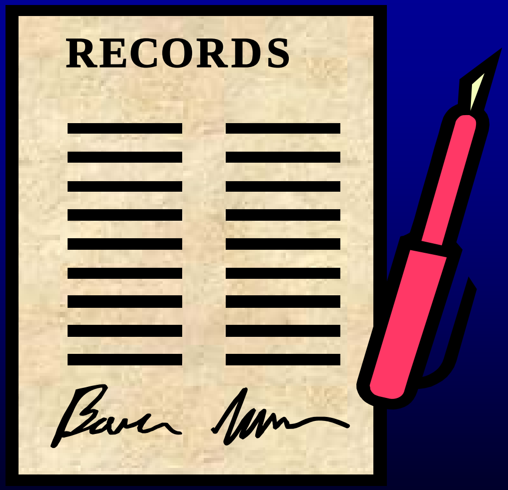

Lab workers   Health workers   Counselors

Records Should be Permanent, Secure, Traceable

- Permanent:
  - Keep books bound
  - Number pages
  - Use permanent ink
  - Control storage
- Secure:
  - Maintain confidentiality
  - Limit access
  - Protect from environmental hazards
- Traceable:
  - Sign and date every record

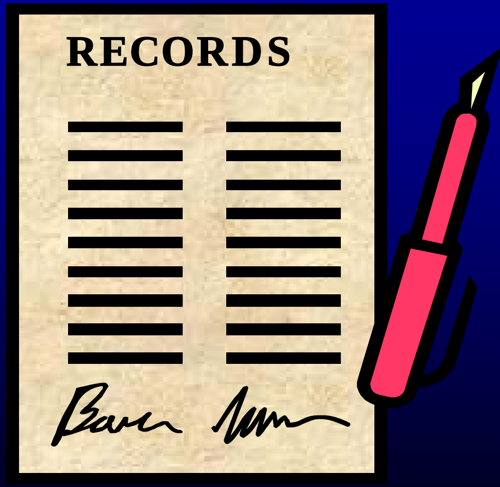

Lab workers Health workers Counselors

Information Recorded will Feed Into Monitoring and Evaluation Systems

- Provide in-country information:
  - When will what be reported?
  - How will it be reported?
  - Whom will it be reported to?
  - How will the data be used?

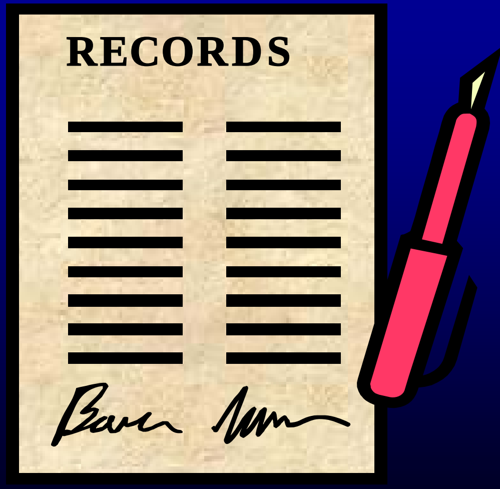

Summary

- What is a document?
- What is a record?
- List records used at MOH TT testing sites.
- What are SOPs?
- Why are SOPs important?
- What are some tips for good record-keeping?
- How should records be maintained?

Key Messages

- Written policies and procedures are the backbone of the quality system
- Complete quality assurance records make quality management possible
- Keeping records facilitates meeting program reporting requirements

Module 16: Professional Ethics

Learning Objectives

By the end of the module, you will be able to:

- Describe ethical issues related to HIV rapid testing
- Explain the importance of professional ethics
- Apply ethical conduct to HIV rapid testing
- Take appropriate actions to maintain client confidentiality

Content Outline

- What is ethics?
- Why is ethics important?
- Who is responsible for ethics?
- How is ethics applied to HIV rapid testing?
- Maintaining confidentiality
- Code of conduct

Scenario I

A pregnant woman comes for HIV testing. Your test site has just run out of the 2nd test in the algorithm. You tell her that she will have to come back in 2 days. She becomes very emotional and explains that she has traveled a long distance after finally deciding to get tested and won't be back in the area for a long time.

Feeling sorry for her, you proceed to perform test one, and report a resulting positive test to the client.

Scenario II

At the HIV rapid testing site, you discover that you just run out of the buffer for Test 1 of the algorithm. Rather than denying testing to clients, you decide to go ahead and perform Test 1 using the buffer from kits of Test 2.

Scenario III

Today is Monday. You discover that there are enough test devices to last through the entire week, but they will expire on Wednesday.

Since resources are tight and you don't want to waste any test kits (it is only a couple of days past expiration anyway), you decide to use the test devices until the end of the week.

What Could Be the Consequences of...

- A false positive HIV result?
- A false negative result?

What Is Ethics?

"A set of principles of right conduct"

Why is Ethics Important?

"Decisions about diagnosis, prognosis and treatment are frequently based on results and interpretations of laboratory tests. Irreversible harm may be caused by erroneous tests."

International Federation of Clinical Chemistry and Laboratory Medicine (IFCC)

Scenario IV

Rick, the tester, is excited about getting home at the end of his work day, because a relative he hasn’t seen in quite some time is scheduled to arrive. Right before he is ready to leave, he gets distracted by a phone call and forgets to lock up the lab register in the cabinet.

Maintaining Confidentiality

It is important to:

- Keep all client/patient information private
- Secure all records / logbooks
- Restrict access to testing areas

Warning

People often violate ethics not because they mean to, but because they are careless. As a matter of fact, they sometimes act with good intentions.

Role-Play

Watch the role-play and discuss:

- What happened?
- What were the ethical issues involved?
- What were the implications?
- What would you do if you were in this situation?

Who is Responsible for Ethics?

- EVERYONE!
  - Medical Laboratory Technician
  - Nurse Counselor
  - Clerk
  - Secretary
  - General Hand
  - Driver

How Do We Apply Ethics To HIV Rapid Testing?

- Work done
- Behavior of the staff
- Behavior of management

Code of Ethics (IFBLS)

Excerpts from International Federation of Biomedical Laboratory Science (IFBLS)

- Maintain strict confidentiality of patient information and test results
- Safeguard the dignity and privacy of patients
- Be accountable for the quality and integrity of clinical laboratory services

Code of Ethics (ASCP)

Excerpts from American Society for Clinical Pathology (ASCP)

- Treat patients and colleagues with respect, care and thoughtfulness
- Perform duties in an accurate, precise, timely and responsible manner
- Safeguard patient information as confidential, within the limits of the law
- Prudently use laboratory resources

Code of Ethics (CASMET)

Caribbean Association of Medical Technologists
(CASMET)

CODE OF ETHICS

On entering at this time into the practice of Medical Technology, I accept with full realization of its implication, the responsibility associated with my duties.

I am aware that since the Physician or Surgeon relies upon my work in the diagnosis and treatment of disease, even an apparent trivial error may affect seriously the health or even the life of a Patient. Every procedure, therefore, must be carried out with thoughtfulness and accuracy.

Knowing these things I recognize that my integrity and that of my profession must be pledged to the absolute reliability of my work.

I am aware of the need for co-operation and friendly understanding between my fellow workers and myself and for the patience, humanity and tact which must be exercised toward the patient who by reason of his illness is particularly needful of my skill and kindness.

I realize that the knowledge obtained concerning persons in the course of my work is privileged and confidential and that since the Physician or Surgeon has the ultimate responsibility in diagnosis and treatment, my results may be made known only to him or another having duly constituted authority.

To these principles I hereby subscribe, promising to conduct myself at all times in a manner appropriate to the dignity of my profession.

Features of CASMET Code of Ethics

Testing is

- Thoughtful and accurate
- Reliabile
- Confidential

Summary

- In your own words, what is ethics?
- Why is it important?
- Give examples of actions you can take to maintain client confidentiality.
- Give an example of a code of ethics to which you are willing to personally commit.

Key Messages

- Ethical issues are important. We must constantly remind ourselves of the code of conducts and ensure we do the right thing.
- Ethical issues are often hard to deal with because they create dilemmas.
- People often violate ethics not because they mean to, but because they are careless. As a matter of fact, they sometimes act with good intentions.
