# Supplementary material for: Same-visit HIV testing in Trinidad and Tobago
Source: BMC Public Health. 2010 Apr 9;10:185. doi: 10.1186/1471-2458-10-185 (PMC2858728; doi:10.1186/1471-2458-10-185)
Supplement: Additional file 3 — Reference Manual. This is the Reference Manual provided to each person trained to provide rapid tests to provide "same-visit" HIV test results. [file 1471-2458-10-185-S3.PDF]

Reference Manual

MOH TT Certified
"Same-Visit"
HIV Testing

2005

Insert Your Workshop Certificate Here

Insert Your MOH TT Certificate Here

Insert Your Workshop Certificate Here

MOH TT Certification of HIV Rapid Testing

MOH TT certification of HIV testing is an important part of the "same-visit" testing process. "Same visit" testing is initiated with a medical order from the Chief Medical Officer of TT to "conduct 'same-visit' testing on behalf of MOH TT." Only those who are certified by MOH TT to provide testing are eligible to implement this order.

Training for HIV testing is expensive and will be limited to those candidates with:

- Experience in HIV/AIDS counseling
- Access to opportunities to provide MOH TT "same-visit" testing within 2 months of completing the workshop

Full certification for those who provide "same visit" HIV testing on behalf of MOH TT includes:

- Successful completion of 20 hour HIV testing workshop.
  This means:
  - Score of >80% on the written exam
  - Score of 100% on the practical exam
- Successful completion of an internship under supervision of an MOH TT tester.
  This means:
  - Correct interpretation of HIV status for 50 samples using the MOH TT algorithm. This internship is only currently available at POSGH.
  - Completion of an externally-provided HIV verification panels with a score of 100%. This will be managed by the Quality Monitor of MOH TT.

Certificate of Completion

This certificate is awarded to

Upon successful completion of the Training for 'same-visit' HIV Testing Workshop

Held at the Lecture Theatre, CAREC

September 21-23, 2005

DR. LYNETTE BERKELEY,<br>LABORATORY MANAGER, CAREC	MS. ROBIN WEAVER<br>LABORATORY ADVISER, CAREC-SPSTI	Date

VCT and HIV Status

Many people in TT are unaware of their HIV-infection status. Current access to HIV testing in TT is limited. MOH TT intends to simplify the process by which people in TT can “know their status”. One of the parts of this process is “same visit” HIV testing.

Many countries provide Voluntary Counseling and Testing (VCT) services to increase access to HIV testing. VCT is a combination of two activities – counseling and testing – into a single service that amplifies the benefits of both activities. The VCT approach is useful in all settings – resource-rich and resource-poor – urban and rural. VCT is adaptable to client needs. People of all ages and backgrounds can have access to VCT services.

When “same-visit” HIV testing is part of VCT service, clients seeking to “know their status” can do so during a single visit to a VCT site. All clients leave VCT “same-visit” testing sites with a “risk reduction plan” and knowledge of their HIV-infection status. Risk reduction plans allow each client to know what personal changes they should make to reduce their *personal risk* of HIV infection. Clients who leave VCT sites with knowledge that they are HIV positive, also leave with information about additional referrals for support and treatment.

VCT services do not require medically trained personnel. VCT services can be provided by anyone who has certified training in both of the activities involved - counseling and testing. Your possession of this manual indicates that you have begun certification for MOH TT “same-visit” HIV testing. This testing will be used in VCT services in TT. When your certification for both testing and counseling is complete, you will be competent to provide VCT services in TT.

Universal Safety Precautions

Universal safety precautions are recommendations to reduce exposure to infectious agents.

Infectious agents are organisms or substances capable of causing infection, disease or death.

In practice, universal safety precautions include:

- Washing hands
- Wearing gloves
- Wearing lab coat or apron
- Disposing of contaminated materials properly
- Not eating, drinking or smoking in test areas
- Not storing food or drink in QC sample refrigerator
- Not applying make-up at the test site
- Not inserting (or removing) contact lenses at the test site
- Not placing pens, pencils or any other object in mouth

MOH TT HIV Rapid Test Algorithm

Step 1.

Perform two rapid tests (DETERMINE and UNI-GOLD) simultaneously (in parallel) using whole blood.

DETERMINE:	1 drop of whole blood (50 ul), apply to the test area<br>1 drop of chase buffer<br>Read result after 15 min
UNI-GOLD:	2 drops of whole blood, apply to the test area<br>2 drops of wash buffer<br>Read result after 10 minutes

Step 2.

If the first two tests are concordant (have the same result), no further testing is needed. If the first two tests are discordant (have different results), the third test is needed to resolve the discordance.
`

STAT-PAK	1 loop of blood sample, apply to the test area<br>3 drops of buffer<br>Read result after 10 minutes

Result Interpretation

DETERMINE	UNI-GOLD	ACTION
Negative	Negative	Record/Report NEGATIVE
Positive	Positive	Record/Report POSITIVE
Negative	Positive	Do STAT-PAK,<br><br>If negative, record/report NEGATIVE<br><br>If positive, record/report POSITIVE
Positive	Negative	Do STAT-PAK,<br><br>If negative, record/report NEGATIVE<br><br>If positive, record/report POSITIVE

MOH TT HIV Rapid Test Algorithm

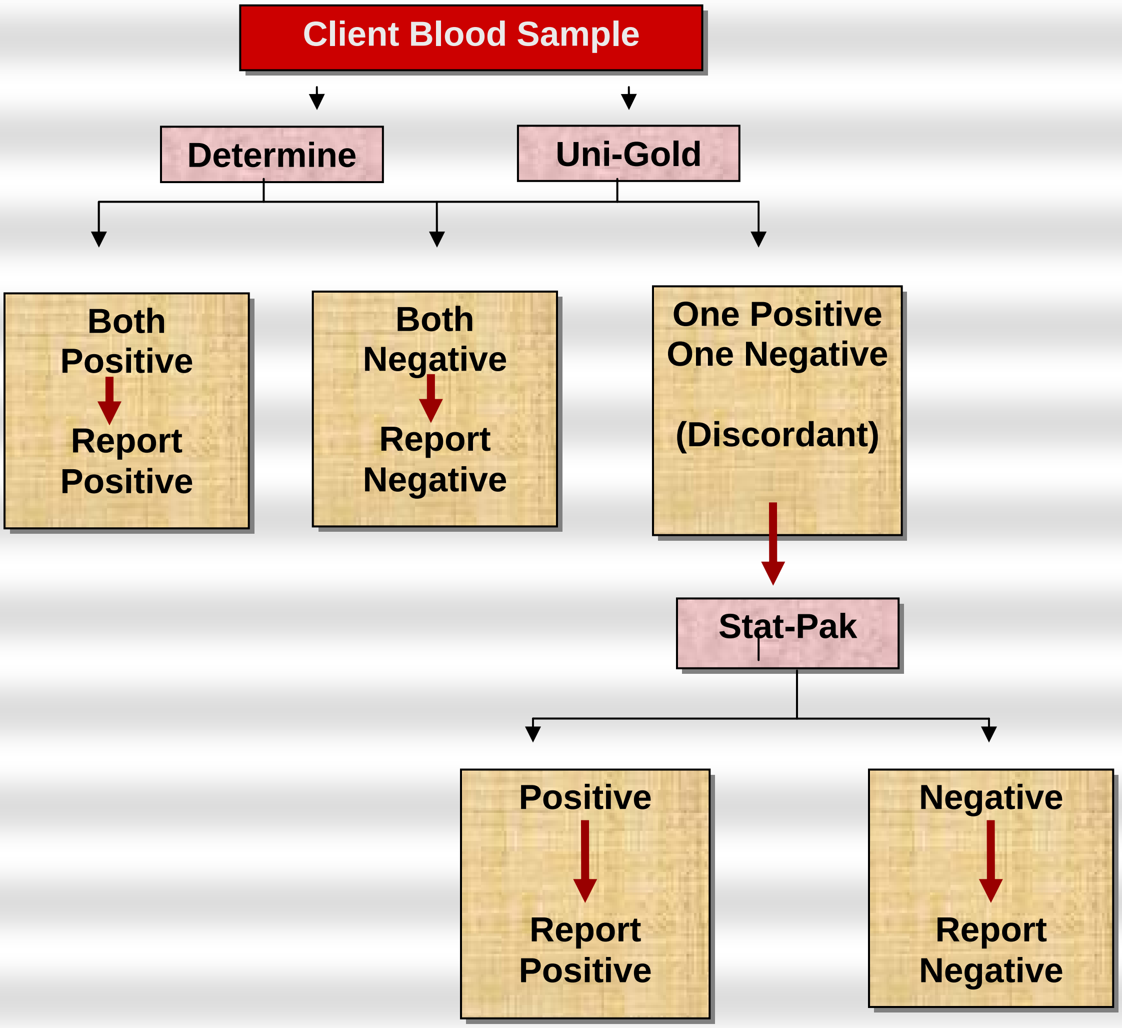

MOH TT certified diagnosis of infection requires use of this algorithm. Use of this algorithm permits testers to report HIV status to clients who want to "know their status" for HIV infection.

To receive their HIV status test report, each client must have two valid concordant test results.

Standard Operation at Testing Sites

Your responsibility is to maintain high-quality testing at your testing site. Each client who seeks HIV testing to "Know Their Status" must have confidence that the report they receive is correct. Routine delivery of quality reports requires that each testing site follow standard operating procedures. These procedures consist of three activities: 1) Temperature Logs, 2) QC Sample Logs (and testing), and 3) client testing. Forms and procedures for each of these activities are included in this manual. As the person performing testing, you must document each of these activities during each day of testing. These records show the activities at your testing site have been conducted properly. Standard operation at MOH TT testing sites includes:

1) **Temperature Logs.** Each day of testing, record three temperatures:

   a) Testing site temperature (less than 28°C),
   b) HIV rapid kit storage room temperature (less than 28°C)
   c) QC Sample refrigerator (2 - 8°C)

2) **QC Sample Logs**. Each day of testing, maintain a QC Sample Log:

   Accurate QC Sample Logs are essential to quality testing. Each person who provides test results must maintain a QC Sample Log. Each tester must test 6 control samples at the beginning of each day of testing. No client should be tested until the daily QC Sample Log has been completed. **If any problems arise in QC Sample testing, you must stop testing and contact your manager and the Quality Monitor.**

   To maintain a QC Sample Log, each tester must test two externally-provided QC samples (one HIV positive sample and one HIV negative sample) using each type of HIV rapid test (6 tests total). Performance of each test should follow the SOP for that test. Rapid test devices used for QC sample testing should be labeled "QC +" or QC –". Results of each QC Sample test must be recorded in the QC Sample Log. If a QC test result is wrong (a QC positive sample tests negative or vice versa), the test must be repeated with a second testing device of the same type. Both results should be recorded in the QC Sample Log. If the second test is also wrong, you must stop testing until the problem is resolved.

If a QC test result is invalid (no bands appear in the control window), the second QC Sample must be retested with a second device and the second result must also be recorded. As in the previous case, both sets of results should be recorded in the QC Sample Log. If the second test is also invalid, you must stop testing until the problem is resolved. No test should be recorded as “weakly reactive”.

3) Client Testing. When you have completed QC Sample testing satisfactorily, you are ready to receive clients for testing. When you have completed pre-test counseling with an individual client, you can begin testing. Before proceeding with client testing, you must record the lot number and expiry dates for one Determine® and one Uni-Gold™ device on the HIV Rapid Test Report. When you have recorded this information, open the Determine® and Uni-Gold™ rapid test devices. After preparing the client for collection of blood by finger stick blood draw, follow the procedure for Finger-Stick Blood Collection. Test this blood sample using each test device and following the SOP for the device.

Place the testing devices out-of-sight during incubation of the test. An easy way to do this is to place a box up-side-down over the devices. Remove the cover when the timer indicates the test is complete.

Interpret the test results for your client. If the results of are concordant, the diagnosis can reported to the client on the basis of the two tests. If the results are discordant, a third test is needed to resolve the discordance. If either test is invalid, you cannot report a result to the client. You must refer the client for additional testing at another location.

If a third test is required, you must repeat the finger-stick blood collection. The second sample must be tested using a Stat-Pak testing device and following the SOP for Stat-Pak. You should also incubate this test out-of-sight. **The result of the Stat-Pak test is the HIV status for the client**: if the Stat-Pak result is negative, the HIV status is negative. If the Stat-Pak result is positive, the HIV status is positive. Add results of the Stat-Pak test to the HIV Rapid Test Report. The HIV status reported to the client should also be indicated on the form. These records may be either manual or electronic.

HIV Tests

Many types of HIV tests are available. Testing for HIV infection provides evidence that HIV antibodies are present. All tests used to allow clients to “know their HIV status” are based on technical principles for detection of antibodies to HIV.

The types of tests included in the MOH TT “same-visit” testing process are HIV rapid tests. In MOH TT-certified testing, no *single* test result is sufficient to provide clients with information about their HIV-infection status. In the MOH TT-certified testing process, results from an algorithm (a specific combination of HIV rapid tests) are required to provide clients with their HIV status.

Principle of Tests: HIV rapid tests detect antibodies to Human Immunodeficiency Virus (HIV) types 1 and 2 in serum, plasma and whole blood. The principle of each of the three tests (Determine®, Uni-Gold™, and Stat-Pak) in the MOH TT algorithm is the same.

Each HIV rapid test contains a reagent strip. Each reagent strip contains three separate areas: sample application, internal control, and result. HIV 1 and HIV 2 proteins (antigens) linked to colloidal gold (or another agent) are present in the result region of the reagent strip. When sample (blood) and buffer are added to the specimen area, they migrate along the strip. If antibodies to either HIV 1 or HIV 2 are present in the sample, a pink/red band appears in the “result” area. If no HIV antibodies are present in the sample, no band will appear in the “result” area.

Each reagent strip also contains an internal control area. This area has been sensitized to show a pink/red band when the test is performed. If this “control” line does not appear, the individual test result is invalid. No test result is available from invalid tests. Information about what to do when invalid test results are obtained is included in the daily testing procedures.

Finger-Stick Blood Draw

Always use universal safety precautions

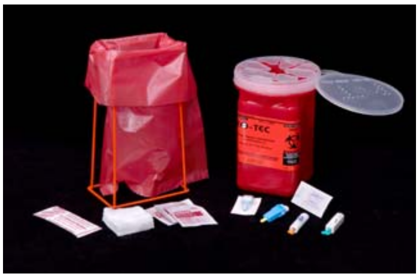

1. Collect supplies. Wear gloves.

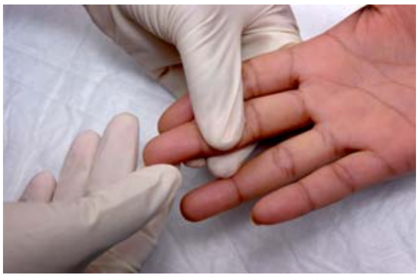

2. Choose whichever finger is least calloused.

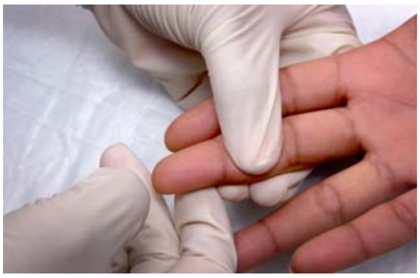

3. Massage the finger to increase blood to flow.

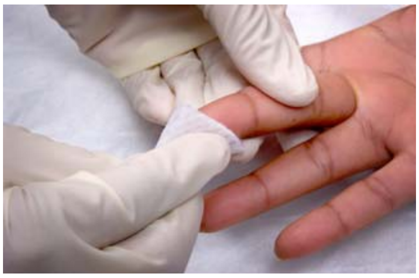

4. Clean fingertip with alcohol. Work from the middle out to reduce contamination. Allow the area to dry.

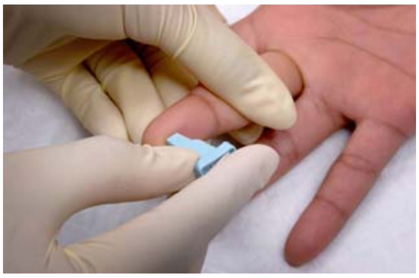

5. Grasp the finger and place a new sterile lancet on the side of the fingertip.

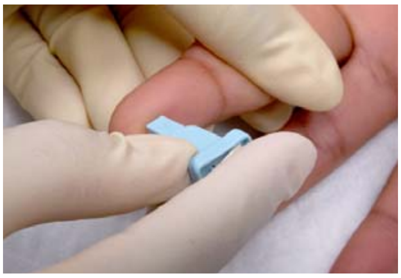

6. Firmly press the lancet to puncture the fingertip.

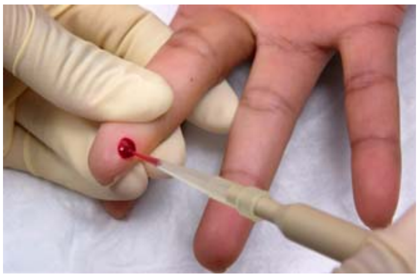

7. Collect the sample. Blood may flow best when finger is held lower than the elbow.

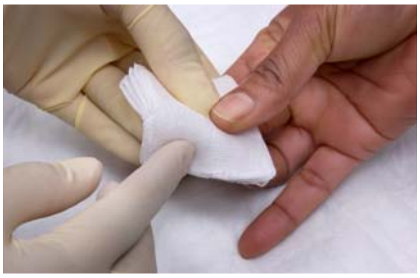

8. Apply a gauze pad or cotton ball to puncture site until bleeding stops.

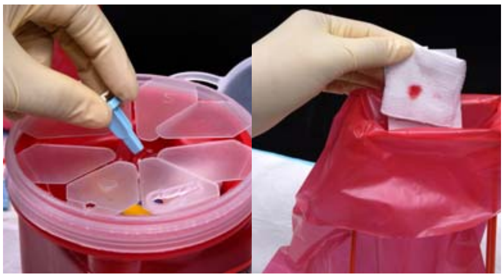

9. Properly dispose of all contaminated supplies.

WHO

Use of trade names and commercial sources is for identification only and does not imply endorsement by WHO, the Public Health Service, or by the U.S. Department of Health and Human Services (2005).

CDC

Determine® HIV-1/2 SOP

Summary of the manufacturer's working protocol for Determine® Rapid Tests.

Procedure

1. Check the expiration date. Do not use expired kits.
2. Remove the protective kit cover from the test device.
3. Label test device with the appropriate patient/client identification.
4. Apply one drop of whole blood to the sample pad marked by an arrow symbol.
5. Wait one minute until blood is absorbed into the sample pad.
6. Apply 1 drop of Chase Buffer to the sample pad.
7. Read the result after15 minutes.
8. Record results on the worksheet

Interpretation of Test Results (Only three results are possible with this test):

Positive (Two bands)

Red bands appear in both the control window (labeled 'C') and the patient window (labeled 'P') of the strip. Any visible red color in the patient window should be interpreted as positive.

Negative (One Band)

One red band appears in the control window of the strip (labeled 'C') and no red band appears in the patient window of the strip (labeled 'P').

Invalid (No Band)

If there is no band in the control window of the strip the test is invalid. Even if a red band is present in the patient window of the strip, the result is invalid and should be repeated.

Determine HIV Rapid Test

(For use with whole blood, serum, or plasma)
Store kit: 2 - 30° C

- **Check kit before use. Use only items that have not expired or been damaged.**
- **Bring kit and previously stored specimens to room temperature prior to use.**
- **Always use universal safety precautions when handling specimens. Keep work areas clean and organized.**

This outline is not intended to replace the product insert or your standard operating procedure (SOP).

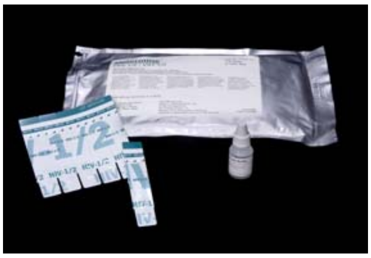

1. Collect test items and other necessary lab supplies.

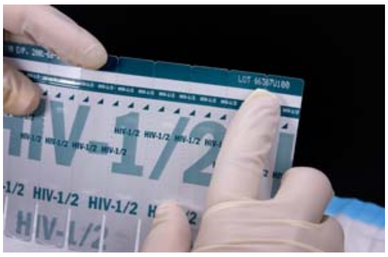

2. Use 1 strip per test and be sure to preserve the lot number on the remaining packet of strips.

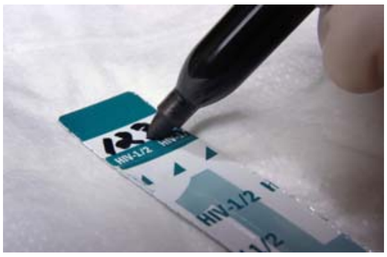

3. Label the test strip with client identification number.

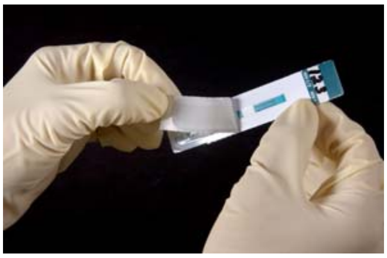

4. Pull off the protective foil cover.

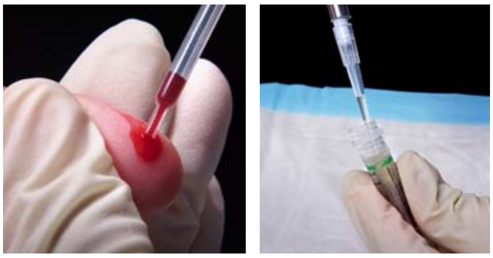

5. Collect 50 µl of specimen using either a transfer or a precision pipette.

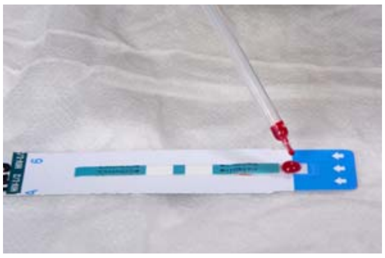

6. Apply the specimen to the absorbent pad on the strip.

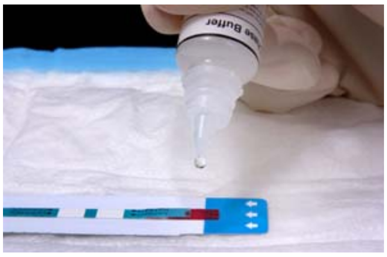

7. <u>For whole blood only</u> add 1 drop of chase buffer to the specimen pad.

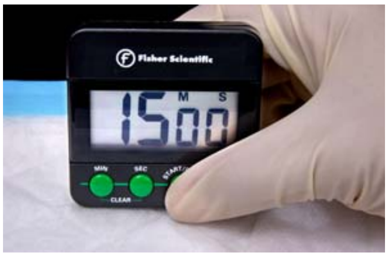

8. Wait 15 minutes (no longer than 60 minutes) before reading the results.

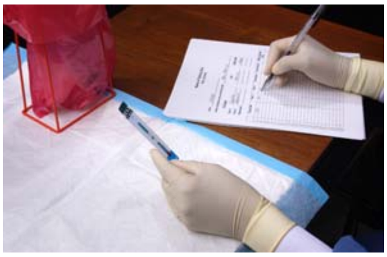

9. Read and record the results and other pertinent info on the worksheet.

Determine HIV Rapid Test Results

Positive

2 lines of any intensity appear in both the control and client areas.

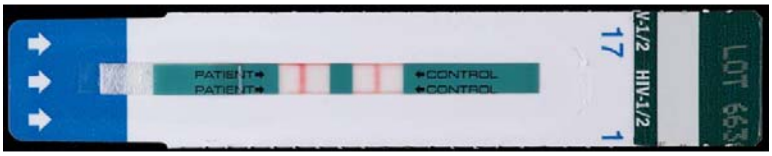

Negative

1 line appears in the control area and no line in the client area.

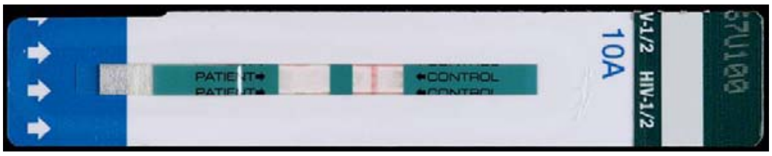

Invalid

No line appears in the control area. Do not report invalid results. Repeat test with a new test device even if a line appears in the client area.

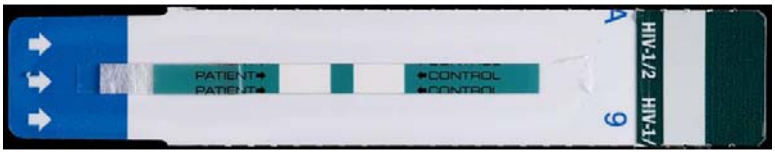

WHO

Use of trade names and commercial sources is for identification only and does not imply endorsement by WHO, the Public Health Service, or by the U.S. Department of Health and Human Services (2005).

CDC

Uni-Gold™ HIV SOP

Summary of the manufacturer's working protocol for Uni-Gold™ HIV Rapid Tests.

Procedure

1. Check the expiration date. Do not use expired kits.
2. Remove the protective kit cover from test device.
3. Label test device with the appropriate patient/client identification.
4. Apply two drops of whole blood to the sample well.
5. Apply 2 drops of Wash Reagent to the sample well.
6. Read the result after 10 minutes.
7. Record results on the worksheet.

Interpretation of Test Results (Only three results are possible with this test):

Positive (Two bands)

Red bands appear in both the control area (labeled 'C') and the test area (labeled 'T') of the device. Any visible red color in the test area should be interpreted as positive.

Negative (One Band)

One red band appears in the control area of the device (labeled 'C') and no red band appears in the test area of the device (labeled 'T').

Invalid (No Band)

If there is no band in the control area of the device, the test is invalid. Even if a red band is present in the test area of the device, the result is invalid and should be repeated.

Uni-Gold HIV Rapid Test

For use with whole blood, serum, or plasma
Store Kits: 2 - 30° C

- **Check kit before use. Use only items that have not expired or been damaged.**
- **Bring kit and previously stored specimens to room temperature prior to use.**
- **Always use universal safety precautions when handling specimens. Keep work areas clean and organized.**

This outline is not intended to replace the product insert or your standard operating procedure (SOP).

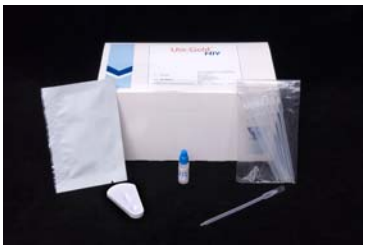

1. **Collect test items and other necessary lab supplies.**

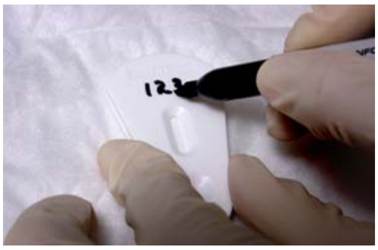

2. **Remove device from package and label device with client identification number.**

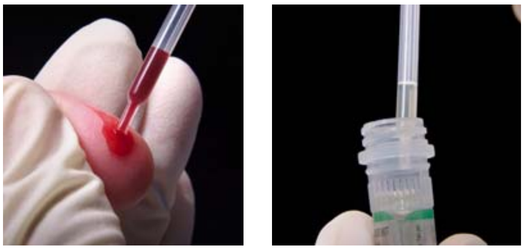

3. **Collect specimen using the disposable pipette.**

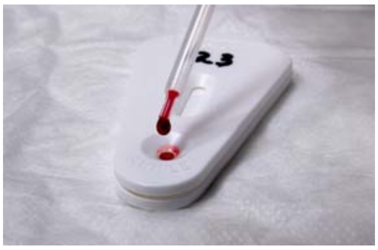

4. **Add 2 drops (approx. 60µl) of specimen to the sample port in the device.**

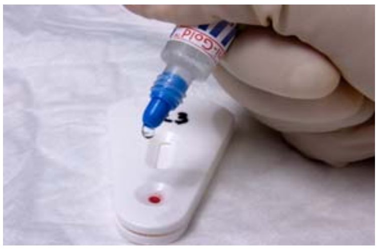

5. **Add 2 drops (approx. 60µl) of the appropriate wash reagent to sample port.**

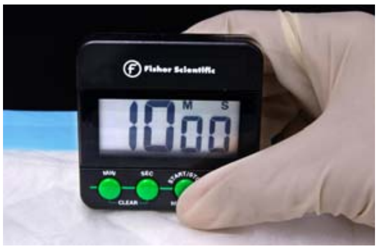

6. **Wait for 10 minutes (no longer than 20 min.) before reading the results.**

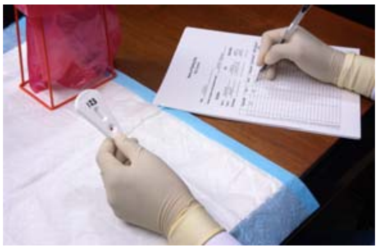

7. **Read and record the results and other pertinent info on the worksheet.**

Uni-Gold HIV Rapid Test Results

Positive

2 lines of any intensity appear in both the control and test areas.

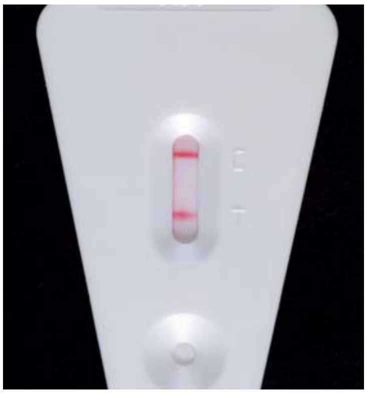

Negative

1 line appears in the control area and no line in the test area.

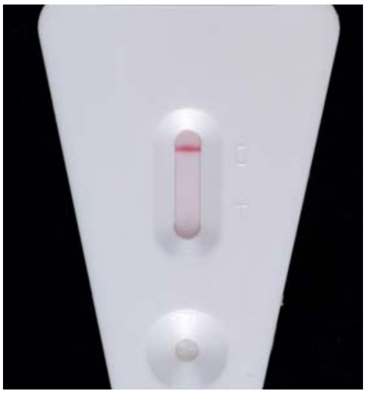

Invalid

No line appears in the control area. Do not report invalid results. Repeat test with a new test device even if a line appears in the test area.

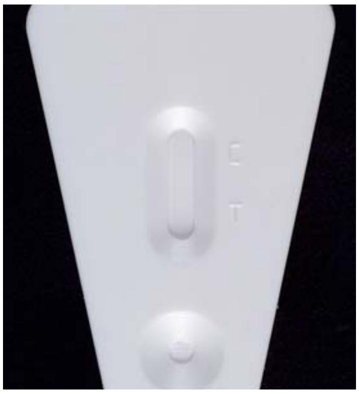

WHO

Use of trade names and commercial sources is for identification only and does not imply endorsement by WHO, the Public Health Service, or by the U.S. Department of Health and Human Services (2005).

HIV1/2 Stat Pak SOP

Summary of the manufacturer's working protocol for HIV1/2 Stat-Pak Rapid Tests.

Procedure

1. Check the expiration date. Do not use expired kits.
2. Remove the protective kit cover from the device.
3. Label the device with the appropriate patient/client identification.
4. Collect blood sample with the 5 µl loop provided.
5. Touch the loop to the center of the sample well and wait 3 seconds.
6. Slowly add three drops of buffer to the sample well.
7. Read the result after 10 minutes.
8. Record results on the worksheet.

Interpretation of Test Results (Only three results are possible with this test) :

Positive (Two bands)

Red bands appear in both the control area (labeled 'C') and the test area (labeled 'T') of the device. Any visible red color in the test area should be interpreted as positive.

Negative (One Band)

One red band appears in the control area of the device (labeled 'C') and no red band appears in the test area of the device (labeled 'T').

Invalid (No Band)

If there is no band in the control area of the device, the test is invalid. Even if a red band is present in the test area of the device, the result is invalid and should be repeated.

HIV 1/2 Stat-Pak

For use with whole blood, serum, or plasma
Store Kits: 8 - 30° C

- **Check kit before use. Use only items that have not expired or been damaged.**
- **Bring kit and previously stored specimens to room temperature prior to use.**
- **Always use universal safety precautions when handling specimens. Keep work areas clean and organized.**

This outline is not intended to replace the product insert or your standard operating procedure (SOP).

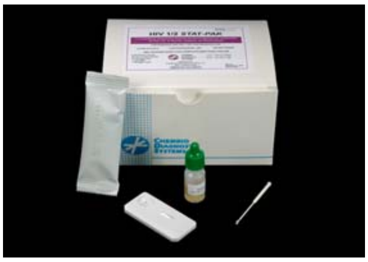

1. **Collect test items and other necessary lab supplies.**

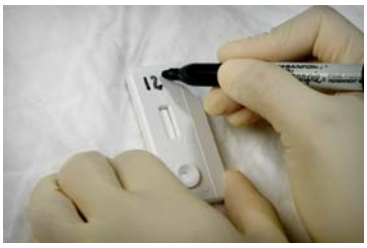

2. **Remove device from package and label device with client identification number.**

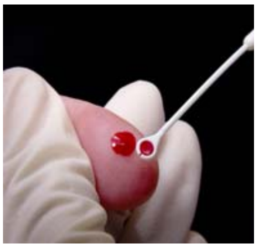

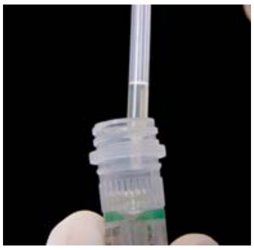

3. **Collect approximately 5 µl of specimen using a new disposable loop or pipette.**

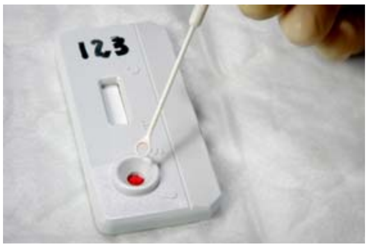

4. **Dispense the sample in the center of SAMPLE well.**

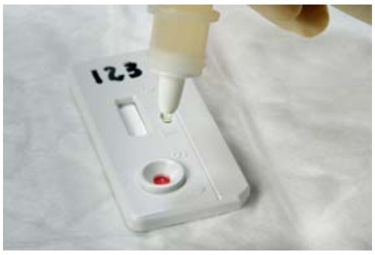

5. **Add 3 drops of buffer, holding vial vertically over the SAMPLE well.**

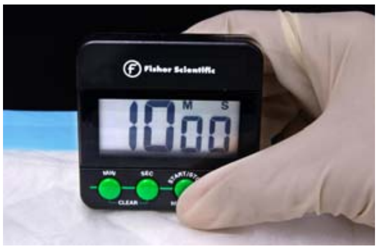

6. **Wait for 10 minutes before reading the results.**

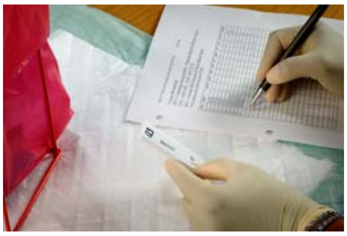

7. **Read and record the results and other pertinent info on the worksheet.**

HIV 1 / 2 Stat-Pak Test Results

Positive

2 lines of any intensity appear in both the control and test areas.

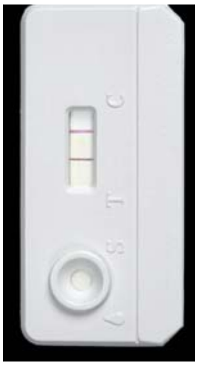

Negative

1 line appears in the control area and no line in the test area.

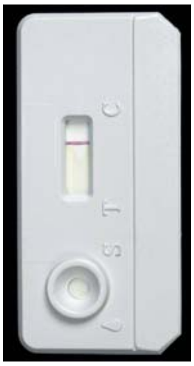

Invalid

No line appears in the control area. Do not report invalid results. Repeat test with a new test device even if a line appears in the test area.

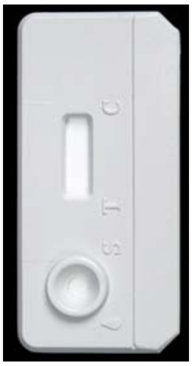

Use of trade names and commercial sources is for identification only and does not imply endorsement by WHO, the Public Health Service, or by the U.S. Department of Health and Human Services (2005).

Year ___________

Kit Storage
Temperature Record

(less than 28 ^{0}C)

	1	2	3	4	5	6	7	8	9	10	11	12	13	14	15	16	17	18	19	20	21	22	23	24	25	26	27	28	29	30	31
Jan																															
Feb																															
Mar																															
Apr																															
May																															
Jun																															
Jul																															
Aug																															
Sep																															
Oct																															
Nov																															
Dec																															

Year ___________

Testing Area
Temperature Record

(less than 28 ^{0}C)

	1	2	3	4	5	6	7	8	9	10	11	12	13	14	15	16	17	18	19	20	21	22	23	24	25	26	27	28	29	30	31
Jan																															
Feb																															
Mar																															
Apr																															
May																															
Jun																															
Jul																															
Aug																															
Sep																															
Oct																															
Nov																															
Dec																															

Manufacturer________________
Model No___________________

Year ____________

QS Sample Refrigerator
Temperature Record

Range (2 to 8 ^{0}C)

	1	2	3	4	5	6	7	8	9	10	11	12	13	14	15	16	17	18	19	20	21	22	23	24	25	26	27	28	29	30	31
Jan																															
Feb																															
Mar																															
Apr																															
May																															
Jun																															
Jul																															
Aug																															
Sep																															
Oct																															
Nov																															
Dec																															

QC SAMPLE LOG

Date and Time	Tester	QC samples Lot #	Determine®			Uni-Gold™			Stat-Pak		
			Lot #	Ex Date	Result	Lot #	Ex Date	Result	Lot #	Ex Date	Result
		Pos									
		Neg									
		Pos									
		Neg									
		Pos									
		Neg									
		Pos									
		Neg									
		Pos									
		Neg									
		Pos									
		Neg									
		Pos									
		Neg									
		Pos									
		Neg									
		Pos									
		Neg									
		Pos									
		Neg									

Inventory Record at HIV Rapid testing site

- Item Name: ______________________________ Unit: ___________
- Minimum Stock (Re-Order Level): ___________
- Site: _________________________

Date	Received From	Issued To	Quantity Received	Lot #	Amount used	Balance	Signature

HIV RAPID TEST REPORT

Client Name:________________________

Client ID:____________________________

Client DOB ___________________

Client Gender: M (   )   F (   )

Tester Name: _________________________

Testing Location: _____________________

Rapid test	**Determine®**	**Uni-Gold™**	**Stat-Pak**
Lot #			
Exp date			
Result			
**HIV Status**			

Accident Report Form

Date:__________________

Time:__________________

What happened?

What did you do?

Signature
