# Supplementary material for: Same-visit HIV testing in Trinidad and Tobago
Source: BMC Public Health. 2010 Apr 9;10:185. doi: 10.1186/1471-2458-10-185 (PMC2858728; doi:10.1186/1471-2458-10-185)
Supplement: Additional file 4 — Certification Criteria. This document lists certification criteria and supplies for both testers and venues that provide "same-visit" HIV test results. [file 1471-2458-10-185-S4.PDF]

Check List: HIV Testing Site

Reception Area	Check	Essential
Tables and drawers		Yes
Chairs		Yes
Registration forms or client cards		yes
Client record (intake)		Yes
Behavior change materials		Yes
Stationery		Yes
Clock		No
**Waiting Area**		
TV/VCR		No
Radio/Cassette player		No
Posters		Yes
Pamphlets		Yes
Chairs		Yes
Drinking water		No
**Counseling Room**		
Table with lockable drawers		Yes
3 chairs		Yes
Counseling protocol and cue cards		Yes
Condom demonstration tools e.g., penis model		Yes
Condoms Male		Yes
Condoms Female		No
Referral notebooks		Yes
Stamp pads		Yes
Posters		Yes
Screens/curtains (if HIV testing is done in the counseling room		Yes
Stationary (files, pens, notepads, etc.)		Yes
Tissue paper/Paper towels		Yes
Timer/Clock		Yes
**Testing Room**		
Test kits		Yes
Lancets		Yes
Sharps disposal container		Yes
Waste disposal bag (non sharps)		Yes
Pipettes		Yes
Tubes, needles, test tube rack		Yes

Centrifuge		No
Refrigerator		Yes
Incinerator		No
Gloves		Yes
Disinfectant + cleaning agent		Yes
Sundry supplies - cotton, dishes		Yes
Needles and syringes		Yes
Soap and water		Yes
Lighting		Yes
Protective wear		Yes
Stationery		Yes
Worksheets		Yes
Filing cabinet		Yes
Lab slips		Yes
Stamp pad		Yes
**Management Information System**		
Client number system		
Computer and accessories		No
Stationery		Yes
Lockable drawers		Yes
Client labels		Yes
Chairs and tables		Yes
Lockable cupboards/file cabinets for client records		Yes
Office supplies (pens, paper, staples, hole punch, binders, etc.)		Yes

Essential items are those that the HIV counselor/tester will need before seeing clients.

The nonessential items are those that are good to have at a "same-visit" HIV testing site and that a site manager can acquire for a site after testing has begun.

Note: A refrigerator is essential for storage of QC materials used in MOHTT HIV testing.

MOH TT Certification Criteria for Testers

MOH TT certification of HIV testing is an important part of the "same-visit" testing process. "Same visit" testing is initiated with a medical order from the Chief Medical Officer of TT to "conduct 'same-visit' testing on behalf of MOH TT." Only those who are certified by MOH TT to provide testing are eligible to implement this order.

Training for HIV testing is expensive and will be limited to those candidates with:

- Experience in HIV/AIDS counseling

- Access to opportunities to provide MOH TT "same-visit" testing within 2 months of completing the workshop

Full certification for those who provide "same visit" HIV testing on behalf of MOH TT includes:

- Successful completion of 20 hour HIV testing workshop.
  This means:
  - Score of >80% on the written exam
  - Score of 100% on the practical exam

- Successful completion of an internship under supervision of an MOH TT tester.
  This means:
  - Correct interpretation of HIV status for 50 samples using the MOH TT algorithm. This internship is only currently available at POSGH.
  - Completion of an externally-provided HIV verification panels with a score of 100%. This will be managed by the Quality Monitor of MOH TT.

“Same-Visit” HIV Testing Certification Supplies

This budget was prepared using prices from local suppliers including the MOHTT Medical Supply Warehouse. The protocol specifies that each MOHTT-tester must test 50 samples to complete the certification process. This budget includes supplies needed for 20 participants to complete certification.

Item	Amt	Unit cost	Total TT$*
**Testing supplies**			
Determine HIV Rapid Tests	1600 tests	$2016.00/kit of 100	32,256.00
Uni-Gold HIV Rapid Tests	1600 tests	$325.00/kit of 20	26,000.00
Stat-Pak HIV Rapid Tests	1600 tests	$290.00/kit of 20	23,200.00
Retractable Safety Lancets	1 case	$3,600.00	3,600.00
Lab coats (2 sizes)	2 cases	$934.00 /case	1,868.00
Gloves (3 sizes)	3 boxes	$100.00/box	300.00
Paper Towels	20 rolls	$10.00/roll	200.00
Timers	20	$300.00 ea	6,000.00
Transfer pipettes	3 cases	$341.00/case	1,021.00
Verification panels	30	$740.00 ea	22,200.00
Autoclave bags	3 packs	$100.00/pack	300.00
Sharpies	20	$4.00 ea	80.00
Alcohol prep pads	3 cases	$200.00 /case	150.00
Gauze 4” squares	3 packs	$50.00/pack	150.00
Masking tape	1 roll	$30.00/roll	30.00
Duct tape	1 roll	$50.00/roll	50.00
Bleach	1 gallon	$20.00/gallon	20.00
Tubes/vials	10 bags	$100.00/bag	1000.00
Racks (for above)	20	$300.00 ea	6000.00
Thermometer (0-100°C)	20	$50.00 ea	300.00
Testing Supplies Sub-total			124,725.00
**Reference Manual**			
3-ring binders ½”	25	$10.00 ea	250.00
Copy-paper	1 ream	$30.00 ream	30.00
Colour copies	120	$9.00 ea	1,080.00
Dividers	50 packs	$20.00 pack	1000.00
Plastic page protectors	375	$0.60 ea	225.00
Reference Manual Sub-total			2,335.00
Total Supply Cost			TT$ 127,060.00
Total Supply Cost in US$			US$ 21,176.66

Cost per participant = TT$ 6,353.00 (US$ 1,065.00)

*One US dollar is approximately six TT dollars. Divide all $TT values by 6 for approximate $US values.
