# Supplementary material for: Same-visit HIV testing in Trinidad and Tobago
Source: BMC Public Health. 2010 Apr 9;10:185. doi: 10.1186/1471-2458-10-185 (PMC2858728; doi:10.1186/1471-2458-10-185)
Supplement: Additional file 5 — Check List: HIV Testing Site. This document lists items that must be ready on-site before testing can begin. [file 1471-2458-10-185-S5.PDF]

Check List: HIV Testing Site

Reception Area	Check	Essential
Tables and drawers		Yes
Chairs		Yes
Registration forms or client cards		yes
Client record (intake)		Yes
Behavior change materials		Yes
Stationery		Yes
Clock		No
**Waiting Area**		
TV/VCR		No
Radio/Cassette player		No
Posters		Yes
Pamphlets		Yes
Chairs		Yes
Drinking water		No
**Counseling Room**		
Table with lockable drawers		Yes
3 chairs		Yes
Counseling protocol and cue cards		Yes
Condom demonstration tools e.g., penis model		Yes
Condoms Male		Yes
Condoms Female		No
Referral notebooks		Yes
Stamp pads		Yes
Posters		Yes
Screens/curtains (if HIV testing is done in the counseling room		Yes
Stationary (files, pens, notepads, etc.)		Yes
Tissue paper/Paper towels		Yes
Timer/Clock		Yes
**Testing Room**		
Test kits		Yes
Lancets		Yes
Sharps disposal container		Yes
Waste disposal bag (non sharps)		Yes
Pipettes		Yes
Tubes, needles, test tube rack		Yes

Centrifuge		No
Refrigerator		Yes
Incinerator		No
Gloves		Yes
Disinfectant + cleaning agent		Yes
Sundry supplies - cotton, dishes		Yes
Needles and syringes		Yes
Soap and water		Yes
Lighting		Yes
Protective wear		Yes
Stationery		Yes
Worksheets		Yes
Filing cabinet		Yes
Lab slips		Yes
Stamp pad		Yes
**Management Information System**		
Client number system		
Computer and accessories		No
Stationery		Yes
Lockable drawers		Yes
Client labels		Yes
Chairs and tables		Yes
Lockable cupboards/file cabinets for client records		Yes
Office supplies (pens, paper, staples, hole punch, binders, etc.)		Yes

Essential items are those that the HIV counselor/tester will need before seeing clients.

The nonessential items are those that are good to have at a "same-visit" HIV testing site and that a site manager can acquire for a site after testing has begun.

Note: A refrigerator is essential for storage of QC materials used in MOHTT HIV testing.
