# Supplementary material for: Same-visit HIV testing in Trinidad and Tobago
Source: BMC Public Health. 2010 Apr 9;10:185. doi: 10.1186/1471-2458-10-185 (PMC2858728; doi:10.1186/1471-2458-10-185)
Supplement: Additional file 6 — Tester certification training program. This document provides an overview of the three-step training program required for tester certification. [file 1471-2458-10-185-S6.PDF]

MOH TT Certification Criteria for Testers

MOH TT certification of HIV testing is an important part of the "same-visit" testing process. "Same visit" testing is initiated with a medical order from the Chief Medical Officer of TT to "conduct 'same-visit' testing on behalf of MOH TT." Only those who are certified by MOH TT to provide testing are eligible to implement this order.

Training for HIV testing is expensive and will be limited to those candidates with:

- Experience in HIV/AIDS counseling

- Access to opportunities to provide MOH TT "same-visit" testing within 2 months of completing the workshop

Full certification for those who provide "same visit" HIV testing on behalf of MOH TT includes:

- Successful completion of 20 hour HIV testing workshop.
  This means:
  - Score of >80% on the written exam
  - Score of 100% on the practical exam

- Successful completion of an internship under supervision of an MOH TT tester.
  This means:
  - Correct interpretation of HIV status for 50 samples using the MOH TT algorithm. This internship is only currently available at POSGH.
  - Completion of an externally-provided HIV verification panels with a score of 100%. This will be managed by the Quality Monitor of MOH TT.
