# Supplementary material for: Same-visit HIV testing in Trinidad and Tobago
Source: BMC Public Health. 2010 Apr 9;10:185. doi: 10.1186/1471-2458-10-185 (PMC2858728; doi:10.1186/1471-2458-10-185)
Supplement: Additional file 7 — Hands-on training materials. This document describes the hands-on portion of training for tester-certification. [file 1471-2458-10-185-S7.PDF]

Preparation for Laboratory Sessions

The MOHTT workshop included 4 laboratory sessions. This document contains the three files that guided preparation of those sessions:

- Instructor preparation of unknown samples and QC materials
- Laboratory Preparation of student unknown specimens
- Laboratory Practicals: Setup and Activity

Instructor preparation of unknown samples and QC materials

1. For 20 participants:
    - HIV Positive blood - 80ml
    - HIV negative blood – 60ml
    - HIV positive plasma/serum – 30ml
    - HIV negative plasma/serum – 30ml.
2. Label tubes 1-10 for each participant
3. Dispense positive blood in 0.5 ml amounts into tubes 2, 3, 5, 6, 9, and 10.
4. Dispense negative blood in 0.5 ml amounts into tubes 1, 4, 7, and 8.
5. For QC materials, label 20 QC-positive and 20 QC-negative tubes.
6. Dispense positive plasma/serum in 0.5 ml amounts into QC-positive tubes
7. Dispense negative plasma/serum in 0.5 ml amounts into QC-negative tubes

Additional supplies required

- 245 - 2 ml screw-capped (external thread) tubes (Wheaton, Corning, Nunc) 200 for unknown blood samples, 40 for QC materials and 5 for instructors)
- Labels, for labeling the QC as follows:

  QC Positive
  Lot No. 006150
  EXP 01-07

  Label "Negative" in the same way:
  The number and expiry dates are "made up" for in – house QC.
  Commercially prepared QC will have this detail on each vial.
- Pipettes to deliver 500µl.
- Test tube racks.
- 50 ml centrifuge tubes.

NB. Use different colored caps for positive QC material if possible. Wheaton tubes come with white caps and Corning tubes with orange caps. We used Corning tubes for positive QC samples and instructors tubes and placed the unknown blood samples in white-capped tubes. If you only have white-capped (or orange-capped) tubes, use a sharpie to color-code the caps of the positive QC material.
If you can obtain a "weak positive" HIV blood, include this as one of the positives.

For Lab session 1

Dispense 1.5 ml of QC positive plasma/serum into each of 5 tubes. Each instructor will have a tube to take to each participant and watch each person as they remove an aliquot with the pipette (Uni-Gold) and pipette two drops onto the testing device.

Laboratory preparation of student unknown specimens

There are many ways to prepare simulated positive specimens. The method described here is one that worked at CAREC for the workshop (Sept 21-23/2005)

Materials required:

1. HIV negative blood (quantity will depend on how many specimens you want to prepare)
2. HIV positive plasma
3. 1ml polypropylene tubes (Nunc – outside threaded tops*)
4. Pipettes and tips (to deliver 100-1000 ul)
5. Test tube racks.
6. Sterile 50 ml centrifuge tubes.

Method:

1. Check all plasma and blood with Determine, Uni-Gold and Stat-Pak (positive plasma at dilutions 1:2, 1:100, 1:200) rapid tests to verify that results are as expected.
2. Aliquot 0.5 ml of negative blood into tubes – this is your negative sample.
3. Spin down rest of negative blood in 15 ml tubes.
4. For a strong positive dilute negative plasma from one 50 ml tube 1:2 with positive plasma.
5. For a "weak" positive, choose one of the positive plasma samples that gave a "weak" reaction at 1:200 and dilute 1:25 with negative plasma in the 50 ml tube.
6. Dilute another positive plasma 1:10 with negative plasma in the 50 ml tube.

    - When you spin 15 ml blood you will get approximately 7-8 ml plasma. To make a 1:2 dilution and remove half of the negative plasma (reserve this) and replace it with positive plasma. Remix to resuspend red cells and aliquot in 0.5 ml amounts. Follow the procedure for the other positive samples (5 or 6).
    - It is easier to mix the blood in 50 ml tubes.

*External threads are a good biosafety feature when working with HIV infected blood.

Laboratory Practicals: Setup and Activities

Session 1 (Wednesday PM)

Performing rapid tests using Determine & Uni-Gold

1. There are two small plastic cups at your workstation. Pipette 10 drops of water from one to the other using the plastic pipette provided. Repeat as many times until you get used to dropping drops.

2. Observe the “carry over “demonstration with pipettes.

3. Observe demonstration of Determine rapid test and interpretation of test results.

4. Follow the SOP (on work bench) and perform Determine HIV rapid test.

5. Observe demonstration of Uni-Gold rapid test and interpretation of test results.

6. Follow the SOP (on work bench) and perform Uni-Gold HIV rapid test.

7. Discard all test materials and gloves into red bio-hazard bags provided.

8. Remove apron, fold and place on your chair.

9. Wash hands.

Each instructor will take a tube of HIV-positive blood to each participant during this session. The instructors will observe each person as s/he removes an aliquot with the pipette (Uni-Gold) and pipette two drops onto the testing devices (Determine and Uni-Gold).

NB Lab instructor:

1. Place two small plastic cups (fill one with water) and one Uni-Gold pipette at each workstation.

2. Place one Determine device and one Uni-Gold device and two Uni-Gold pipettes at each work station.

Laboratory Practical

Session 3 (Thursday AM)

Performing HIV Rapid Tests using QC samples

1. Observe demonstration of Stat-Pak HIV rapid test and interpretation of results.

2. Following the SOPs, set up Determine, Uni-Gold and Stat-Pak HIV rapid tests using a QC positive sample.

3. Following the SOPs, set up Determine, Uni-Gold and Stat-Pak HIV rapid tests using a QC negative sample.

4. Record results on QC sample log, recording all information (i.e filling out all columns on log sheet).

5. Discard all test materials and gloves into red bio-hazard bags provided.

6. Remove apron, fold and place on your chair.

7. Wash hands.

NB Lab instructor:

1. Place 2 testing devices of each Determine, Uni-Gold and Stat-Pak, 2 Uni-Gold pipettes, 2 Stat-Pak loops and one QC sample log at each work station.

2. Take out QC positive and negative samples for this lab.

3. Instruct participants to label their positive and negative QC tubes with an identifier. They will be required to use the same QC samples the next day.

Laboratory Practical

Session 4 (Thursday PM)

Performing HIV Rapid Tests using all three rapid test kits on samples 1- 5

1. Following the SOPs, set up Determine, Uni-Gold and Stat-Pak HIV rapid tests on samples 1-5.

2. Record results on Certification log, recording all information (i.e. filling out all columns on log sheet).

3. All results must be seen and initialed by one of the instructors

4. Discard all test materials and gloves into red bio-hazard bags provided.

5. Remove apron, fold and place on your chair.

6. Wash hands.

NB Lab instructor:

1. Take out unknown samples 1-5 for this lab

2. Place 5 testing devices of each Determine, Uni-Gold and Stat-Pak , 5 pipettes at each work station

Laboratory Practical

Session 5 (Friday AM)

Performing HIV Rapid Tests using the MOHTT Algorithm

1. Perform QC and record your results on the QC log
2. Using the MOHTT algorithm identify the HIV status on samples 6-10
3. Record your results on the certification log.
4. All results must be seen and initialed by one of the instructors
5. Discard all test materials and gloves and apron into red bio-hazard bags provided.
6. Wash hands.

NB Lab instructor:

1. Take out unknown samples 6-10 for this lab.
2. Take out QC samples.
3. Place 7 testing devices of each Determine, Uni-Gold, 2 each of Stat-Pak , 7 pipettes, and two Stat-Pak loops at each work station.
4. Have available on top table, Stat-Pak kits if required when testing according to the algorithm.

Instructor preparation of unknown samples and QC materials

1. For 20 participants:
    - HIV Positive blood - 80ml
    - HIV negative blood – 60ml
    - HIV positive plasma/serum – 30ml
    - HIV negative plasma/serum – 30ml.
2. Label tubes 1-10 for each participant
3. Dispense positive blood in 0.5 ml amounts into tubes 2, 3, 5, 6, 9, and 10.
4. Dispense negative blood in 0.5 ml amounts into tubes 1, 4, 7, and 8.
5. For QC materials, label 20 QC-positive and 20 QC-negative tubes.
6. Dispense positive plasma/serum in 0.5 ml amounts into QC-positive tubes
7. Dispense negative plasma/serum in 0.5 ml amounts into QC-negative tubes

Additional supplies required

- 245 - 2 ml screw-capped (external thread) tubes (Wheaton, Corning, Nunc) 200 for unknown blood samples, 40 for QC materials and 5 for instructors)
- Labels, for labeling the QC as follows:

  QC Positive
  Lot No. 006150
  EXP 01-07

  Label "Negative" in the same way:
  The number and expiry dates are "made up" for in – house QC.
  Commercially prepared QC will have this detail on each vial.
- Pipettes to deliver 500µl.
- Test tube racks.
- 50 ml centrifuge tubes.

NB. Use different colored caps for positive QC material if possible. Wheaton tubes come with white caps and Corning tubes with orange caps. We used Corning tubes for positive QC samples and instructors tubes and placed the unknown blood samples in white-capped tubes. If you only have white-capped (or orange-capped) tubes, use a sharpie to color-code the caps of the positive QC material.
If you can obtain a "weak positive" HIV blood, include this as one of the positives.

For Lab session 1

Dispense 1.5 ml of QC positive plasma/serum into each of 5 tubes. Each instructor will have a tube to take to each participant and watch each person as they remove an aliquot with the pipette (Uni-Gold) and pipette two drops onto the testing device.

Laboratory preparation of student unknown specimens

There are many ways to prepare simulated positive specimens. The method described here is one that worked at CAREC for the workshop (Sept 21-23/2005)

Materials required:

1. HIV negative blood (quantity will depend on how many specimens you want to prepare)
2. HIV positive plasma
3. 1ml polypropylene tubes (Nunc – outside threaded tops*)
4. Pipettes and tips (to deliver 100-1000 ul)
5. Test tube racks.
6. Sterile 50 ml centrifuge tubes.

Method:

1. Check all plasma and blood with Determine, Uni-Gold and Stat-Pak (positive plasma at dilutions 1:2, 1:100, 1:200) rapid tests to verify that results are as expected.
2. Aliquot 0.5 ml of negative blood into tubes – this is your negative sample.
3. Spin down rest of negative blood in 15 ml tubes.
4. For a strong positive dilute negative plasma from one 50 ml tube 1:2 with positive plasma.
5. For a "weak" positive, choose one of the positive plasma samples that gave a "weak" reaction at 1:200 and dilute 1:25 with negative plasma in the 50 ml tube.
6. Dilute another positive plasma 1:10 with negative plasma in the 50 ml tube.

    - When you spin 15 ml blood you will get approximately 7-8 ml plasma. To make a 1:2 dilution and remove half of the negative plasma (reserve this) and replace it with positive plasma. Remix to resuspend red cells and aliquot in 0.5 ml amounts. Follow the procedure for the other positive samples (5 or 6).
    - It is easier to mix the blood in 50 ml tubes.

*External threads are a good biosafety feature when working with HIV infected blood.

Laboratory Practicals: Setup and Activities

Session 1 (Wednesday PM)

Performing rapid tests using Determine & Uni-Gold

1. There are two small plastic cups at your workstation. Pipette 10 drops of water from one to the other using the plastic pipette provided. Repeat as many times until you get used to dropping drops.

2. Observe the “carry over “demonstration with pipettes.

3. Observe demonstration of Determine rapid test and interpretation of test results.

4. Follow the SOP (on work bench) and perform Determine HIV rapid test.

5. Observe demonstration of Uni-Gold rapid test and interpretation of test results.

6. Follow the SOP (on work bench) and perform Uni-Gold HIV rapid test.

7. Discard all test materials and gloves into red bio-hazard bags provided.

8. Remove apron, fold and place on your chair.

9. Wash hands.

Each instructor will take a tube of HIV-positive blood to each participant during this session. The instructors will observe each person as s/he removes an aliquot with the pipette (Uni-Gold) and pipette two drops onto the testing devices (Determine and Uni-Gold).

NB Lab instructor:

1\. Place two small plastic cups (fill one with water) and one Uni-Gold pipette at each workstation.

2\. Place one Determine device and one Uni-Gold device and two Uni-Gold pipettes at each work station.

Laboratory Practical

Session 3 (Thursday AM)

Performing HIV Rapid Tests using QC samples

1. Observe demonstration of Stat-Pak HIV rapid test and interpretation of results.

2. Following the SOPs, set up Determine, Uni-Gold and Stat-Pak HIV rapid tests using a QC positive sample.

3. Following the SOPs, set up Determine, Uni-Gold and Stat-Pak HIV rapid tests using a QC negative sample.

4. Record results on QC sample log, recording all information (i.e filling out all columns on log sheet).

5. Discard all test materials and gloves into red bio-hazard bags provided.

6. Remove apron, fold and place on your chair.

7. Wash hands.

NB Lab instructor:

1. Place 2 testing devices of each Determine, Uni-Gold and Stat-Pak, 2 Uni-Gold pipettes, 2 Stat-Pak loops and one QC sample log at each work station.

2. Take out QC positive and negative samples for this lab.

3. Instruct participants to label their positive and negative QC tubes with an identifier. They will be required to use the same QC samples the next day.

Laboratory Practical

Session 4 (Thursday PM)

Performing HIV Rapid Tests using all three rapid test kits on samples 1- 5

1. Following the SOPs, set up Determine, Uni-Gold and Stat-Pak HIV rapid tests on samples 1-5.

2. Record results on Certification log, recording all information (i.e. filling out all columns on log sheet).

3. All results must be seen and initialed by one of the instructors

4. Discard all test materials and gloves into red bio-hazard bags provided.

5. Remove apron, fold and place on your chair.

6. Wash hands.

NB Lab instructor:

1. Take out unknown samples 1-5 for this lab

2. Place 5 testing devices of each Determine, Uni-Gold and Stat-Pak , 5 pipettes at each work station

Laboratory Practical

Session 5 (Friday AM)

Performing HIV Rapid Tests using the MOHTT Algorithm

1. Perform QC and record your results on the QC log
2. Using the MOHTT algorithm identify the HIV status on samples 6-10
3. Record your results on the certification log.
4. All results must be seen and initialed by one of the instructors
5. Discard all test materials and gloves and apron into red bio-hazard bags provided.
6. Wash hands.

NB Lab instructor:

1. Take out unknown samples 6-10 for this lab.
2. Take out QC samples.
3. Place 7 testing devices of each Determine, Uni-Gold, 2 each of Stat-Pak , 7 pipettes, and two Stat-Pak loops at each work station.
4. Have available on top table, Stat-Pak kits if required when testing according to the algorithm.
