# Supplementary material for: Same-visit HIV testing in Trinidad and Tobago
Source: BMC Public Health. 2010 Apr 9;10:185. doi: 10.1186/1471-2458-10-185 (PMC2858728; doi:10.1186/1471-2458-10-185)
Supplement: Additional file 8 — Sample questions. This document lists sample test questions used to prepare exams required for tester certification. The question list was submitted to a web-based program which generated a random selection of test questions for each exam. Each exam included 25 questions. No two exams were identical. [file 1471-2458-10-185-S8.PDF]

Sample Test Questions

1. People who "Know Their Status" have had a diagnostic test for HIV infection.

True False

2. If you use a high quality rapid testing device, you can obtain a diagnostic quality HIV test with a *single* testing device.

True False

3. Which of the following work habits minimize exposure to biological hazards?

a) Changing gloves between clients
b) Washing hands between clients
c) Disposing of infectious materials as biohazardous waste
d) All of the above

4. Clients with negative diagnostic HIV rapid test ("same-visit") results should consider retesting in 3 months if they have participated in:

a) Unprotected oral, anal, or vaginal sex within 3 months prior to the present test
b) Needle-sharing within 3 months prior to the present test
c) a and b

5. If the results of the first two tests in the MOHTT testing algorithm are discordant, the next step in testing is:

a) Collect urine sample for confirmatory testing
b) Send a sample to the laboratory for confirmation
c) Test the client again using a Stat-Pak test
d) No additional testing is needed

6. Determine buffer can be used in the Uni-Gold assay.

True False

7. The most important advantage of "same visit" HIV testing is that many more of those tested learn the results of their tests. When testing is lab-based, people come for testing, and their samples are sent to labs, but then many people fail to return for their test results.

True False

8. What should you do when your first QC sample gives an invalid test result?

a) Repeat the test using a second device.
b) Collect urine sample for a confirmatory test.
c) Attempt to read the test after 2 hours.
d) Stop testing for the day

9. Infectious material must be handled using Universal Precautions.

True False

10. List the tests used in the MOH TT algorithm:

____

____

____

11. You can store food in the same refrigerator with the QC samples as long as the food is in a bag.

True False

12. You obtain the following results for the HIV-negative sample during the QC sample testing: Determine, invalid; Uni-Gold, negative; Stat-Pak, negative. What should you do next?

a) Record the results and run a second Determine test
b) Record the results and run a second Uni-Gold test
c) Record the results and run a second Stat-Pak test
d) Proceed with client testing

13. Client samples for MOHTT "same visit" HIV testing are whole blood.

True False

14. When should you repeat a test result which is invalid?

a) When you obtain this result with a client sample
b) When it is the result from your first set of daily QC samples
c) When it is the result from a retest of an QC sample

15. Discordant test results are:

a) The same as each other
b) Different from each other
c) The same as invalid results

16. The MOHTT HIV rapid test algorithm is:

a) Parallel and uses Uni-Gold and Stat-Pak as the first two tests.
b) Serial and uses OraQuick and Determine tests.
c) Parallel and uses Determine and Uni-Gold as the first two tests.

17. The MOHTT HIV rapid test algorithm uses:

a) Three tests run in parallel
b) Two tests in parallel to test a single blood sample
c) Whole blood samples obtained by veni-puncture
d) Determine, and OraQuick

18. When two tests results are concordant, they are:

a) Both positive
b) Both negative
c) Different from each other
d) Either (a) or (b)

19. An invalid test is:

a) No line seen in the control area of the device
b) Two lines seen on the testing device
c) One line seen on the testing device
d) The same as a discordant result

20. Use of retractable lancets to obtain client blood samples:

a) Minimizes risk of accidents at the testing site
b) Simplifies disposal of bio-hazardous waste
c) Reduces pain caused during blood collection
d) All of the above

21. When reading the test results:

a) Intensity of the red line is important
b) Presence of the internal control line is important
c) Absence of the internal control line is important
d) Both b and c

22. QC samples should be tested:

a) Each day testing is performed
b) By anyone who has time to test them
c) Whenever the tester feels it is time to test them

d) At any time it is convenient
e) By the person who performs client testing
f) Both a and e

23. QC samples must be stored:

a) In the refrigerator
b) With the rapid test kits
c) In the client testing room

24. Temperature records:

a) Should be kept for three areas: testing area, kit storage area, QC sample refrigerator.
b) Can be completed by anyone trained to keep them.
c) Must be completed by the person who does testing.
d) a and b
e) a and c
f) b and c

25. During site audits, Quality Monitors will be interested in:

a) Temperature records
b) Test result records
c) QC sample records
d) Inventory records
e) All of the above

26. Uni-Gold buffer should be used in the Uni-Gold test:

True False

27. Uni-Gold buffer can also be used in the Stat-Pak test:

True False

28. Stat-Pak buffer should be used in the Stat-Pak test:

True False

29. You obtain the following results for the HIV-negative sample during the QC sample testing: Determine, negative; Uni-Gold, negative; Stat-Pak, invalid. What should you do next?

a) Record all results and run another Determine test
b) Record all results and run another Uni-Gold test

c) Record all results and run another Stat-Pak test
d) Proceed with client testing

30. You obtain the following results for the HIV-positive sample during the QC sample testing: Determine, positive; Uni-Gold, positive; Stat-Pak, invalid. What should you do next?

a) Record all results and run another Determine test
b) Record all results and run another Uni-Gold test
c) Record all results and run another Stat-Pak test
d) Proceed with client testing

31. If the results of the first two tests (Determine and Uni-Gold) in the testing algorithm are discordant, the next step in testing is:

a) Use the third test to resolve the discordance
b) Collect urine sample for confirmatory testing
c) Send a sample to the laboratory for confirmation
d) Test the client again using a Stat-Pak test
e) No additional testing is needed
f) b and c
g) a and d

32. You have tested a client with a single Determine HIV Rapid Test. The test result is positive. This test result is all you need to provide a quality HIV status report to the client.

True False

33. Which of the following can cause incorrect or invalid test results?

a) Using rapid test kits which have been stored improperly.
b) Interpreting test results 30 minutes after starting the test.
c) Interpreting test result 2 minutes after starting test.
d) Using less blood than instructions require.
e) Using the same buffer on the first two tests.
f) All of the above.

34. Stat-Pak buffer can be used in the Determine assay.

True False

35. Concordant test results are:

a) The same as each other
b) Different from each other

c) The same as invalid results

36. You can your lunch in the same refrigerator with the QC samples as long as it is in a bag.

True False

37. The following results were obtained for the HIV-positive samples during the QC sample testing: Determine, positive; Uni-Gold, negative; Stat-Pak, positive. What should the tester do next?

a) Record all results and run another Determine test
b) Record all results and run another Uni-Gold test
c) Record all results and run another Stat-Pak test
d) Proceed with client testing

38. When using the MOH TT HIV rapid test algorithm, we perform:

a) One test after another
b) Two rapid tests at the same time
c) All three tests at the same time
d) More than one test with a single blood sample
e) a and c
f) b and d

39. When two tests results are discordant, they are:

a) Both positive
b) Both negative
c) Different from each other
d) Either (a) or (b)

40. Universal Safety Precautions apply when:

a) Only when testing the sample
b) When you are counseling the client
c) At all stages from collection to disposal

41. When reading the test results:

a) Intensity of the red line is important
b) Absence of the red line is important
c) Presence of the red line is important
d) Both b and c

42. One positive and one negative QC sample should be tested:

a) Before each client is tested
b) Each day testing is performed
c) At any time it is convenient
d) Both a and c

43. QC samples must be stored:

a) In the refrigerator
b) With the rapid test kits
c) In the client testing room

44. Temperature records:

a) Should be kept for three areas: testing area, kit storage area, QC sample refrigerator.
b) Can be completed by anyone trained to keep them.
c) Must be completed by the person who does testing.
d) a and b
e) a and c
f) b and c

45. Quality monitors will be interested in:

a) Cleanliness of your testing site
b) Cleanliness of your QC sample refrigerator
c) Status of your inventory of test kits
d) QC sample records
e) Temperature records
f) Al l of the above

46. Decontamination of a spill should be done using 10% bleach:

True False

47. When the temperature inside the QC sample refrigerator is 10° C, the person keeping temperature records should:

a) Not record the temperature because it is not 4° C.
b) Write down 10° C, because that is what the thermometer says.
c) Write down the 4° C because that is the correct temperature.
d) Report the 10° C to the site manager.
e) a and d
f) b and d

48. You accidentally spill your QC positive sample on your work space. Should you:

a) Wipe it off with a paper towel
b) Cover the spill with paper towels and then saturate the paper towels with 10% bleach.
c) Wipe up the spill and discard the paper towels.
d) Cover the spill with paper towels and then saturate the paper towels with 10% bleach. Wait 5 minutes and then wipe up the spill and discard the paper towels.

49. Retractable lancets are:

a) Safe because the blade is not exposed before or after use
b) Designed for single use
c) Both a and b

50. When you remove your gloves after attending to a client, do you:

a) Discard them into the general waste bin
b) Discard them into the bio-hazardous waste bag
c) Leave them in your client testing area

51. You can store bottled drinks in the QC sample refrigerator because they are in sealed containers.

True False

52. Uni-Gold tests require:

a) One drop of blood
b) Two drops of blood
c) One five-microliter loopful of blood

53. Determine tests require:

a) One drop of blood
b) Two drops of blood
c) One five-microliter loopful of blood

54. Stat-Pak tests require:

a) One drop of blood
b) Two drops of blood
c) One five-microliter loopful of blood

55. To make sure that you do not run out of kits, you should:

a) Check with your supplier frequently
b) Calculate maximum usage, minimum stock level plus lead time, and order appropriately
c) Order for six months

56. The MOHTT rapid test which includes pipettes packaged with the product is:

a) Uni-Gold
b) Determine
c) Ora-Quick

57. The first two tests in the MOHTT algorithm are:

a) Uni-Gold and Stat-Pak
b) Stat-Pak and Determine
c) Determine and Uni-Gold

58. The length of the blade in the retractable lancets used in the MOHTT HIV testing method is:

a) 0.5 mm
b) 1 mm
c) 2 mm

59. When you have results on your QC Sample Log, it is a record.

True False

60. The following results were obtained for the HIV-positive samples during the QC sample testing: Determine, positive; UniGold, positive; Stat Pak, invalid. After recording the results, what should the tester do next?

a) Record all results and run another Determine test
b) Record all results and run another Uni-Gold test
c) Record all results and run another Stat-Pak test
d) Proceed with client testing

61. Which of the following are documents:

a) Standard Operating Procedures (SOPs)
b) Signed certificate of workshop completion
c) Blank temperature chart
d) Manufacturer’s insert

e) a, b, and c
f) a, c, and d

62. When the temperature inside the QC sample refrigerator is 0° C, the person keeping temperature records should:

a) Not record the temperature because it is not 4° C.
b) Write down 0° C, because that is what the thermometer says.
c) Write down the 4° C because that is the correct temperature.

63. If the results of the first two tests in the MOHTT algorithm are discordant, the tester should:

a) Repeat both tests
b) Repeat the negative test
c) Repeat the positive test
d) Run the third test in the algorithm
e) None of the above

64. If the results of the first two tests in the MOHTT algorithm are concordant, the tester should:

a) Run the third test in the algorithm
b) Repeat the negative test
c) Repeat the positive test
d) Report the result to the client
e) All of the above

65. Which activities are included in Universal Safety Precautions?

a) Changing gloves between clients
b) Disposing of bio-hazardous waste properly
c) Keeping all objects out of your mouth
d) All of the above

66. Safety is important because:

a) Fewer accidents occur where good safety practices are observed
b) Clients feel more comfortable in testing sites where safety is practiced
c) Testers feel more confident when they are not worried about accidents during testing
d) All of the above

67. The third test in the MOHTT HIV testing algorithm is:

a) Determine

b) Stat-Pak
c) OraQuick
d) Uni-Gold

68. To be MOHTT-certified to provide HIV testing, testers must correctly interpret the HIV status of 50 samples under the supervision of an MOHTT certified tester.

True False

69. To be MOHTT-certified to provide HIV testing, testers must successfully complete a "same visit" HIV testing workshop.

True False

70. To be MOHTT-certified to provide HIV testing, testers must successfully identify all samples in an externally-provided HIV verification panel.

True False

71. How would you make a 10% bleach solution?

a) One part commercial bleach plus 10 parts of water
b) One part commercial bleach plus 9 parts water
c) One part commercial bleach plus 99 parts of water.

72. Select the correct answer:

a) One part commercial bleach plus 9 parts water = 10% solution
b) One part commercial bleach plus 10 parts water = 10% solution
c) One part commercial bleach plus 99 parts water = 1% solution

73. Positive and negative QC samples must be run each day of testing only with Determine and Uni-Gold because these are the two tests of the algorithm.

True False

74. Decontamination of an infectious spill should be done using a 1% bleach solution.

True False

75. The following results were obtained for the HIV-positive sample during the QC sample testing: Determine, negative; Uni-Gold, positive; Stat-Pak, positive. What should the tester do next?

a) Record the results and run a second Determine test
b) Record the results and run a second Uni-Gold test
c) Record the results and run a second Stat-Pak test
d) Proceed with client testing

76. The following results were obtained for the HIV-positive samples during the QC sample testing: Determine, positive; Uni-Gold, positive; Stat-Pak, negative. What should the tester do next?

a) Record the results and run a second Determine test
b) Record the results and run a second Uni-Gold test
c) Record the results and run a second Stat-Pak test
d) Proceed with client testing

77. You obtain the following results for the HIV-negative sample during the QC sample testing: Determine, negative; Uni-Gold, invalid; Stat-Pak, negative. What should you do next?

a) Record the results and run a second Uni-Gold test
b) Record the results and run a second Determine test
c) Record the results and run a second Stat-Pak test
d) Proceed with client testing

78. You obtain the following results for the HIV-negative sample during the QC sample testing: Determine, negative; Uni-Gold, negative; Stat-Pak, invalid. What should you do next?

a) Record the results and run a second Uni-Gold test
b) Record the results and run a second Determine test
c) Record the results and run a second Stat-Pak test
d) Proceed with client testing

79. Significant risk factors for HIV-infection include:

a) Unprotected oral, anal, or vaginal sex with partners of unknown HIV-status
b) Needle-sharing within 3 months prior to the present test
c) a and b

80. Clients prefer "same-visit" HIV testing because they only have to make one visit to a testing site to learn their HIV status.

True False

81. Stat-Pak buffer can be used in the Uni-Gold assay.

True False

82. Determine buffer should be used in the Determine assay.

True False

83. Rapid Test Kits should be stored:

a) Anywhere that is convenient
b) At a temperature of less than 28° C
c) In a locked storage space
d) Both a and b
e) Both b and c

84. Full certification by MOHTT for HIV-testing includes the following parts:

a) Successful completion of the HIV training workshop
b) Accurate interpretation of the HIV status of 50 samples
c) Accurate testing of a verification panel
d) All of the above

85. The following results were obtained for the HIV-positive samples during the QC sample testing: Determine, positive; Uni-Gold, positive; Stat-Pak, positive. What should the tester do next?

a) Record the results and run a second Determine test
b) Record the results and run a second Uni-Gold test
c) Record the results and run a second Stat-Pak test
d) Proceed with client testing

86. The following results were obtained for the HIV-negative samples during the QC sample testing: Determine, negative; Uni-Gold, negative; Stat-Pak, negative. What should the tester do next?

a) Record the results and run a second Determine test
b) Record the results and run a second Uni-Gold test
c) Record the results and run a second Stat-Pak test
d) Proceed with client testing
