# Supplementary material for: Same-visit HIV testing in Trinidad and Tobago
Source: BMC Public Health. 2010 Apr 9;10:185. doi: 10.1186/1471-2458-10-185 (PMC2858728; doi:10.1186/1471-2458-10-185)
Supplement: Additional file 9 — Cost of testing. This document presents costs associated with "same-visit" HIV testing. [file 1471-2458-10-185-S9.PDF]

April 2006

Supply Costs for MOHTT "Same-visit" HIV Testing

The following prices for rapid test kits, retractable lancets, gloves and alcohol swipes were obtained from the MOHTT Medical Supply Warehouse in April 2006. The price of QC materials was obtained from the import company which agreed to import the QC control prepared by BBI. All prices are given in TT$*.

The MOHTT protocol specifies that each tester must test one positive and one negative QC sample with each test in the algorithm on each day of testing. This means that each tester must use 6 HIV rapid tests for QC materials each day of testing. Thus the daily cost of testing per client varies according to the number of clients tested. The number of clients tested by each tester each day can vary between 1 and 10.

Supply list:

Determine HIV rapid test	15.75
Uni-Gold HIV rapid test	13.53
Stat-Pak HIV rapid test	16.25
Retractable Lancets	2.10
Alcohol Swipes	.08
Gloves	.33
Total	48.04
QC materials for one day	15.00

Cost of QC testing per day = 45.53 x 2 + 15.00 = TT$ 106.06 (US$ 17.67)

Cost of testing one client with Determine and Uni-Gold = TT$ 31.79 (US$5.30)

Daily testing costs *per client* including QC:

For one client:
$TT 106.06 plus 31.79 is TT$ 169.64 (US$ 26.93)

For ten clients:
$TT 106.06 plus 317.90 is TT$ 423.96 divided by 10 is TT$ 42.40 (US$ 6.73)

*One US dollar is approximately six TT dollars. Divide all $TT values by 6 for approximate $US values.
